# Supplementary material for: Ni-catalyzed regio- and stereo-defined intermolecular cross-electrophile dialkylation of alkynes without directing group
Source: Nat Commun. 2021 Feb 10;12:928. doi: 10.1038/s41467-021-21083-w (PMC7876002; doi:10.1038/s41467-021-21083-w)
Supplement: Supplementary file 1 — Supplemtary Information [file 41467_2021_21083_MOESM1_ESM.pdf]

Ni-Catalyzed Regio- and Stereo-defined Intermolecular Cross-Electrophile Dialkylation of Alkynes Without Directing Group

Yi-Zhou Zhan,† Nan Xiao,† and Wei Shu*,†

†*Shenzhen Grubbs Institute, Department of Chemistry, and Guangdong Provincial Key Laboratory of Catalytic Chemistry, Southern University of Science and Technology, Shenzhen 518055, Guangdong, P.R. China*

*E-mail: shuw@sustech.edu.cn

Table of Contents

1. Supplementary Note 1 .... S2

2. Supplementary Methods, Tables and Figures S2

2.1 General procedures S2

2.2 Characterization of new compounds S4

2.3 Control and mechanistic experiments S31

2.4 ^{1}H, ^{13}C, and ^{19}F spectra of new compounds S50

2.5 X-ray diffraction data of 5c and 6b S126

3. Supplementary References S130

1. Supplementary Note 1

General information

NMR spectra were recorded on 400 MHz or 600 MHz Bruker spectrometers. Chemical shifts are given in ppm. The spectra are calibrated to the residual ^{1}H and ^{13}C signals of the solvents. Multiplicities are abbreviated as follows: singlet (s), doublet (d), triplet (t), quartet (q), doublet-doublet (dd), quintet (quint), septet (sept), multiplet (m), and broad (br). High-resolution electrospray ionization and electronic impact mass spectrometry was performed on a Finnigan MAT 900 (Thermo Finnigan, San Jose, CA; USA) double focusing magnetic sector mass spectrometer. A mass accuracy ≤ 2 ppm was obtained in the peak matching acquisition mode by using a solution containing 2 <l PEG200, 2 <l PPG450, and 1.5 mg NaOAc (all obtained from Sigma-Aldrich, CH-Buchs) dissolved in 100 mL MeOH (HPLC Supra grade, Scharlau, E-Barcelona) as internal standard.

Materials and Methods All reactions were carried out under an inert atmosphere of nitrogen in an oven dried or flame dried glassware with magnetic stirring. Unless otherwise stated, starting materials were purchased from Aladdin (1-bromobutane, KI, *t*-BuOH, pentafluoropyridine), Aldrich (*t*-BuOD), TCI (anhydrous $CeCl_3$), Macklin Reagent (N,N-dimethylformamide, 1-hexyne), Adamas-beta (phenylacetylene, 2-cyclohexen-1-one), Bide Pharm (4-cyanopyridine), Yishi Chemical Industry ($NiBr_2$·DME), J&K Chemicals and Jiuding chemistry. $CeCl_3$ was further dehydrated by a heat gun under reduced pressure. Solvents were purchased in HPLC quality, degassed by purging with nitrogen and dried over activated molecular sieves of appropriatesize. DMF was handled by vacuum distillation after dried over CaH_2. Conversion was monitored by thin layer chromatography (TLC) using Merck TLC silica gel 60 F254. Compounds were visualized by UV light at 254 nm and by dipping the plates in an ethanolic phosphomolybdic acid solution or an aqueous potassium permanganate solution followed by heating. Flashcolumn chromatography was performed over silica gel (300-400 mesh).

2. Supplementary Methods, Tables and Figures

2.1 General procedures

General procedure for Conditions A:

The reaction was operated in a nitrogen-filled glove box. 4-Cyanopyridine (5 mol%), zinc (4.5 equiv), $CeCl_3$ (0.8 equiv), KI (2.0 equiv), $NiBr_2$·DME (10 mol%), alkyne (2.0 equiv, if solid), and alkyl bromide (2.0 equiv, if solid) were added into an oven-dried vial containing a magnetic stirring bar. If the alkyne or alkyl bromide is a solid, it was added along with other solid reagents.

Anhydrous DMF (0.1 M) was added and rapid stirring was commenced. Then H_2O (1.5 equiv), alkyne (2.0 equiv, if liquid), enone (0.2 mmol) and alkyl bromide (2.0 equiv, if liquid, in the case of **7s**, alkyl iodide was used) were added sequentially via syringe. The vial was sealed with parafilm and stirred vigorously for 48 h. The reaction was diluted with ethyl acetate (30 mL) and washed with brine (50 mL). The aqueous layer was extracted twice with ethyl acetate (20 mL). The combined organic layer was dried over magnesium sulfate, filtered, evaporated and purified by silica gel chromatography with hexanes: ethyl acetate as eluent to give the corresponding product in pure form.

General procedure for Conditions B:

The reaction was operated in a nitrogen-filled glove box. Zinc (2.5 equiv), $ZnBr_2$ (0.1 equiv), KI (2.0 equiv), $NiBr_2{\cdot}DME$ (10 mol%), alkyne (2.0 equiv, if solid), and alkyl bromide (2.0 equiv, if solid) were added to an oven-dried vial containing a magnetic stirring bar. Anhydrous DMF (0.1 M) was added and rapid stirring was commenced. Then pentafluoropyridine (20 mol%), 2-methyl-2-butanol (2.0 equiv), alkyne (2.0 equiv, if liquid), enone (0.2 mmol) and alkyl bromide (2.0 equiv, if liquid) were added sequentially via syringe. The vial was sealed with parafilm and stirred vigorously for 24 h. The reaction was diluted with ethyl acetate (30 mL) and washed with brine (50 mL). The aqueous layer was extracted twice with ethyl acetate (20 mL). The combined organic layer was dried over magnesium sulfate, filtered, evaporated and purified by silica gel chromatography with hexanes: ethyl acetate as eluent to give the corresponding product in pure form.

General procedure for Conditions C:

The reaction was operated in a nitrogen-filled glove box. Zinc (5.0 equiv), ZnI_2 (0.3 equiv), CsI (5.0 equiv), $NiBr_2{\cdot}DME$ (10 mol%), alkyne (2.8 equiv, if solid), and alkyl bromide (3.5 equiv, if solid) were added to an oven-dried vial containing a magnetic stirring bar. Anhydrous DMF (0.05 M) was added and rapid stirring was commenced. Then 2-methyl-2-butanol (3.0 equiv), alkyne (2.8 equiv, if liquid), enone (0.2 mmol) and alkyl bromide (3.5 equiv, if liquid) were added sequentially via syringe. The vial was sealed with parafilm and stirred vigorously for 24 h. The reaction was diluted with ethyl acetate (30 mL) and washed with brine (50 mL). The aqueous layer was extracted twice with ethyl acetate (20 mL). The combined organic layer was dried over magnesium sulfate, filtered, evaporated and purified by silica gel chromatography with hexanes: ethyl acetate as eluent to give the corresponding product in pure form.

General procedure for gram-scale synthesis:

The reaction was operated in a nitrogen-filled glove box. 4-Cyanopyridine (52 mg, 5 mol%), zinc (2.94 g, 4.5 equiv), $CeCl_3$ (1.96 g, 0.8 equiv), KI (3.32 g, 2.0 equiv) and $NiBr_2{\cdot}DME$ (0.31

g, 10 mol%) were added to an oven-dried 250 mL Schlenk flask containing a magnetic stirring bar. Anhydrous DMF (100 mL, 0.1 M) was added and rapid stirring was commenced. Then H_2O (27 µL, 1.5 equiv), 1-hexyne (2.29 mL, 2.0 equiv), 2-cyclohexen-1-one (0.96 mL, 10 mmol) and 1-bromobutane (2.28 mL, 2.0 equiv) were added sequentially via syringe. The flask was sealed with parafilm and stirred vigorously for 48 h. The reaction mixture was filtered through a plug of celite and washed with ethyl acetate. The organic layers were diluted with ethyl acetate (300 mL) and washed with brine (200 mL), aqueous layer was extracted twice with ethyl acetate (200 mL). The combined organic layer was dried over magnesium sulfate, evaporated and purified by silica gel chromatography with hexanes: ethyl acetate mixtures as eluent (gradient from 0-2%) to give **4a** (1.68 g, 67% yield) as a yellow oil.

General procedure for synthesis of unfunctionalized tri- and tetrasubstituted alkenes:

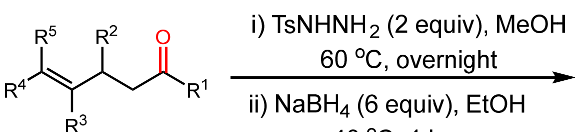

9

Under nitrogen, ketone product (0.1 mmol) and $TsNHNH_2$ (0.2 mmol, 37.2 mg) were dissolved in dry MeOH (2.5 mL). The mixture was stirred at 60 °C until the ketone was completely consumed (monitored by TLC). After completion, MeOH was evaporated under reduced pressure. The residue was dissolved in dry EtOH (2.5 mL) without separation and $NaBH_4$ (0.6 mmol, 22.7 mg) was added. The resulting mixture was stirred at 40 °C for 1 h. After completion, EtOH was evaporated under reduced pressure and the residue was purified by flash chromatography to give the corresponding product.

2.2 Characterization of new compounds

3-(2-butylhex-1-en-1-yl)cyclohexan-1-one (4a)

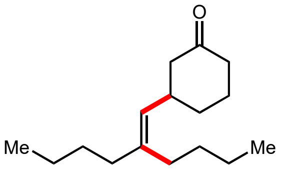

Following general procedure for Conditions A, the crude mixure was purified by flash chromatography on silica gel (eluted with ethyl acetate: hexanes = 1% ~ 2%) to give the product **4a** (30.2 mg, 64% yield) as a colorless oil. ^{1}H NMR (600 MHz, $CDCl_3$) δ 4.96 (d, J = 9.2 Hz, 1H), 2.72 – 2.63 (m, 1H), 2.37 – 2.32 (m, 1H), 2.32 – 2.28 (m, 1H), 2.27 – 2.22 (m, 1H), 2.13 – 2.07 (m, 1H), 2.07 – 2.02 (m, 1H), 1.98 – 1.92 (m, 4H), 1.80 – 1.75 (m, 1H), 1.73 – 1.64 (m, 2H), 1.47 – 1.40 (m, 1H), 1.36 – 1.31 (m, 3H), 1.31 – 1.26 (m, 4H), 0.88 (t, J = 7.2 Hz, 6H); ^{13}C NMR (151 MHz, $CDCl_3$) δ 211.79, 140.03, 127.73, 48.62, 41.32, 38.02, 36.39, 32.30, 31.01, 30.39, 30.05, 25.45, 22.92, 22.48, 14.10, 14.08; HR-MS (ESI) m/z calcd for $C_{16}H_{29}O$[M + H$^+$]: 237.2213, found: 237.2211.

(*E*)-3-(2-butyloct-1-en-1-yl)cyclohexan-1-one (4b)

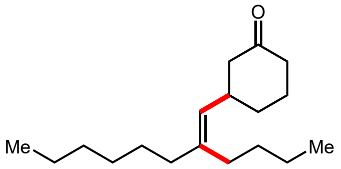

Following general procedure for Conditions A, the crude mixture was purified by flash chromatography on silica gel (eluted with ethyl acetate : hexanes = 1% ~ 2%) to give the product **4b** (33.8 mg, 64% yield) as a colorless oil. ^{1}H NMR (400 MHz, $CDCl_3$) δ 4.95 (d, *J* = 9.3 Hz, 1H), 2.76 – 2.61 (m, 1H), 2.39 – 2.29 (m, 2H), 2.28 – 2.20 (m, 1H), 2.14 – 2.00 (m, 2H), 1.99 – 1.90 (m, 4H), 1.81 – 1.73 (m, 1H), 1.72 – 1.66 (m, 1H), 1.49 – 1.41 (m, 1H), 1.40 – 1.24 (m, 12H), 0.88 (q, *J* = 6.8 Hz, 6H); ^{13}C NMR (101 MHz, $CDCl_3$) δ 211.83, 140.04, 127.74, 48.63, 41.32, 38.03, 36.67, 32.30, 31.83, 31.00, 30.04, 29.06, 28.11, 25.46, 22.92, 22.73, 14.17, 14.11; HR-MS (ESI) m/z calcd for $C_{18}H_{33}O[M + H^+]$: 265.2526, found: 265.2524.

(*E*)-3-(2-phenethylhex-1-en-1-yl)cyclohexan-1-one (4c)

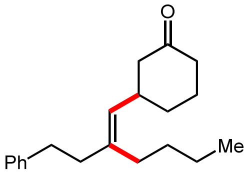

Following general procedure for Conditions A, the crude mixture was purified by flash chromatography on silica gel (eluted with ethyl acetate : hexanes = 1% ~ 2%) to give the product **4c** (39.8 mg, 70% yield) as a colorless oil. ^{1}H NMR (400 MHz, $CDCl_3$) δ 7.35 – 7.27 (m, 2H), 7.25 – 7.13 (m, 3H), 4.98 (d, *J* = 9.3 Hz, 1H), 2.79 – 2.66 (m, 3H), 2.43 – 2.36 (m, 1H), 2.36 – 2.23 (m, 4H), 2.14 – 2.02 (m, 4H), 1.85 – 1.67 (m, 3H), 1.51 – 1.32 (m, 5H), 0.95 (t, *J* = 7.0 Hz, 3H); ^{13}C NMR (101 MHz, $CDCl_3$) δ 211.71, 142.20, 138.91, 128.62, 128.51, 128.26, 125.77, 48.42, 41.28, 38.49, 37.98, 34.75, 32.12, 30.98, 30.15, 25.42, 22.88, 14.10; HR-MS (ESI) m/z calcd for $C_{20}H_{29}O[M + H^+]$: 285.2213, found: 285.2212.

(*E*)-3-(2-(3-chloropropyl)hex-1-en-1-yl)cyclohexan-1-one (4d)

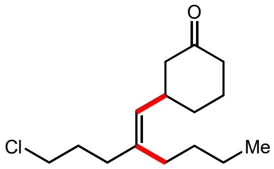

Following general procedure for Conditions A, the crude mixture was purified by flash chromatography on silica gel (eluted with ethyl acetate : hexanes = 1% ~ 2%) to give the product **4d** (25.2 mg, 49% yield) as a colorless oil. ^{1}H NMR (600 MHz, $CDCl_3$) δ 5.02 (d, *J* = 9.3 Hz, 1H), 3.49 (t, *J* = 6.6 Hz, 2H), 2.73 – 2.64 (m, 1H), 2.38 – 2.22 (m, 3H), 2.13 – 2.08 (m, 3H), 2.08 – 2.02 (m, 1H), 1.97 (t, *J* = 7.4 Hz, 2H), 1.87 – 1.81 (m, 2H), 1.80 – 1.74 (m, 1H), 1.73 – 1.68 (m, 1H), 1.49 – 1.41 (m, 1H), 1.34 – 1.24 (m, 4H), 0.89 (t, *J* = 6.9 Hz, 3H); ^{13}C NMR (151 MHz, $CDCl_3$) δ 211.48, 138.08, 129.09, 48.43, 44.60, 41.26, 38.01, 33.61, 32.17, 30.96, 30.92, 29.95, 25.40, 22.85, 14.06; HR-MS (ESI) m/z calcd for $C_{15}H_{26}ClO[M + H^+]$: 257.1667, found: 257.1665.

(*E*)-4-((3-oxocyclohexyl)methylene)octyl acetate (4e)

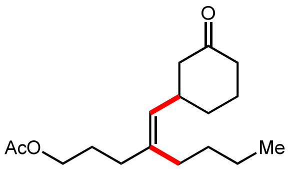

Following general procedure for Conditions A, the crude mixture was purified by flash chromatography on silica gel (eluted with ethyl acetate : hexanes = 1% ~ 2%) to give the product **4e** (29.7 mg, 53% yield) as a colorless oil. ^{1}H NMR (600 MHz, $CDCl_3$) δ 4.98 (d, *J* = 9.3 Hz, 1H), 4.02 (t, *J* = 6.7 Hz, 2H), 2.72 – 2.62 (m, 1H), 2.38 – 2.30 (m, 1H), 2.32 – 2.26 (m, 1H), 2.28 – 2.20 (m, 1H), 2.15 – 2.04 (m, 2H), 2.03 (s, 3H), 2.02 – 1.95 (m, 4H), 1.79 – 1.73 (m, 1H), 1.74 – 1.63 (m, 3H), 1.48 – 1.38 (m, 1H), 1.34 – 1.23 (m, 4H), 0.88 (t, *J* = 7.0 Hz, 3H); ^{13}C NMR (151 MHz, $CDCl_3$) δ 211.33, 171.10, 138.53, 128.53, 64.07, 48.36, 41.19, 37.91, 32.69, 32.12, 30.85, 29.97, 27.01, 25.32, 22.78, 20.95, 13.97; HR-MS (ESI) m/z calcd for $C_{17}H_{29}O_3$[M + H$^+$]: 281.2111, found: 281.2110.

(*E*)-N,N-dibenzyl-5-((3-oxocyclohexyl)methylene)nonanamide (4f)

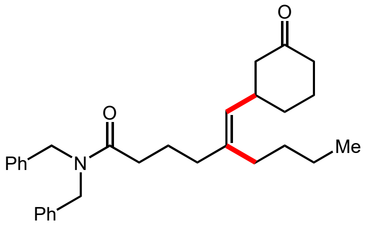

Following general procedure for Conditions A, the crude mixture was purified by flash chromatography on silica gel (eluted with ethyl acetate : hexanes = 1 : 40) to give the product **4f** (53.5 mg, 60% yield) as a colorless oil. ^{1}H NMR (600 MHz, $CDCl_3$) δ 7.29 (t, *J* = 7.5 Hz, 2H), 7.27 – 7.18 (m, 4H), 7.17 – 7.13 (m, 2H), 7.07 (d, *J* = 7.5 Hz, 2H), 4.88 (d, *J* = 9.2 Hz, 1H), 4.54 (s, 2H), 4.36 (s, 2H), 2.62 – 2.52 (m, 1H), 2.30 (t, *J* = 7.6 Hz, 2H), 2.28 – 2.23 (m, 1H), 2.19 – 2.12 (m, 2H), 1.98 – 1.91 (m, 4H), 1.87 (t, *J* = 7.3 Hz, 2H), 1.76 – 1.70 (m, 2H), 1.64 – 1.54 (m, 2H), 1.33 – 1.25 (m, 1H), 1.24 – 1.16 (m, 4H), 0.81 (t, *J* = 6.8 Hz, 3H); ^{13}C NMR (151 MHz, $CDCl_3$) δ 211.49, 173.54, 139.02, 137.56, 136.64, 129.00, 128.63, 128.61, 128.35, 127.64, 127.43, 126.34, 49.94, 48.42, 48.25, 41.23, 37.95, 36.02, 32.55, 32.14, 30.92, 29.93, 25.40, 23.67, 22.85, 14.06; HR-MS (ESI) m/z calcd for $C_{30}H_{40}NO_2$[M + H$^+$]: 446.3054, found: 446.3050.

dimethyl (*E*)-2-(3-((3-oxocyclohexyl)methylene)heptyl)malonate (4g)

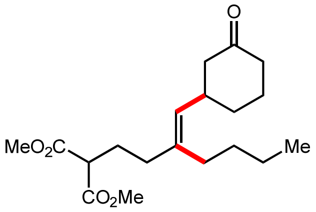

Following general procedure for Conditions A, the crude mixture was purified by flash chromatography on silica gel (eluted with ethyl acetate : hexanes = 1 : 30) to give the product **4g** (27.1 mg, 40% yield) as a colorless oil. ^{1}H NMR (400 MHz, $CDCl_3$) δ 4.98 (d, *J* = 9.3 Hz, 1H), 3.73 (d, *J* = 1.2 Hz, 6H), 3.37 – 3.29 (m, 1H), 2.73 – 2.61 (m, 1H), 2.39 – 2.20 (m, 3H), 2.15 – 2.02 (m, 2H), 2.01 – 1.92 (m, 6H), 1.82 – 1.69 (m, 1H), 1.49 – 1.37 (m, 1H), 1.33 – 1.22 (m, 5H), 0.87 (t, *J* = 6.8 Hz, 3H); ^{13}C NMR (101 MHz, $CDCl_3$) δ

211.50, 169.92, 169.90, 138.03, 129.43, 52.59, 51.04, 48.38, 41.26, 38.00, 34.03, 32.13, 30.84, 29.79, 27.29, 25.40, 22.83, 14.07; HR-MS (ESI) m/z calcd for $C_{19}H_{31}O_5[M + H^+]$: 339.2166, found: 339.2164.

adamantan-1-ylmethyl (*E*)-5-((3-oxocyclohexyl)methylene)nonanoate (4h)

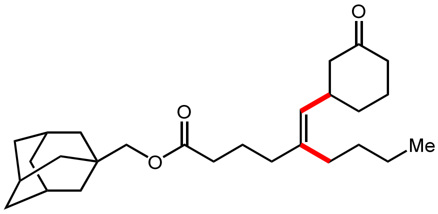

Following general procedure for Conditions A, the crude mixture was purified by flash chromatography on silica gel (eluted with ethyl acetate : hexanes = 1 : 40) to give the product **4h** (41.5 mg, 50% yield) as a colorless oil. ^{1}H NMR (600 MHz, $CDCl_3$) δ 4.99 (d, *J* = 9.2 Hz, 1H), 3.67 (s, 2H), 2.72 – 2.64 (m, 1H), 2.39 – 2.33 (m, 1H), 2.33 – 2.22 (m, 4H), 2.13 – 2.07 (m, 1H), 2.07 – 2.03 (m, 1H), 2.02 – 1.95 (m, 7H), 1.81 – 1.75 (m, 1H), 1.75 – 1.67 (m, 6H), 1.66 – 1.64 (m, 2H), 1.64 – 1.62 (m, 1H), 1.53 (d, *J* = 2.9 Hz, 6H), 1.48 – 1.40 (m, 1H), 1.33 – 1.24 (m, 4H), 0.89 (t, *J* = 7.0 Hz, 3H); ^{13}C NMR (151 MHz, $CDCl_3$) δ 211.55, 173.96, 138.86, 128.81, 73.99, 48.52, 41.29, 39.39, 38.04, 37.04, 35.98, 33.84, 33.26, 32.25, 30.97, 29.94, 28.12, 25.45, 23.49, 22.90, 14.08; HR-MS (ESI) m/z calcd for $C_{27}H_{42}O_3Na[M + Na^+]$: 437.3026, found: 437.3019.

(8R,9S,13S,14S)-13-methyl-3-(((*E*)-4-(((3-oxocyclohexyl)methylene)octyl)oxy)-6,7,8,9,11,12,13,14,15,16-decahydro-17*H*-cyclopenta[*a*]phenanthren-17-one (4i)

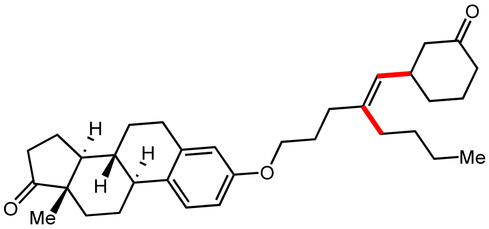

Following general procedure for Conditions A, the crude mixture was purified by flash chromatography on silica gel (eluted with ethyl acetate : hexanes = 1 : 40) to give the product **4i** (51.0 mg, 52% yield) as a colorless oil. ^{1}H NMR (600 MHz, $CDCl_3$) δ 7.19 (d, *J* = 8.6 Hz, 1H), 6.69 (dd, *J* = 8.6, 2.7 Hz, 1H), 6.62 (d, *J* = 2.7 Hz, 1H), 5.10 (d, *J* = 9.3 Hz, 1H), 3.97 (t, *J* = 7.0 Hz, 2H), 2.93 – 2.84 (m, 2H), 2.75 – 2.67 (m, 1H), 2.50 (dd, *J* = 19.1, 8.7 Hz, 1H), 2.43 (t, *J* = 7.0 Hz, 2H), 2.41 – 2.34 (m, 2H), 2.34 – 2.30 (m, 1H), 2.30 – 2.22 (m, 2H), 2.17 – 2.10 (m, 2H), 2.09 – 1.98 (m, 5H), 1.97 – 1.92 (m, 1H), 1.83 – 1.77 (m, 1H), 1.74 – 1.66 (m, 2H), 1.64 – 1.57 (m, 2H), 1.56 – 1.38 (m, 6H), 1.36 – 1.25 (m, 4H), 0.92 – 0.88 (m, 6H); ^{13}C NMR (151 MHz, $CDCl_3$) δ 221.03, 211.45, 156.96, 137.78, 136.22, 132.08, 130.19, 126.36, 114.65, 112.25, 66.98, 50.47, 48.37, 48.08, 44.05, 41.27, 38.43, 38.03, 36.23, 35.94, 32.12, 31.64, 30.93, 30.44, 29.72, 26.62, 25.98, 25.39, 22.84, 21.65, 14.08, 13.92; HR-MS (ESI) m/z calcd for $C_{33}H_{47}O_3[M + H^+]$: 491.3520, found: 491.3517.

(*E*)-4-((3-oxocyclohexyl)methylene)octyl 3-(4,5-diphenyloxazol-2-yl)propanoate (4j)

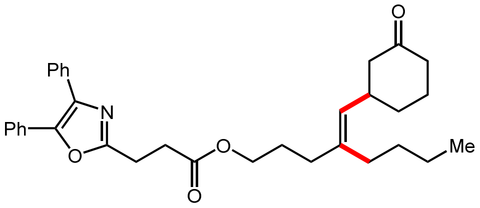

Following general procedure for Conditions A, the crude mixture was purified by flash chromatography on silica gel (eluted with ethyl acetate : hexanes = 1 : 40) to give the product **4j** (58.6 mg, 57% yield) as a colorless oil. ^{1}H NMR (400 MHz, $CDCl_3$) δ 7.66 – 7.60 (m, 2H), 7.59 – 7.54 (m, 2H), 7.40 – 7.28 (m, 6H), 4.96 (d, *J* = 9.2 Hz, 1H), 4.10 (t, *J* = 6.6 Hz, 2H), 3.19 (t, *J* = 7.5 Hz, 2H), 2.92 (t, *J* = 7.5 Hz, 2H), 2.72 – 2.60 (m, 1H), 2.39 – 2.18 (m, 3H), 2.10 – 1.92 (m, 6H), 1.79 – 1.61 (m, 4H), 1.47 – 1.35 (m, 1H), 1.33 – 1.23 (m, 4H), 0.88 (t, *J* = 7.0 Hz, 3H); ^{13}C NMR (101 MHz, $CDCl_3$) δ 211.52, 172.06, 161.86, 145.48, 138.45, 135.15, 132.46, 129.01, 128.74, 128.72, 128.62, 128.54, 128.15, 127.95, 126.52, 64.46, 48.42, 41.26, 38.00, 32.70, 32.16, 31.24, 30.92, 29.97, 27.04, 25.43, 23.64, 22.85, 14.08; HR-MS (ESI) m/z calcd for $C_{33}H_{40}O_4$[M + H$^+$]: 514.2952, found: 514.2947.

(*E*)-3-(2-phenylhex-1-en-1-yl)cyclohexan-1-one (5a)

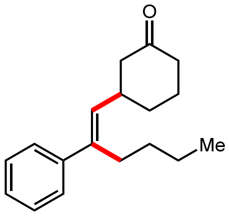

Following general procedure for Conditions B, the crude mixture was purified by flash chromatography on silica gel (eluted with ethyl acetate : hexanes = 1% ~ 2%) to give the product **5a** (30.8 mg, 60% yield) as a colorless oil. ^{1}H NMR (400 MHz, $CDCl_3$) δ 7.28 – 7.20 (m, 4H), 7.18 – 7.14 (m, 1H), 5.39 (d, *J* = 9.4 Hz, 1H), 2.84 – 2.74 (m, 1H), 2.47 – 2.30 (m, 4H), 2.29 – 2.20 (m, 1H), 2.20 – 2.10 (m, 1H), 2.08 – 2.00 (m, 1H), 1.86 – 1.80 (m, 1H), 1.75 – 1.63 (m, 1H), 1.54 – 1.44 (m, 1H), 1.24 – 1.18 (m, 4H), 0.77 (t, *J* = 7.2 Hz, 3H); ^{13}C NMR (101 MHz, $CDCl_3$) δ 211.25, 142.78, 140.52, 131.35, 128.27, 126.93, 126.52, 48.19, 41.29, 38.70, 32.04, 31.14, 29.82, 25.43, 22.72, 14.00; HR-MS (ESI) m/z calcd for $C_{18}H_{25}O$[M + H$^+$]: 257.1900, found: 257.1898.

(*E*)-3-(2-(*p*-tolyl)hex-1-en-1-yl)cyclohexan-1-one (5b)

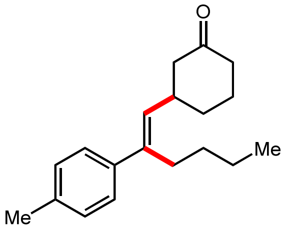

Following general procedure for Conditions B with slight modification: After 5 h, 1.0 equiv alkyne was added. The crude mixture was purified by flash chromatography on silica gel (eluted with ethyl acetate : hexanes = 1% ~ 2%) to give the product **5b** (36.8 mg, 68% yield) as a colorless oil. ^{1}H NMR (400 MHz, $CDCl_3$) δ 7.22 (d, J = 8.2 Hz, 2H), 7.12 (d, *J* = 8.2 Hz, 2H), 5.44 (d, *J* = 9.4 Hz, 1H), 2.90 – 2.80 (m, 1H), 2.48 – 2.37 (m, 4H), 2.34 (s, 3H), 2.32 – 2.25 (m, 1H), 2.25 – 2.17 (m, 1H), 2.15 – 2.07 (m, 1H), 1.94 – 1.86 (m,

1H), 1.82 – 1.70 (m, 1H), 1.61 – 1.51 (m, 1H), 1.30 – 1.26 (m, 4H), 0.84(t, *J* = 7.2 Hz, 3H); ^{13}C NMR (101 MHz, $CDCl_3$) δ 211.34, 140.30, 139.86, 136.64, 130.71, 129.00, 126.38, 48.28, 41.33, 38.72, 32.10, 31.19, 29.80, 25.45, 22.75, 21.12, 14.02; HR-MS (ESI) m/z calcd for $C_{19}H_{27}O$[M + H^+]: 271.2056, found: 271.2055.

(*E*)-3-(2-(4-methoxyphenyl)hex-1-en-1-yl)cyclohexan-1-one (5c)

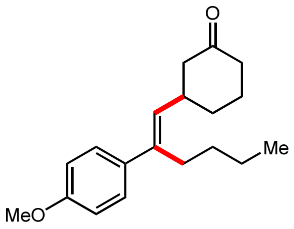

Following general procedure for Conditions B with slight modification: After 3 h, 1.0 equiv of alkyne was added. The crude mixture was purified by flash chromatography on silica gel (eluted with ethyl acetate : hexanes = 1% ~ 2%) to give the product **5c** (41.2 mg, 72% yield) as a colorless oil. ^{1}H NMR (400 MHz, $CDCl_3$) δ 7.25 (d, *J* = 8.8 Hz, 2H), 6.84 (d, *J* = 8.8 Hz, 2H), 5.40 (d, *J* = 9.3 Hz, 1H), 3.80 (s, 3H), 2.90 – 2.78 (m, 1H), 2.48 – 2.37 (m, 4H), 2.35 – 2.25 (m, 1H), 2.25 – 2.17 (m, 1H), 2.15 – 2.06 (m, 1H), 1.92 – 1.85 (m, 1H), 1.82 – 1.68 (m, 1H), 1.58 – 1.50 (m, 1H), 1.31 – 1.26 (m, 4H), 0.84(t, *J* = 6.8 Hz, 3H); ^{13}C NMR (101 MHz, $CDCl_3$) δ 211.36, 158.73, 139.83, 135.19, 130.10, 127.53, 113.65, 55.34, 48.33, 41.33, 38.73, 32.15, 31.20, 29.78, 25.45, 22.74, 14.03; HR-MS (ESI) m/z calcd for $C_{19}H_{27}O_2$[M + H^+]: 287.2006, found: 287.2003.

(*E*)-3-(2-(4-(*tert*-butyl)phenyl)hex-1-en-1-yl)cyclohexan-1-one (5d)

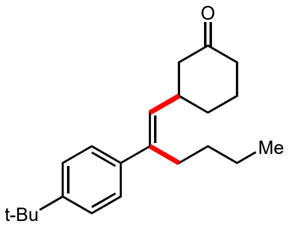

Following general procedure for Conditions B, the crude mixture was purified by flash chromatography on silica gel (eluted with ethyl acetate : hexanes = 1% ~ 2%) to give the product **5d** (37.5 mg, 60% yield) as a colorless oil. ^{1}H NMR (400 MHz, $CDCl_3$) δ 7.28 – 7.15 (m, 4H), 5.40 (d, *J* = 9.4 Hz, 1H), 2.85 – 2.72 (m, 1H), 2.43 – 2.29 (m, 4H), 2.28 – 2.19 (m, 1H), 2.19 – 2.09 (m, 1H), 2.08 – 1.98 (m, 1H), 1.87 – 1.77 (m, 1H), 1.75 – 1.62 (m, 1H), 1.55 – 1.42 (m, 1H), 1.24 (s, 9H), 1.22 – 1.10 (m, 4H), 0.79 (t, *J* = 6.8 Hz, 3H); ^{13}C NMR (101 MHz, $CDCl_3$) δ 211.32, 149.87, 140.04, 139.63, 130.75, 126.00, 125.18, 48.27, 41.30, 38.73, 34.48, 32.09, 31.40, 31.30, 29.73, 25.45, 22.81, 14.02; HR-MS (ESI) m/z calcd for $C_{22}H_{33}O$[M + H^+]: 313.2526, found: 313.2525.

(*E*)-3-(2-(4-fluorophenyl)hex-1-en-1-yl)cyclohexan-1-one (5e)

Following general procedure for Conditions B, the crude mixture was purified by flash chromatography on silica gel (eluted with ethyl acetate : hexanes = 1% ~ 2%) to give the product **5e** (32.9 mg, 60% yield) as a colorless oil. ^{1}H NMR (400 MHz, $CDCl_3$) δ 7.22 – 7.17 (m, 2H), 6.95 – 6.88 (m, 2H), 5.34 (d, *J* = 9.4 Hz, 1H), 2.84 – 2.71 (m, 1H), 2.40 – 2.30 (m, 4H), 2.29 –

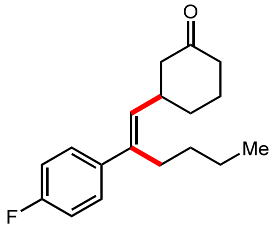

2.19 (m, 1H), 2.18 – 2.10 (m, 1H), 2.08 – 2.01 (m, 1H), 1.86 – 1.79 (m, 1H), 1.75 – 1.62 (m, 1H), 1.54 – 1.44 (m, 1H), 1.23 – 1.16 (m, 4H), 0.77 (t, *J* = 6.8 Hz, 3H); ^{13}C NMR (101 MHz, $CDCl_3$) δ 211.15, 162.02 (d, *J* = 246.5 Hz, 1C), 139.63, 138.79 (d, *J* = 3.26 Hz, 1C), 131.36 (d, *J* = 0.74 Hz, 1C), 128.05 (d, *J* = 7.75 Hz, 1C), 115.05 (d, *J* = 21.22 Hz, 1C), 48.17, 41.29, 38.68, 32.03, 31.03, 29.96, 25.41, 22.67, 14.00; ^{19}F NMR (376 MHz, $CDCl_3$) δ -116.01 – -116.23 (m, 1F); HR-MS (ESI) m/z calcd for $C_{18}H_{24}FO$ [M + H$^+$]: 275.1806, found: 275.1804.

(*E*)-3-(2-(4-chlorophenyl)hex-1-en-1-yl)cyclohexan-1-one (5f)

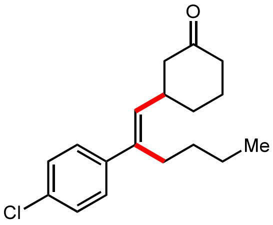

Following general procedure for Conditions C with slight modification: 2.0 equiv of alkyne was used and 1.0 equiv of alkyne was added after 3 h. The crude mixture was purified by flash chromatography on silica gel (eluted with ethyl acetate : hexanes = 1% ~ 2%) to give the product **5f** (32.5 mg, 56% yield) as a colorless oil. ^{1}H NMR (400 MHz, $CDCl_3$) δ 7.26 – 7.19 (m, 4H), 5.42 (d, *J* = 9.3 Hz, 1H), 2.88 – 2.78 (m, 1H), 2.46 – 2.36 (m, 4H), 2.33 – 2.25 (m, 1H), 2.22 – 2.16 (m, 1H), 2.13 – 2.06 (m, 1H), 1.90 – 1.83 (m, 1H), 1.79 – 1.68 (m, 1H), 1.58 – 1.49 (m, 1H), 1.26 – 1.22 (m, 4H), 0.83 (t, *J* = 6.8 Hz, 3H); ^{13}C NMR (101 MHz, $CDCl_3$) δ 211.03, 141.23, 139.56, 132.68, 131.83, 128.42, 127.87, 48.09, 41.29, 38.69, 31.98, 31.04, 29.76, 25.41, 22.67, 13.99; HR-MS (ESI) m/z calcd for $C_{18}H_{24}ClO$[M + H$^+$]: 291.1510, found: 291.1506.

(*E*)-3-(2-(4-bromophenyl)hex-1-en-1-yl)cyclohexan-1-one (5g)

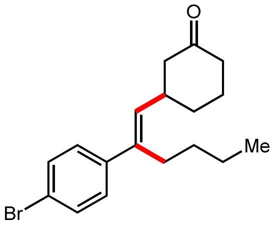

Following general procedure for Conditions B, the crude mixture was purified by flash chromatography on silica gel (eluted with ethyl acetate : hexanes = 1% ~ 2%) to give the product **5g** (33.5 mg, 50% yield) as a colorless oil. ^{1}H NMR (600 MHz, $CDCl_3$) δ 7.42 (d, *J* = 8.1 Hz, 2H), 7.18 (d, *J* = 8.1 Hz, 2H), 5.45 (d, *J* = 9.4 Hz, 1H), 2.90 – 2.79 (m, 1H), 2.48 – 2.37 (m, 4H), 2.35 – 2.27 (m, 1H), 2.23 – 2.19 (m, 1H), 2.15 – 2.06 (m, 1H), 1.90 – 1.88 (m, 1H), 1.81 – 1.71 (m, 1H), 1.64 – 1.51 (m, 1H), 1.30 – 1.23 (m, 4H), 0.85 (t, *J* = 6.2 Hz, 3H); ^{13}C NMR (151 MHz, $CDCl_3$) δ 210.91, 141.73, 139.62, 131.89, 131.37, 128.25, 120.80, 48.06, 41.29, 38.69, 31.96, 31.04, 29.71, 25.40, 22.66, 13.98; HR-MS (ESI) m/z calcd for $C_{18}H_{24}BrO$[M + H$^+$]: 335.1005, found: 335.1002.

(*E*)-3-(2-(*m*-tolyl)hex-1-en-1-yl)cyclohexan-1-one (5h)

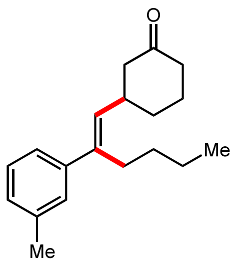

Following general procedure for Conditions B with slight modification: After 5 h, 1.0 equiv alkyne was added. The crude mixture was purified by flash chromatography on silica gel (eluted with ethyl acetate : hexanes = 1% ~ 2%) to give the product **5h** (33.5 mg, 62% yield) as a colorless oil. ^{1}H NMR (400 MHz, $CDCl_3$) δ 7.22 – 7.17 (m, 1H), 7.14 – 7.09 (m, 2H), 7.06 (dd, J = 7.1, 1.8 Hz, 1H), 5.45 (d, J = 9.4 Hz, 1H), 2.91 – 2.80 (m, 1H), 2.50 – 2.37 (m, 4H), 2.35 (s, 3H), 2.34 – 2.26 (m, 1H), 2.26 – 2.18 (m, 1H), 2.14 – 2.08 (m, 1H), 1.94 – 1.86 (m, 1H), 1.82 – 1.70 (m, 1H), 1.62 – 1.50 (m, 1H), 1.32 – 1.24 (m, 4H), 0.85 (t, J = 7.2 Hz, 3H); ^{13}C NMR (101 MHz, $CDCl_3$) δ 211.30, 142.83, 140.65, 137.80, 131.18, 128.17, 127.71, 127.29, 123.64, 48.23, 41.32, 38.71, 32.07, 31.19, 29.87, 25.45, 22.74, 21.58, 14.01; HR-MS (ESI) m/z calcd for $C_{19}H_{27}O$[M + H$^+$]: 271.2056, found: 271.2055.

(*E*)-3-(2-(3-methoxyphenyl)hex-1-en-1-yl)cyclohexan-1-one (5i)

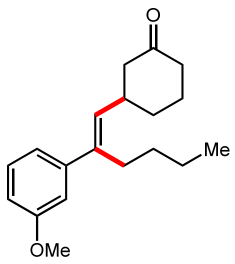

Following general procedure for Conditions B with slight modification: After 5 h, 1.0 equiv alkyne was added. The crude mixture was purified by flash chromatography on silica gel (eluted with ethyl acetate : hexanes = 1% ~ 2%) to give the product **5i** (35.5 mg, 62% yield) as a colorless oil. ^{1}H NMR (400 MHz, $CDCl_3$) δ 7.22 (t, J = 7.9 Hz, 1H), 6.94 – 6.89 (m, 1H), 6.87 – 6.84 (m, 1H), 6.82 – 6.77 (m, 1H), 5.47 (d, J = 9.4 Hz, 1H), 3.82 (s, 3H), 2.91 – 2.79 (m, 1H), 2.48 – 2.37 (m, 4H), 2.36 – 2.26 (m, 1H), 2.25 – 2.18 (m, 1H), 2.16 – 2.07 (m, 1H), 1.94 – 1.85 (m, 1H), 1.82 – 1.70 (m, 1H), 1.61 – 1.50 (m, 1H), 1.31 – 1.26 (m, 4H), 0.84(t, J = 7.2 Hz, 3H); ^{13}C NMR (101 MHz, $CDCl_3$) δ 211.23, 159.56, 144.43, 140.42, 131.46, 129.23, 119.13, 112.71, 111.91, 55.30, 48.17, 41.30, 38.69, 32.03, 31.17, 29.91, 25.44, 22.74, 14.01; HR-MS (ESI) m/z calcd for $C_{19}H_{27}O_2$[M + H$^+$]: 287.2006, found: 287.2004.

(*E*)-3-(2-(3-fluorophenyl)hex-1-en-1-yl)cyclohexan-1-one (5j)

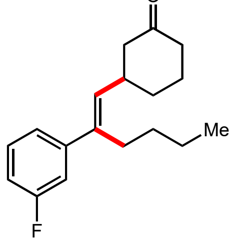

Following general procedure for Conditions C with slight modification: 2.0 equiv alkyne was used and after 3 h, 1.0 equiv alkyne was added. The crude mixture was purified by flash chromatography on silica gel (eluted with ethyl acetate : hexanes = 1% ~ 2%) to give the product **5j** (29.6 mg, 54% yield) as a colorless oil. ^{1}H NMR (600 MHz, $CDCl_3$) δ 7.22 – 7.15 (m, 1H), 7.02 (d, J = 7.8 Hz, 1H), 6.94 (d, J = 10.5 Hz, 1H), 6.88 – 6.83 (m, 1H), 5.42 (d, J = 9.4 Hz, 1H), 2.83 – 2.74 (m, 1H), 2.42 – 2.31 (m, 4H), 2.29 – 2.20 (m, 1H), 2.19 – 2.12 (m, 1H), 2.09 – 2.01 (m, 1H), 1.86 – 1.79 (m, 1H), 1.73 – 1.65 (m, 1H), 1.54 – 1.45 (m, 1H), 1.25 – 1.18 (m, 4H), 0.78 (t, J = 6.6 Hz, 3H); ^{13}C NMR (151 MHz, $CDCl_3$) δ 210.92, 162.93 (d, J = 245.01 Hz,

1C), 145.19 (d, J = 7.32 Hz, 1C), 139.59 (d, J = 2.14 Hz, 1C), 132.19, 129.67 (d, J = 8.27 Hz, 1C), 122.16 (d, J = 2.70 Hz, 1C), 113.70 (d, J = 21.2 Hz, 1C), 113.46 (d, J = 21.69 Hz, 1C), 48.04, 41.28, 38.67, 31.95, 31.10, 29.77, 25.41, 22.69, 13.98; ^{19}F NMR (565 MHz, $CDCl_3$) δ -113.73 – -113.77 (m, 1F); HR-MS (ESI) m/z calcd for $C_{18}H_{24}FO$ [M + H^+]: 275.1806, found: 275.1802.

(*E*)-3-(2-(2-fluorophenyl)hex-1-en-1-yl)cyclohexan-1-one (5k)

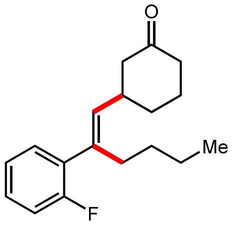

Following general procedure for Conditions B, the crude mixture was purified by flash chromatography on silica gel (eluted with ethyl acetate : hexanes = 1% ~ 2%) to give the product **5k** (35.7 mg, 65% yield) as a colorless oil. ^{1}H NMR (400 MHz, $CDCl_3$) δ 7.29 – 7.23 (m, 1H), 7.12 – 7.06 (m, 1H), 7.04 – 6.99 (m, 1H), 6.97 – 6.90 (m, 1H), 5.49 (d, J = 9.4 Hz, 1H), 2.92 – 2.79 (m, 1H), 2.49 – 2.38 (m, 4H), 2.36 – 2.27 (m, 1H), 2.26 – 2.18 (m, 1H), 2.17 – 2.08 (m, 1H), 1.94 – 1.86 (m, 1H), 1.83 – 1.70 (m, 1H), 1.63 – 1.51 (m, 1H), 1.31 – 1.24 (m, 4H), 0.85 (t, J = 6.8 Hz, 3H); ^{13}C NMR (101 MHz, $CDCl_3$) δ 210.97, 162.92 (d, J = 245.96 Hz, 1C), 145.18 (d, J = 7.28 Hz, 1C), 139.58 (d, J = 2.02 Hz, 1C), 132.19, 129.67 (d, J = 8.44 Hz, 1C), 122.17 (d, J = 2.77 Hz, 1C), 113.71 (d, J = 21.36 Hz, 1C), 113.45 (d, J = 21.65 Hz, 1C), 48.05, 41.29, 38.68, 31.95, 31.09, 29.77, 25.42, 22.69, 13.99; ^{19}F NMR (376 MHz, $CDCl_3$) δ -111.72 – -113.79 (m, 1F); HR-MS (ESI) m/z calcd for $C_{18}H_{24}FO$[M + H^+]: 275.1806, found: 275.1802.

***tert*-butyl (*E*)-5-(1-(3-oxocyclohexyl)hex-1-en-2-yl)-1*H*-indole-1-carboxylate (5l)**

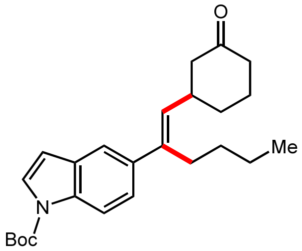

Following general procedure for Conditions B with slight modification: After 3 h, 1.0 equiv alkyne was added. The crude mixture was purified by flash chromatography on silica gel (eluted with ethyl acetate : hexanes = 1 : 40) to give the product **5l** (49.8 mg, 63% yield) as a colorless oil. ^{1}H NMR (400 MHz, $CDCl_3$) δ 8.05 (d, J = 8.6 Hz, 1H), 7.58 (d, J = 3.6 Hz, 1H), 7.49 (d, J = 1.8 Hz, 1H), 7.28 (dd, J = 8.6, 1.8 Hz, 1H), 6.54 (d, J = 3.6 Hz, 1H), 5.48 (d, J = 9.3 Hz, 1H), 2.96 – 2.82 (m, 1H), 2.56 – 2.49 (m, 2H), 2.49 – 2.37 (m, 2H), 2.36 – 2.30 (m, 1H), 2.28 – 2.20 (m, 1H), 2.17 – 2.08 (m, 1H), 1.97 – 1.88 (m, 1H), 1.82 – 1.75 (m, 1H), 1.67 (s, 9H), 1.61 – 1.53 (m, 1H), 1.32 – 1.26 (m, 4H), 0.83(t, J = 7.2 Hz, 3H); ^{13}C NMR (101 MHz, $CDCl_3$) δ 211.40, 149.82, 140.84, 137.63, 134.34, 130.99, 130.69, 126.31, 123.26, 118.82, 114.79, 107.47, 83.71, 48.34, 41.34, 38.79, 32.15, 31.14, 30.23, 28.27, 25.47, 22.72, 14.03; HR-MS (ESI) m/z calcd for $C_{25}H_{34}NO_3$[M + H^+]: 396.2533, found: 396.2528.

(*E*)-3-(3-phenylhept-2-en-2-yl)cyclohexan-1-one (6a)

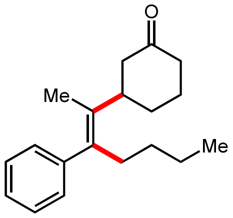

Following general procedure for Conditions A, the crude mixture was purified by flash chromatography on silica gel (eluted with ethyl acetate : hexanes = 1% ~ 2%) to give the product **6a** (36.2 mg, 67% yield) as a colorless oil. ^{1}H NMR (400 MHz, $CDCl_3$) δ 7.33 – 7.27 (m, 2H), 7.24 – 7.18 (m, 1H), 7.07 – 7.03 (m, 2H), 3.10 – 3.00 (m, 1H), 2.50 – 2.39 (m, 2H), 2.36 – 2.25 (m, 4H), 2.19 – 2.11 (m, 1H), 1.80 – 1.72 (m, 2H), 1.68 – 1.61 (m, 1H), 1.45 (s, 3H), 1.27 – 1.16 (m, 4H), 0.81 (t, J = 6.9 Hz, 3H); ^{13}C NMR (101 MHz, $CDCl_3$) δ 212.05, 131.98, 128.88, 128.03, 126.05, 46.44, 41.56, 41.36, 33.62, 30.84, 29.91, 25.86, 22.65, 14.78, 14.04; HR-MS (ESI) m/z calcd for $C_{19}H_{27}O[M + H^+]$: 271.2056, found: 271.2055.

(*E*)-3-(3-(*p*-tolyl)hept-2-en-2-yl)cyclohexan-1-one (6b)

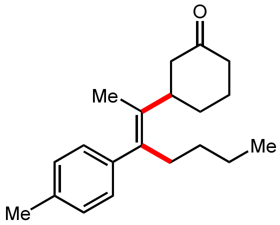

Following general procedure for Conditions A, the crude mixture was purified by flash chromatography on silica gel (eluted with ethyl acetate : hexanes = 1% ~ 2%) to give the product **6b** (39.8 mg, 70% yield) as a colorless oil. ^{1}H NMR (600 MHz, $CDCl_3$) δ 7.13 – 7.10 (m, 2H), 6.96 – 6.92 (m, 2H), 3.08 – 3.01 (m, 1H), 2.48 – 2.40 (m, 2H), 2.34 (s, 3H), 2.33 – 2.23 (m, 4H), 2.18 – 2.11 (m, 1H), 1.79 – 1.72 (m, 3H), 1.45 (s, 3H), 1.25 – 1.15 (m, 4H), 0.81 (t, J = 7.2 Hz, 3H); ^{13}C NMR (151 MHz, $CDCl_3$) δ 212.04, 141.04, 137.12, 135.52, 131.81, 128.76, 128.72, 46.45, 41.61, 41.36, 33.68, 30.89, 29.93, 25.87, 22.67, 21.19, 14.77, 14.04; HR-MS (ESI) m/z calcd for $C_{20}H_{29}O[M + H^+]$: 285.2213, found: 285.2211.

(*E*)-3-(3-(4-methoxyphenyl)hept-2-en-2-yl)cyclohexan-1-one (6c)

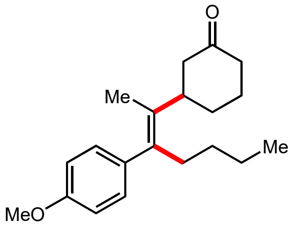

Following general procedure for Conditions A, the crude mixture was purified by flash chromatography on silica gel (eluted with ethyl acetate : hexanes = 1% ~ 2%) to give the product **6c** (36.6 mg, 61% yield) as a colorless oil. ^{1}H NMR (600 MHz, $CDCl_3$) δ 6.99 – 6.95 (m, 2H), 6.86 – 6.82 (m, 2H), 3.81 (s, 3H), 3.08 – 3.00 (m, 1H), 2.47 – 2.40 (m, 2H), 2.34 – 2.23 (m, 4H), 2.18 – 2.11 (m, 1H), 1.79 – 1.70 (m, 3H), 1.46 (s, 3H), 1.26 – 1.14 (m, 4H), 0.81 (t, J = 7.2 Hz, 3H); ^{13}C NMR (151 MHz, $CDCl_3$) δ 212.04, 157.86, 136.75, 136.32, 131.96, 129.92, 113.40, 55.25, 46.46, 41.65, 41.36, 33.72, 30.90, 29.94, 25.87, 22.65, 14.79, 14.05; HR-MS (ESI) m/z calcd for $C_{20}H_{29}O_2[M + H^+]$: 301.2162, found: 301.2160.

(*E*)-3-(3-(4-chlorophenyl)hept-2-en-2-yl)cyclohexan-1-one (6d)

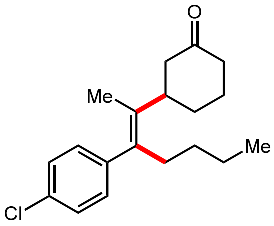

Following general procedure for Conditions A, the crude mixture was purified by flash chromatography on silica gel (eluted with ethyl acetate : hexanes = 1% ~ 2%) to give the product **6d** (30.5 mg, 50% yield) as a colorless oil. ^{1}H NMR (600 MHz, $CDCl_3$) δ 7.30 – 7.25 (m, 2H), 7.02 – 6.95 (m, 2H), 3.07 – 3.00 (m, 1H), 2.47 – 2.40 (m, 2H), 2.34 – 2.22 (m, 4H), 2.20 – 2.10 (m, 1H), 1.80 – 1.70 (m, 3H), 1.44 (s, 3H), 1.26 – 1.13 (m, 4H), 0.81 (t, J = 7.2 Hz, 3H); ^{13}C NMR (151 MHz, $CDCl_3$) δ 211.74, 142.42, 136.17, 132.77, 131.89, 130.31, 128.29, 46.34, 41.56, 41.33, 33.51, 30.78, 29.87, 25.82, 22.61, 14.82, 14.01; HR-MS (ESI) m/z calcd for $C_{19}H_{26}ClO$[M + H$^+$]: 305.1667, found: 305.1664.

(*E*)-3-(4-phenyloct-3-en-3-yl)cyclohexan-1-one (6e)

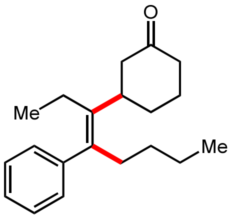

Following general procedure for Conditions A, the crude mixture was purified by flash chromatography on silica gel (eluted with ethyl acetate : hexanes = 1% ~ 2%) to give the product **6e** (39.8 mg, 70% yield) as a colorless oil. ^{1}H NMR of the crude mixture revealed a regioselectivity ratio as 5 : 1. ^{1}H NMR (600 MHz, $CDCl_3$) δ 7.36 – 7.27 (m, 2H), 7.25 – 7.18 (m, 1H), 7.06 (d, J = 7.5 Hz, 2H), 3.10 – 2.98 (m, 1H), 2.53 – 2.40 (m, 2H), 2.39 – 2.21 (m, 4H), 2.19 – 2.11 (m, 1H), 1.94 – 1.80 (m, 3H), 1.80 – 1.66 (m, 2H), 1.28 – 1.17 (m, 4H), 0.82 (t, J = 7.2 Hz, 3H), 0.77(t, J = 7.8 Hz, 3H); ^{13}C NMR (151 MHz, $CDCl_3$) δ 211.84, 143.96, 138.29, 138.19, 128.82, 128.00, 126.00, 47.27, 41.88, 41.41, 34.13, 30.69, 30.64, 26.02, 22.75, 21.93, 15.42, 14.06; HR-MS (ESI) m/z calcd for $C_{20}H_{29}O$ [M + H$^+$]: 285.2213, found: 285.2210.

(*Z*)-3-(3-methylhept-2-en-2-yl)cyclohexan-1-one (6f)

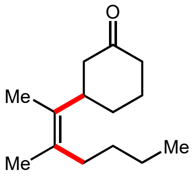

Following general procedure for Conditions A, the crude mixture was purified by flash chromatography on silica gel (eluted with ethyl acetate : hexanes = 1% ~ 2%) to give the product **6f** (30.0 mg, 72% yield) as a colorless oil. ^{1}H NMR (600 MHz, $CDCl_3$) δ 2.95 – 2.88 (m, 1H), 2.40 – 2.33 (m, 2H), 2.29 – 2.21 (m, 1H), 2.15 – 2.05 (m, 2H), 2.04 – 1.93 (m, 2H), 1.69 – 1.64 (m, 2H), 1.62 (s, 3H), 1.61 – 1.58 (m, 1H), 1.57 (s, 3H), 1.31 – 1.22 (m, 4H), 0.88 (t, J = 7.2 Hz, 3H); ^{13}C NMR (151 MHz, $CDCl_3$) δ 212.37, 129.87, 129.03, 46.49, 41.57, 41.36, 33.75, 31.13, 29.84, 25.79, 22.82, 19.20, 14.13, 13.04; HR-MS (ESI) m/z calcd for $C_{14}H_{25}O$[M + H$^+$]: 209.1900, found: 209.1899.

(*Z*)-3-(5-propylnon-4-en-4-yl)cyclohexan-1-one (6g)

Following general procedure for Conditions A, the crude mixture was purified by flash chromatography on silica gel (eluted with ethyl acetate : hexanes = 1% ~ 2%) to give the product

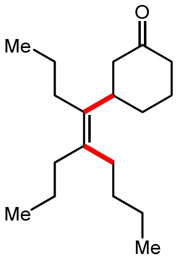

6g (26.4 mg, 50% yield) as a colorless oil. ^{1}H NMR (600 MHz, $CDCl_3$) δ 2.89 – 2.80 (m, 1H), 2.40 – 2.32 (m, 2H), 2.30 – 2.23 (m, 1H), 2.19 – 2.13 (m, 1H), 2.11 – 2.05 (m, 1H), 1.99 – 1.87 (m, 6H), 1.67 – 1.60 (m, 3H), 1.40 – 1.30 (m, 4H), 1.29 – 1.22 (m, 4H), 0.94 – 0.85 (m, 9H); ^{13}C NMR (151 MHz, $CDCl_3$) δ 212.29, 135.59, 134.67, 47.20, 41.99, 41.35, 34.50, 31.97, 30.77, 30.49, 30.32, 25.95, 24.48, 23.03, 22.05, 14.90, 14.40, 14.12; HR-MS (ESI) m/z calcd for $C_{18}H_{33}O[M + H^+]$: 265.2526, found: 265.2524.

(*E*)-3-(3-butylnon-2-en-2-yl)cyclohexan-1-one (6h)

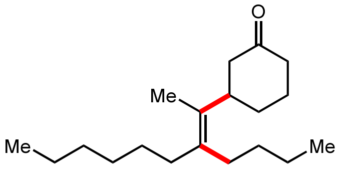

Following general procedure for Conditions A, the crude mixture was purified by flash chromatography on silica gel (eluted with ethyl acetate : hexanes = 1% ~ 2%) to give the product **6h** (40.6 mg, 73% yield) as a colorless oil. ^{1}H NMR (600 MHz, $CDCl_3$) δ 2.90 – 2.83 (m, 1H), 2.41 – 2.35 (m, 2H), 2.29 – 2.22 (m, 1H), 2.16 – 2.06 (m, 2H), 2.00 – 1.90 (m, 4H), 1.71 – 1.61 (m, 3H), 1.57 (s, 3H), 1.32 – 1.22 (m, 12H), 0.88 (t, *J* = 7.2 Hz, 6H); ^{13}C NMR (151 MHz, $CDCl_3$) δ 212.42, 134.98, 129.26, 46.58, 41.74, 41.37, 33.09, 31.92, 31.83, 31.79, 29.94, 29.63, 28.65, 25.86, 23.05, 22.76, 14.17, 14.12, 12.55; HR-MS (ESI) m/z calcd for $C_{19}H_{35}O[M + H^+]$: 279.2682, found:279.2680.

(*E*)-3-(3-butyl-6-methylhept-2-en-2-yl)cyclohexan-1-one (6i)

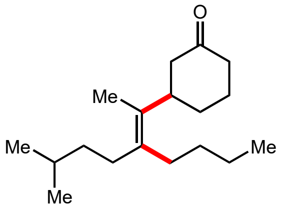

Following general procedure for Conditions A, the crude mixture was purified by flash chromatography on silica gel (eluted with ethyl acetate : hexanes = 1% ~ 2%) to give the product **6i** (33.3 mg, 63% yield) as a colorless oil. ^{1}H NMR (600 MHz, $CDCl_3$) δ 2.90 – 2.83 (m, 1H), 2.41 – 2.34 (m, 2H), 2.29 – 2.22 (m, 1H), 2.16 – 2.05 (m, 2H), 2.00 – 1.90 (m, 4H), 1.71 – 1.64 (m, 2H), 1.64 – 1.61 (m, 1H), 1.57 (s, 3H), 1.54 – 1.47 (m, 1H), 1.31 – 1.23 (m, 4H), 1.21 – 1.15 (m, 2H), 0.92 – 0.87 (m, 9H); ^{13}C NMR (151 MHz, $CDCl_3$) δ 212.39, 135.14, 129.05, 46.56, 41.71, 41.37, 37.84, 31.82, 31.01, 29.93, 28.58, 25.85, 23.03, 22.67, 14.12, 12.43; HR-MS (ESI) m/z calcd for $C_{18}H_{33}O[M + H^+]$: 265.2526, found: 265.2525.

(*E*)-3-(2-phenylbut-1-en-1-yl)cyclohexan-1-one (7a)

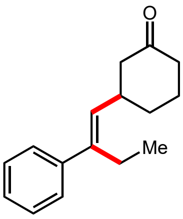

Following general procedure for Conditions B, the crude mixture was purified by flash chromatography on silica gel (eluted with ethyl acetate : hexanes = 1% ~ 2%) to give the product **7a** (27.4 mg, 60% yield) as a colorless oil. ^{1}H NMR (400 MHz, $CDCl_3$) δ 7.28 – 7.21 (m, 4H), 7.19 – 7.14 (m, 1H), 5.39 (d, *J* = 9.3

Hz, 1H), 2.86 – 2.74 (m, 1H), 2.42 (q, J = 7.5 Hz, 2H), 2.39 – 2.30 (m, 2H), 2.28 – 2.20 (m, 1H), 2.20 – 2.11 (m, 1H), 2.08 – 2.00 (m, 1H), 1.87 – 1.80 (m, 1H), 1.75 – 1.64 (m, 1H), 1.55 – 1.45 (m, 1H), 0.89 (t, J = 7.5 Hz, 3H); ^{13}C NMR (101 MHz, $CDCl_3$) δ 211.22, 142.42, 141.95, 130.71, 128.29, 126.96, 126.49, 48.23, 41.29, 38.64, 32.07, 25.44, 23.23, 14.04; HR-MS (ESI) m/z calcd for $C_{16}H_{21}O[M + H^+]$: 229.1587, found: 229.1585.

(*E*)-3-(2-phenylnon-1-en-1-yl)cyclohexan-1-one (7b)

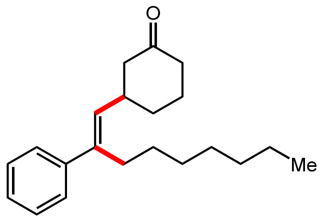

Following general procedure for Conditions B with slight modification: After 5 h, 1.0 equiv alkyne was added. The crude mixture was purified by flash chromatography on silica gel (eluted with ethyl acetate : hexanes = 1% ~ 2%) to give the product **7b** (34.0 mg, 57% yield) as a colorless oil. ^{1}H NMR (400 MHz, $CDCl_3$) δ 7.26 – 7.20 (m, 4H), 7.20 – 7.14 (m, 1H), 5.39 (d, J = 9.4 Hz, 1H), 2.84 – 2.74 (m, 1H), 2.42 – 2.30 (m, 4H), 2.29 – 2.19 (m, 1H), 2.18 – 2.11 (m, 1H), 2.08 – 2.01 (m, 1H), 1.86 – 1.79 (m, 1H), 1.75 – 1.63 (m, 1H), 1.55 – 1.44 (m, 1H), 1.25 – 1.13 (m, 10H), 0.78 (t, J = 6.4 Hz, 3H); ^{13}C NMR (101 MHz, $CDCl_3$) δ 211.24, 142.80, 140.60, 131.34, 128.28, 126.93, 126.54, 48.21, 41.31, 38.71, 32.06, 31.85, 30.07, 29.60, 29.15, 28.97, 25.43, 22.69, 14.15; HR-MS (ESI) m/z calcd for $C_{21}H_{31}O[M + H^+]$: 299.2369, found: 299.2369.

(*E*)-3-(5-methyl-2-phenylhex-1-en-1-yl)cyclohexan-1-one (7c)

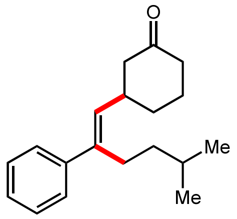

Following general procedure for Conditions B with slight modification: After 3 h, 1.0 equiv alkyne was added. The crude mixture was purified by flash chromatography on silica gel (eluted with ethyl acetate : hexanes = 1% ~ 2%) to give the product **7c** (32.9 mg, 61% yield) as a colorless oil. ^{1}H NMR (400 MHz, $CDCl_3$) δ 7.26 – 7.21 (m, 4H), 7.19 – 7.14 (m, 1H), 5.39 (d, J = 9.4 Hz, 1H), 2.85 – 2.72 (m, 1H), 2.42 – 2.30 (m, 4H), 2.29 – 2.19 (m, 1H), 2.19 – 2.11 (m, 1H), 2.08 – 2.01 (m, 1H), 1.87 – 1.80 (m, 1H), 1.75 – 1.63 (m, 1H), 1.55 – 1.42 (m, 2H), 1.17 – 1.09 (m, 2H), 0.79 (d, J = 6.8 Hz, 6H); ^{13}C NMR (101 MHz, $CDCl_3$) δ 211.20, 142.80, 140.79, 131.12, 128.30, 126.94, 126.48, 48.21, 41.30, 38.73, 38.35, 32.07, 28.25, 28.08, 25.43, 22.52; HR-MS (ESI) m/z calcd for $C_{19}H_{27}O[M + H^+]$: 271.2056, found: 271.2054.

(*E*)-3-(2-ethylhex-1-en-1-yl)cyclohexan-1-one (7d)

Following general procedure for Conditions A, the crude mixture was purified by flash chromatography on silica gel (eluted with ethyl acetate : hexanes = 1% ~ 2%) to give the product **7d** (22.5 mg, 54% yield) as a colorless oil. ^{1}H NMR (400 MHz, $CDCl_3$) δ 4.88 (d, J = 9.2 Hz,

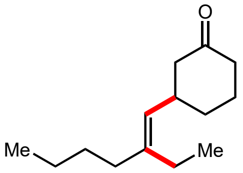

1H), 2.71 – 2.53 (m, 1H), 2.34 – 2.14 (m, 3H), 2.10 – 1.97 (m, 2H), 1.97 – 1.85 (m, 4H), 1.77 – 1.68 (m, 1H), 1.66 – 1.59 (m, 1H), 1.45 – 1.33 (m, 1H), 1.31 – 1.17 (m, 4H), 0.88 (t, J = 7.6 Hz, 3H), 0.83 (t, J = 7.2 Hz, 3H); ^{13}C NMR (101 MHz, $CDCl_3$) δ 211.78, 141.61, 127.17, 48.67, 41.32, 37.97, 36.02, 32.34, 30.34, 25.47, 23.29, 22.49, 14.09, 13.68; HR-MS (ESI) m/z calcd for $C_{14}H_{25}O$[M + H$^+$]: 209.1900, found: 209.1898.

(*E*)-3-(6-methoxy-2-phenylhex-1-en-1-yl)cyclohexan-1-one (7e)

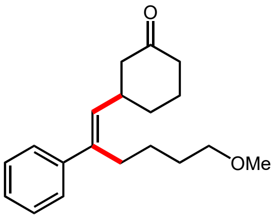

Following general procedure for Conditions B, the crude mixture was purified by flash chromatography on silica gel (eluted with ethyl acetate : hexanes = 1% ~ 2%) to give the product **7e** (36.1 mg, 63% yield) as a colorless oil. ^{1}H NMR (400 MHz, $CDCl_3$) δ 7.25 – 7.23 (m, 3H), 7.19 – 7.14 (m, 2H), 5.41 (d, J = 9.4 Hz, 1H), 3.27 – 3.22 (m, 2H), 3.21 (s, 3H), 2.85 – 2.73 (m, 1H), 2.47 – 2.40 (m, 2H), 2.39 – 2.30 (m, 2H), 2.29 – 2.20 (m, 1H), 2.19 – 2.11 (m, 1H), 2.09 – 2.00 (m, 1H), 1.88 – 1.80 (m, 1H), 1.76 – 1.63 (m, 1H), 1.50 – 1.42 (m, 3H), 1.35 – 1.26 (m, 2H); ^{13}C NMR (101 MHz, $CDCl_3$) δ 211.08, 142.58, 140.22, 131.68, 128.32, 127.00, 126.54, 72.60, 58.59, 48.16, 41.29, 38.69, 32.03, 29.77, 29.49, 25.48, 25.42; HR-MS (ESI) m/z calcd for $C_{19}H_{26}NaO_2$[M + Na$^+$]: 309.1825, found: 309.1824.

(*E*)-3-(6-phenoxy-2-phenylhex-1-en-1-yl)cyclohexan-1-one (7f)

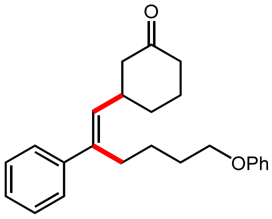

Following general procedure for Conditions B with slight modification: After 5 h, 1.0 equiv alkyne was added. The crude mixture was purified by flash chromatography on silica gel (eluted with ethyl acetate : hexanes = 2%) to give the product **7f** (41.8 mg, 60% yield) as a colorless oil. ^{1}H NMR (600 MHz, $CDCl_3$) δ 7.33 – 7.28 (m, 4H), 7.26 – 7.21 (m, 3H), 6.91 (t, J = 7.3 Hz, 1H), 6.86 – 6.81 (m, 2H), 5.49 (d, J = 9.4 Hz, 1H), 3.91 – 3.86 (m, 2H), 2.91 – 2.82 (m, 1H), 2.55 (t, J = 7.8 Hz, 2H), 2.46 – 2.36 (m, 2H), 2.34 – 2.26 (m, 1H), 2.25 – 2.19 (m, 1H), 2.12 – 2.06 (m, 1H), 1.92 – 1.86 (m, 1H), 1.77 – 1.68 (m, 3H), 1.59 – 1.53 (m, 1H), 1.52 – 1.46 (m, 2H); ^{13}C NMR (101 MHz, $CDCl_3$) δ 211.11, 159.00, 142.50, 140.09, 131.82, 129.46, 128.36, 127.06, 126.55, 120.59, 114.50, 67.44, 48.16, 41.27, 38.67, 32.01, 29.76, 29.62, 29.03, 25.35; HR-MS (ESI) m/z calcd for $C_{24}H_{28}NaO_2$[M + Na$^+$]: 371.1982, found: 371.1980.

(*E*)-3-(2,5-diphenylpent-1-en-1-yl)cyclohexan-1-one (7g)

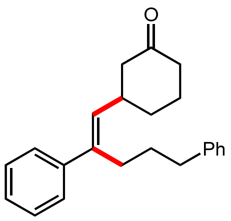

Following general procedure for Conditions B, the crude mixture was purified by flash chromatography on silica gel (eluted with ethyl acetate : hexanes = 1% ~2%) to give the product **7g** (40.1 mg, 63% yield) as a colorless oil. ^{1}H NMR (400 MHz, $CDCl_3$) δ 7.23 – 7.20 (m, 4H), 7.19 – 7.14 (m, 3H), 7.11 – 7.06 (m, 1H), 7.05 – 7.00 (m, 2H), 5.40 (d, *J* = 9.3 Hz, 1H), 2.71 – 2.60 (m, 1H), 2.50 (t, *J* = 7.6 Hz, 2H), 2.43 (t, *J* = 7.8 Hz, 2H), 2.36 – 2.27 (m, 2H), 2.26 – 2.18 (m, 1H), 2.16 – 2.08 (m, 1H), 2.04 – 1.97 (m, 1H), 1.80 – 1.74 (m, 1H), 1.64 – 1.57 (m, 3H), 1.51 – 1.40 (m, 1H); ^{13}C NMR (101 MHz, $CDCl_3$) δ 211.10, 142.49, 142.01, 140.16, 131.74, 128.44, 128.35, 128.34, 127.03, 126.53, 125.86, 48.12, 41.26, 38.59, 35.57, 31.98, 30.43, 29.36, 25.36; HR-MS (ESI) m/z calcd for $C_{23}H_{27}O$[M + H$^+$]: 319.2056, found: 319.2054.

(*E*)-3-(4-(2,3-dihydrobenzofuran-5-yl)-2-phenylbut-1-en-1-yl)cyclohexan-1-one (7h)

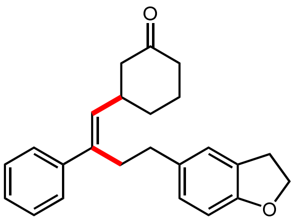

Following general procedure for Conditions B with slight modification: After 5 h, 1.0 equiv alkyne was added. The crude mixture was purified by flash chromatography on silica gel (eluted with ethyl acetate : hexanes = 1 : 40) to give the product **7h** (34.6 mg, 50% yield) as a colorless oil. ^{1}H NMR (400 MHz, $CDCl_3$) δ 7.40 – 7.31 (m, 4H), 7.31 – 7.26 (m, 1H), 6.95 (d, *J* = 1.8 Hz, 1H), 6.82 – 6.76 (m, 1H), 6.67 (d, *J* = 8.1 Hz, 1H), 5.46 (d, *J* = 9.5 Hz, 1H), 4.60 – 4.49 (m, 2H), 3.25 – 3.09 (m, 2H), 2.80 – 2.70 (m, 2H), 2.70 – 2.58 (m, 1H), 2.58 – 2.47 (m, 2H), 2.40 – 2.32 (m, 1H), 2.30 – 2.21 (m, 1H), 2.20 – 2.14 (m, 1H), 2.13 – 1.99 (m, 2H), 1.67 – 1.58 (m, 2H), 1.49 – 1.36 (m, 1H); ^{13}C NMR (101 MHz, $CDCl_3$) δ 211.03, 158.42, 142.38, 139.02, 133.63, 132.31, 128.44, 128.04, 127.13, 127.11, 126.71, 124.94, 108.88, 71.27, 47.91, 41.28, 38.87, 34.16, 32.47, 31.81, 29.87, 25.42; HR-MS (ESI) m/z calcd for $C_{24}H_{27}O_2$[M + H$^+$]: 347.2006, found: 347.2004.

(*E*)-3-(6-chloro-2-phenylhex-1-en-1-yl)cyclohexan-1-one (7i)

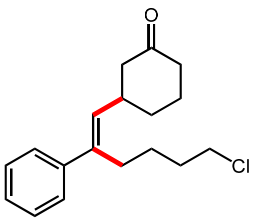

Following general procedure for Conditions B with slight modification: After 3 h, 1.0 equiv alkyne was added. The crude mixture was purified by flash chromatography on silica gel (eluted with ethyl acetate : hexanes = 1% ~2%) to give the product **7i** (37.2 mg, 64% yield) as a colorless oil. ^{1}H NMR (400 MHz, $CDCl_3$) δ 7.33 – 7.28 (m, 4H), 7.26 – 7.22 (m, 1H), 5.49 (d, *J* = 9.4 Hz, 1H), 3.50 – 3.42 (m, 2H), 2.91 – 2.78 (m, 1H), 2.50 (t, *J* = 7.7 Hz, 2H), 2.45 – 2.37 (m, 2H), 2.36 – 2.29 (m, 1H), 2.28 – 2.18 (m, 1H), 2.16 – 2.07 (m, 1H), 1.94 – 1.85 (m, 1H), 1.82 – 1.69 (m, 3H), 1.64 – 1.52 (m, 1H), 1.50 – 1.41 (m, 2H); ^{13}C NMR (101 MHz, $CDCl_3$) δ 210.99, 142.34, 139.76, 131.97, 128.39, 127.13, 126.51, 48.12, 44.74, 41.25, 38.70, 32.29,

32.01, 29.17, 26.07, 25.37; HR-MS (ESI) m/z calcd for $C_{18}H_{23}{}^{35}ClONa[M + Na^+]$: 313.1330, found: 313.1329.

(*E*)-3-(3-phenylundec-2-en-2-yl)cyclohexan-1-one (7j)

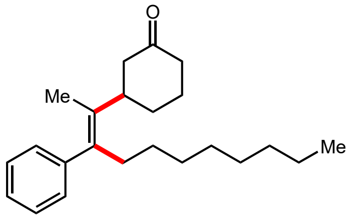

Following general procedure for Conditions A, the crude mixture was purified by flash chromatography on silica gel (eluted with ethyl acetate : hexanes = 1% ~2%) to give the product **7j** (41.1 mg, 63% yield) as a colorless oil. ^1H NMR (400 MHz, $CDCl_3$) δ 7.34 – 7.27 (m, 2H), 7.24 – 7.18 (m, 1H), 7.10 – 7.02 (m, 2H), 3.13 – 3.00 (m, 1H), 2.50 – 2.39 (m, 2H), 2.37 – 2.22 (m, 4H), 2.21 – 2.10 (m, 1H), 1.83 – 1.70 (m, 3H), 1.45 (s, 3H), 1.30 – 1.15 (m, 12H), 0.86 (t, J = 6.8 Hz, 3H); ^{13}C NMR (101 MHz, $CDCl_3$) δ 211.97, 144.08, 137.31, 131.98, 128.89, 128.03, 126.05, 46.43, 41.57, 41.36, 33.84, 31.91, 29.94, 29.57, 29.49, 29.32, 28.61, 25.86, 22.71, 14.78, 14.17; HR-MS (ESI) m/z calcd for $C_{23}H_{35}O[M + H^+]$: 327.2682, found: 327.2680.

(*E*)-3-(3-phenylpent-2-en-2-yl)cyclohexan-1-one (7k)

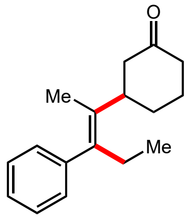

Following general procedure for Conditions A, the crude mixture was purified by flash chromatography on silica gel (eluted with ethyl acetate : hexanes = 1% ~2%) to give the product **7k** (28.1 mg, 58% yield) as a colorless oil. ^1H NMR (600 MHz, $CDCl_3$) δ 7.34 – 7.27 (m, 2H), 7.24 – 7.20 (m, 1H), 7.08 – 7.03 (m, 2H), 3.10 – 3.02 (m, 1H), 2.49 – 2.40 (m, 2H), 2.37 – 2.26 (m, 4H), 2.19 – 2.12 (m, 1H), 1.81 – 1.73 (m, 3H), 1.45 (s, 3H), 0.86 (t, J = 7.2 Hz, 3H); ^{13}C NMR (151 MHz, $CDCl_3$) δ 211.97, 143.84, 138.69, 131.58, 128.92, 128.05, 126.09, 46.55, 41.45, 41.35, 29.99, 26.93, 25.86, 14.72, 13.52; HR-MS (ESI) m/z calcd for $C_{17}H_{23}O[M + H^+]$: 243.1743, found: 243.1740.

(*E*)-3-(7-methoxy-3-phenylhept-2-en-2-yl)cyclohexan-1-one (7l)

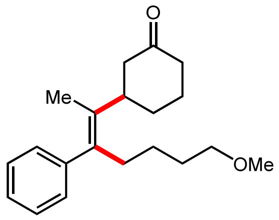

Following general procedure for Conditions A, the crude mixture was purified by flash chromatography on silica gel (eluted with ethyl acetate : hexanes = 1 : 40) to give the product **7l** (40.8 mg, 68% yield) as a colorless oil. ^1H NMR (400 MHz, $CDCl_3$) δ 7.35 – 7.26 (m, 2H), 7.21 (t, J = 7.4 Hz, 1H), 7.05 (d, J = 7.4 Hz, 2H), 3.42 – 3.29 (m, 2H), 3.27 (s, 3H), 3.12 – 3.00 (m, 1H), 2.51 – 2.25 (m, 6H), 2.19 – 2.10 (m, 1H), 1.82 – 1.69 (m, 3H), 1.58 – 1.47 (m, 2H), 1.46 (s, 3H), 1.32 – 1.22 (m, 2H); ^{13}C NMR (101 MHz, $CDCl_3$) δ 211.85, 143.75, 136.81, 132.37, 128.84, 128.04, 126.09, 72.65, 58.55, 46.35, 41.53, 41.29, 33.48, 29.85,

29.39, 25.80, 25.09, 14.78; HR-MS (ESI) m/z calcd for $C_{20}H_{29}O_2$[M + H$^+$]: 301.2162, found: 301.2160.

(*E*)-3-(2-phenylhepta-1,6-dien-1-yl)cyclohexan-1-one (7m)

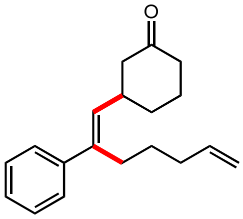

Following general procedure for Conditions B, the crude mixture was purified by flash chromatography on silica gel (eluted with ethyl acetate : hexanes = 1% ~2%) to give the product **7m** (36.5 mg, 68% yield) as a colorless oil. ^{1}H NMR (400 MHz, $CDCl_3$) δ 7.35 – 7.27 (m, 4H), 7.27 – 7.21 (m, 1H), 5.81 – 5.67 (m, 1H), 5.48 (d, *J* = 9.5 Hz, 1H), 5.01 – 4.89 (m, 2H), 2.91 – 2.80 (m, 1H), 2.48 (t, *J* = 7.9 Hz, 2H), 2.45 – 2.36 (m, 2H), 2.36 – 2.27 (m, 1H), 2.22 (t, *J* = 12.9 Hz, 1H), 2.15 – 2.07 (m, 1H), 2.06 – 1.98 (m, 2H), 1.94 – 1.85 (m, 1H), 1.82 – 1.69 (m, 1H), 1.62 – 1.51 (m, 1H), 1.46 – 1.36 (m, 2H); ^{13}C NMR (101 MHz, $CDCl_3$) δ 211.17, 142.59, 140.21, 138.43, 131.68, 128.32, 127.01, 126.54, 114.91, 48.17, 41.29, 38.67, 33.54, 32.03, 29.40, 28.05, 25.41; HR-MS (ESI) m/z calcd for $C_{19}H_{24}ONa$[M + Na$^+$]: 291.1719, found: 291.1719.

(*E*)-3-(2,7-diphenylhept-1-en-6-yn-1-yl)cyclohexan-1-one (7n)

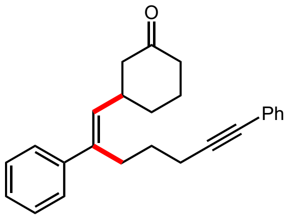

Following general procedure for Conditions B with slight modification: After 3 h, 1.0 equiv of alkyne was added. The crude mixture was purified by flash chromatography on silica gel (eluted with ethyl acetate : hexanes = 1% ~2%) to give the product **7n** (44.5 mg, 65% yield) as a colorless oil. ^{1}H NMR (400 MHz, $CDCl_3$) δ 7.35 – 7.27 (m, 5H), 7.25 – 7.17 (m, 5H), 5.49 (d, *J* = 9.4 Hz, 1H), 2.99 – 2.86 (m, 1H), 2.71 – 2.57 (m, 2H), 2.43 – 2.35 (m, 1H), 2.31 (t, *J* = 6.9 Hz, 3H), 2.27 – 2.20 (m, 1H), 2.19 – 2.12 (m, 1H), 2.05 – 1.96 (m, 1H), 1.91 – 1.82 (m, 1H), 1.64 – 1.52 (m, 4H); ^{13}C NMR (101 MHz, $CDCl_3$) δ 210.92, 142.20, 139.32, 132.38, 131.52, 128.36, 128.26, 127.64, 127.09, 126.51, 123.81, 89.74, 81.19, 48.08, 41.19, 38.55, 31.98, 28.71, 27.61, 25.22, 18.95; HR-MS (ESI) m/z calcd for $C_{25}H_{27}O$[M + H$^+$]: 343.2056, found: 343.2055.

ethyl (*E*)-6-(3-oxocyclohexyl)-5-phenylhex-5-enoate (7o)

Following general procedure for Conditions B with slight modification: After 5 h, 1.0 equiv alkyne was added. The crude mixture was purified by flash chromatography on silica gel (eluted with ethyl acetate : hexanes = 1 : 40) to give the product **7o** (38.3 mg, 61% yield) as a colorless oil. ^{1}H NMR (400 MHz, $CDCl_3$) δ 7.33 – 7.28 (m, 4H), 7.26 – 7.22 (m, 1H), 5.52 (d, *J* = 9.4 Hz, 1H), 4.10 (q, *J* = 7.2 Hz, 2H), 2.90 – 2.80 (m, 1H), 2.57 – 2.50 (m, 2H), 2.45 – 2.37 (m,

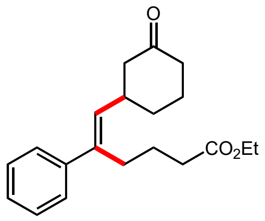

2H), 2.36 – 2.29 (m, 1H), 2.25 (t, J = 7.3 Hz, 2H), 2.23 – 2.18 (m, 1H), 2.16 – 2.07 (m, 1H), 1.94 – 1.86 (m, 1H), 1.82 – 1.73 (m, 1H), 1.66 – 1.61 (m, 2H), 1.60 – 1.51 (m, 1H), 1.23 (t, J = 7.2 Hz, 3H); ^{13}C NMR (101 MHz, $CDCl_3$) δ 210.98, 173.38, 142.18, 139.41, 132.30, 128.41, 127.16, 126.52, 60.41, 48.08, 41.27, 38.61, 33.69, 32.00, 29.12, 25.35, 23.91, 14.30; HR-MS (ESI) m/z calcd for $C_{20}H_{27}O_3$[M + H$^+$]: 315.1955, found: 315.1955.

Adamantan-1-yl)methyl (*E*)-7-(3-oxocyclohexyl)-6-phenyloct-6-enoate (7p)

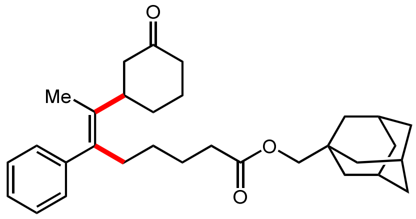

Following general procedure for Conditions A, the crude mixture was purified by flash chromatography on silica gel (eluted with ethyl acetate : hexanes = 1 : 40) to give the product **7p** (57.3 mg, 61% yield) as a colorless oil. ^{1}H NMR (600 MHz, $CDCl_3$) δ 7.30 – 7.28 (m, 2H), 7.21 – 7.19 (m, 1H), 7.04 – 7.03 (m, 2H), 3.61 (s, 2H), 3.09 – 2.99 (m, 1H), 2.48 – 2.40 (m, 2H), 2.39 – 2.26 (m, 4H), 2.25 – 2.19 (m, 2H), 2.17 – 2.10 (m, 1H), 2.00 – 1.93 (m, 3H), 1.80 – 1.69 (m, 7H), 1.67 – 1.60 (m, 4H), 1.59 – 1.51 (m, 3H), 1.51 – 1.47 (m, 5H), 1.45 (s, 3H); ^{13}C NMR (151 MHz, $CDCl_3$) δ 211.74, 173.81, 143.69, 136.61, 132.52, 128.83, 128.09, 126.16, 73.91, 46.35, 41.55, 41.29, 39.29, 37.01, 34.34, 33.42, 33.16, 29.88, 28.13, 28.07, 25.77, 24.96, 14.81; HR-MS (ESI) m/z calcd for $C_{31}H_{42}O_3Na$[M + Na$^+$]: 485.3026, found: 485.3023.

(*E*)-8-(3-oxocyclohexyl)-7-phenylnon-7-en-1-yl benzoate (7q)

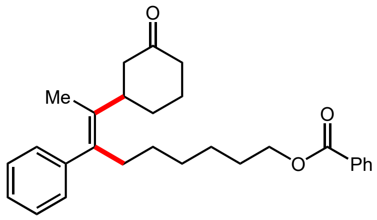

Following general procedure for Conditions A, the crude mixture was purified by flash chromatography on silica gel (eluted with ethyl acetate : hexanes = 1 : 40) to give the product **7q** (52.7 mg, 63% yield) as a colorless oil. ^{1}H NMR (400 MHz, $CDCl_3$) δ 8.07 – 7.99 (m, 2H), 7.59 – 7.52 (m, 1H), 7.47 – 7.40 (m, 2H), 7.34 – 7.27 (m, 2H), 7.24 – 7.18 (m, 1H), 7.08 – 7.01 (m, 2H), 4.26 (t, J = 6.6 Hz, 2H), 3.10 – 3.00 (m, 1H), 2.50 – 2.39 (m, 2H), 2.37 – 2.24 (m, 4H), 2.19 – 2.10 (m, 1H), 1.79 – 1.73 (m, 2H), 1.73 – 1.64 (m, 2H), 1.45 (s, 3H), 1.40 – 1.18 (m, 7H); ^{13}C NMR (101 MHz, $CDCl_3$) δ 211.93, 166.74, 137.05, 132.86, 132.20, 130.55, 129.60, 128.88, 128.41, 128.38, 128.08, 126.13, 65.09, 46.45, 41.60, 41.34, 33.74, 29.92, 29.25, 28.75, 28.52, 26.00, 25.85, 14.81; HR-MS (ESI) m/z calcd for $C_{28}H_{34}O_3Na$[M + Na$^+$]: 441.2400, found: 441.2396.

***tert*-butyl (*E*)-4-(4-(3-oxocyclohexyl)-3-phenylpent-3-en-1-yl)piperidine-1-carboxylate (7r)**

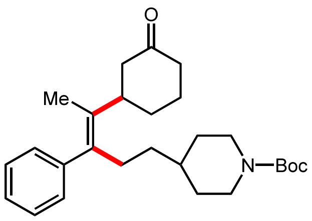

Following general procedure for Conditions A, the crude mixture was purified by flash chromatography on silica gel (eluted with ethyl acetate : hexanes = 1 : 30) to give the product **7r** (49.4 mg, 58% yield) as a colorless oil. ^{1}H NMR (400 MHz, $CDCl_3$) δ 7.33 – 7.27 (m, 2H), 7.24 – 7.18 (m, 1H), 7.06 – 7.00 (m, 2H), 4.01 (d, J = 13.0 Hz, 2H), 3.07 – 2.92 (m, 1H), 2.59 (t, J = 13.0 Hz, 2H), 2.50 – 2.39 (m, 2H), 2.38 – 2.22 (m, 4H), 2.20 – 2.10 (m, 1H), 1.80 – 1.70 (m, 3H), 1.59 – 1.50 (m, 2H), 1.45 (s, 3H), 1.42 (s, 9H), 1.31 – 1.22 (m, 1H), 1.21 – 1.07 (m, 2H), 1.04 – 0.90 (m, 2H); ^{13}C NMR (101 MHz, $CDCl_3$) δ 211.79, 154.87, 143.79, 136.97, 132.23, 128.83, 128.09, 126.18, 79.23, 46.46, 41.71, 41.31, 35.95, 35.49, 32.11, 32.10, 31.02, 29.95, 28.51, 25.90, 14.79; HR-MS (ESI) m/z calcd for $C_{27}H_{39}NO_3Na$[M + Na$^+$]: 448.2822, found: 448.2819.

(*Z*)-3-(2-(tetrahydro-2*H*-pyran-4-yl)hex-1-en-1-yl)cyclohexan-1-one (7s)

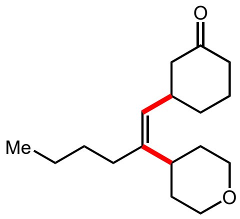

Following general procedure for Conditions A and 4-iodotetrahydro-2*H*-pyran (2.0 equiv) was used, the crude mixture was purified by flash chromatography on silica gel (eluted with ethyl acetate : hexanes = 1 : 50) to give the product **7s** (27.4 mg, 52% yield) as a colorless oil. ^{1}H NMR (400 MHz, $CDCl_3$) δ 4.96 (d, J = 9.4 Hz, 1H), 3.99 – 3.95 (m, 2H), 3.44 – 3.37 (m, 2H), 2.83 – 2.71 (m, 1H), 2.62 – 2.54 (m, 1H), 2.33 – 2.41 (m, 1H), 2.32 – 2.21 (m, 2H), 2.16 – 2.02 (m, 2H), 1.94 – 1.87 (m, 2H), 1.81 – 1.65 (m, 4H), 1.51 – 1.41 (m, 1H), 1.39 – 1.24 (m, 6H), 0.89 (t, J = 7.1 Hz, 3H); ^{13}C NMR (101 MHz, $CDCl_3$) δ 211.49, 142.49, 127.91, 68.32, 68.30, 48.72, 41.26, 37.88, 37.58, 32.45, 32.20, 31.64, 31.37, 31.29, 25.42, 22.78, 14.11; HR-MS (ESI) m/z calcd for $C_{17}H_{29}O_2$[M + H$^+$]: 265.2162, found: 265.2160.

(*E*)-3-(2-phenylhex-1-en-1-yl)cycloheptan-1-one (8a)

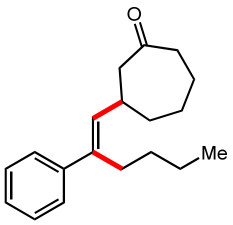

Following general procedure for Conditions C with slight modification: After 5 h, 1.0 equiv of alkyne was added. The crude mixture was purified by flash chromatography on silica gel (eluted with ethyl acetate : hexanes = 1% ~2%) to give the product **8a** (32.9 mg, 61% yield) as a colorless oil. ^{1}H NMR (400 MHz, $CDCl_3$) δ 7.24 – 7.20 (m, 4H), 7.19 – 7.13 (m, 1H), 5.39 (d, J = 9.5 Hz, 1H), 2.72 – 2.63 (m, 1H), 2.58 – 2.51 (m, 1H), 2.49 – 2.45 (m, 2H), 2.45 – 2.39 (m, 3H), 1.95 – 1.82 (m, 3H), 1.59 – 1.54 (m, 1H), 1.49 – 1.35 (m, 2H), 1.30 – 1.18 (m, 4H), 0.78 (t, J = 6.8 Hz, 3H); ^{13}C NMR (101 MHz, $CDCl_3$) δ 213.75, 142.99, 139.30, 132.59, 128.26, 126.82, 126.57, 50.27, 44.19, 37.87, 35.79, 30.96, 29.82, 28.60, 24.13, 22.73, 14.03; HR-MS (ESI) m/z calcd for $C_{19}H_{27}O$[M + H$^+$]: 271.2056, found: 271.2054.

(*E*)-5-methyl-7-phenylundec-6-en-3-one (8b)

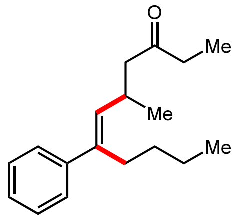

Following general procedure for Conditions C with slight modification: After 5 h, 1.0 equiv of alkyne was added. The crude mixture was purified by flash chromatography on silica gel (eluted with ethyl acetate : hexanes = 1% ~2%) to give the product **8b** (29.9 mg, 58% yield) as a colorless oil. ^{1}H NMR (600 MHz, $CDCl_3$) δ 7.33 – 7.27 (m, 4H), 7.25 – 7.20 (m, 1H), 5.38 (d, J = 9.8 Hz, 1H), 3.17 – 3.07 (m, 1H), 2.59 – 2.52 (m, 1H), 2.50 – 2.38 (m, 5H), 1.34 – 1.25 (m, 4H), 1.09 – 1.02 (m, 6H), 0.87 (t, J = 6.6 Hz, 3H); ^{13}C NMR (151 MHz, $CDCl_3$) δ 210.86, 143.22, 139.81, 133.31, 128.22, 126.71, 126.55, 50.06, 36.88, 31.15, 29.78, 29.56, 22.79, 21.45, 14.05, 7.78; HR-MS (ESI) m/z calcd for $C_{18}H_{27}O[M + H^+]$: 259.2056, found: 259.2053.

(*E*)-4-methyl-6-phenyldec-5-en-2-one (8c)

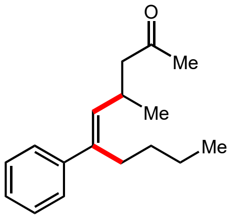

Following general procedure for Conditions C with slight modification: After 5 h, 1.0 equiv of alkyne was added. The crude mixture was purified by flash chromatography on silica gel (eluted with ethyl acetate : hexanes = 1% ~2%) to give the product **8c** (25.9 mg, 53% yield) as a colorless oil. ^{1}H NMR (400 MHz, $CDCl_3$) δ 7.32 – 7.27 (m, 4H), 7.25 – 7.20 (m, 1H), 5.39 (d, J = 9.8 Hz, 1H), 3.16 – 3.05 (m, 1H), 2.63 – 2.49 (m, 2H), 2.48 – 2.43 (m, 2H), 2.14 (s, 3H), 1.35 – 1.27 (m, 4H), 1.07 (d, J = 6.6 Hz, 3H), 0.86 (t, J = 6.8 Hz, 3H); ^{13}C NMR (101 MHz, $CDCl_3$) δ 208.28, 143.15, 139.89, 133.15, 128.23, 126.74, 126.55, 51.33, 31.13, 30.80, 29.79, 29.44, 22.80, 21.37, 14.05; HR-MS (ESI) m/z calcd for $C_{17}H_{24}ONa[M + Na^+]$: 267.1719, found: 267.1718.

(*E*)-4-methyl-6-(p-tolyl)dec-5-en-2-one (8d)

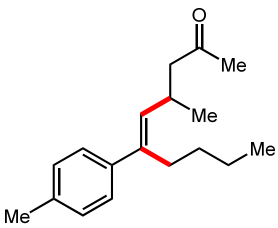

Following general procedure for Conditions C with slight modification: After 3 h, 1.0 equiv alkyne was added. The crude mixture was purified by flash chromatography on silica gel (eluted with ethyl acetate : hexanes = 1% ~2%) to give the product **8d** (31.5 mg, 61% yield) as a colorless oil. ^{1}H NMR (400 MHz, $CDCl_3$) δ 7.23 – 7.18 (m, 2H), 7.13 – 7.08 (m, 2H), 5.36 (d, J = 9.8 Hz, 1H), 3.15 – 3.03 (m, 1H), 2.57 – 2.39 (m, 4H), 2.33 (s, 3H), 2.14 (s, 3H), 1.35 – 1.27 (m, 4H), 1.06 (d, J = 6.7 Hz, 3H), 0.86 (t, J = 6.8 Hz, 3H); ^{13}C NMR (101 MHz, $CDCl_3$) δ 208.39, 140.19, 139.65, 136.39, 132.46, 128.93, 126.39, 51.39, 31.15,

30.79, 29.75, 29.45, 22.81, 21.40, 21.11, 14.05; HR-MS (ESI) m/z calcd for $C_{18}H_{27}O[M + H^{+}]$: 259.2056, found: 259.2054.

(*E*)-6-(4-methoxyphenyl)-4-methyldec-5-en-2-one (8e)

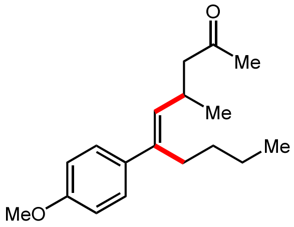

Following general procedure for Conditions C with slight modification: After 3 h, 1.0 equiv of alkyne was added. The crude mixture was purified by flash chromatography on silica gel (eluted with ethyl acetate : hexanes = 1% ~2%) to give the product **8e** (30.7 mg, 56% yield) as a colorless oil. ^{1}H NMR (400 MHz, $CDCl_3$) δ 7.26 – 7.21 (m, 2H), 6.86 – 6.81 (m, 2H), 5.32 (d, *J* = 9.7 Hz, 1H), 3.80 (s, 3H), 3.14 – 3.02 (m, 1H), 2.56 – 2.38 (m, 4H), 2.13 (s, 3H), 1.36 – 1.26 (m, 4H), 1.05 (d, *J* = 6.6 Hz, 3H), 0.86 (t, *J* = 6.8 Hz, 3H); ^{13}C NMR (101 MHz, $CDCl_3$) δ 208.42, 158.58, 139.19, 135.56, 131.86, 127.51, 113.60, 55.34, 51.44, 31.15, 30.78, 29.79, 29.45, 22.80, 21.45, 14.07; HR-MS (ESI) m/z calcd for $C_{18}H_{27}O_2[M + H^{+}]$: 275.2006, found: 275.2002.

3-(2-butylhex-1-en-1-yl)-4,4-dimethylcyclohexan-1-one (8f)

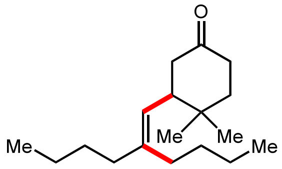

Following general procedure for Conditions A, the crude mixure was purified by flash chromatography on silica gel (eluted with ethyl acetate: hexanes = 1% ~ 2%) to give the product **8f** (27.5 mg, 52% yield) as a colorless oil. ^{1}H NMR (600 MHz, $CDCl_3$) δ 4.96 (d, *J* = 10.2 Hz, 1H), 2.54 – 2.49 (m, 1H), 2.44 – 2.38 (m, 1H), 2.31 – 2.27 (m, 1H), 2.26 – 2.22 (m, 1H), 2.19 – 2.15 (m, 1H), 2.07 – 1.94 (m, 3H), 1.94 – 1.86 (m, 1H), 1.77 – 1.73 (m, 1H), 1.65 – 1.57 (m, 2H), 1.39 – 1.31 (m, 2H), 1.33 – 1.23 (m, 6H), 1.03 (s, 3H), 0.95 (s, 3H), 0.91 – 0.89 (m, 6H); ^{13}C NMR (151 MHz, $CDCl_3$) δ 212.09, 141.43, 124.49, 45.33, 44.69, 40.05, 38.38, 36.67, 33.29, 30.73, 30.57, 29.92, 29.05, 23.00, 22.48, 20.01, 14.12, 14.07; HR-MS (ESI) m/z calcd for $C_{18}H_{33}O[M + H^{+}]$: 265.2526, found: 265.2522

3-(2-butylhex-1-en-1-yl)spiro[5.5]undecan-1-one (8g)

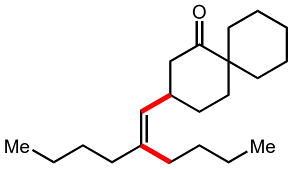

Following general procedure for Conditions A, the crude mixure was purified by flash chromatography on silica gel (eluted with ethyl acetate: hexanes= 1% ~ 2%) to give the product **8g** (40.2 mg, 66% yield) as a colorless oil. ^{1}H NMR (400 MHz, $CDCl_3$) δ 4.97 (d, *J* = 9.2 Hz, 1H), 2.71 – 2.58 (m, 1H), 2.40 – 2.34 (m, 1H), 2.18 – 2.13 (m, 1H), 2.00 – 1.91 (m, 5H), 1.87 – 1.81 (m, 1H), 1.72 – 1.49 (m, 5H), 1.46 – 1.37 (m, 4H), 1.35 – 1.24 (m, 10H), 1.15 – 1.09 (m, 1H), 0.91 – 0.87 (m, 6H); ^{13}C NMR (151 MHz, $CDCl_3$) δ 216.18, 139.87, 127.97,

48.17, 45.17, 39.31, 37.13, 36.40, 33.84, 33.60, 31.05, 30.41, 30.08, 27.94, 26.38, 22.93, 22.49, 22.19, 22.07, 14.12, 14.10; HR-MS (ESI) m/z calcd for $C_{21}H_{37}O[M + H^+]$: 305.2839, found: 305.2835.

(*E*)-6-phenyl-4-propyldec-5-en-2-one (8h)

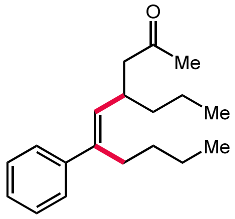

Following general procedure for Conditions C with slight modification: After 3 h, 1.0 equiv alkyne was added. The crude mixture was purified by flash chromatography on silica gel (eluted with ethyl acetate : hexanes = 1% ~2%) to give the product **8h** (24.5 mg, 45% yield) as a colorless oil. ^1H NMR (400 MHz, $CDCl_3$) δ 7.32 – 7.29 (m, 4H), 7.25 – 7.20 (m, 1H), 5.31 (d, *J* = 10.1 Hz, 1H), 3.05 – 2.94 (m, 1H), 2.59 – 2.39 (m, 4H), 2.14 (s, 3H), 1.49 – 1.36 (m, 1H), 1.35 – 1.23 (m, 6H), 0.91 (t, *J* = 6.8 Hz, 3H), 0.86 (t, *J* = 8.2 Hz, 3H); ^{13}C NMR (101 MHz, $CDCl_3$) δ 208.46, 143.29, 141.11, 132.24, 128.21, 126.71, 126.58, 50.03, 38.23, 34.55, 31.02, 30.93, 29.89, 22.89, 20.63, 14.30, 14.04; HR-MS (ESI) m/z calcd for $C_{19}H_{29}O[M + H^+]$: 273.2213, found: 273.2210.

(*E*)-3-methyl-5-phenyl-1-(1*H*-pyrrol-1-yl)non-4-en-1-one (8i)

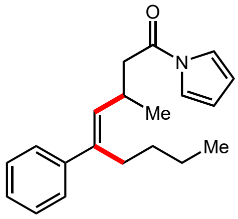

Following general procedure for Conditions C with slight modification: After 5 h, 1.0 equiv alkyne was added. The crude mixture was purified by flash chromatography on silica gel (eluted with ethyl acetate : hexanes = 1% ~2%) to give the product **8i** (12.9 mg, 22% yield) as a colorless oil. ^1H NMR (600 MHz, $CDCl_3$) δ 7.36 – 7.31 (m, 2H), 7.30 – 7.27 (m, 4H), 7.24 – 7.21 (m, 1H), 6.30 – 6.29 (m, 2H), 5.44 (d, *J* = 9.9 Hz, 1H), 3.33 – 3.22 (m, 1H), 2.85 (d, *J* = 7.0 Hz, 2H), 2.60 – 2.41 (m, 2H), 1.29 – 1.22 (m, 4H), 1.18 (d, *J* = 6.6 Hz, 3H), 0.84 (t, *J* = 6.8 Hz, 3H); ^{13}C NMR (151 MHz, $CDCl_3$) δ 169.48, 143.03, 140.80, 132.24, 128.23, 126.85, 126.61, 119.19, 113.19, 42.38, 31.10, 30.34, 29.90, 22.78, 21.45, 14.02; HR-MS (ESI) m/z calcd for $C_{20}H_{26}NO[M + H^+]$: 296.2009, found: 296.2007.

(2-butylhex-1-en-1-yl)cyclohexane (9a)

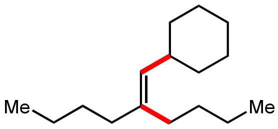

General Procedure for Synthesis of Unfunctionalized Tri- and Tetrasubstituted Alkenes, the crude mixture was purified by flash chromatography on silica gel (eluted with ethyl acetate : hexanes = 1%) to give the product **9a** (14.5 mg, 65% yield) as a colorless oil. ^1H NMR (400 MHz, $CDCl_3$) δ 4.92 (d, *J* = 9.3 Hz, 1H), 2.20 – 2.09 (m, 1H), 2.03 – 1.96 (m, 2H), 1.96 – 1.90 (m, 2H), 1.74 – 1.58 (m, 4H), 1.39 – 1.24 (m, 10H), 1.23 – 1.12 (m, 2H), 1.08 – 0.97 (m, 2H), 0.94 – 0.86 (m,

6H); ^{13}C NMR (101 MHz, $CDCl_3$) δ 137.69, 131.12, 36.81, 36.55, 33.92, 31.23, 30.61, 30.02, 26.26, 26.25, 22.97, 22.55, 14.17, 14.14; HR-MS (EI) m/z calcd for $C_{16}H_{30}[M^+]$: 222.2348, found: 222.2319.

(*E*)-(1-cyclohexylhex-1-en-2-yl)benzene (9b)

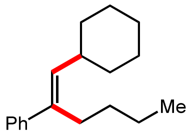

General Procedure for Synthesis of Unfunctionalized Tri- and Tetrasubstituted Alkenes, the crude mixture was purified by flash chromatography on silica gel (eluted with ethyl acetate : hexanes = 1%) to give the product **9b** (15.8 mg, 65% yield) as a colorless oil. ^{1}H NMR (400 MHz, $CDCl_3$) δ 7.37 – 7.26 (m, 4H), 7.24 – 7.18 (m, 1H), 5.47 (d, J = 9.4 Hz, 1H), 2.54 – 2.47 (m, 2H), 2.40 – 2.29 (m, 1H), 1.79 – 1.65 (m, 5H), 1.39 – 1.28 (m, 6H), 1.23 – 1.09 (m, 3H), 0.87 (t, J = 7.2 Hz, 3H); ^{13}C NMR (101 MHz, $CDCl_3$) δ 143.60, 138.36, 135.16, 128.16, 126.50, 126.41, 37.70, 33.57, 31.39, 29.66, 26.19, 26.10, 22.78, 14.07; HR-MS (EI) m/z calcd for $C_{18}H_{26}[M^+]$: 242.2035, found: 242.2021.

(*E*)-(3-(cyclohexylmethylene)heptyl)benzene (9c)

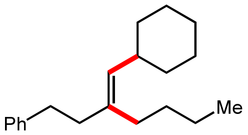

General Procedure for Synthesis of Unfunctionalized Tri- and Tetrasubstituted Alkenes, the crude mixture was purified by flash chromatography on silica gel (eluted with ethyl acetate : hexanes = 1%) to give the product **9c** (16.2 mg, 60% yield) as a colorless oil. ^{1}H NMR (400 MHz, $CDCl_3$) δ 7.26 – 7.21 (m, 2H), 7.18 – 7.12 (m, 3H), 4.92 (d, J = 9.3 Hz, 1H), 2.70 – 2.63 (m, 2H), 2.25 – 2.19 (m, 2H), 2.18 – 2.08 (m, 1H), 2.02 (t, J = 7.4 Hz, 2H), 1.73 – 1.53 (m, 4H), 1.39 – 1.19 (m, 7H), 1.17 – 1.07 (m, 1H), 1.03 – 0.92 (m, 2H), 0.89 (t, J = 6.8 Hz, 3H); ^{13}C NMR (101 MHz, $CDCl_3$) δ 142.74, 136.86, 131.82, 128.54, 128.24, 125.66, 38.73, 36.82, 35.09, 33.77, 31.20, 30.23, 26.23, 26.20, 22.94, 14.17; HR-MS (EI) m/z calcd for $C_{20}H_{30}[M^+]$: 270.2348, found: 270.2338.

(*Z*)-(5-propylnon-4-en-4-yl)cyclohexane (9d)

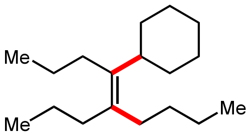

General Procedure for Synthesis of Unfunctionalized Tri- and Tetrasubstituted Alkenes, the crude mixture was purified by flash chromatography on silica gel (eluted with ethyl acetate : hexanes = 1%) to give the product **9d** (15.5 mg, 62% yield) as a colorless oil. ^{1}H NMR (400 MHz, $CDCl_3$) δ 2.42 – 2.31 (m, 1H), 2.01 – 1.89 (m, 4H), 1.89 – 1.83 (m, 2H), 1.78 – 1.70 (m, 2H), 1.69 – 1.62 (m, 1H), 1.49 – 1.41 (m, 2H), 1.40 – 1.23 (m, 13H), 0.96 – 0.84 (m, 9H); ^{13}C NMR (101 MHz, $CDCl_3$) δ 138.33, 133.18, 41.60, 34.52, 32.14, 32.02, 30.92, 30.80, 27.07, 26.45, 24.57, 23.11, 22.28, 14.98, 14.49, 14.21; HR-MS (EI) m/z calcd for $C_{18}H_{34}[M^+]$: 250.2661, found: 250.2649.

(*E*)-(2-cyclohexylhept-2-en-3-yl)benzene (9e)

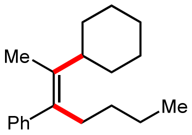

General Procedure for Synthesis of Unfunctionalized Tri- and Tetrasubstituted Alkenes, the crude mixture was purified by flash chromatography on silica gel (eluted with ethyl acetate : hexanes = 1%) to give the product **9e** (16.4 mg, 64% yield) as a colorless oil. ^{1}H NMR (400 MHz, $CDCl_3$) δ 7.33 – 7.26 (m, 2H), 7.22 – 7.16 (m, 1H), 7.09 – 7.04 (m, 2H), 2.61 – 2.51 (m, 1H), 2.33 (t, *J* = 7.3 Hz, 2H), 1.85 – 1.76 (m, 2H), 1.75 – 1.67 (m, 1H), 1.61 – 1.58 (m, 1H), 1.44 – 1.32 (m, 7H), 1.31 – 1.15 (m, 6H), 0.84 (t, *J* = 6.8 Hz, 3H); ^{13}C NMR (101 MHz, $CDCl_3$) δ 144.99, 135.89, 135.01, 129.20, 127.88, 125.64, 41.16, 33.49, 31.37, 31.04, 26.90, 26.42, 22.67, 15.35, 14.11; HR-MS (EI) m/z calcd for $C_{19}H_{28}$[M $^+$]: 256.2191, found: 256.2203.

(*E*)-(7-methyldec-5-en-5-yl)benzene (9f)

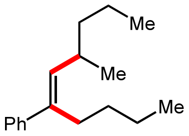

General Procedure for Synthesis of Unfunctionalized Tri- and Tetrasubstituted Alkenes, the crude mixture was purified by flash chromatography on silica gel (eluted with ethyl acetate : hexanes = 1%) to give the product **9f** (13.8 mg, 60% yield) as a colorless oil. ^{1}H NMR (600 MHz, $CDCl_3$) δ 7.36 – 7.32 (m, 2H), 7.31 – 7.27 (m, 2H), 7.23 – 7.19 (m, 1H), 5.39 (d, *J* = 9.8 Hz, 1H), 2.57 – 2.51 (m, 1H), 2.51 – 2.44 (m, 2H), 1.40 – 1.27 (m, 8H), 1.01 (d, *J* = 6.7 Hz, 3H), 0.90 (t, *J* = 7.2 Hz, 3H), 0.86(t, *J* = 6.6 Hz, 3H); ^{13}C NMR (151 MHz, $CDCl_3$) δ 143.69, 138.70, 135.84, 128.17, 126.51, 126.41, 40.25, 32.78, 31.28, 29.73, 22.84, 21.52, 20.86, 14.42, 14.07; HR-MS (EI) m/z calcd for $C_{17}H_{26}$[M$^+$]: 230.2035, found: 230.2008.

N-3-(2-butylhex-1-en-1-yl)cyclohexyl)aniline (10)

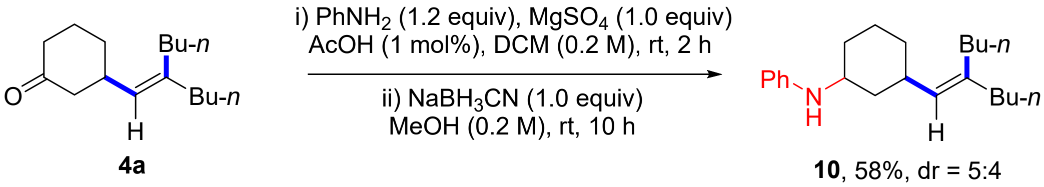

To a 10 mL Schlenk tube, **4a** (0.2 mmol, 47.2 mg), $PhNH_2$ (1.2 equiv, 22 µL), $MgSO_4$ (1.0 equiv, 2.4 mg) and AcOH (1 mol%, 1.2 µL) were dissolved in dry DCM (1.0 mL). The mixture was stirred at room temperature for 2 h before evapoartion of the solvent. The residue was directly dissolved in MeOH (1.0 mL) and $NaBH_3CN$ (1.0 equiv, 12.6 mg) was added. The mixture was stirred at room temperature for 10 h. The reaction was evaporated under reduced pressure, the residue was dissolved in water and neutralized with NaOH (2 M) aqeous solution to PH = 7. The solution was extracted with DCM (20 mL) and the organic layer was

concentrated and purified by column chromatography to give 23.1 mg (58% yield, dr = 5:4) **10** as colourless oil. ^{1}H NMR (400 MHz, $CDCl_3$) δ 7.20 – 7.11 (m, 2H), 6.71 – 6.56 (m, 3H), 5.08 (d, J = 9.1 Hz, $1H_{maj}$), 4.91 (d, J = 9.2 Hz, $1H_{min}$), 3.71 – 3.64 (m, $1H_{maj}$), 3.33 – 3.23 (m, $1H_{min}$), 2.65 – 2.52 (m, $1H_{maj}$), 2.39 – 2.26 (m, $1H_{min}$), 2.17 – 2.08 (m, $1H_{maj}$), 2.07 – 1.91 (m, 5H), 1.87 – 1.78 (m, $1H_{min}$), 1.71 – 1.53 (m, 4H), 1.42 – 1.22 (m, 10H), 0.96 – 0.84 (m, 6H); ^{13}C NMR (101 MHz, $CDCl_3$) δ 147.43, 147.37, 139.08, 138.57, 129.73, 129.34, 129.31, 128.82, 117.04, 116.92, 113.28, 113.26, 52.00, 47.72, 40.86, 37.77, 36.58, 36.48, 36.14, 33.34, 33.19, 32.54, 31.20, 31.18, 31.03, 30.87, 30.59, 30.54, 30.14, 29.96, 24.98, 22.99, 22.96, 22.54, 22.52, 20.67, 14.19, 14.13; HR-MS (ESI) m/z calcd for $C_{22}H_{36}N[M + H^+]$: 314.2842, found: 314.2838.

3-(2-butylhex-1-en-1-yl)cyclohexan-1-ol (11)

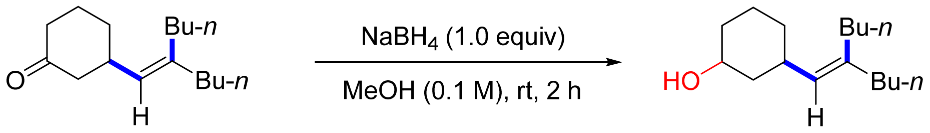

4a **11**, 90%, dr = 10:1

To a 10 mL Schlenk tube, **4a** (0.1 mmol, 23.6 mg) was dissolved in MeOH (1 mL), then $NaBH_4$ (1.0 equiv, 3.78 mg) was added. The mixture was stirred at room temperature for 2 h. After completion, MeOH was evaporated under reduced pressure. ^{1}H NMR of the crude mixture revealed a diastereoselectivity ratio of 10 : 1. The residue was purified by flash chromatography (hexanes : ethyl acetate = 20:1) to give 21.4 mg (90% yield, dr = 10:1) **11** as a yellow oil. ^{1}H NMR (400 MHz, $CDCl_3$) δ 4.90 (d, J = 9.2 Hz, 1H), 3.65 – 3.53 (m, 1H), 2.29 – 2.16 (m, 1H), 2.02 – 1.90 (m, 5H), 1.89 – 1.82 (m, 1H), 1.80 – 1.71 (m, 1H), 1.56 – 1.47 (m, 1H), 1.39 – 1.22 (m, 10H), 1.19 – 1.09 (m, 1H), 1.06 – 0.95 (m, 1H), 0.94 – 0.85 (m, 6H); ^{13}C NMR (101 MHz, $CDCl_3$) δ 138.48, 129.58, 70.62, 43.06, 36.43, 35.52, 35.37, 32.65, 31.11, 30.49, 30.00, 24.12, 22.93, 22.48, 14.14, 14.10; HR-MS (ESI) m/z calcd for $C_{16}H_{31}O[M + H^+]$: 239.2369, found: 239.2367.

1-(2-butylhex-1-en-1-yl)-3-methylenecyclohexane (12)

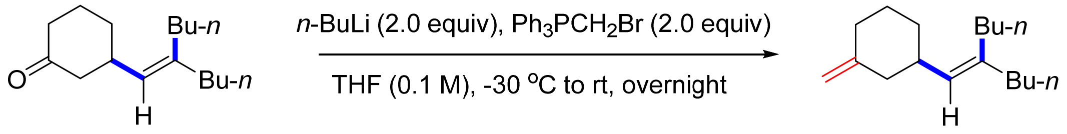

4a **12**, 76%

To a 10 mL Schlenk tube, methyltriphenyl phosphoniumbromide (2.0 equiv, 0.14 g) was added. Dry THF (1 mL) was added to the flask under nitrogen, and the resulting suspension was stirred

at -30 °C, followed by the dropwise addition of *n*-BuLi (2.0 equiv, 1.6 M in hexane). During dropwise addition of base the reaction turned into a yellow suspension, finally became a clear red-orange solution. **4a** (0.2 mmol, 47.2 mg) in dry and deoxygenated THF (1 mL) was added dropwise to the mixture over minutes. After 1 h, the mixture was warmed up to room temperature slowly. When the reaction was completed as determined by TLC (10-20 h), deionized water (2 mL) was added. The organic layer was washed with water (3 times), brine (3 times), dried over magnesium sulfate, and the filtrate concentrated in vacuo, purification by column chromatography to give 34.6 mg (76% yield) **12** as colourless oil. ^{1}H NMR (400 MHz, $CDCl_3$) δ 4.97 (d, *J* = 9.2 Hz, 1H), 4.65 – 4.59 (m, 2H), 2.34 – 2.17 (m, 3H), 2.03 – 1.92 (m, 5H), 1.85 – 1.75 (m, 2H), 1.68 – 1.60 (m, 1H), 1.41 – 1.25 (m, 9H), 1.23 – 1.12 (m, 1H), 0.94 – 0.87 (m, 6H); ^{13}C NMR (101 MHz, $CDCl_3$) δ 149.29, 138.54, 129.93, 107.09, 42.32, 38.36, 36.53, 34.82, 33.38, 31.18, 30.58, 30.06, 27.13, 22.94, 22.54, 14.17, 14.14; HR-MS (ESI) m/z calcd for $C_{17}H_{31}$[M + H$^+$]: 235.2420, found: 235.2419.

3-(3,3-dibutyloxiran-2-yl)cyclohexan-1-one (13)

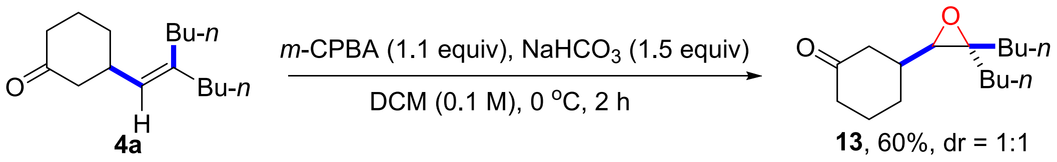

To a 10 mL Schlenk tube, **4a** (0.2 mmol, 47.2 mg), DCM (2 mL) and $NaHCO_3$ (1.5 equiv, 25.2 mg) were added. The mixture was cooled to 0 °C before *m*-CPBA (1.1 equiv, 37.9 mg) was added. After being stirred at 0 °C for 2 h, the mixture was diluted with DCM (10 mL) and quenched with saturated Na_2SO_3 (5 mL). The mixture was further stirred for 10 min and the organic layer was washed with brine, dried over magnesium sulfate, and the filtrate was concentrated in vacuo. The residue was purified by column chromatography to give 30.2 mg (60% yield, dr = 1:1) **13** as colourless oil. ^{1}H NMR (400 MHz, $CDCl_3$) δ 2.64 – 2.57 (m, 0.5H), 2.52 (dd, *J* = 9.0, 3.0 Hz, 1H), 2.43 – 2.35 (m, 1H), 2.35 – 2.28 (m, 1H), 2.28 – 2.18 (m, 1H), 2.17 – 2.07 (m, 2H), 1.87 – 1.79 (m, 0.5H), 1.78 – 1.65 (m, 3H), 1.60 – 1.49 (m, 2H), 1.41 – 1.25 (m, 10H), 0.93 – 0.87 (m, 6H). ^{13}C NMR (101 MHz, $CDCl_3$) δ 210.38, 210.24, 67.07, 64.20, 64.11, 45.28, 44.22, 41.51, 41.41, 38.27, 38.23, 35.14, 35.10, 29.66, 29.48, 28.94, 28.00, 27.44, 27.40, 27.31, 27.27, 25.20, 25.00, 23.16, 23.14, 22.87, 22.83, 14.11, 14.09, 14.06; HR-MS (ESI) m/z calcd for $C_{16}H_{29}O_2$[M + H$^+$]: 253.2162, found: 253.2160.

5-(2-butylhex-1-en-1-yl)cyclohex-2-en-1-one (14)

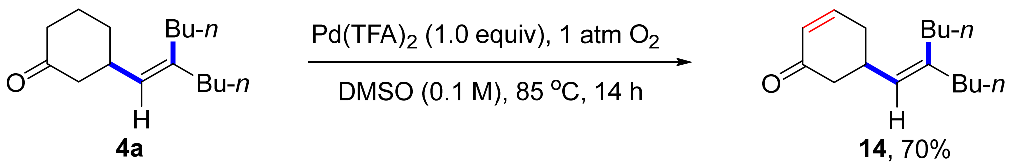

To a 10 mL Schlenk tube, **4a** (0.1 mmol, 47.2 mg), Pd(TFA)$_2$ (0.1 mmol, 33.2 mg) and DMSO (1 mL) were added. The tube was evacuated and back-filled with oxygen (repeat this process three times) and sealed with an oxygen balloon (1 atm). The reaction mixture was stirred at 85 °C for 14 h. After completion, the reaction mixture was filtered through a short plug of celite and concentrated under vacuo. The residue was purified by column chromatography to give 16.4 mg (70% yield) **14** as colorless oil. ^{1}H NMR (400 MHz, CDCl$_3$) δ 7.01 – 6.93 (m, 1H), 6.06 – 5.98 (m, 1H), 5.03 (d, J = 9.3 Hz, 1H), 3.07 – 2.93 (m, 1H), 2.46 – 2.30 (m, 2H), 2.27 – 2.09 (m, 2H), 2.03 – 1.92 (m, 4H), 1.36 – 1.25 (m, 8H), 0.89 (t, J = 7.2 Hz, 6H). ^{13}C NMR (101 MHz, CDCl$_3$) δ 199.86, 150.01, 141.38, 129.74, 126.67, 45.01, 36.43, 34.38, 32.97, 31.09, 30.37, 30.10, 22.95, 22.50, 14.10, 14.08; HR-MS (ESI) m/z calcd for $C_{16}H_{27}O$[M + H$^+$]: 235.2056, found: 235.2055.

3-(2-butylhex-1-en-1-yl)phenol (15)

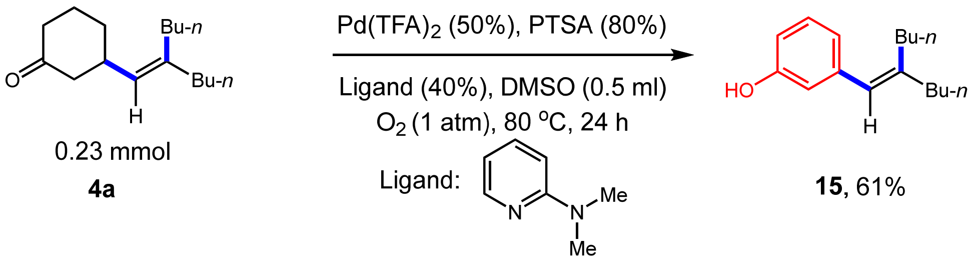

To a 10 mL Schlenk tube, **4a** (0.23 mmol, 55 mg), Pd(TFA)$_2$ (0.12 mmol, 38.7 mg), PTSA (0.18 mmol, 35.7 mg), 2-(N,N-dimethylamino)pyridine (0.09 mmol, 11.6 μl) and DMSO (0.5 mL) were added. The tube was sealed with a rubber septum and O_2 was bubbled through the solutionfor ca. 10 min. The reaction was heated in an oil bath to 80 °C with vigorous stirring under a balloon of O_2 for 24 h. After completion, the reaction mixture was filtered through a short plug of celite and concentrated under vacuo. The residue was purified by column chromatography to give 32.6 mg (61% yield) **15** as colorless oil. ^{1}H NMR (600 MHz, , CDCl$_3$) δ 7.17 (t, J = 7.8 Hz, 1H), 6.78 (d, J = 7.6 Hz, 1H), 6.71 – 6.63 (m, 2H), 6.20 (s, 1H), 2.25 – 2.20 (t, J = 7.7 Hz, 2H), 2.15 (t, J = 7.7 Hz, 2H), 1.52 – 1.40 (m, 4H), 1.41 – 1.27 (m, 4H), 0.95 (t, J = 7.3 Hz, 3H), 0.89 (t, J = 7.3 Hz, 3H); ^{13}C NMR (151 MHz, CDCl$_3$) δ 155.30, 144.48, 140.58, 129.25, 124.34, 121.40, 115.55, 112.82, 37.01, 30.57, 30.51, 30.47, 22.96, 22.63, 14.13, 14.06; HR-MS (ESI) m/z calcd for $C_{16}H_{23}O$[M - H$^+$]: 231.1754, found: 231.1748.

2.3 Control and mechanistic experiments

Deuterium experiments

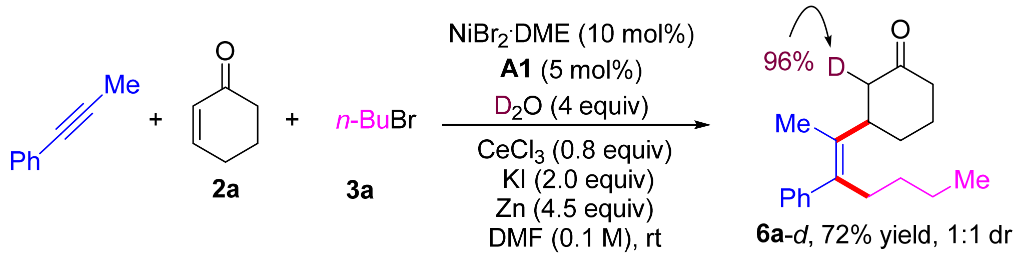

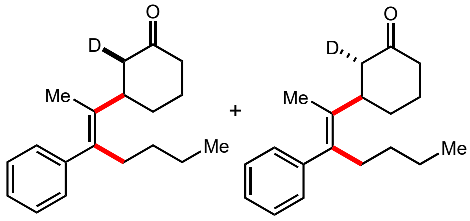

Following general procedure for Conditions A: The reaction was operated in a nitrogen-filled glove box. 4-Cyanopyridine (5 mol%, 1.04 mg), Zinc (4.5 equiv, 58.2 mg), $CeCl_3$ (0.8 equiv, 39.4 mg), KI (2.0 equiv, 66.4 mg) and $NiBr_2$·DME (10 mol%, 6.17 mg) were added to an oven-dried vial containing a magnetic stirring bar. Anhydrous DMF (0.1 M, 2 mL) was added and rapid stirring was commenced. Then D_2O (4.0 equiv, 14.4 μL), 1-phenyl-1-propyne (2.0 equiv, 50.5 μL), **2a** (0.2 mmol, 19.5 μL) and **3a** (2.0 equiv, 45.6 μL) were added sequentially via syringe. The reaction was stirred vigorously for 48 h at room temperature. The reaction was diluted with ethyl acetate (30 mL) and washed with brine (50 mL), aqueous layer was extracted twice with ethyl acetate (20 mL).The combined organic layer was dried over magnesium sulfate, filtrated, evaporated and purified by flash chromatography with hexane:ethyl acetate mixtures as eluent to give 39.1 mg (72% yield) **6a**-*d* as colourless oil. The deuterium ratio was about 96% and calculated by ^{1}H NMR and HR-MS (ESI). ^{1}H NMR (400 MHz, $CDCl_3$) δ 7.35 – 7.27 (m, 2H), 7.24 – 7.17 (m, 1H), 7.08 – 7.02 (m, 2H), 3.14 – 2.96 (m, 1H), 2.50 – 2.23 (m, 5.05H), 2.20 – 2.09 (m, 1H), 1.82 – 1.71 (m, 3H), 1.45 (s, 3H), 1.29 – 1.13 (m, 4H), 0.82 (t, J = 6.9 Hz, 3H); ^{13}C NMR (101 MHz, $CDCl_3$) δ 212.01 (211.94), 144.07, 137.26 (137.25), 131.99, 128.88, 128.03, 126.05, 46.10 (t, J = 19.99 Hz, 1C), 41.52 (41.49), 41.38 (41.34), 33.61, 30.83, 29.91, 25.87, 22.65, 14.77, 14.03; HR-MS (ESI) m/z calcd for $C_{19}H_{26}DO[M + H^+]$: 272.2119, found: 272.2116.

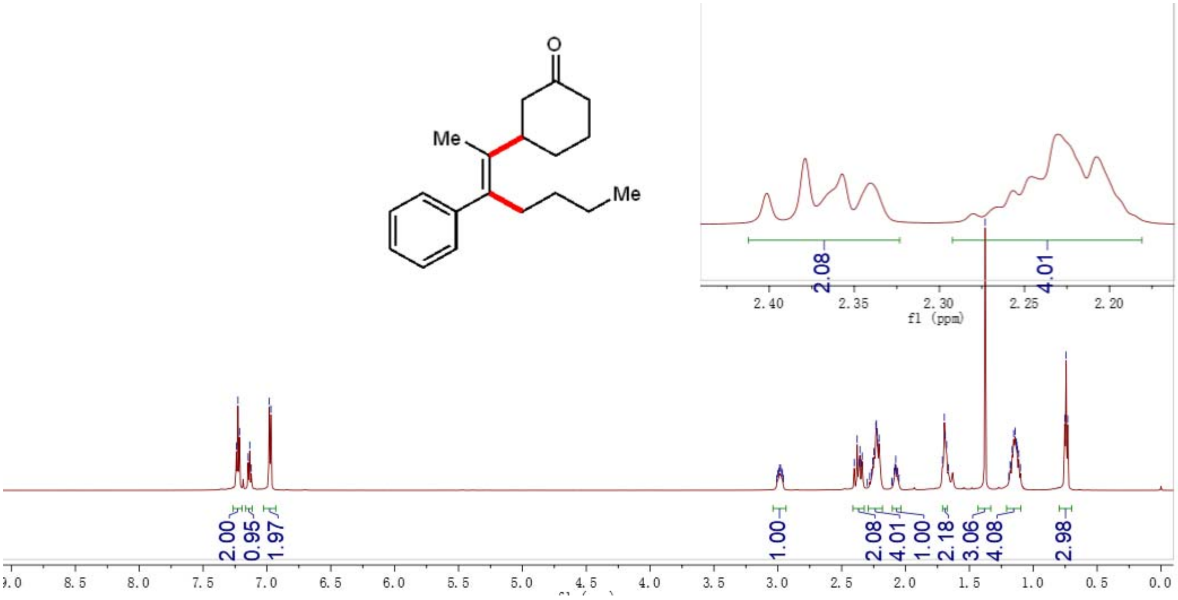

Supplementary Figure 1. ^{1}H NMR (400 MHz, $CDCl_3$) of **6a** (Before deuterium)

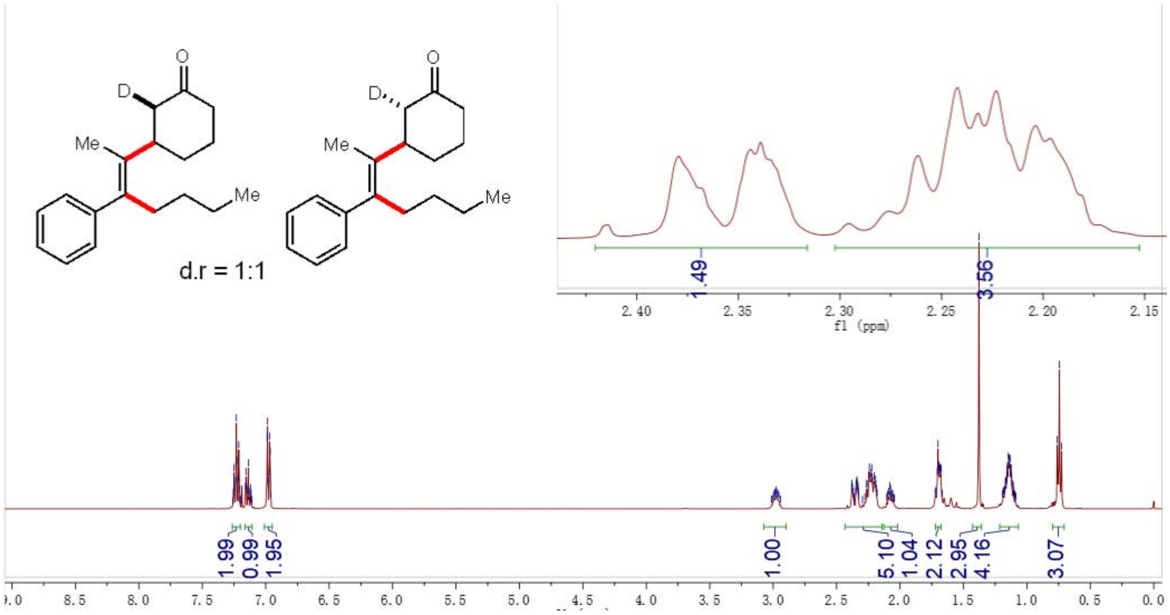

Supplementary Figure 2. ^{1}H NMR (400 MHz, $CDCl_3$) of **6a**-*d* (After deuterium)

Simulated value：$[M+H]^+$

Simulated: C18 H24 O1 D1

m/z= 258.0157-264.3970

m/z	Intensity	Relative
258.1963	819689.1	100.00
259.1996	159579.6	19.47
259.2005	312.2	0.04
259.2025	2262.6	0.28
260.2005	1684.5	0.21
260.2030	14670.7	1.79
260.2038	60.8	0.01
260.2059	440.5	0.05
260.2088	3.0	0.00
261.2039	327.9	0.04
261.2063	846.3	0.10
261.2068	4.6	0.00
261.2072	5.6	0.00
261.2093	40.5	0.00
262.2072	30.1	0.00
262.2097	34.3	0.00
262.2101	0.9	0.00
262.2126	2.3	0.00
263.2106	1.7	0.00
263.2130	1.0	0.00

Simulated: C18 H25 O1

m/z= 257.0094-263.3907

m/z	Intensity	Relative
257.1900	819594.8	100.00
258.1933	159561.3	19.47
258.1942	312.2	0.04
258.1963	2356.6	0.29
259.1942	1684.3	0.21
259.1967	14669.1	1.79
259.1976	60.8	0.01
259.1996	458.8	0.06
259.2025	3.3	0.00
260.1976	327.9	0.04
260.2001	846.2	0.10
260.2005	4.8	0.00
260.2009	5.6	0.00
260.2030	42.2	0.01
261.2009	30.1	0.00
261.2034	34.3	0.00
261.2039	0.9	0.00
261.2063	2.4	0.00
262.2043	1.7	0.00
262.2068	1.0	0.00

Simulated: C18 H23 D2 O1

m/z= 258.0157-264.3970

m/z	Intensity	Relative
259.2025	819783.3	100.00
260.2059	159598.0	19.47
260.2068	312.3	0.04
260.2088	2168.6	0.26
261.2068	1684.6	0.21
261.2093	14672.4	1.79
261.2101	60.8	0.01
261.2122	422.2	0.05
261.2151	2.7	0.00
262.2101	328.0	0.04
262.2126	846.4	0.10
262.2131	4.5	0.00
262.2135	5.6	0.00
262.2155	38.8	0.00
263.2135	30.2	0.00
263.2160	34.3	0.00
263.2164	0.9	0.00
263.2189	2.2	0.00
264.2169	1.7	0.00
264.2193	1.0	0.00

$C_{18}H_{23}DO$	$C_{18}H_{24}O$	$C_{18}H_{22}D_2O$

Detected value:

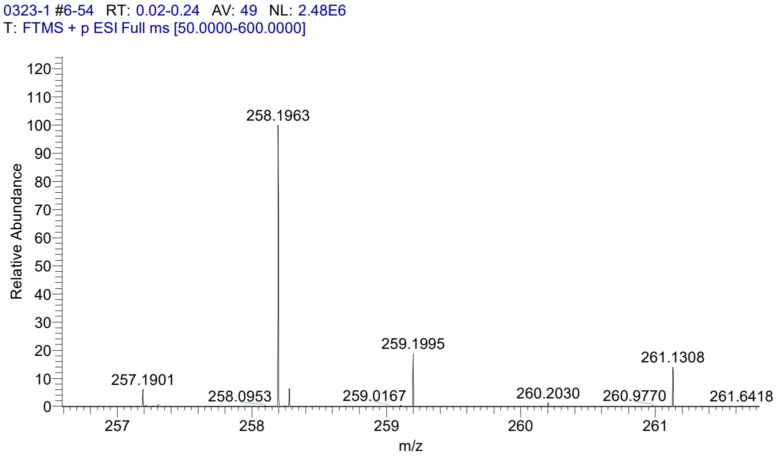

0323-1#6-54 RT: 0.02-0.24 AV: 49
T: FTMS + p ESI Full ms [50.0000-600.0000]
m/z= 256.5881-261.7739

m/z	Intensity	Relative
257.1534	2783.5	0.11
257.1841	4339.2	0.17
257.1901	155040.0	6.01
257.2123	12962.0	0.50
257.2667	6380.2	0.25
257.3032	15962.1	0.62
258.0953	6283.7	0.24
258.1963	2577564.0	100.00
258.2428	4883.9	0.19
258.2789	161161.4	6.25
259.0167	1481.6	0.06
259.1077	11880.5	0.46
259.1232	1580.4	0.06
259.1995	473128.1	18.36
260.0740	1646.2	0.06
260.2030	33934.0	1.32
260.9770	10364.1	0.40
261.1308	349305.8	13.55
261.1488	3698.2	0.14
261.6418	5599.2	0.22

Supplementary Figure 3. HR-MS (ESI) of **6a**-*d*

Deuterium ratio = (98.83%/(98.83%+6.01%)) = 94%

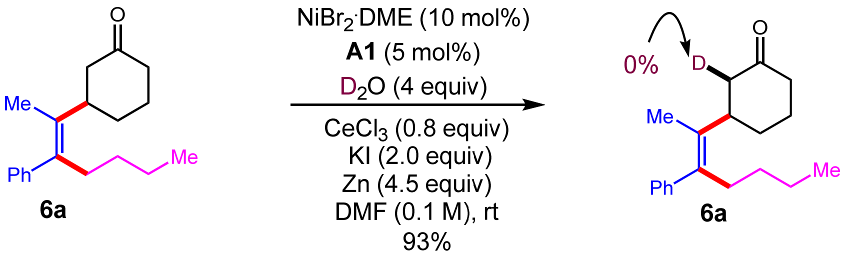

Following general procedure for Conditions A: The reaction was operated in a nitrogen-filled glove box. 4-Cyanopyridine (5 mol%, 0.57 mg), Zinc (4.5 equiv, 32.4 mg), $CeCl_3$ (0.8 equiv, 21.7 mg), KI (2.0 equiv, 36.5 mg) and $NiBr_2$·DME (10 mol%, 3.39 mg) were added to an oven-dried vial containing a magnetic stirring bar. **6a** (0.11 mmol, 30 mg) in anhydrous DMF (0.1 M, 1.1 mL) was added and rapid stirring was commenced. Then D_2O (4.0 equiv, 7.9 μL) was added via syringe. The reaction was stirred vigorously for 48 h at room temperature. The

reaction was diluted with ethyl acetate (30 mL) and washed with brine (50 mL), aqueous layer was extracted twice with ethyl acetate (20 mL). The combined organic layer was dried over magnesium sulfate, filtrated, evaporated and purified by flash chromatography with hexane : ethyl acetate mixtures as eluent to give 27.9 mg (93% yield) of **6a** as colourless oil. The deuterium ratio was assigned to be 0% by ^{1}H NMR.

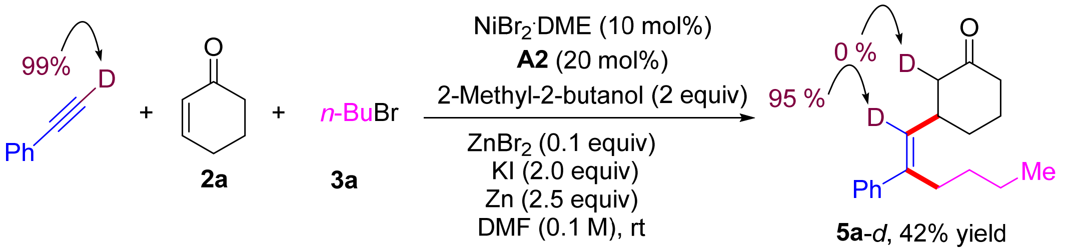

Following general procedure for Conditions B: The reaction was operated in a nitrogen-filled glove box. Zinc (2.5 equiv, 32.7 mg), $ZnBr_2$ (0.1 equiv, 4.5 mg), KI (2.0 equiv, 66.4 mg) and $NiBr_2$·DME (10 mol%, 6.17 mg) were added to an oven-dried vial containing a magnetic stirring bar. Anhydrous DMF (0.1 M, 2 mL) was added and rapid stirring was commenced. Then pentafluoropyridine (20 mol%, 4.4 μL), 2-methyl-2-butanol (2.0 equiv, 43.6 μL), phenylacetylene-D (2.0 equiv, 43.8 μL), **2a** (0.2 mmol, 19.5 μL) and **3a** (2.0 equiv, 45.6 μL) were added sequentially via syringe. The reaction was stirred vigorously for 24 h. The reaction was diluted with ethyl acetate (30 mL) and washed with brine (50 mL), aqueous layer was extracted twice with ethyl acetate (20 mL).The combined organic layer was dried over magnesium sulfate, evaporated and purified by flash chromatography with hexanes : ethyl acetate mixtures as eluent to give (21.6 mg, 42% yield) **5a**-*d* as colourless oil. ^{1}H NMR (400 MHz, $CDCl_3$) δ 7.36 – 7.27 (m, 4H), 7.26 – 7.20 (m, 1H), 2.92 – 2.81 (m, 1H), 2.51 – 2.37 (m, 4H), 2.36 – 2.27 (m, 1H), 2.27 – 2.18 (m, 1H), 2.17 – 2.07 (m, 1H), 1.95 – 1.87 (m, 1H), 1.83 – 1.70 (m, 1H), 1.60 – 1.51 (m, 1H), 1.34 – 1.23 (m, 4H), 0.89 – 0.81 (m, 3H); ^{13}C NMR (101 MHz, $CDCl_3$) δ 211.22, 142.79, 140.47, 131.52, 131.38, 131.00 (t, J = 23.23 Hz), 128.30, 126.94, 126.54, 48.21, 41.32, 38.64, 32.05, 31.17, 29.80, 25.45, 22.74, 14.01; HR-MS (ESI) m/z calcd for $C_{18}H_{24}DO[M + H^+]$: 258.1963, found: 258.1960.

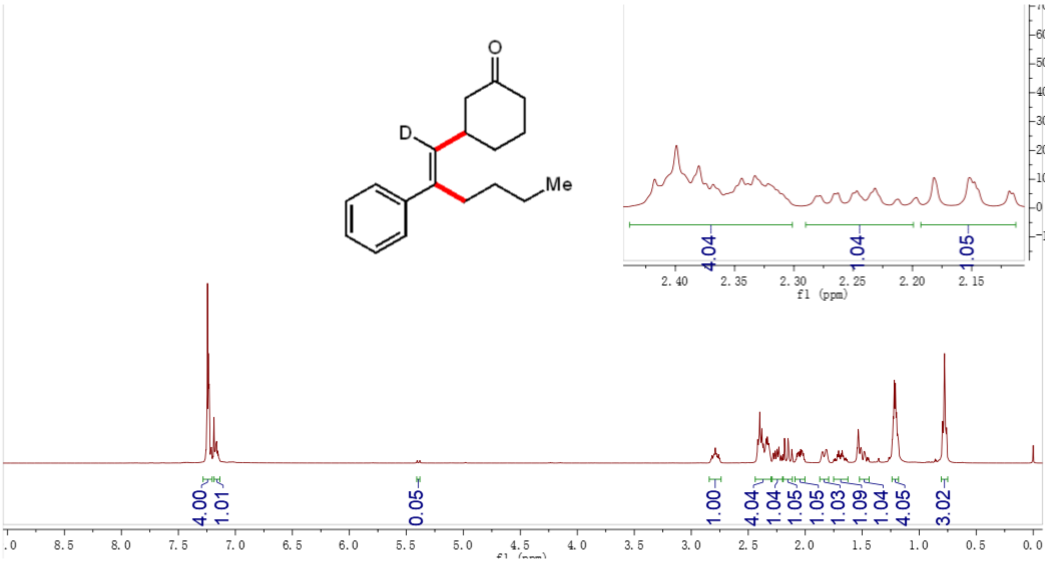

Supplementary Figure 4. ^{1}H NMR (400 MHz, $CDCl_3$) of **5a**-*d*

Radical clock experiments

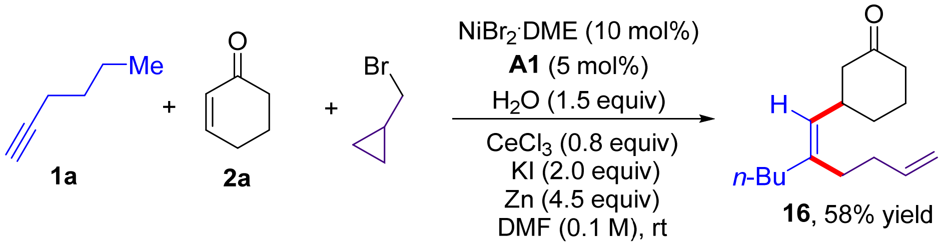

Following general procedure for Conditions A: The reaction was operated in a nitrogen-filled glove box. 4-Cyanopyridine (5 mol%, 1.04 mg), zinc (4.5 equiv, 58.2 mg), $CeCl_3$ (0.8 equiv, 39.4 mg), KI (2.0 equiv, 66.4 mg) and $NiBr_2$·DME (10 mol%, 6.17 mg) were added to an oven-dried vial containing a magnetic stirring bar. Anhydrous DMF (0.1 M, 2 mL) was added and rapid stirring was commenced. Then H_2O (1.5 equiv, 5.4 μL), **1a** (2.0 equiv, 45.9 μL), **2a** (0.2 mmol, 19.5 μL) and (bromomethyl)cyclopropane (2.0 equiv, 38.8 μL) were added sequentially via syringe. The reaction was stirred vigorously for 48 h. The reaction was diluted with ethyl acetate (30 mL) and washed with brine (50 mL), aqueous layer was extracted twice with ethyl acetate (20 mL). The combined organic layer was dried over magnesium sulfate, evaporated and purified by silica gel chromatography with hexane:ethyl acetate mixtures as eluent to give 27.2 mg (58% yield) **16** as colourless oil. ^{1}H NMR (400 MHz, $CDCl_3$) δ 5.86 – 5.72 (m, 1H), 5.06 – 4.90 (m, 3H), 2.74 – 2.61 (m, 1H), 2.41 – 2.22 (m, 3H), 2.12 – 2.01 (m, 5H), 2.00 – 1.92 (m, 2H), 1.83 – 1.65 (m, 2H), 1.51 – 1.42 (m, 1H), 1.41 – 1.23 (m, 5H), 0.89 (t, J = 7.1 Hz, 3H); ^{13}C NMR (101 MHz, $CDCl_3$) δ 211.66, 139.00, 138.41, 128.38, 114.74, 48.55, 41.31, 38.00, 36.32, 32.86, 32.26, 30.34, 29.75, 25.40, 22.48, 14.09; HR-MS (ESI) m/z calcd for $C_{16}H_{27}O$[M + H^+]: 235.2056, found: 235.2053.

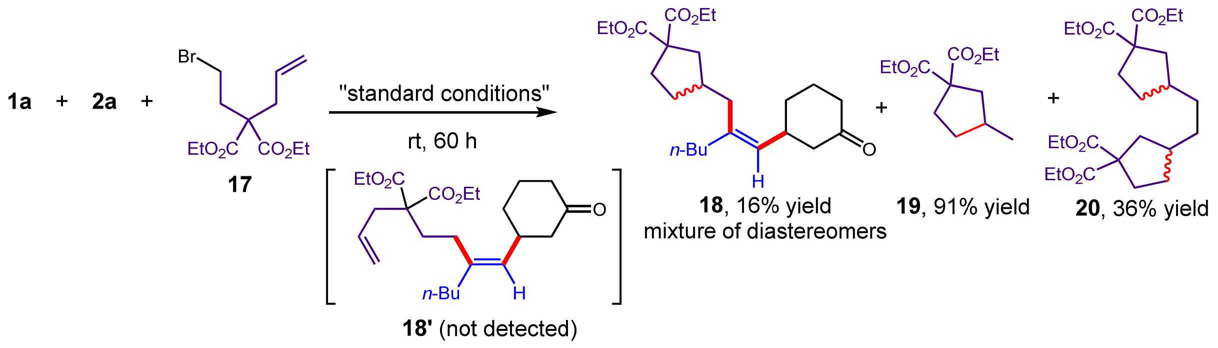

Following general procedure for Conditions A: The reaction was operated in a nitrogen-filled glove box. 4-Cyanopyridine (5 mol%, 1.04 mg), zinc (4.5 equiv, 58.2 mg), $CeCl_3$ (0.8 equiv, 39.4 mg), KI (2.0 equiv, 66.4 mg) and $NiBr_2$·DME (10 mol%, 6.17 mg) were added to an oven-dried vial containing a magnetic stirring bar. Anhydrous DMF (0.1 M, 2mL) was added and rapid stirring was commenced. Then H_2O (1.5 equiv, 5.4 μL), **1a** (2.0 equiv, 45.9 μL), **2a** (0.2 mmol, 19.5 μL) and **17** (2.0 equiv, 0.12 g) were added sequentially via syringe. The reaction was stirred vigorously for 60 h. The reaction was diluted with ethyl acetate (30 mL) and washed with brine (50 mL), aqueous layer was extracted twice with ethyl acetate (20 mL).The combined organic layer was dried over magnesium sulfate, evaporated and purified by flash chromatography with hexanes : ethyl acetate mixtures as eluent to give 26.0 mg (16% yield) **18** as colourless oil. ^{1}H NMR (600 MHz, $CDCl_3$) δ 5.01 (d, J = 9.4 Hz, 1H), 4.22 – 4.11 (m, 4H), 2.76 – 2.66 (m, 1H), 2.45 – 2.22 (m, 5H), 2.17 – 2.00 (m, 6H), 1.94 (t, J = 7.5 Hz, 2H), 1.84 – 1.74 (m, 2H), 1.73 – 1.64 (m, 2H), 1.48 – 1.40 (m, 1H), 1.37 – 1.30 (m, 2H), 1.29 – 1.20 (m, 9H), 0.88 (t, J = 7.2 Hz, 3H); ^{13}C NMR (151 MHz, $CDCl_3$) δ 211.55, 211.43, 172.80, 172.74, 138.34, 128.83, 128.80, 61.46, 61.38, 60.01, 59.95, 48.47, 48.44, 41.29, 40.77, 40.69, 38.51, 38.36, 37.91, 37.87, 36.29, 36.26, 35.17, 35.10, 33.83, 32.29, 32.27, 32.20, 32.18, 30.42, 25.30, 22.43, 14.13, 14.12, 14.08; HR-MS (ESI) m/z calcd for $C_{24}H_{39}O_5$[M + H$^+$]: 407.2792, found: 407.2788. **19** (41.5 mg, 91% yield, based on **2a**) was isolated as a colourless oil. ^{1}H NMR (400 MHz, $CDCl_3$) δ 4.16 (q, J = 7.1 Hz, 4H), 2.44 (dd, J = 13.2, 7.1Hz, 1H), 2.35 - 2.27 (m, 1H), 2.18 - 2.07 (m, 1H), 2.06 - 1.98 (m, 1H), 1.90 - 1.76 (m,1H), 1.64 (dd, J = 13.4, 10.0 Hz, 2H), 1.22 (t, J = 7.1 Hz, 6H), 1.00 (d, J = 6.7 Hz, 3H). The data is consisted with reported datd from literature[1]. **20** (32.7 mg, 36% yield, based on **2a**) was isolated as a colourless oil. ^{1}H NMR (400 MHz, $CDCl_3$) δ 4.16 (q, J = 7.1 Hz, 8H), 2.43 - 2.39 (m, 2H), 2.27 - 2.12 (m, 2H), 2.11 - 2.09 (m, 2H), 1.94 – 1.85 (m, 4H), 1.67 – 1.65 (m, 2H), 1.35 – 1.32 (m, 4H), 1.26 – 1.20 (m, 14H). The data is consisted with literature[2].

Radical capture experiments

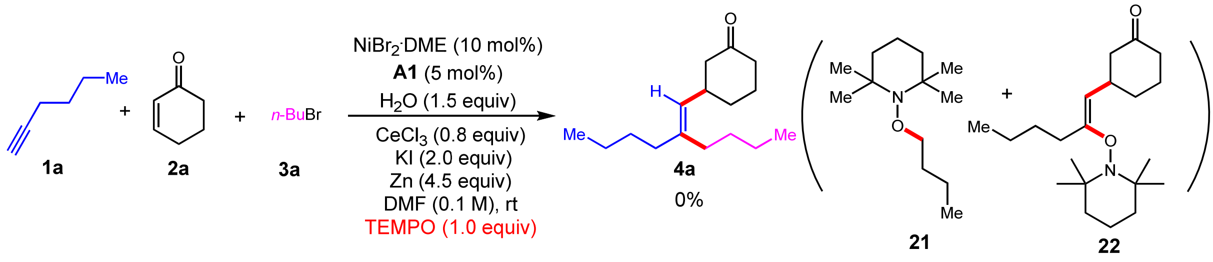

Following general procedure for Conditions A: The reaction was operated in a nitrogen-filled glove box. 4-Cyanopyridine (5 mol%, 1.04 mg), zinc (4.5 equiv, 58.2 mg), $CeCl_3$ (0.8 equiv, 39.4 mg), KI (2.0 equiv, 66.4 mg), TEMPO (1.0 equiv, 31.2 mg) and $NiBr_2$·DME (10 mol%, 6.17 mg) were added to an oven-dried vial containing a magnetic stirring bar. Anhydrous DMF (0.1 M, 2 mL) was added and rapid stirring was commenced. Then H_2O (1.5 equiv, 5.4 µL), **1a** (2.0 equiv, 45.9 µL), **2a** (0.2 mmol, 19.5 µL) and **3a** (2.0 equiv, 45.6 µL) were added sequentially via syringe. The reaction was stirred vigorously for 48 h. The reaction was detected by GC and no desired product was found. Two intermediates were detected by HR-MS (ESI). For **21** HR-MS (ESI) m/z calcd for $C_{13}H_{28}NO[M + H^+]$: 214.2165, found: 214.2162. For **22** HR-MS (ESI) m/z calcd for $C_{21}H_{38}NO_2[M + H^+]$: 336.2897, found: 336.2893.

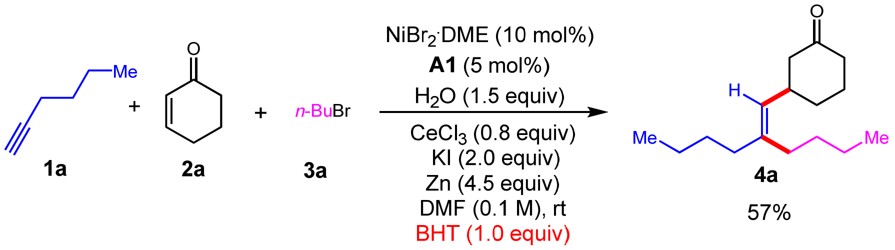

Following general procedure for Conditions A: The reaction was operated in a nitrogen-filled glove box. 4-Cyanopyridine (5 mol%, 1.04 mg), zinc (4.5 equiv, 58.2 mg), $CeCl_3$ (0.8 equiv, 39.4 mg), KI (2.0 equiv, 66.4 mg), BHT (1.0 equiv, 44.1 mg) and $NiBr_2$·DME (10 mol%, 6.17 mg) were added to an oven-dried vial containing a magnetic stirring bar. Anhydrous DMF (0.1 M, 2 mL) was added and rapid stirring was commenced. Then H_2O (1.5 equiv, 5.4 µL), **1a** (2.0 equiv, 45.9 µL), **2a** (0.2 mmol, 19.5 µL) and **3a** (2.0 equiv, 45.6 µL) were added sequentially via syringe. The reaction was stirred vigorously for 48 h. The reaction was diluted with ethyl acetate (30 mL) and washed with brine (50 mL), aqueous layer was extracted twice with ethyl acetate (20 mL).The combined organic layer was dried over magnesium sulfate, evaporated and purified by flash chromatography with hexanes : ethyl acetate mixtures as eluent to give **4a** (26.9 mg, 57% yield) as colourless oil.

Detecting organozinc intermediate

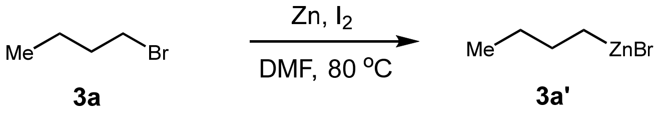

Preparation of organozinc reagents **3a'** was following Morken's procedure[3] with slight modification with DMF as solvent: A 20 mL vial was charged with zinc powder (1.26 g, 19.31 mmol, 2.50 equiv) and a stir bar. The vial was capped with a PTFE-lined pierceable screw cap and the system was heated at 80 °C under high vacuum for 2 hours with stirring. The vial was then cooled to room temperature and backfilled with N_2. At this point the vial was brought into an Ar filled glove box, a solution of iodine (95.84 mg, 0.38 mmol, 0.02 equiv) in DMF (1 mL) and the suspension was stirred until the red color subsided. **3a** (0.82 mL, 7.60 mmol, 1.0 equiv) and an additional 4 mL of DMF were added sequentially. The vial was capped with a Teflon screwcap, taped and the suspension was heated at 80 °C for 12 hours. Next, the mixture was cooled to room temperature, brought inside the glovebox and filtered through a syringe filter. ^{1}H NMR (400 MHz, $CDCl_3$) δ 1.51 – 1.43 (m, 2H), 1.30 – 1.20 (m, 2H), 0.80 (t, J = 7.1, 3H), 0.24 – 0.16 (t, J = 7.9, 2H); ^{13}C NMR (101 MHz, $CDCl_3$) δ 31.68, 30.77, 28.91, 14.03.

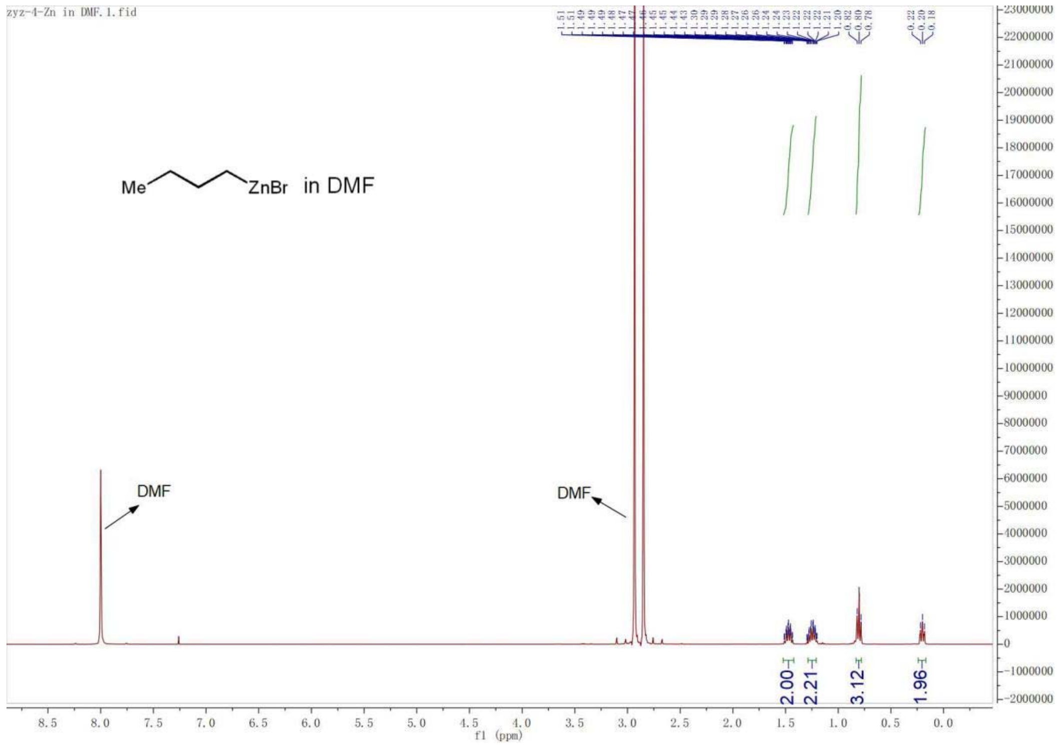

Supplementary Figure 5. ^{1}H NMR (400 MHz, $CDCl_3$) of **3a'**

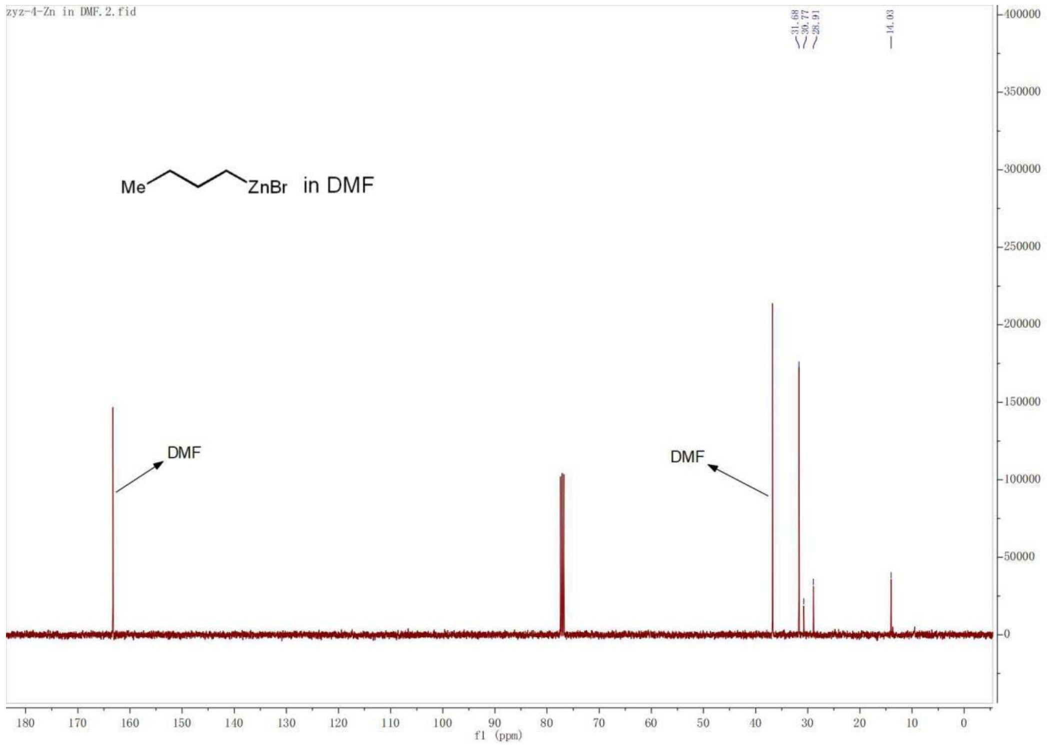

Supplementary Figure 6. ^{13}C NMR (101 MHz, $CDCl_3$) of **3a'**

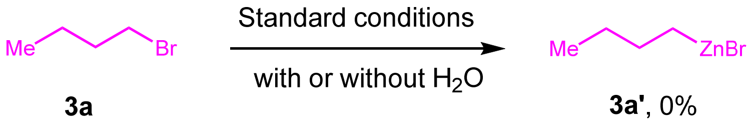

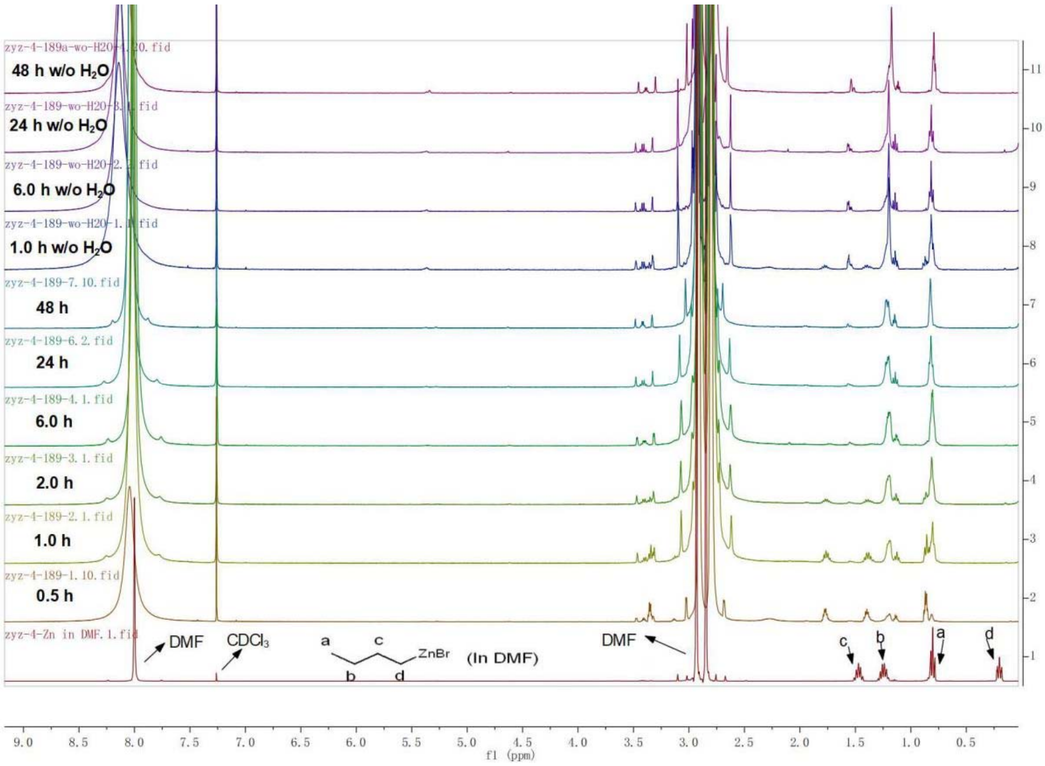

Supplementary Figure 7. Detecting of **3a'** by ^{1}H NMR (400 MHz, $CDCl_3$) (**3a** under standard conditions)

Following general procedure for Conditions A: The reaction was operated in a nitrogen-filled glove box. 4-Cyanopyridine (5 mol%, 1.04 mg), zinc (4.5 equiv, 58.2 mg), $CeCl_3$ (0.8 equiv, 39.4 mg), KI (2.0 equiv, 66.4 mg) and $NiBr_2 \cdot DME$ (10 mol%, 6.2 mg) were added to an oven-dried vial containing a magnetic stirring bar. Anhydrous DMF (0.1 M, 1.4 mL) was added and rapid stirring was commenced. Then H_2O (1.5 equiv, 5.4 μL), **3a** (2.0 equiv, 45.6 μL) was added sequentially via syringe. 60 μL of the reaction mixture was taken out at 0.5 h, 1.0 h, 2.0 h, 6.0 h, 24 h, 48 h (in the case of without H_2O, the sample was taken out at 1.0 h, 6.0 h, 24 h, 48 h) and filtered through a syringe filter using $CDCl_3$ as solvent, the sample was then analyzed by ^1H NMR of the crude mixture.

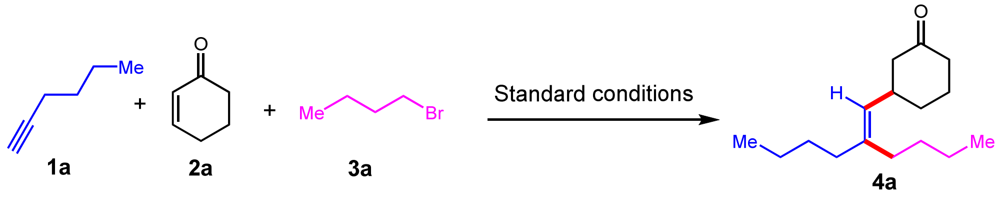

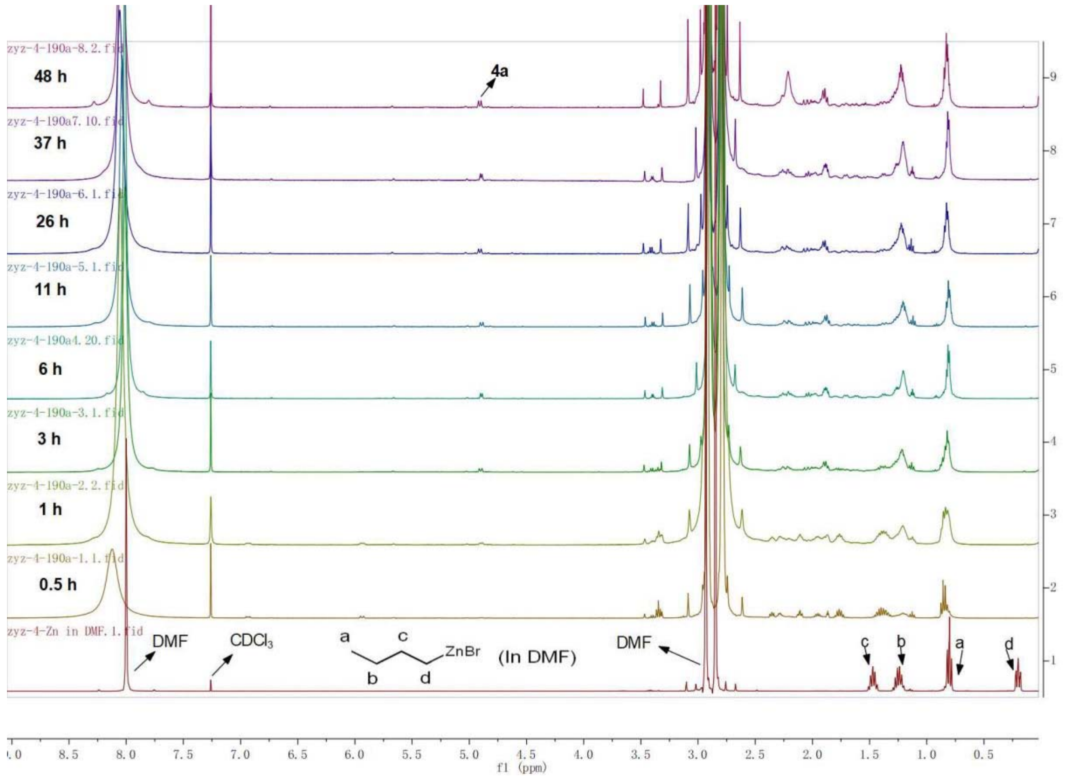

Supplementary Figure 8. Detecting of **3a'** by ^1H NMR (400 MHz, $CDCl_3$) (under standard conditions)

Following general procedure for Conditions A: The reaction was operated in a nitrogen-filled glove box. 4-Cyanopyridine (5 mol%, 1.04 mg), zinc (4.5 equiv, 58.2 mg), $CeCl_3$ (0.8 equiv, 39.4 mg), KI (2.0 equiv, 66.4 mg) and $NiBr_2 \cdot DME$ (10 mol%, 6.2 mg) were added to an oven-dried vial containing a magnetic stirring bar. Anhydrous DMF (0.1 M, 2 mL) was added and

rapid stirring was commenced. Then H_2O (1.5 equiv, 5.4 μL), **1a** (2.0 equiv, 45.9 μL), **2a** (0.2 mmol, 19.5 μL) and **3a** (2.0 equiv, 45.6 μL) were added sequentially via syringe. 60 μL of the reaction mixture was taken out at 0.5 h, 1.0 h, 3.0 h, 6.0 h, 11 h, 26 h, 37 h, 48 h and filtered through a syringe filter using $CDCl_3$ as solvent, the sample was then analyzed by ^{1}H NMR of the crude mixture.

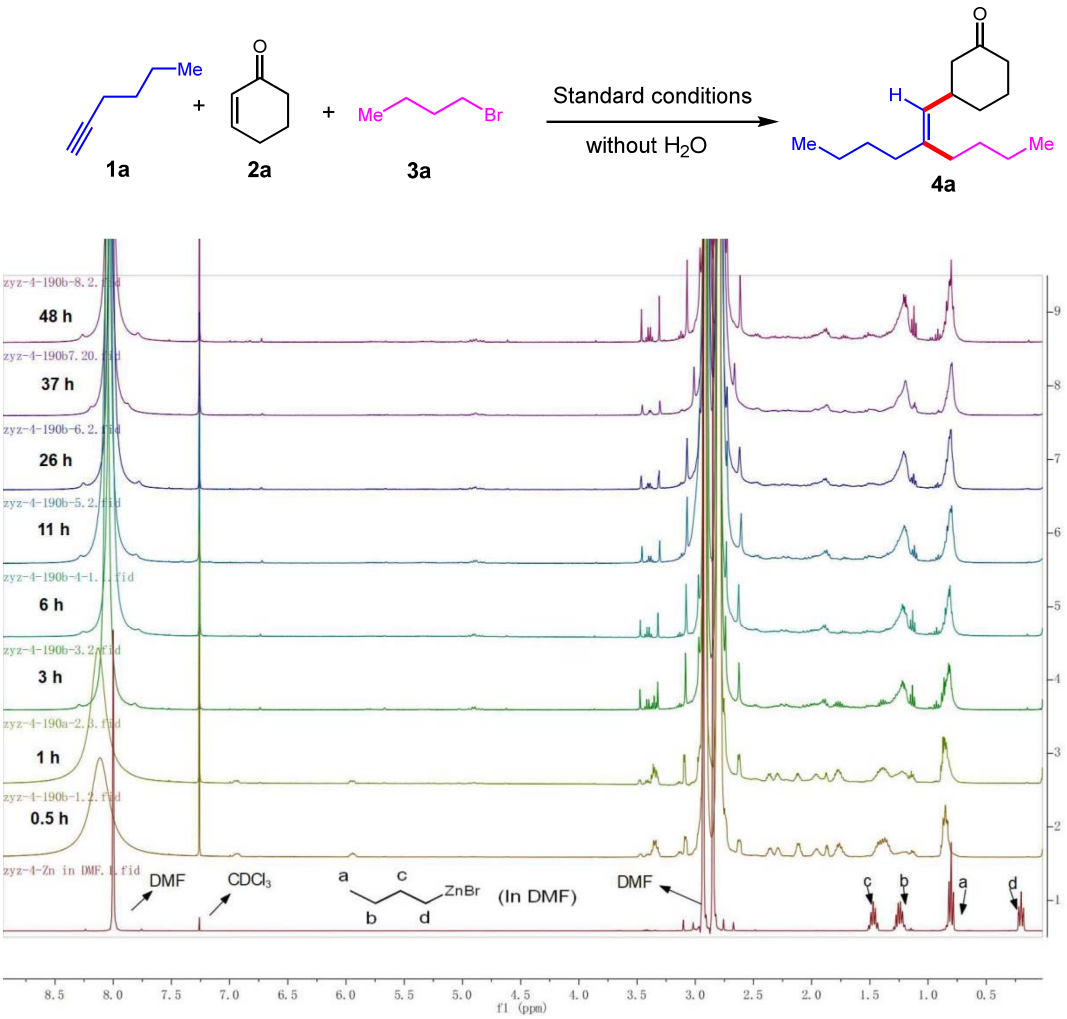

Supplementary Figure 9. Detecting of **3a'** by ^{1}H NMR (400 MHz, $CDCl_3$) (under standard conditions without H_2O)

Following general procedure for Conditions A: The reaction was operated in a nitrogen-filled glove box. 4-Cyanopyridine (5 mol%, 1.04 mg), zinc (4.5 equiv, 58.2 mg), $CeCl_3$ (0.8 equiv, 39.4 mg), KI (2.0 equiv, 66.4 mg) and $NiBr_2 \cdot DME$ (10 mol%, 6.17 mg) were added to an oven-dried vial containing a magnetic stirring bar. Anhydrous DMF (0.1 M, 2 mL) was added and rapid stirring was commenced. Then **1a** (2.0 equiv, 45.9 μL), **2a** (0.2 mmol, 19.5 μL) and **3a** (2.0 equiv, 45.6 μL) were added sequentially via syringe. 60 μL of the reaction mixture was taken out at 0.5 h, 1.0 h, 3.0 h, 6.0 h, 11 h, 26 h, 37 h, 48 h and filtered through a syringe filter using $CDCl_3$ as solvent, the sample was then analyzed by ^{1}H NMR of the crude mixture.

Control experiments

Supplementary Table 1. Condition evaluation: The amount of additives[a]

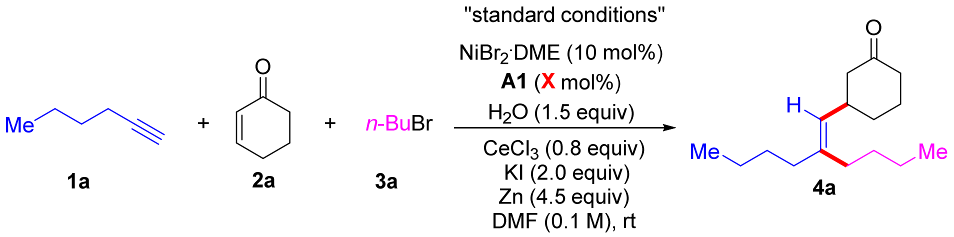

entry	the amount of 4-cyanopyridine (X mol%)	yield of **4a**[b]
1	w/o **A1**	37%
2	5 mol% **A1**	66%
3	10 mol% **A1**	62%
4	20 mol% **A1**	59%
5	40 mol% **A1**	55%
6	80 mol% **A1**	52%
7	100 mol% **A1**	50%
8	150 mol% **A1**	47%
9	200 mol% **A1**	44%

[a]Reaction was run using 0.4 mmol of **1a**, 0.2 mmol of **2a**, and 0.4 mmol of **3a** under standard conditions for 48 h. [b]Yield was determined by GC analysis using *n*-dodecane as internal standard. **A1** = 4-cyanopyridine.

Supplementary Table 2. Condition evaluation: The amount of H_2O[a]

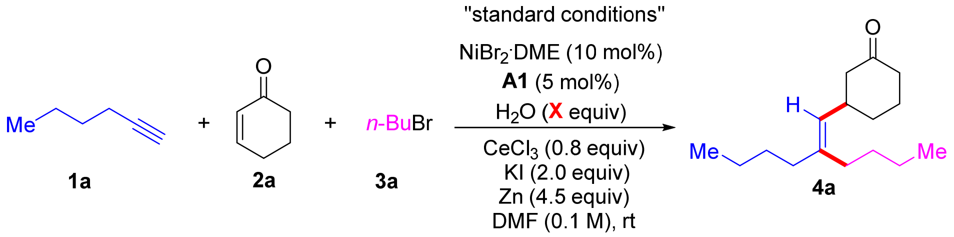

entry	the amount of H_2O (X equiv)	yield of **4a**[b]
1	w/o H_2O	17%
2	0.5 equiv H_2O	46%
3	1.0 equiv H_2O	61%
4	1.5 equiv H_2O	66%
5	2.0 equiv H_2O	51%
6	2.5 equiv H_2O	50%
7	3.0 equiv H_2O	32%
8	6.0 equiv H_2O	10%

[a]Reaction was run using 0.4 mmol of **1a**, 0.2 mmol of **2a**, and 0.4 mmol of **3a** under standard conditions for 48 h. [b]Yield was determined by GC analysis using *n*-dodecane as internal standard. **A1** = 4-cyanopyridine.

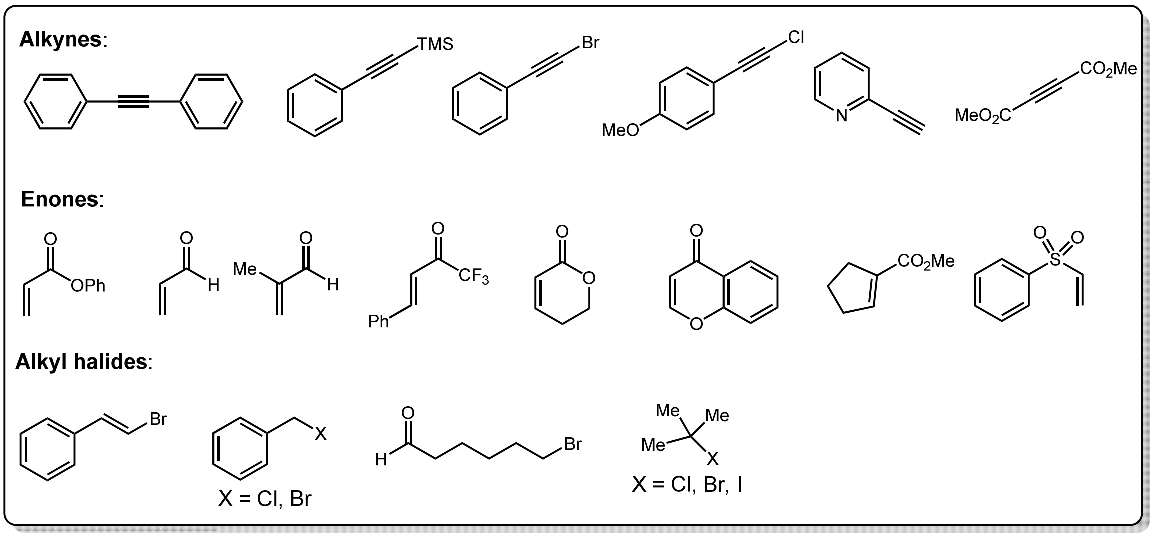

Supplementary Figure 10. Selected unsuccessful substrates

Identification of the mass balance and byproducts of the reaction

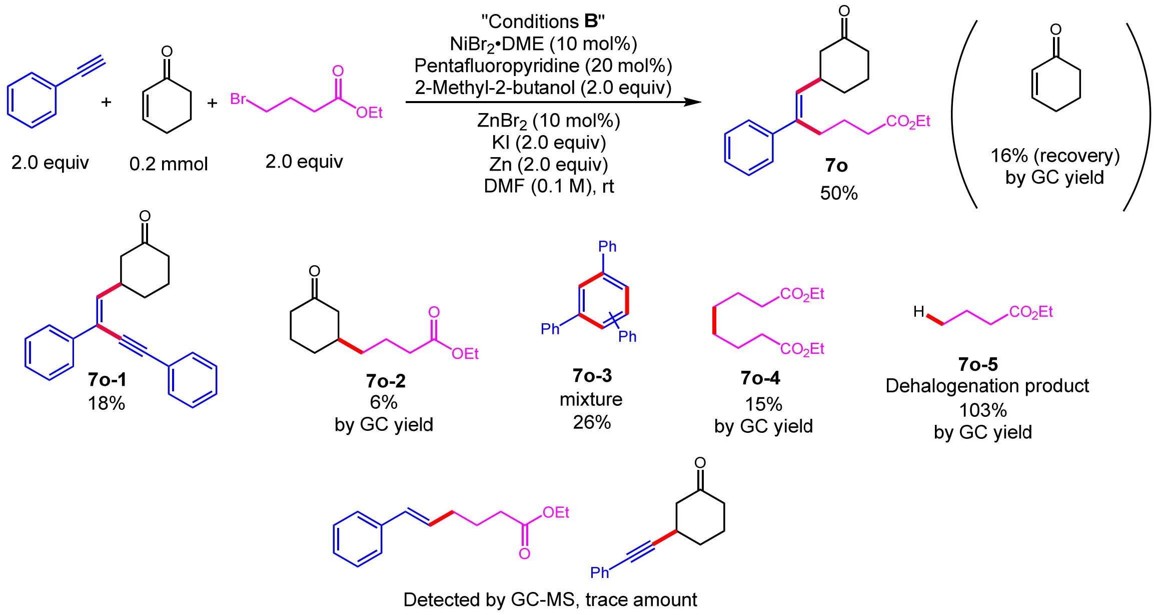

Following general procedure for Conditions B: The reaction was operated in a nitrogen-filled glove box. Zinc (2.5 equiv), $ZnBr_2$ (0.1 equiv), KI (2.0 equiv) and $NiBr_2$·DME (10 mol%) were added to an oven-dried vial containing a magnetic stirring bar. Anhydrous DMF (0.1 M, 2 mL) was added and rapid stirring was commenced. Then pentafluoropyridine (20 mol%, 4.39 μL), 2-methyl-2-butanol (2.0 equiv, 44.2 μL), Phenylacetylene (2.0 equiv, 43.9 μL), **2a** (0.2 mmol, 19.5 μL) and ethyl 4-bromobutyrate (2.0 equiv, 57.4 μL) were added sequentially via syringe. The reaction was stirred vigorously for 24 h. The reaction was diluted with ethyl acetate (30 mL) and washed with brine (50 mL), aqueous layer was extracted twice with ethyl acetate (20 mL).The combined organic layer was dried over magnesium sulfate, evaporated and purified by flash chromatography with hexanes : ethyl acetate mixtures as eluent to give the desired products (for **7o-2**, **7o-4**, **7o-5**, the yield was determined by GC analysis using *n*-dodecane as

internal standard).

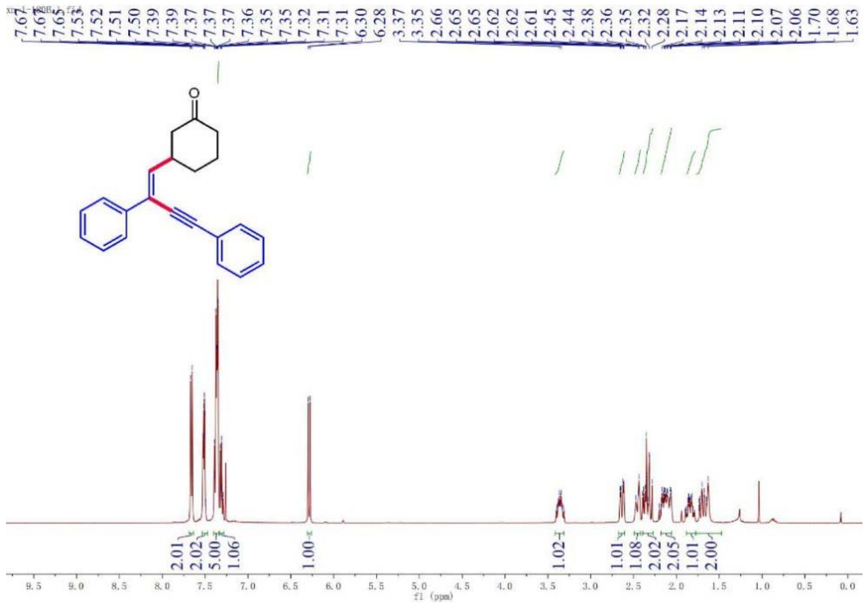

Supplementary Figure 11. ^{1}H NMR (400 MHz, $CDCl_3$) of **7o-1**

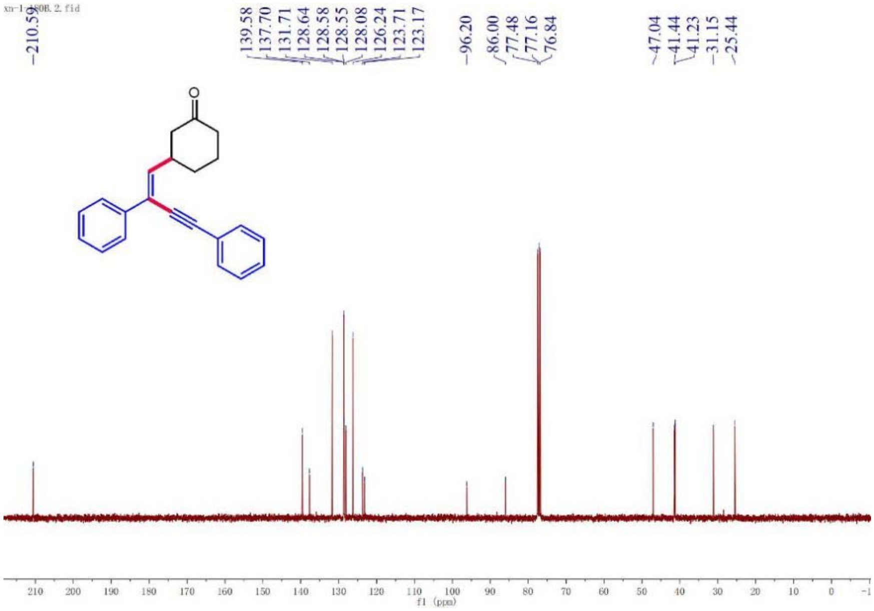

Supplementary Figure 12. ^{13}C NMR (101 MHz, $CDCl_3$) of **7o-1**

7o-1 was isolated as a light yellow oil (10.8 mg, 18% yield). ^{1}H NMR (400 MHz, $CDCl_3$): δ 7.69-7.64 (m, 2H), 7.53-7.49 (m, 2H), 7.40-7.34 (m, 5H), 7.32-7.29 (m, 1H), 6.29 (d, J = 9.1 Hz, 1H), 3.41-3.31 (m, 1H), 2.67-2.61 (m, 1H), 2.49-2.42 (m, 1H), 2.39-2.27 (m, 2H), 2.20-2.06 (m, 2H), 1.88-1.77 (m, 1H), 1.77-1.47 (m, 2H); ^{13}C NMR (101 MHz, $CDCl_3$): δ 210.59, 139.58, 137.70, 131.71, 128.64, 128.58, 128.55, 128.08, 126.24, 123.71, 123.17, 96.20, 86.00, 47.04, 41.44, 41.23, 31.15, 25.44; HR-MS (ESI) m/z calcd for $C_{22}H_{21}O[M+H]^{+}$: 301.1587, found: 301.1583.

The yield of **7o-2** was determined by GC analysis using *n*-dodecane as internal standard. ^{1}H NMR (600 MHz, $CDCl_3$) δ 4.12 (q, J = 7.1 Hz, 2H), 2.43 – 2.40 (m, 1H), 2.36 – 2.33 (m, 1H), 2.29 – 2.22 (m, 3H), 2.06 – 1.98 (m, 2H), 1.92 – 1.88 (m, 1H), 1.81 – 1.74 (m, 1H), 1.67 – 1.61 (m, 3H), 1.40 – 1.30 (m, 3H), 1.24 (t, J = 7.1 Hz, 3H). The data was in accordance with the literature[4].

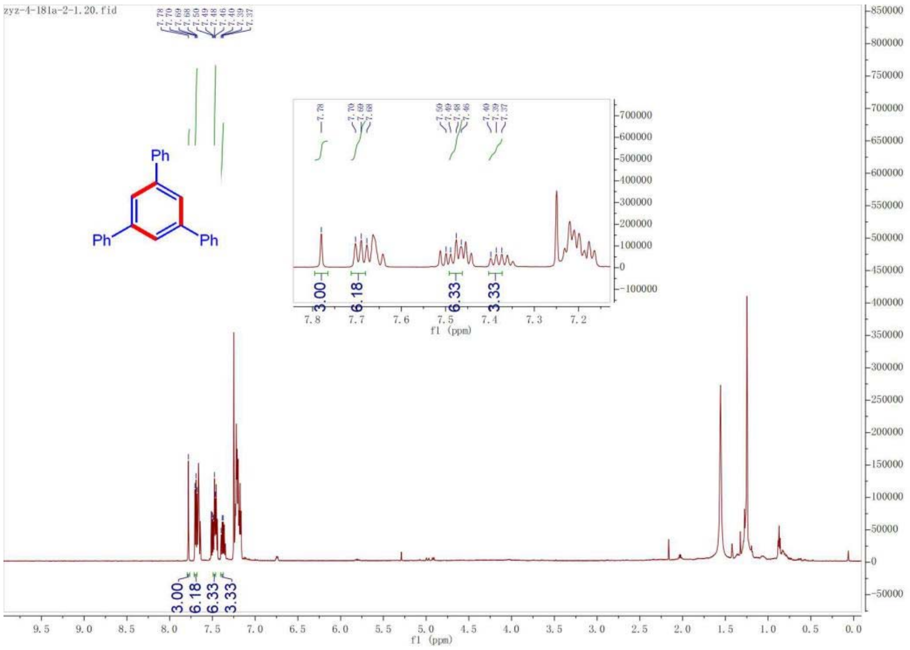

Supplementary Figure 13. ^{1}H NMR (600 MHz, $CDCl_3$) of **7o-3-a**

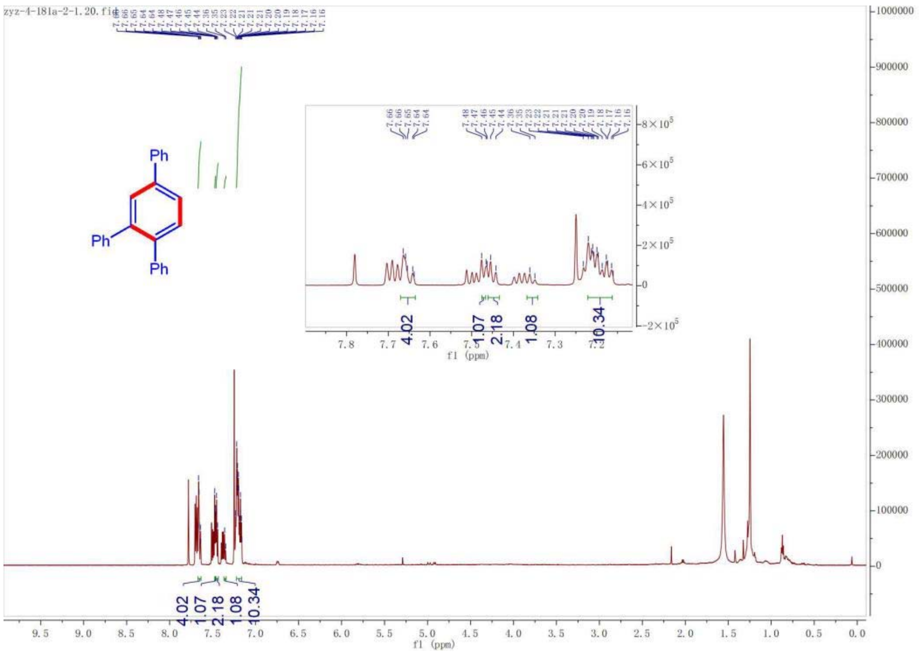

Supplementary Figure 14. ^{1}H NMR (600 MHz, $CDCl_3$) of **7o-3-b**

7o-3 was isolated as a colourless oil, (15.9 mg, 26% yield base on **2a**), the products were a mixture of two inseparable isomers. **7o-3-a**: ^{1}H NMR (600 MHz, $CDCl_3$) δ 7.78 (s, 3H), 7.72 – 7.67 (m, 6H), 7.49 – 7.45 (m, 6H), 7.40 – 7.37 (m, 3H); **7o-3-b**:^{1}H NMR (600 MHz, $CDCl_3$) δ

7.66 – 7.60 (m, 4H), 7.47 (d, J = 7.8 Hz, 1H), 7.42 – 7.39 (m, 2H), 7.33 – 7.30 (m, 1H), 7.20 – 7.15 (m, 10H). The data was in accordance with the literature[5,6].

The yield of **7o-4** was determined to be 15% by GC analysis using n-dodecane as internal standard. ^{1}H NMR (400 MHz, $CDCl_3$) δ 4.11 (q, J = 7.1 Hz, 4H), 2.27 (t, J = 7.5 Hz, 4H), 1.66 – 1.58 (m, 4H), 1.37 – 1.30 (m, 4H), 1.24 (t, J = 7.1 Hz, 6H). The data was in accordance with the literature[7].

The yield of **7o-5** was determined by GC analysis using n-dodecane as internal standard and compared with commercially available ethyl butyrate.

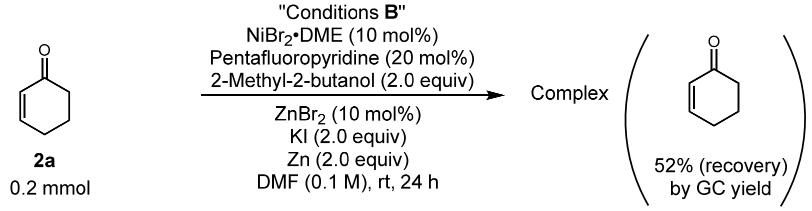

When **2a** was subjected to the reaction conditions, significant oligermization was observed and only 52% **2a** was recovered after 24 h.

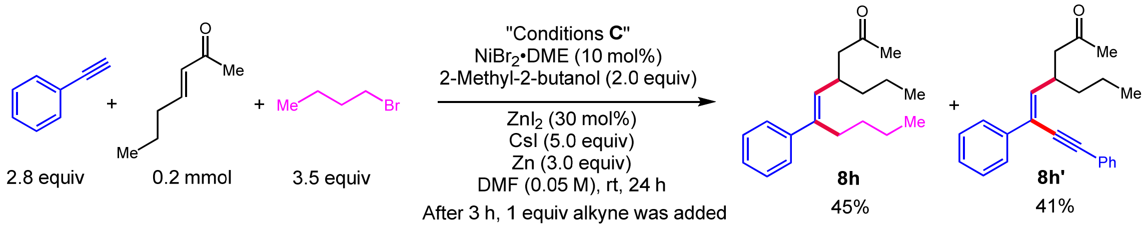

Following general procedure for Conditions C with slight modification: After 3 h, 1.0 equiv alkyne was added. The crude mixture was purified by flash chromatography on silica gel (eluted with ethyl acetate : hexanes = 1% ~2%) to give the product **8h** (24.5 mg, 45% yield) as a colorless oil and **8h'** (25.9 mg, 41% yield) as a colorless oil. ^{1}H NMR (400 MHz, $CDCl_3$) δ 7.67 – 7.65 (m, 2H), 7.55 – 7.52 (m, 2H), 7.39 – 7.34 (m, 5H), 7.32 – 7.27 (m, 1H), 6.24 (d, J = 9.8 Hz, 1H), 3.57 – 3.43 (m, 1H), 2.66 – 2.53 (m, 2H), 2.20 (s, 3H), 1.62 – 1.52 (m, 1H), 1.50 – 1.38 (m, 3H), 0.96 (t, J = 7.1 Hz, 3H); ^{13}C NMR (101 MHz, $CDCl_3$) δ 208.02, 141.11, 137.88, 131.60, 128.48, 128.46, 128.42, 127.83, 126.19, 124.16, 123.39, 95.43, 86.73, 49.65, 37.59, 37.51, 30.14, 20.67, 14.20; HR-MS (ESI) m/z calcd for $C_{23}H_{25}O[M + H^+]$: 317.1900, found: 317.1897.

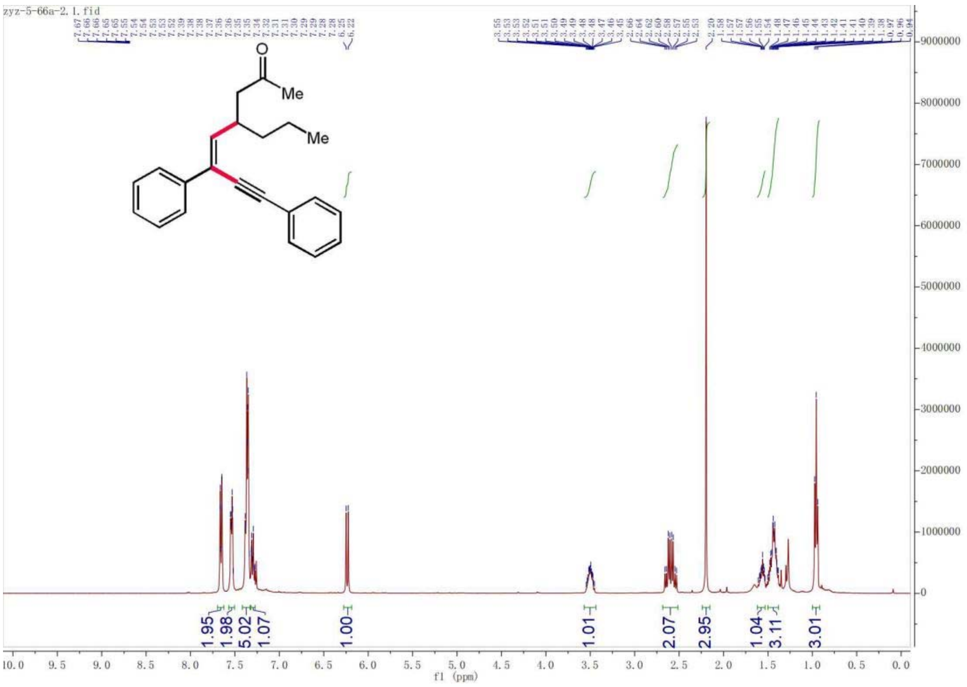

Supplementary Figure 15. ^{1}H NMR (400 MHz, $CDCl_3$) of **8h'**

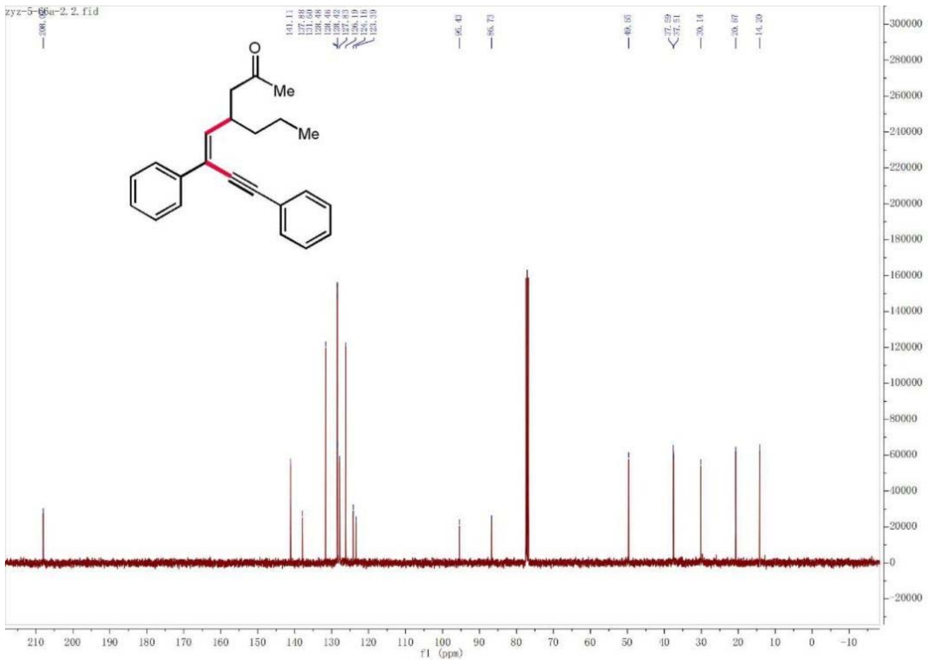

Supplementary Figure 16. ^{13}C NMR (101 MHz, $CDCl_3$) of **8h'**

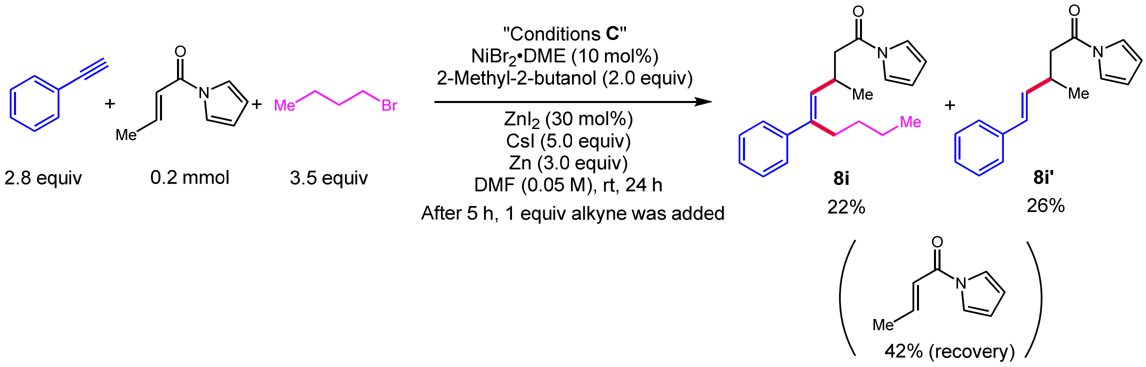

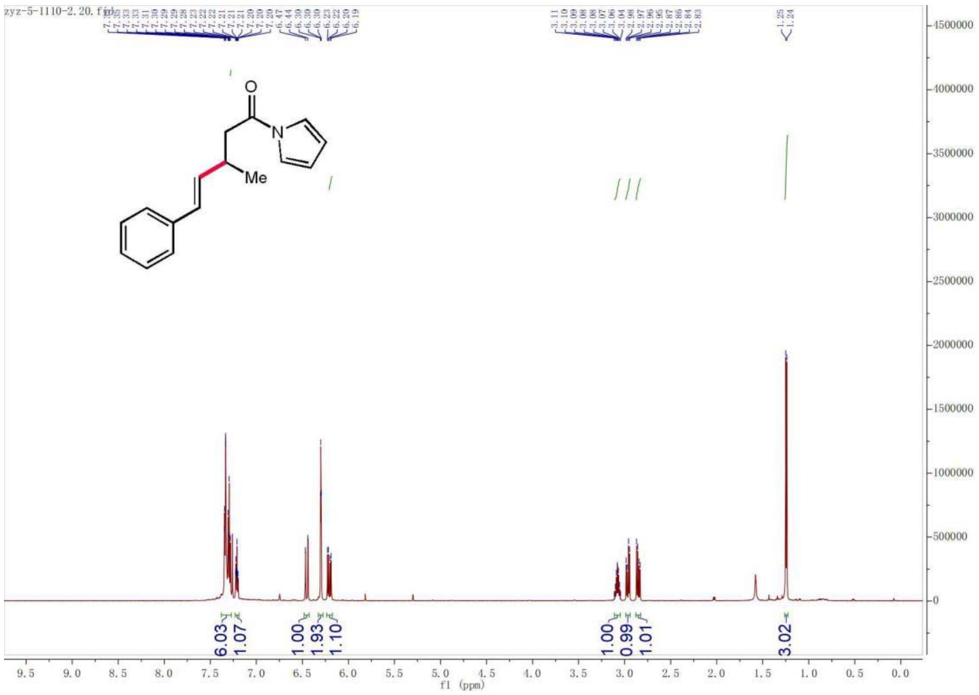

Supplementary Figure 17. ^{1}H NMR (600 MHz, $CDCl_3$) of **8i'**

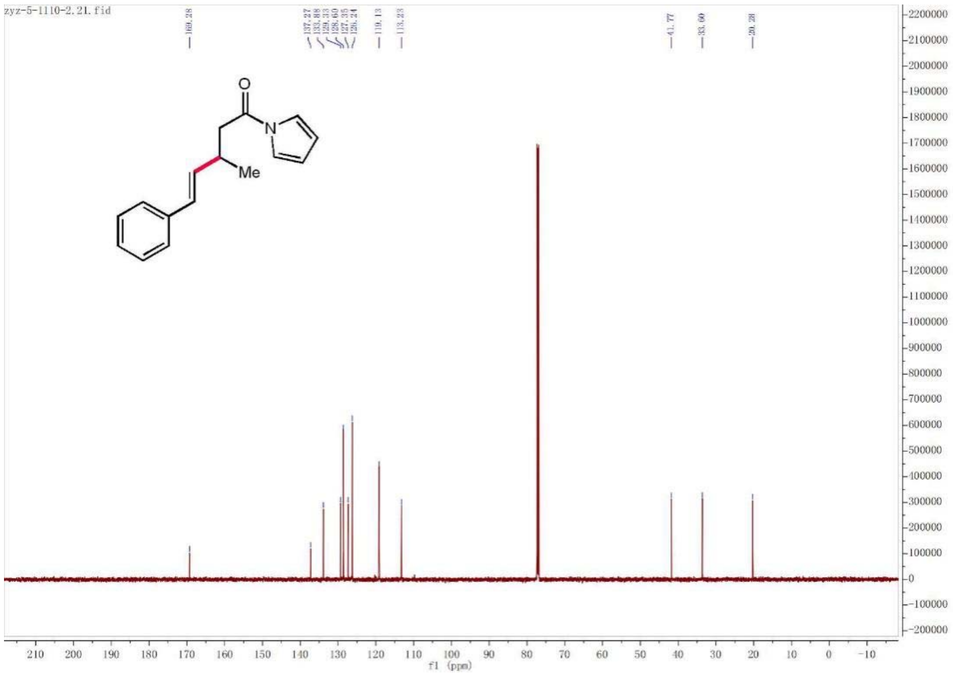

Supplementary Figure 18. ^{13}C NMR (151 MHz, $CDCl_3$) of **8i'**

Following general procedure for Conditions C with slight modification: After 5 h, 1.0 equiv alkyne was added. The crude mixture was purified by flash chromatography on silica gel (eluted with ethyl acetate : hexanes = 1% ~2%) to give the product **8i** (12.9 mg, 22% yield) as a colorless oil, together with **8i'** (12.4 mg, 26% yield) as a colorless oil and recovery of enonamide in 42%. ^{1}H NMR (600 MHz, $CDCl_3$) δ 7.38 – 7.27 (m, 6H), 7.23 – 7.19 (m, 1H), 6.45 (d, J = 15.9 Hz, 1H), 6.30 (t, J = 2.4 Hz, 2H), 6.21 (dd, J = 15.9, 7.4 Hz, 1H), 3.11 – 3.04 (m, 1H), 2.97 (dd, J = 15.8, 6.5 Hz, 1H), 2.85 (dd, J = 15.8, 7.4 Hz, 1H), 1.24 (d, J = 6.7 Hz,

3H); ^{13}C NMR (151 MHz, $CDCl_3$) δ 169.28, 137.27, 133.88, 129.33, 128.60, 127.35, 126.24, 119.13, 113.23, 41.77, 33.60, 20.28; HR-MS (ESI) m/z calcd for $C_{16}H_{18}NO[M + H^+]$: 240.1383, found: 240.1380.

2.4 ¹H, ¹³C, and ¹⁹F spectra of new compounds

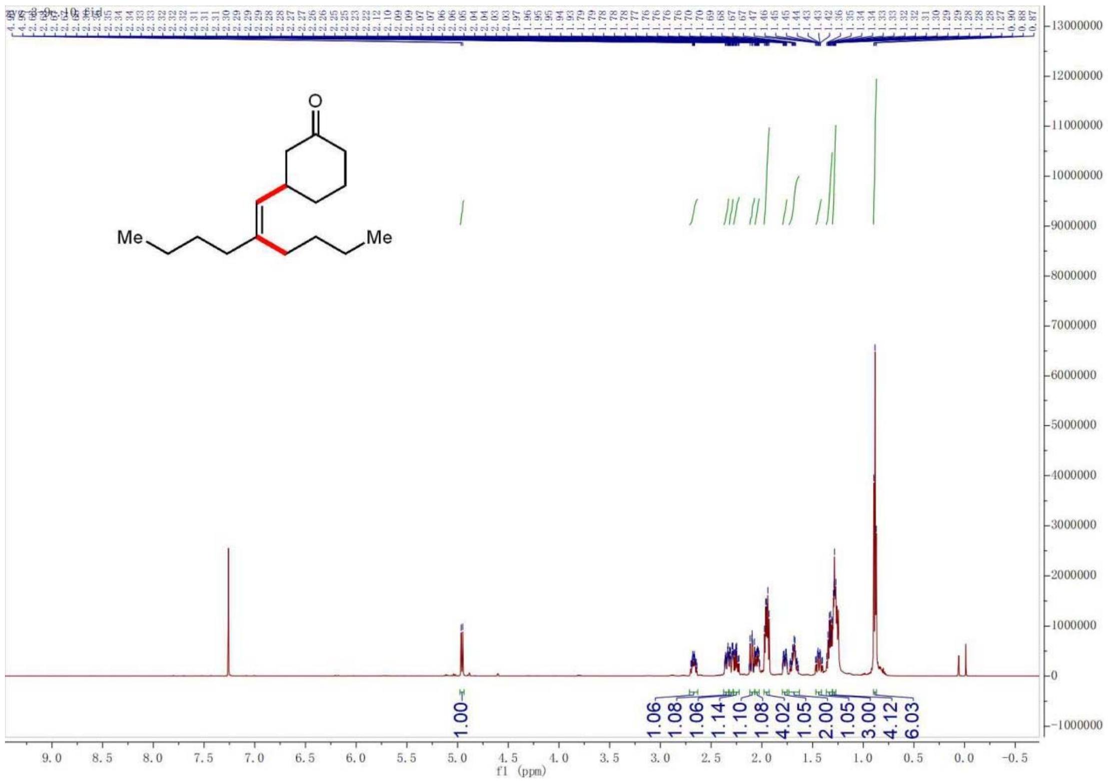

Supplementary Figure 19. ^{1}H NMR (600 MHz, $CDCl_3$) of **4a**

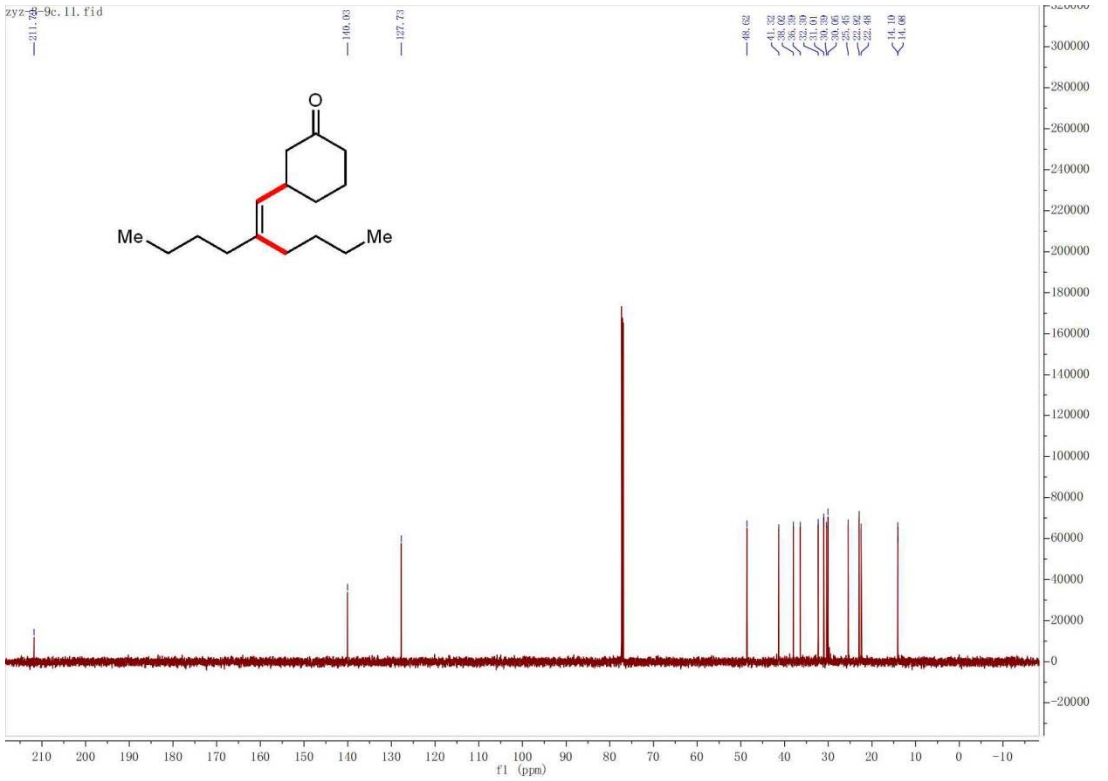

Supplementary Figure 20. ^{13}C NMR (151 MHz, $CDCl_3$) of **4a**

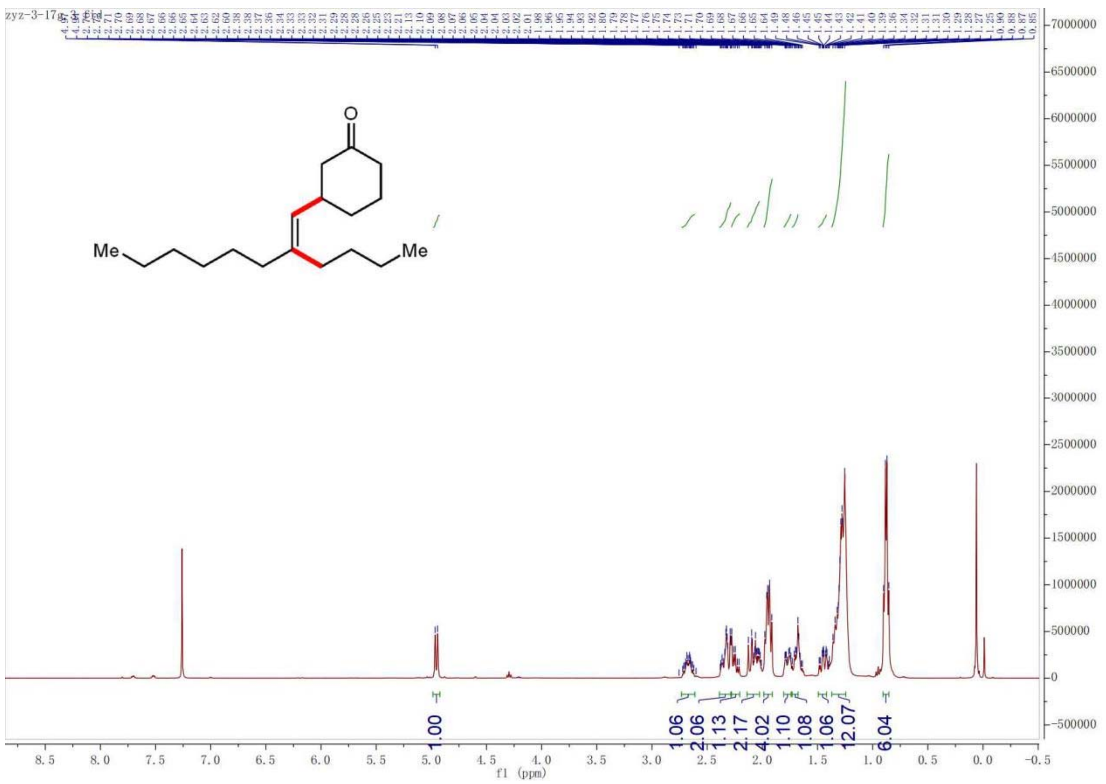

Supplementary Figure 21. ^{1}H NMR (400 MHz, $CDCl_3$) of **4b**

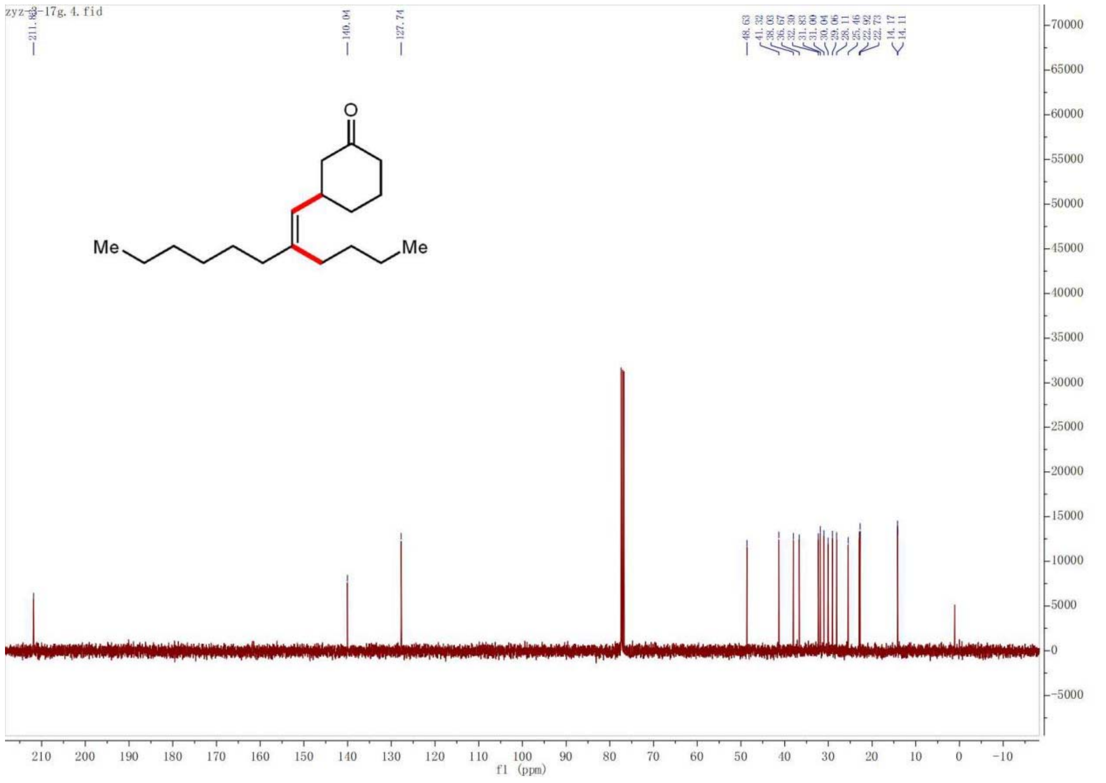

Supplementary Figure 22. ^{13}C NMR (101 MHz, $CDCl_3$) of **4b**

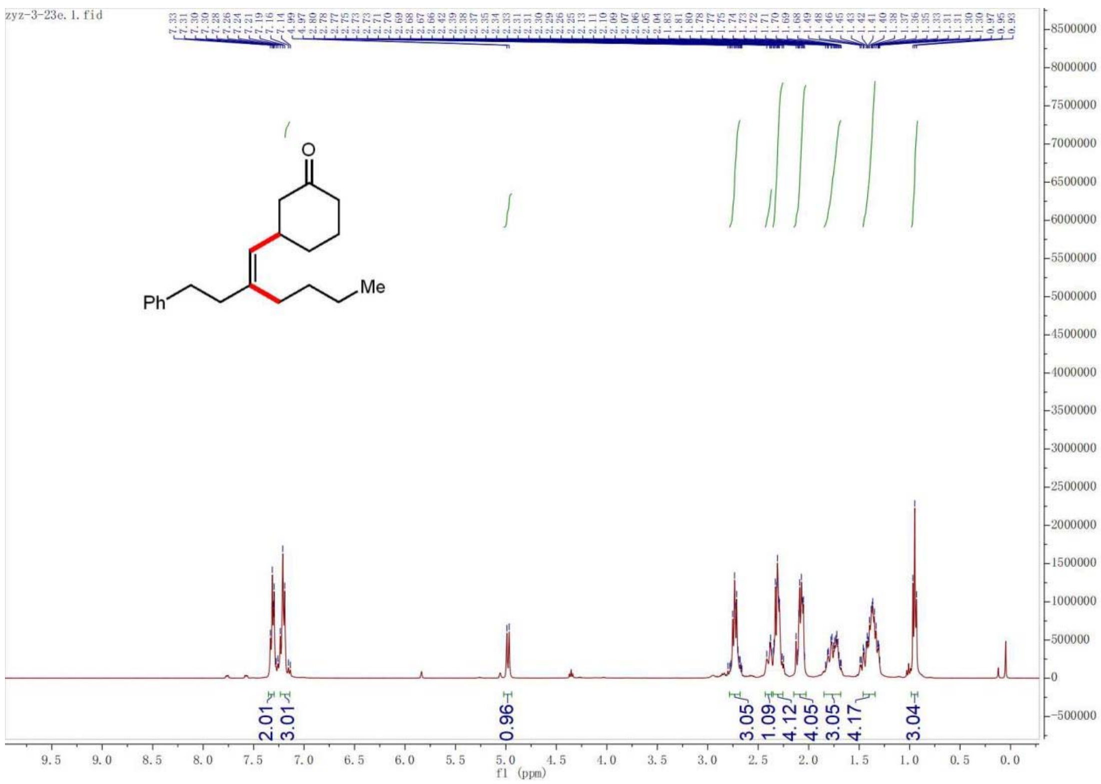

Supplementary Figure 23. ^{1}H NMR (400 MHz, $CDCl_3$) of **4c**

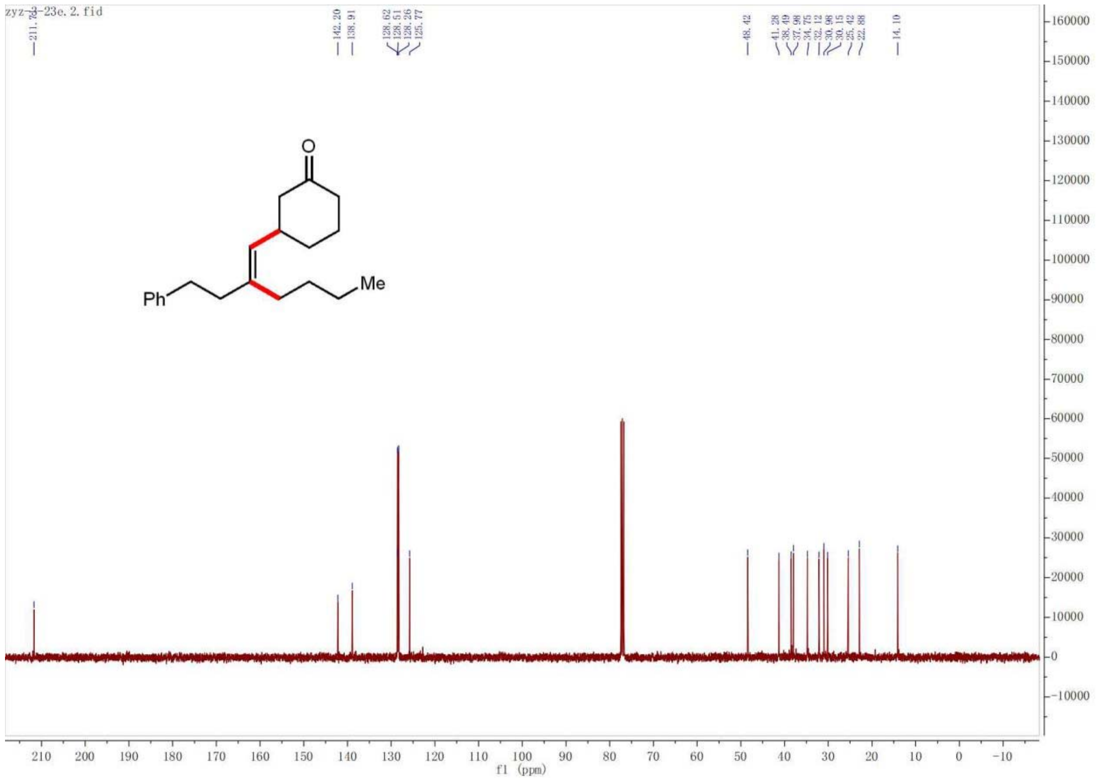

Supplementary Figure 24. ^{13}C NMR (101 MHz, $CDCl_3$) of **4c**

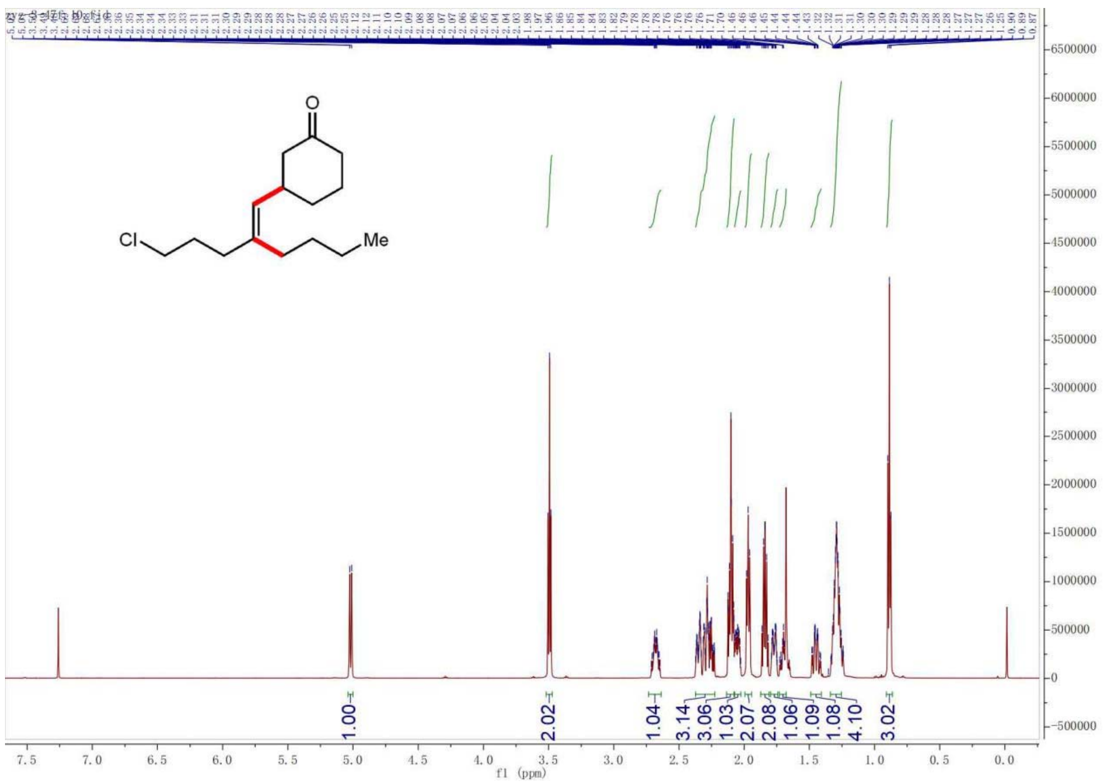

Supplementary Figure 25. ^{1}H NMR (600 MHz, $CDCl_3$) of **4d**

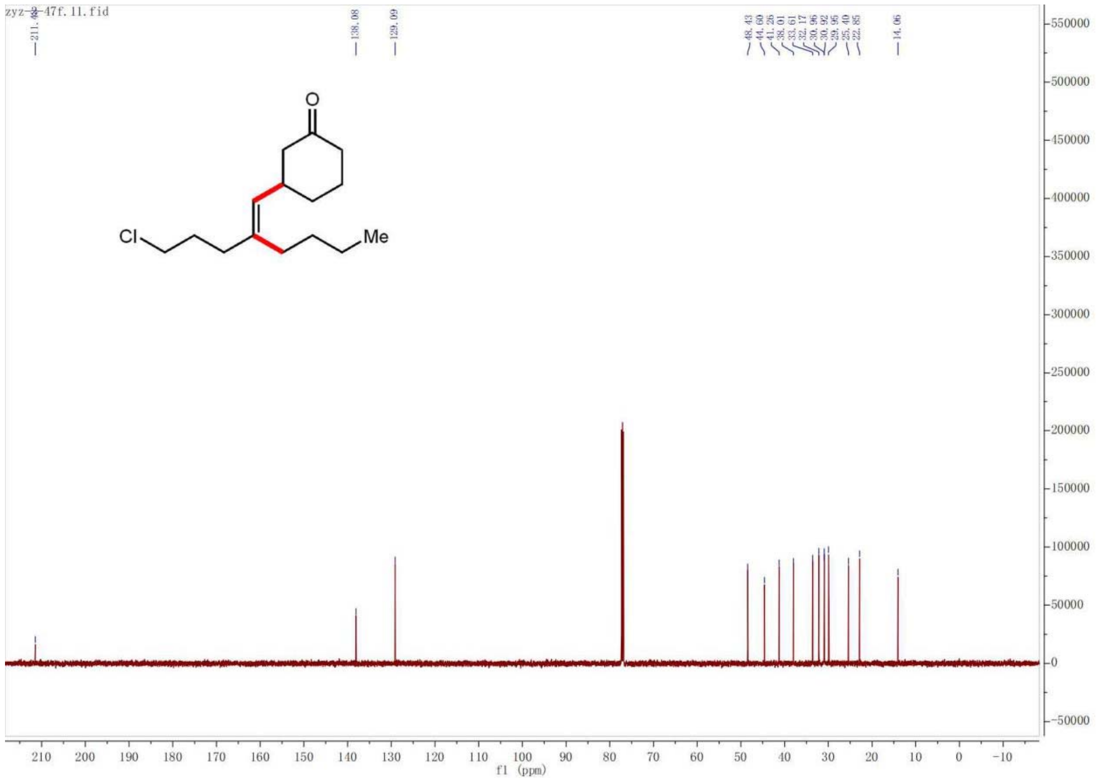

Supplementary Figure 26. ^{13}C NMR (151 MHz, $CDCl_3$) of **4d**

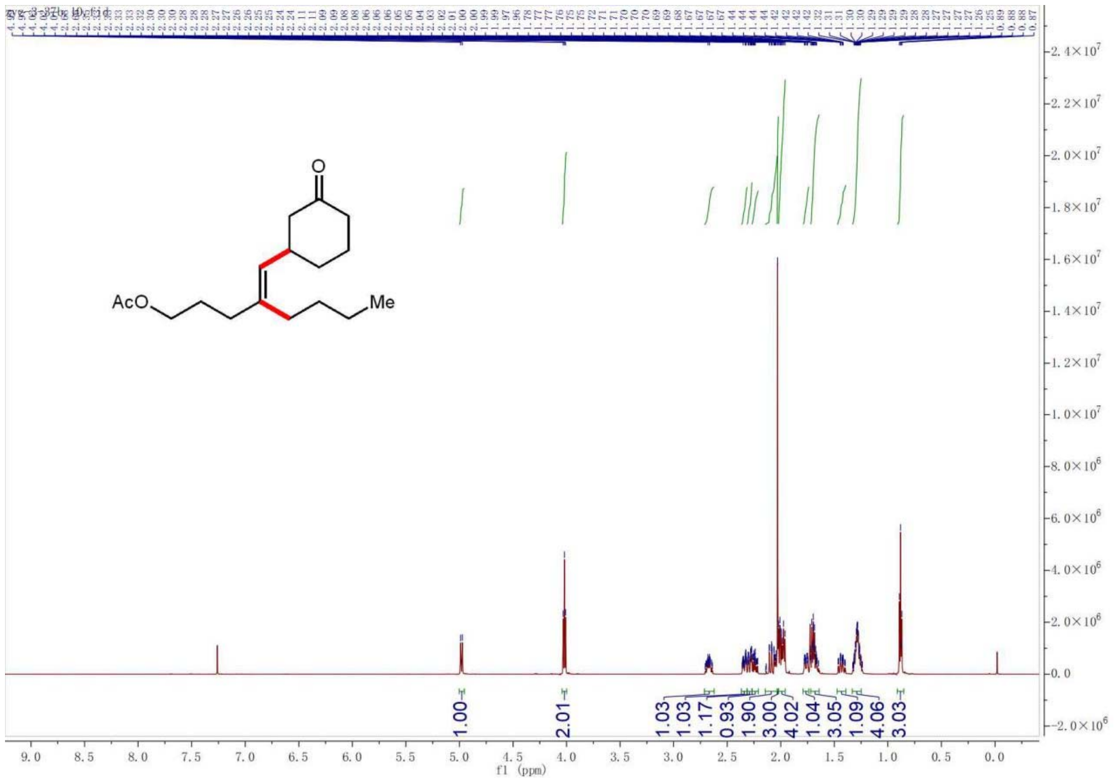

Supplementary Figure 27. ^{1}H NMR (600 MHz, $CDCl_3$) of **4e**

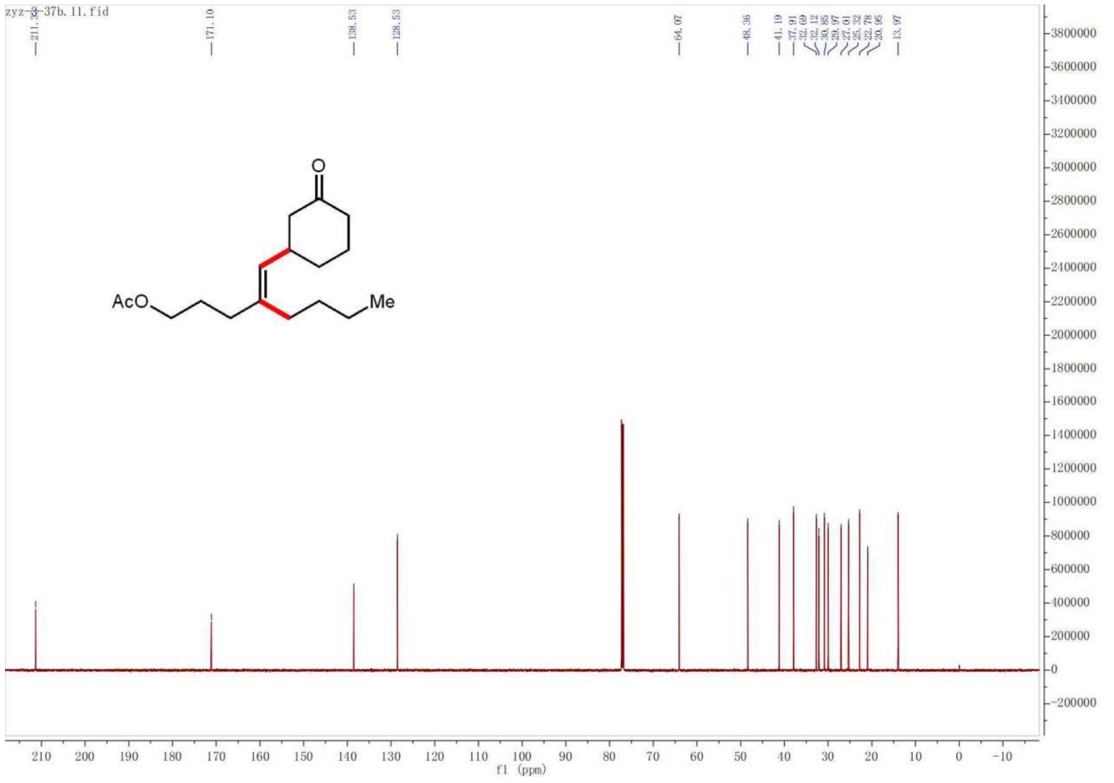

Supplementary Figure 28. ^{13}C NMR (151 MHz, $CDCl_3$) of **4e**

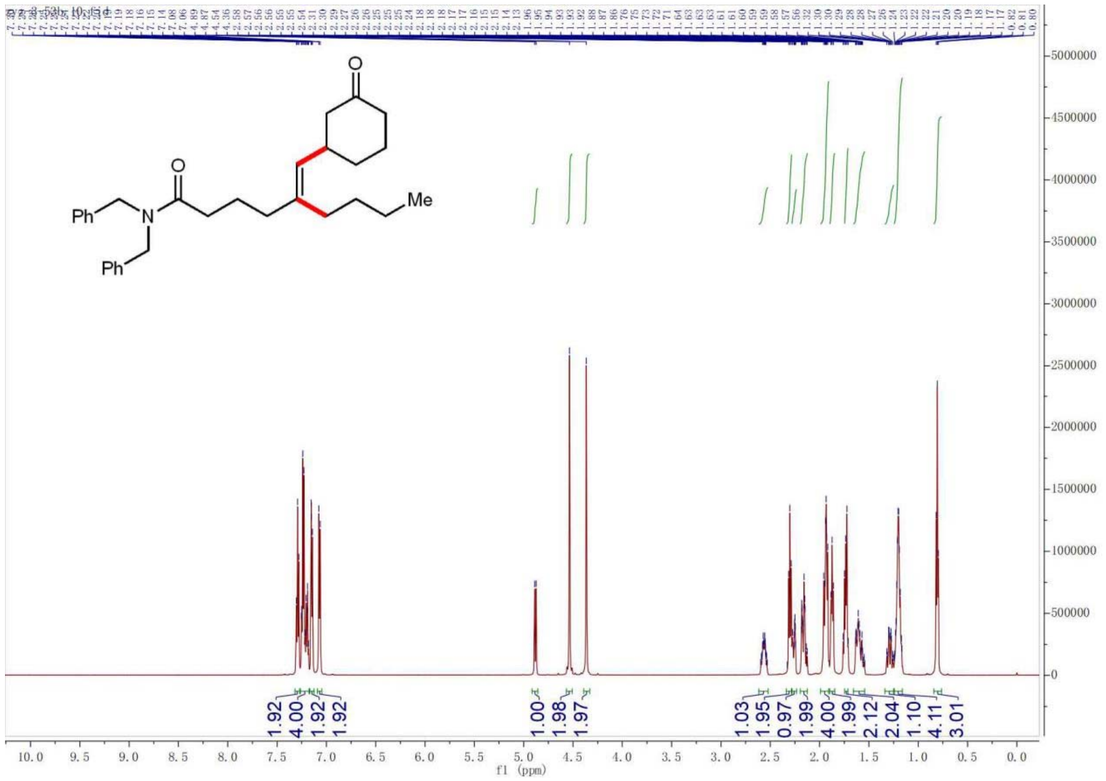

Supplementary Figure 29. ^{1}H NMR (600 MHz, $CDCl_3$) of **4f**

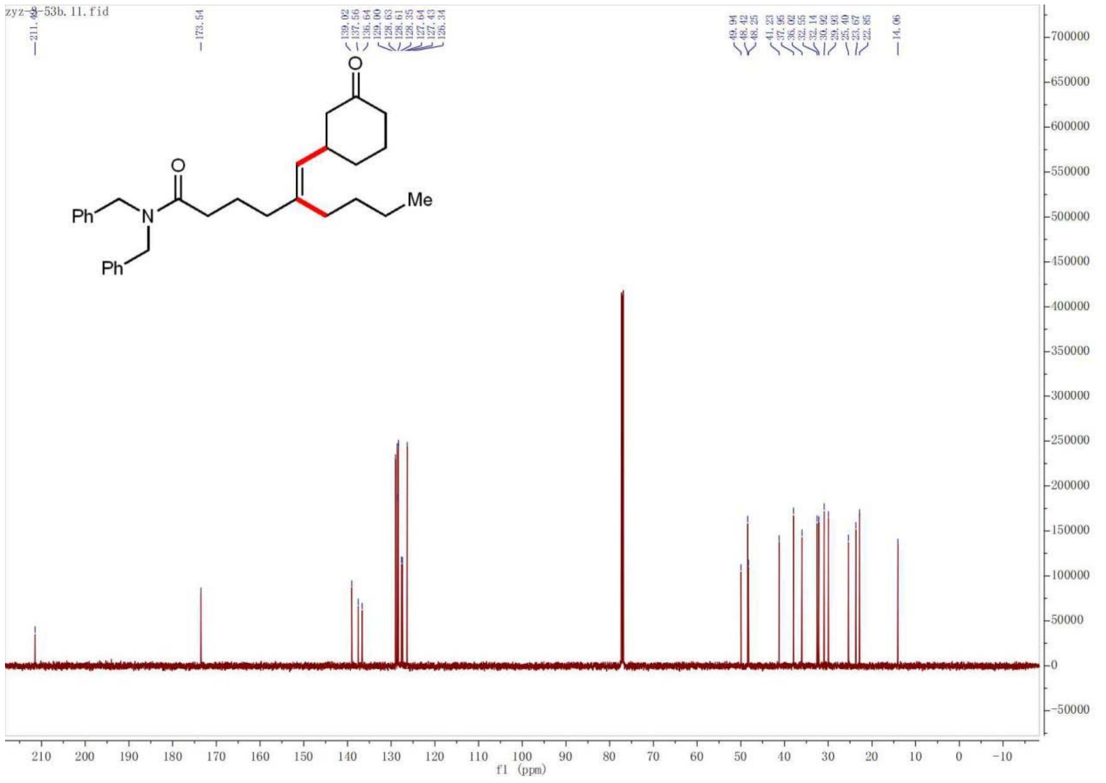

Supplementary Figure 30. ^{13}C NMR (151 MHz, $CDCl_3$) of **4f**

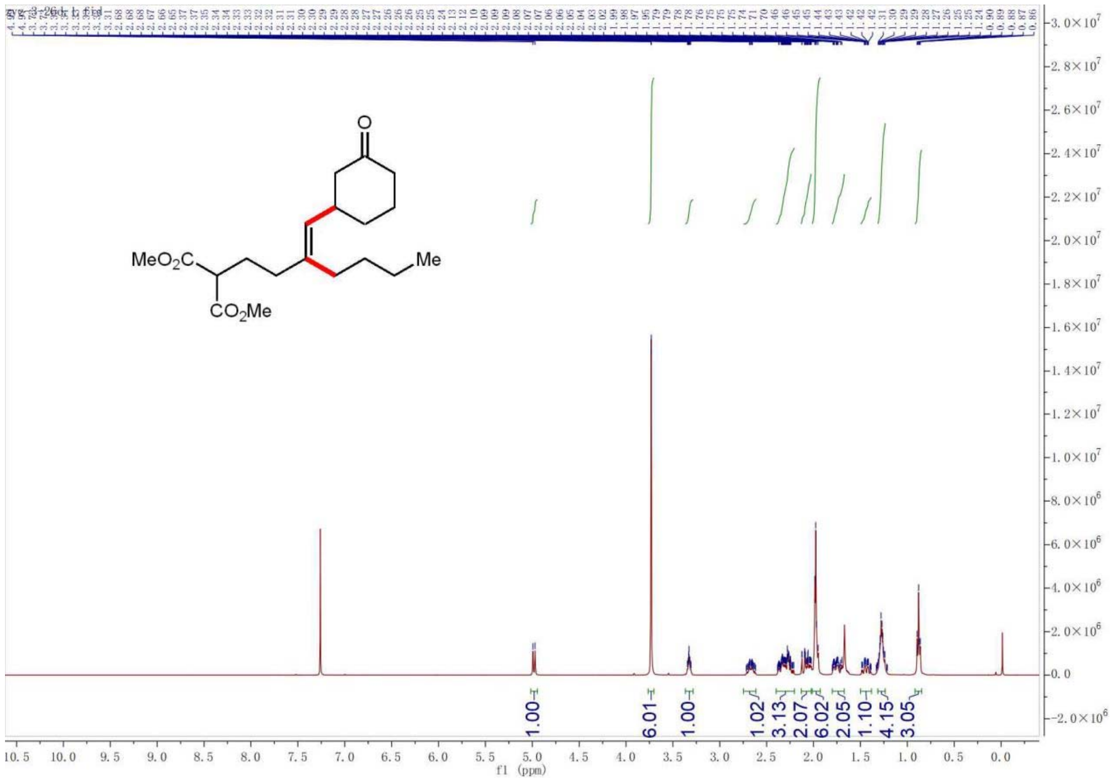

Supplementary Figure 31. ^{1}H NMR (400 MHz, $CDCl_3$) of **4g**

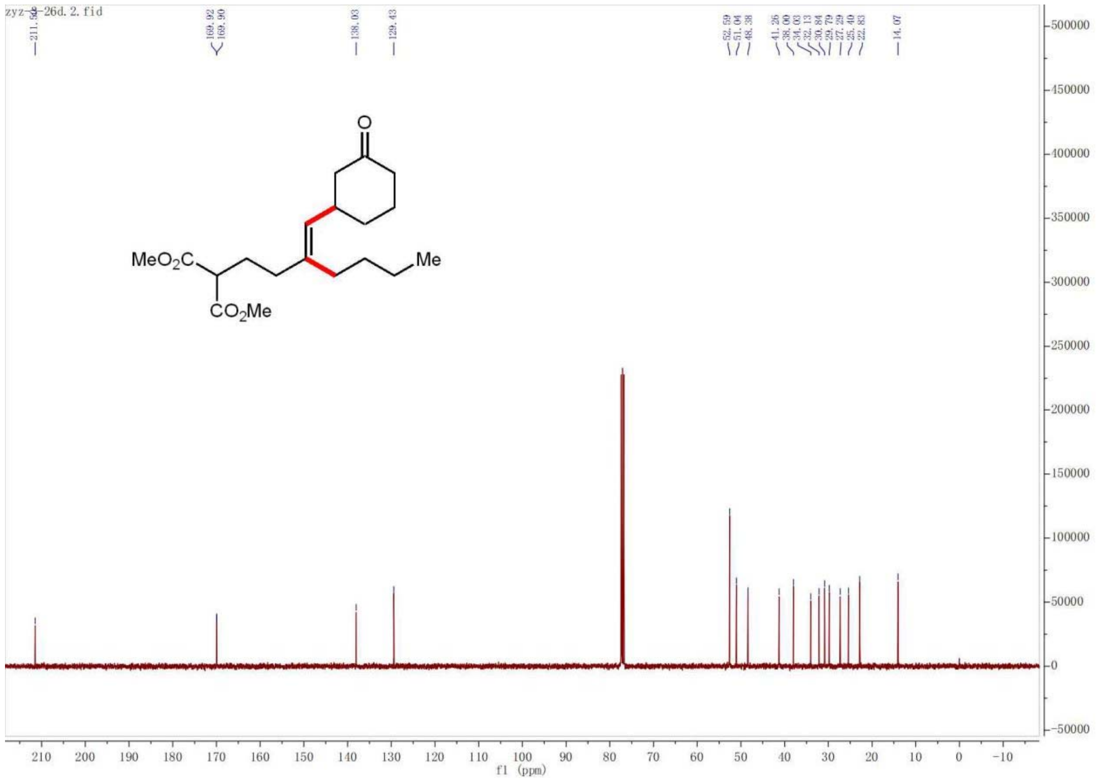

Supplementary Figure 32. ^{13}C NMR (101 MHz, $CDCl_3$) of **4g**

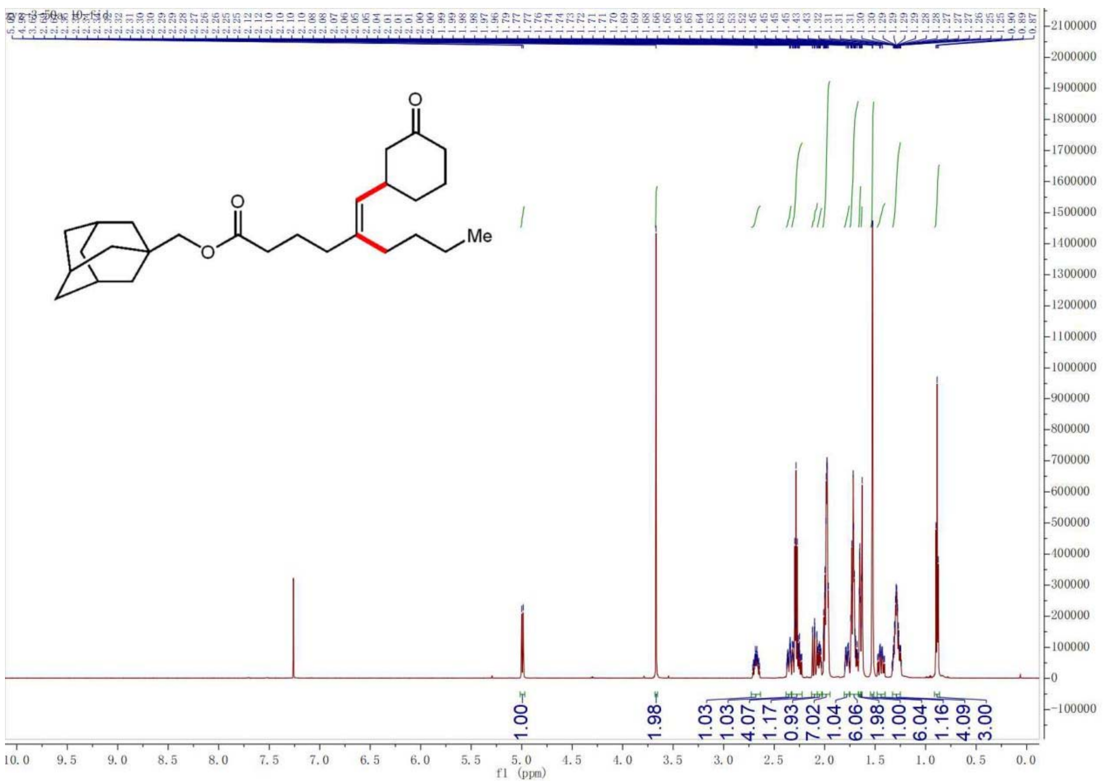

Supplementary Figure 33. ^{1}H NMR (600 MHz, $CDCl_3$) of **4h**

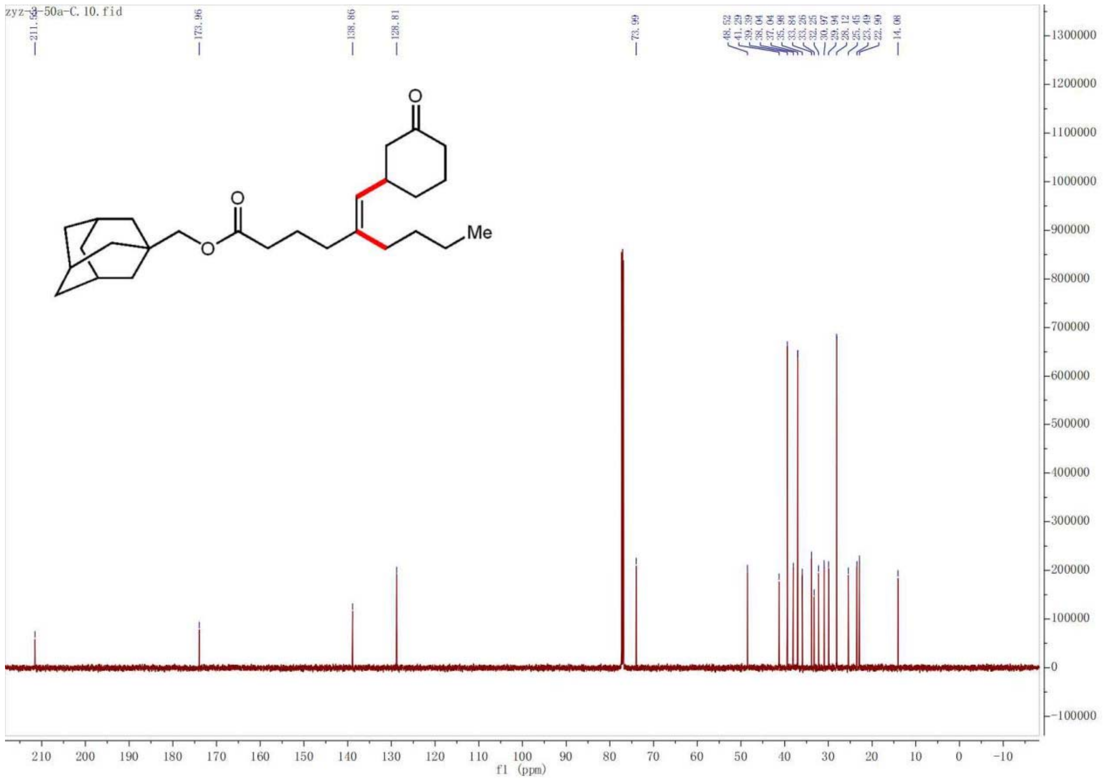

Supplementary Figure 34. ^{13}C NMR (151 MHz, $CDCl_3$) of **4h**

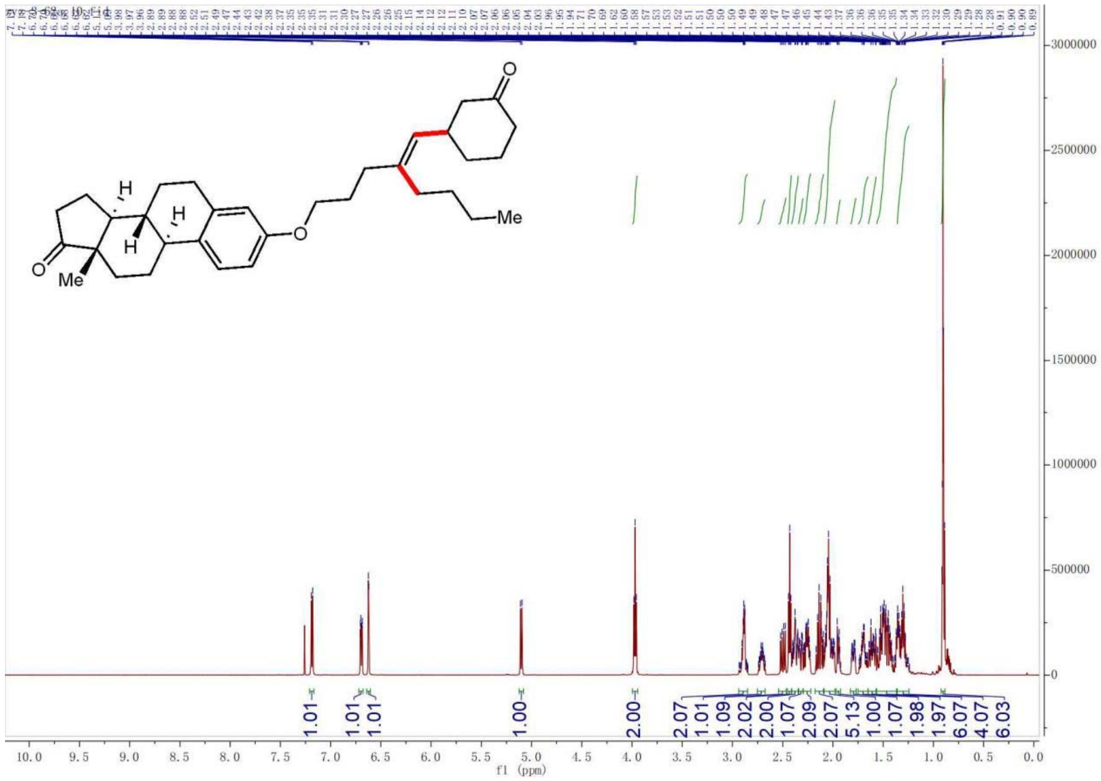

Supplementary Figure 35. ^{1}H NMR (600 MHz, $CDCl_3$) of **4i**

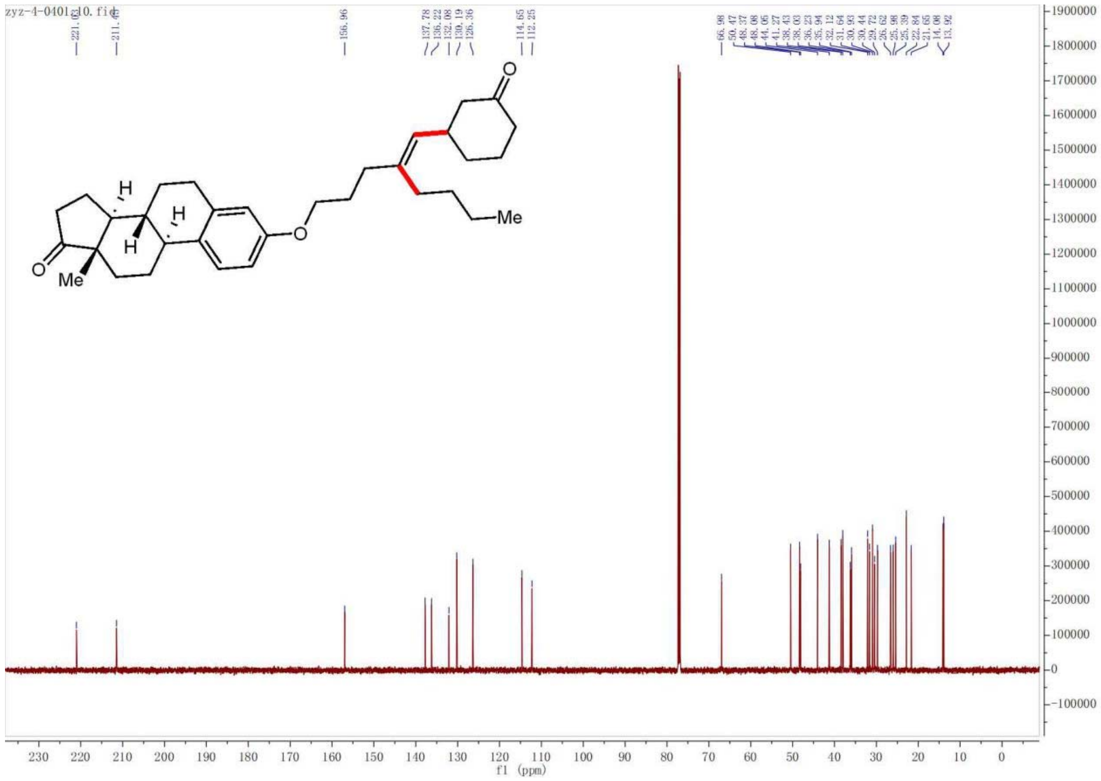

Supplementary Figure 36. ^{13}C NMR (151 MHz, $CDCl_3$) of **4i**

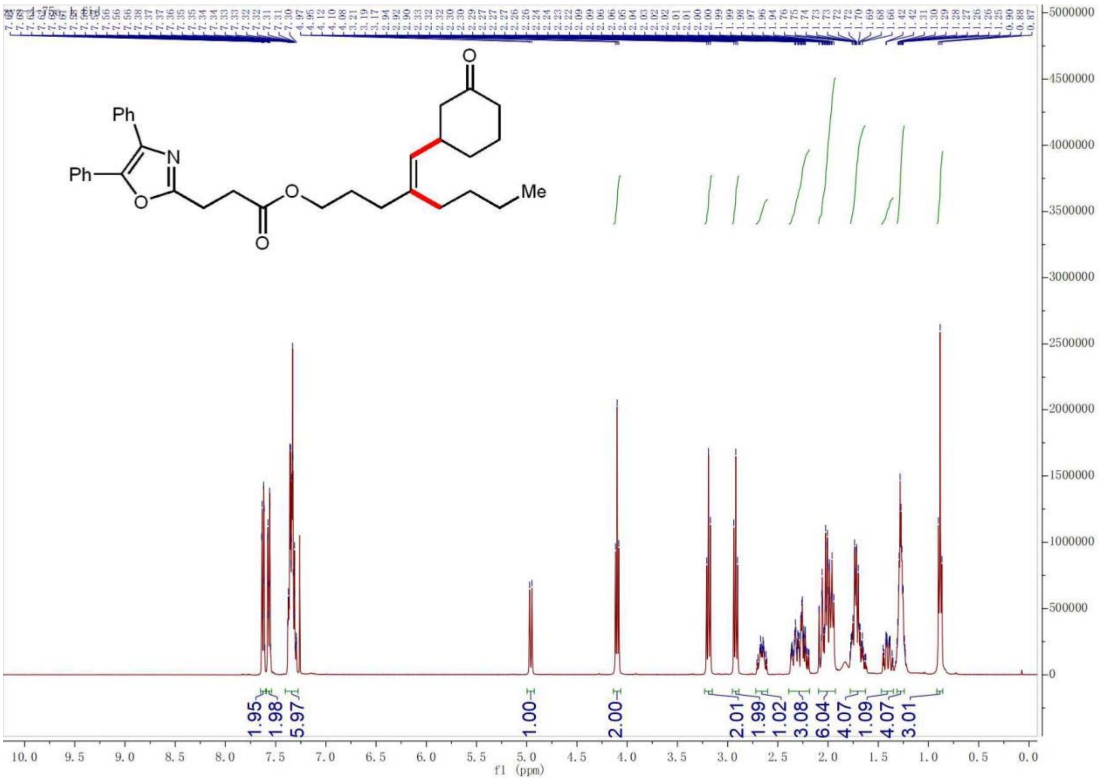

Supplementary Figure 37. ^{1}H NMR (400 MHz, $CDCl_3$) of **4j**

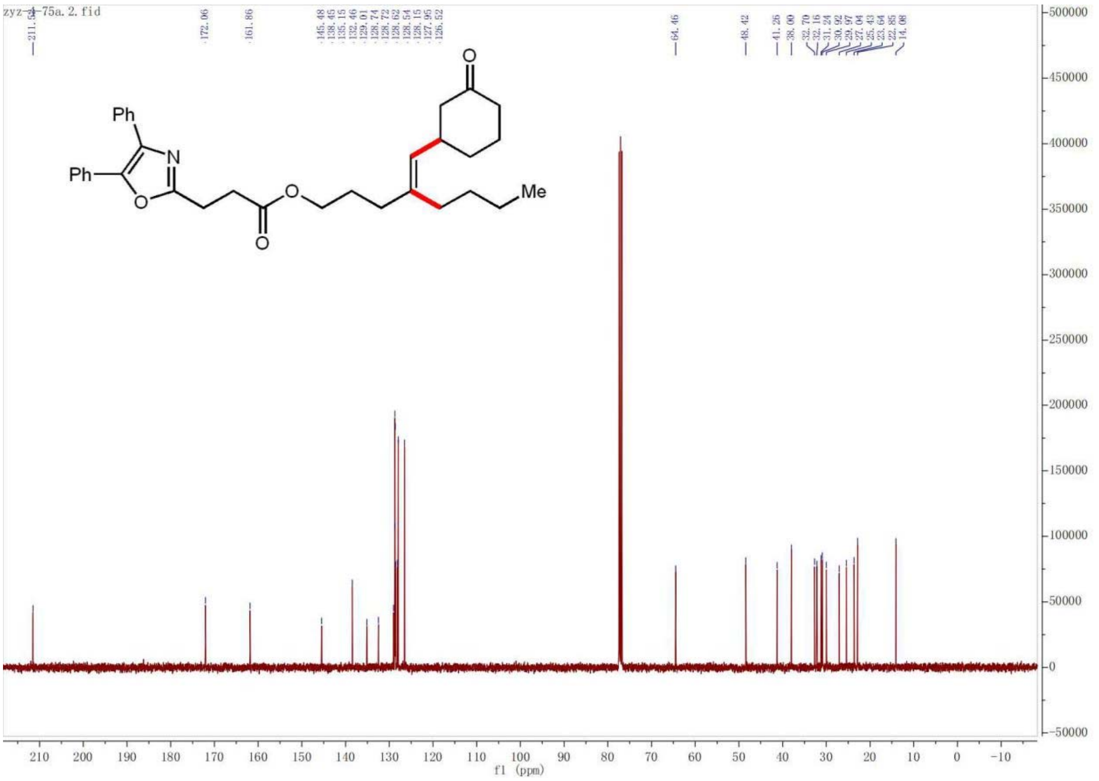

Supplementary Figure 38. ^{13}C NMR (101 MHz, $CDCl_3$) of **4j**

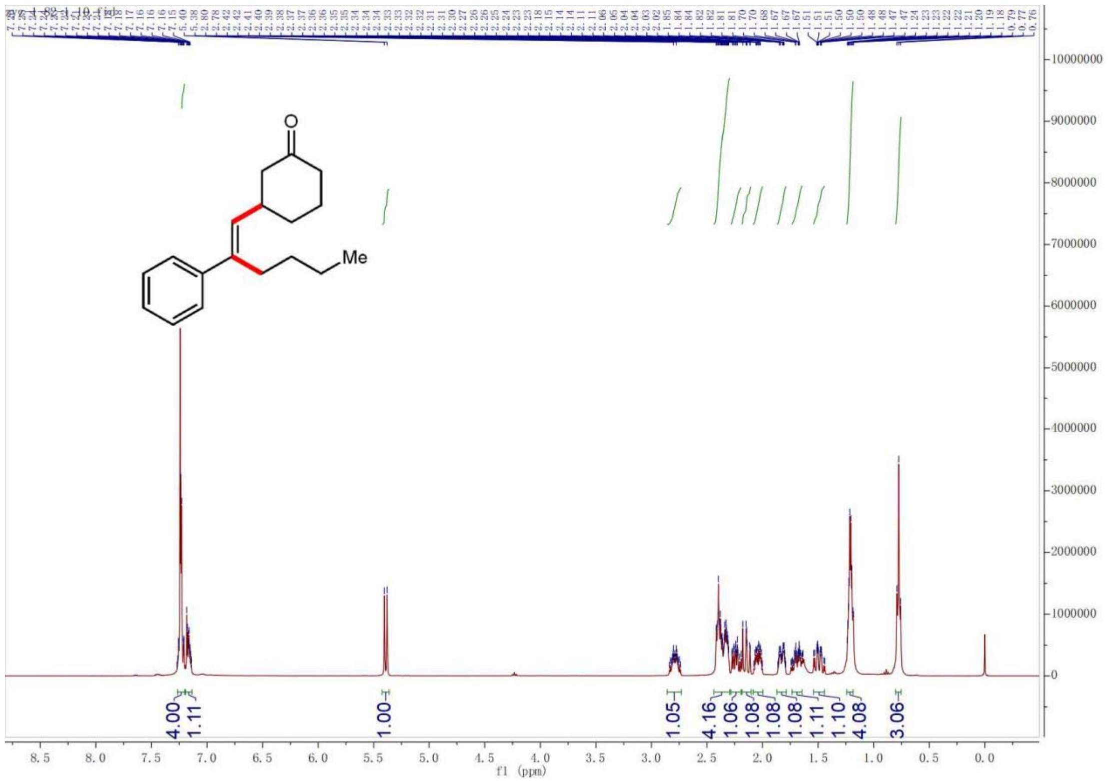

Supplementary Figure 39. ^{1}H NMR (400 MHz, $CDCl_3$) of **5a**

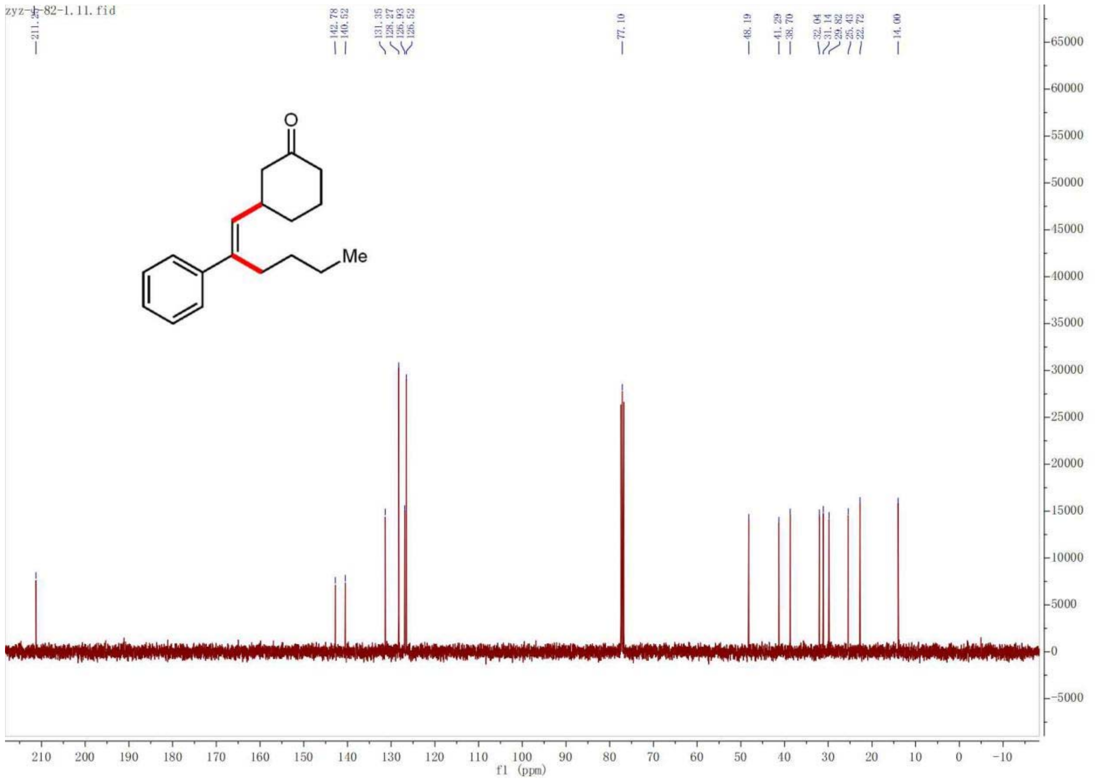

Supplementary Figure 40. ^{13}C NMR (101 MHz, $CDCl_3$) of **5a**

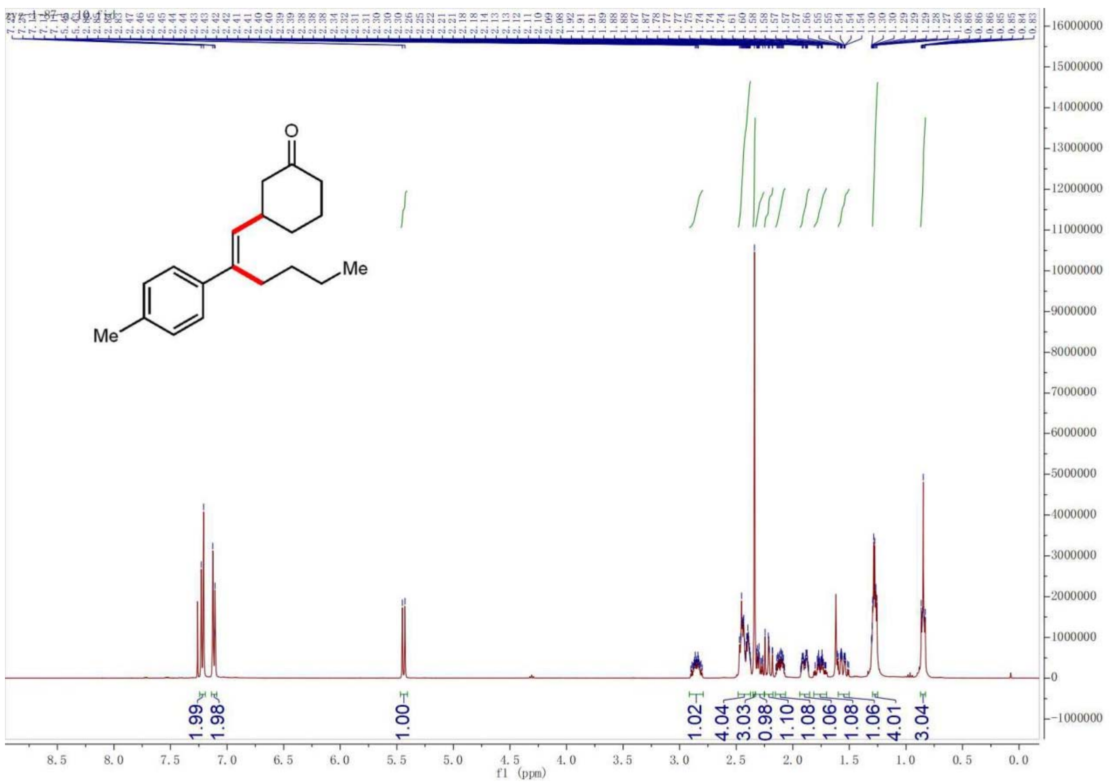

Supplementary Figure 41. ^{1}H NMR (400 MHz, $CDCl_3$) of **5b**

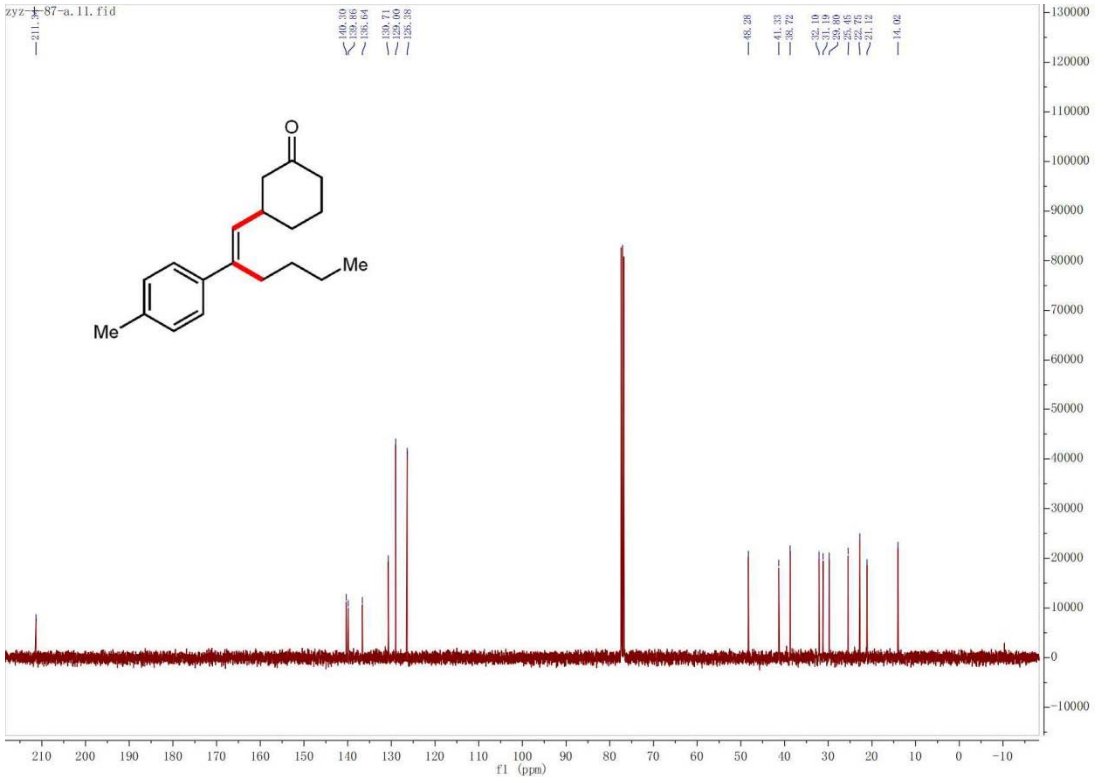

Supplementary Figure 42. ^{13}C NMR (101 MHz, $CDCl_3$) of **5b**

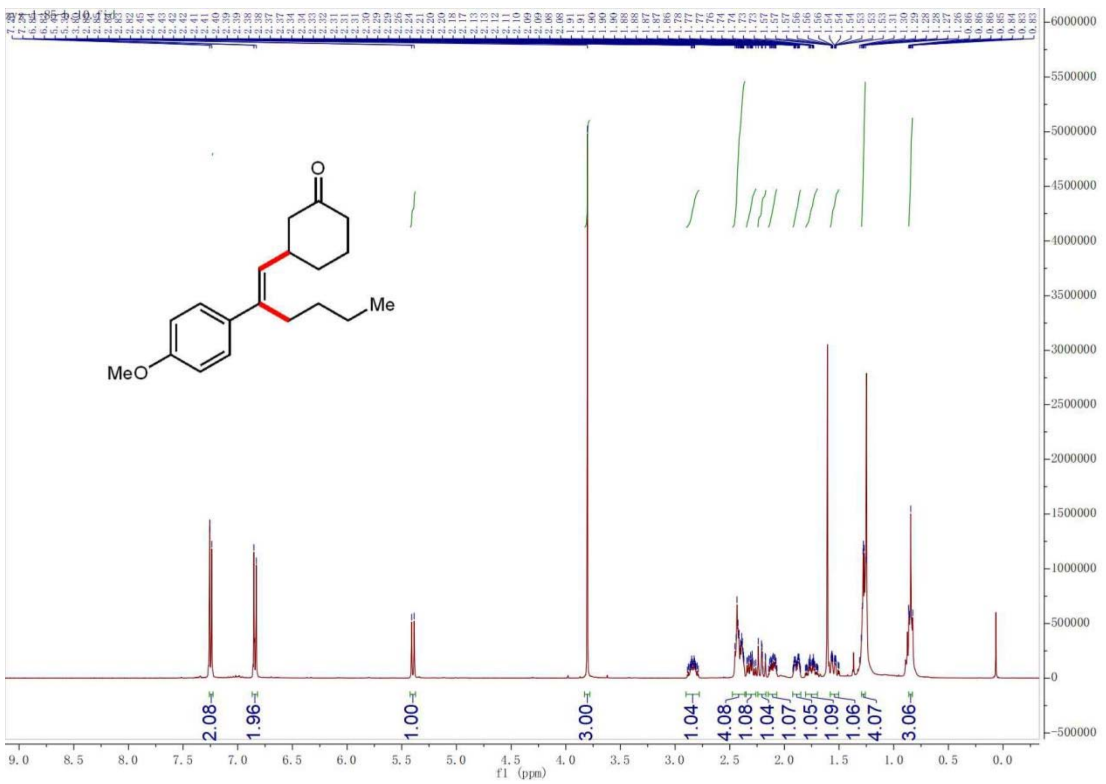

Supplementary Figure 43. ^{1}H NMR (400 MHz, $CDCl_3$) of **5c**

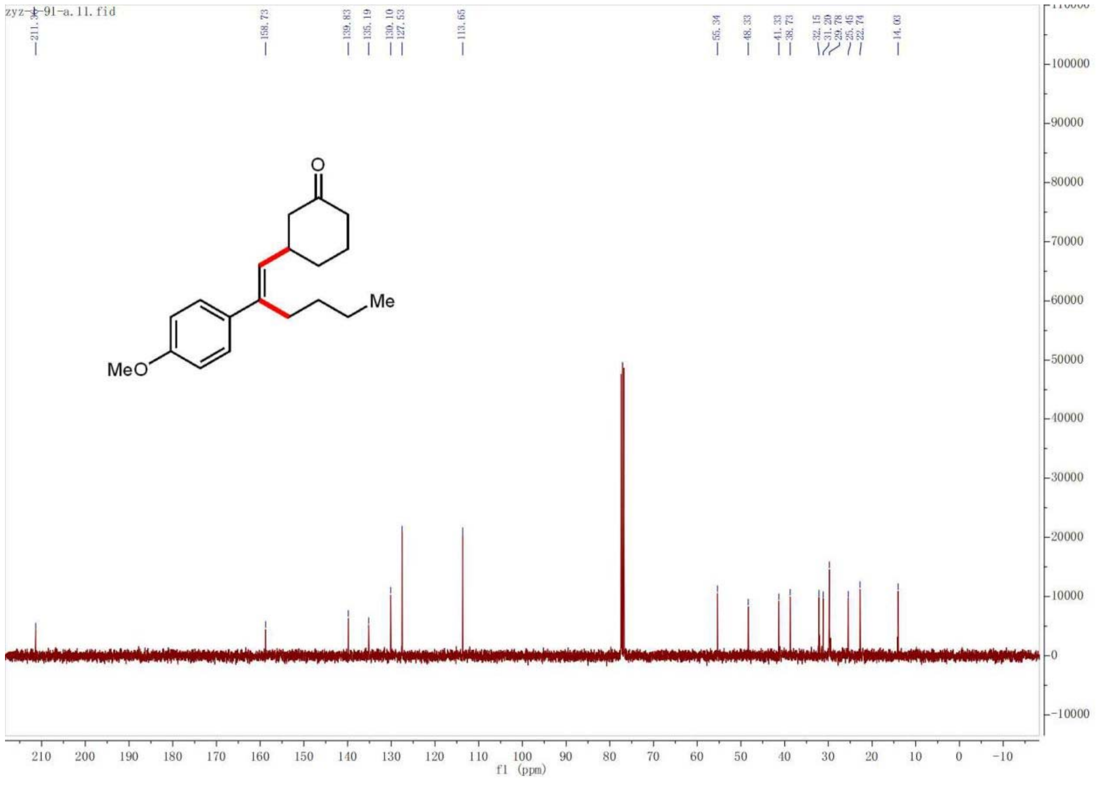

Supplementary Figure 44. ^{13}C NMR (101 MHz, $CDCl_3$) of **5c**

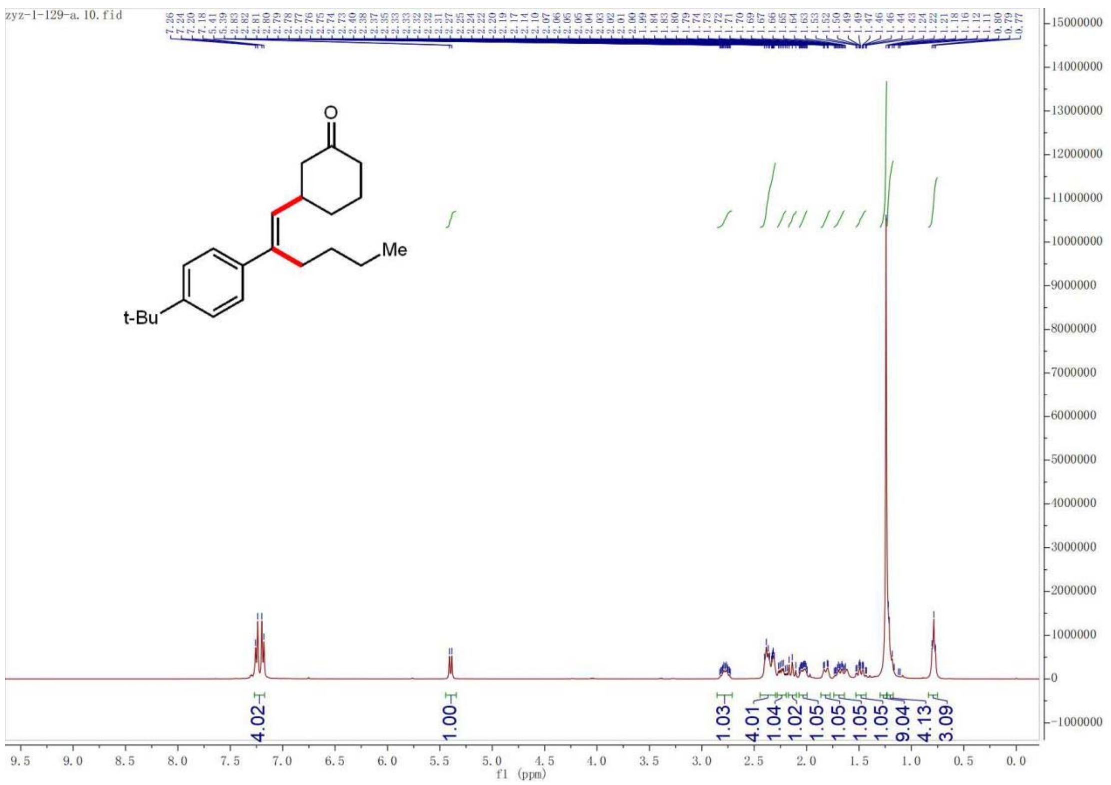

Supplementary Figure 45. ^{1}H NMR (400 MHz, $CDCl_3$) of **5d**

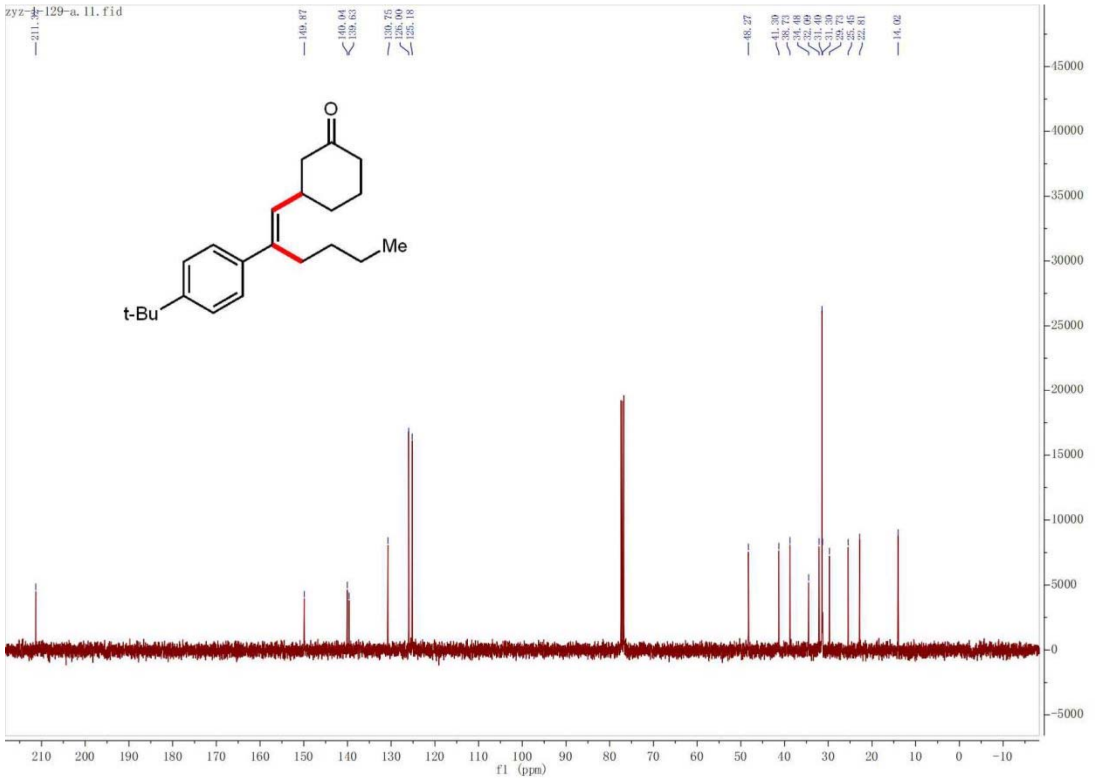

Supplementary Figure 46. ^{13}C NMR (101 MHz, $CDCl_3$) of **5d**

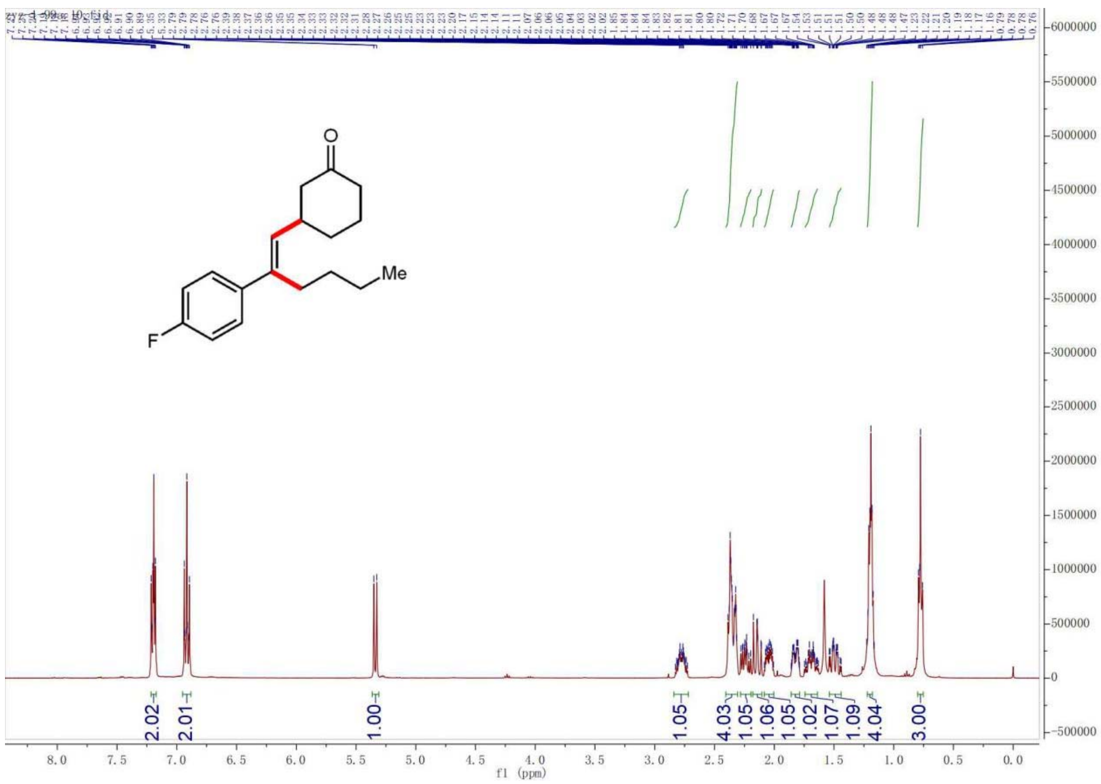

Supplementary Figure 47. ^{1}H NMR (400 MHz, $CDCl_3$) of **5e**

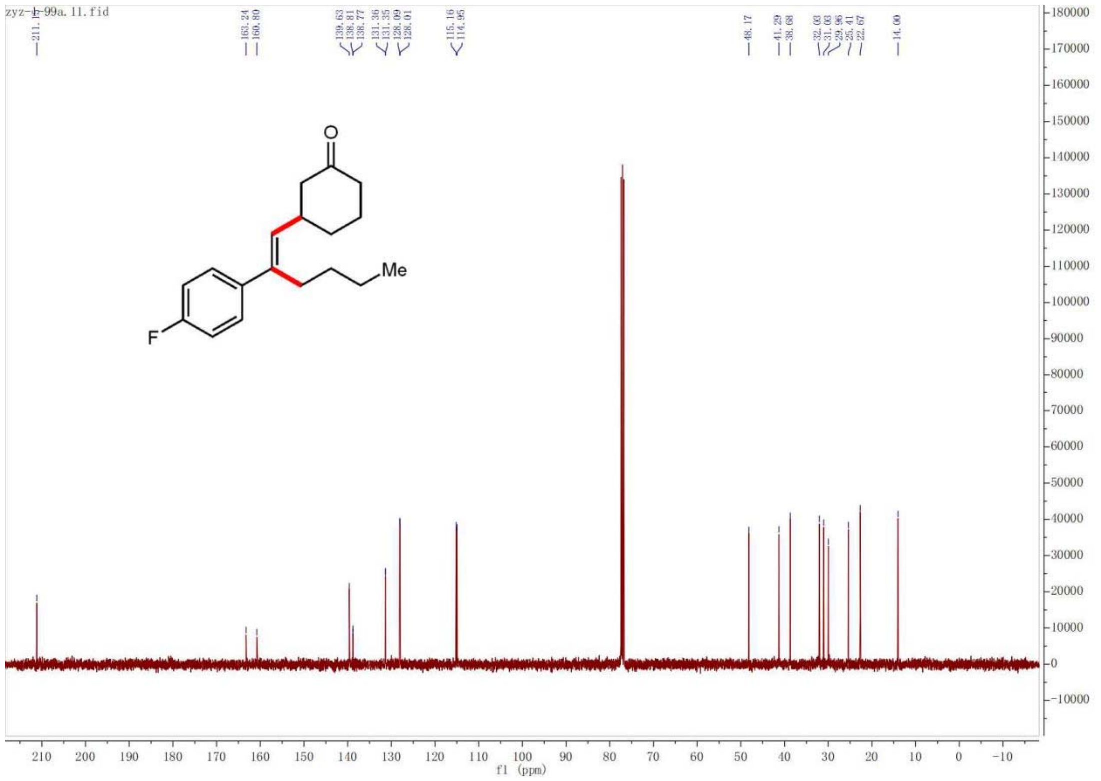

Supplementary Figure 48. ^{13}C NMR (101 MHz, $CDCl_3$) of **5e**

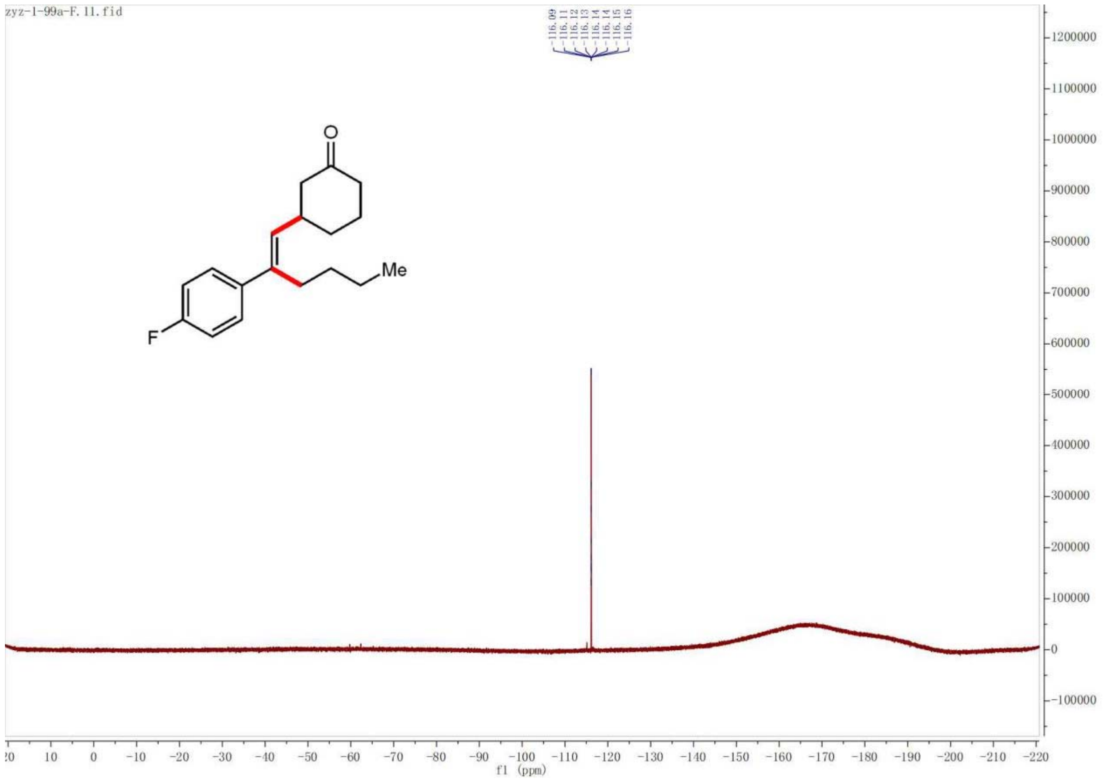

Supplementary Figure 49. ^{19}F NMR (376 MHz, $CDCl_3$) of **5e**

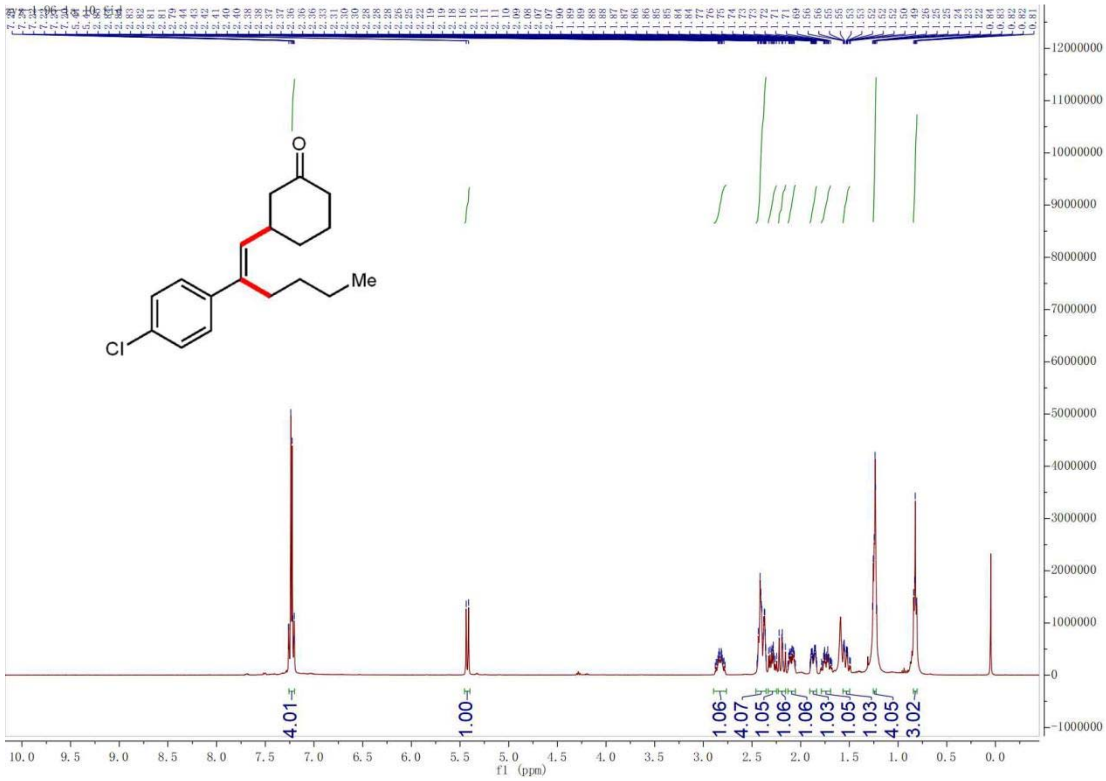

Supplementary Figure 50. ^{1}H NMR (400 MHz, $CDCl_3$) of **5f**

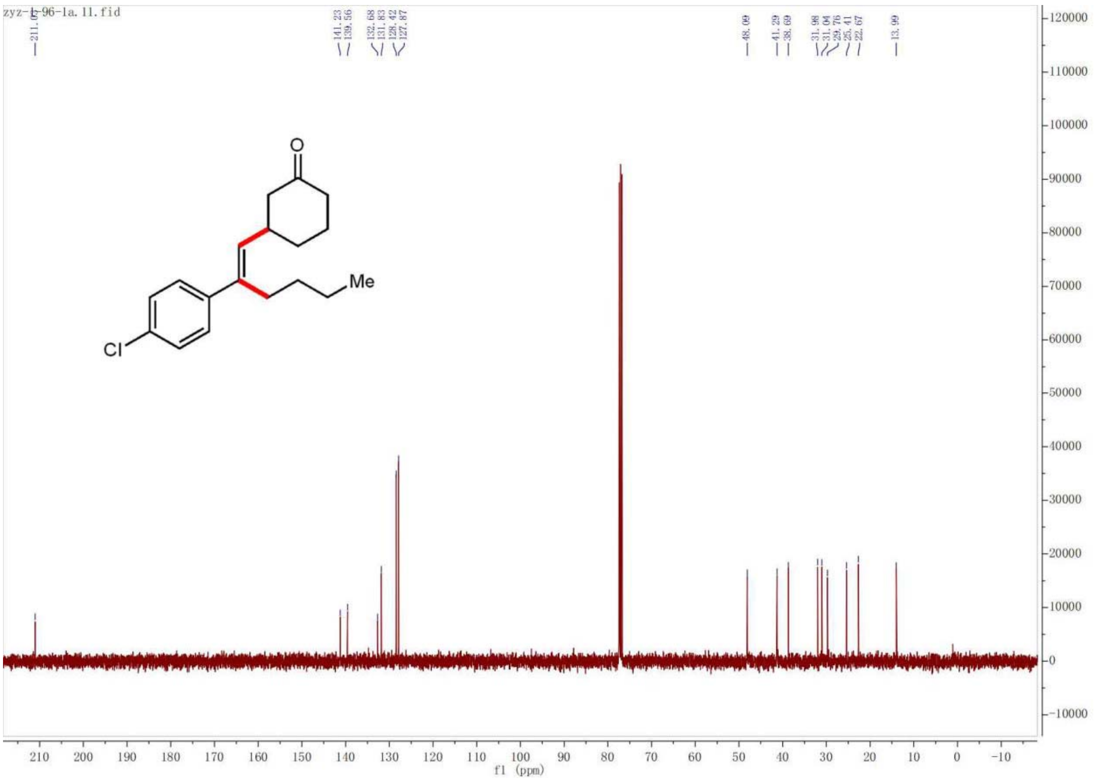

Supplementary Figure 51. ^{13}C NMR (101 MHz, $CDCl_3$) of **5f**

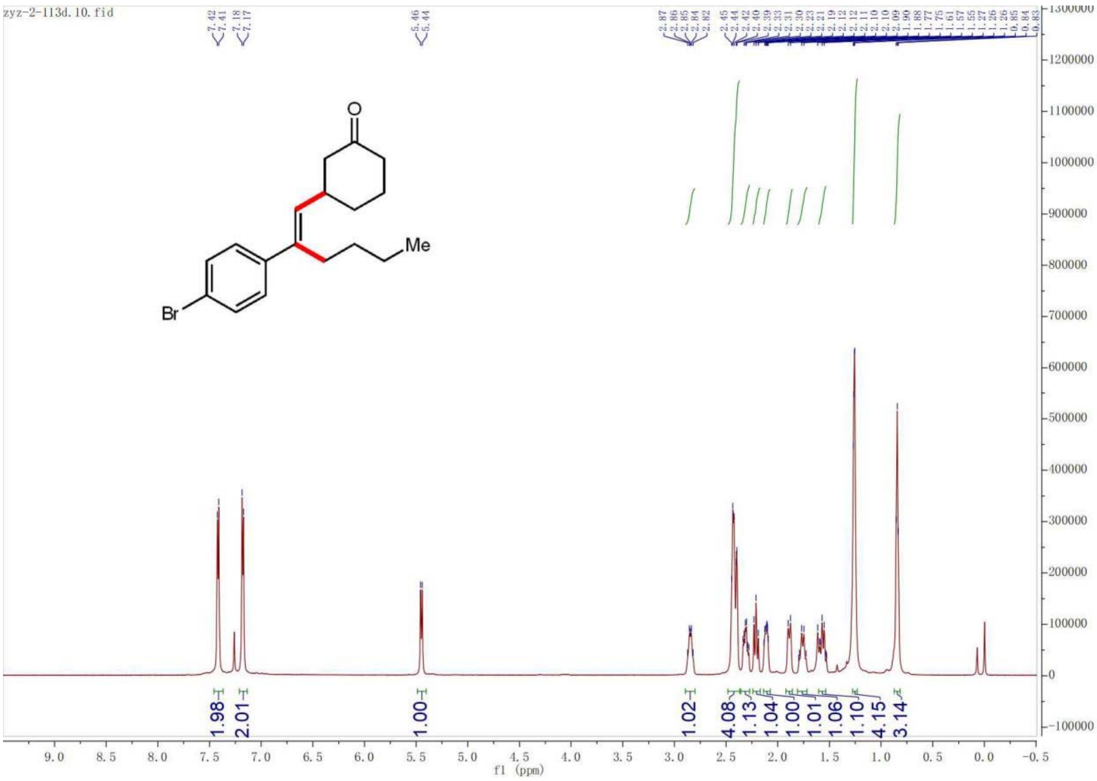

Supplementary Figure 52. ^{1}H NMR (600 MHz, $CDCl_3$) of **5g**

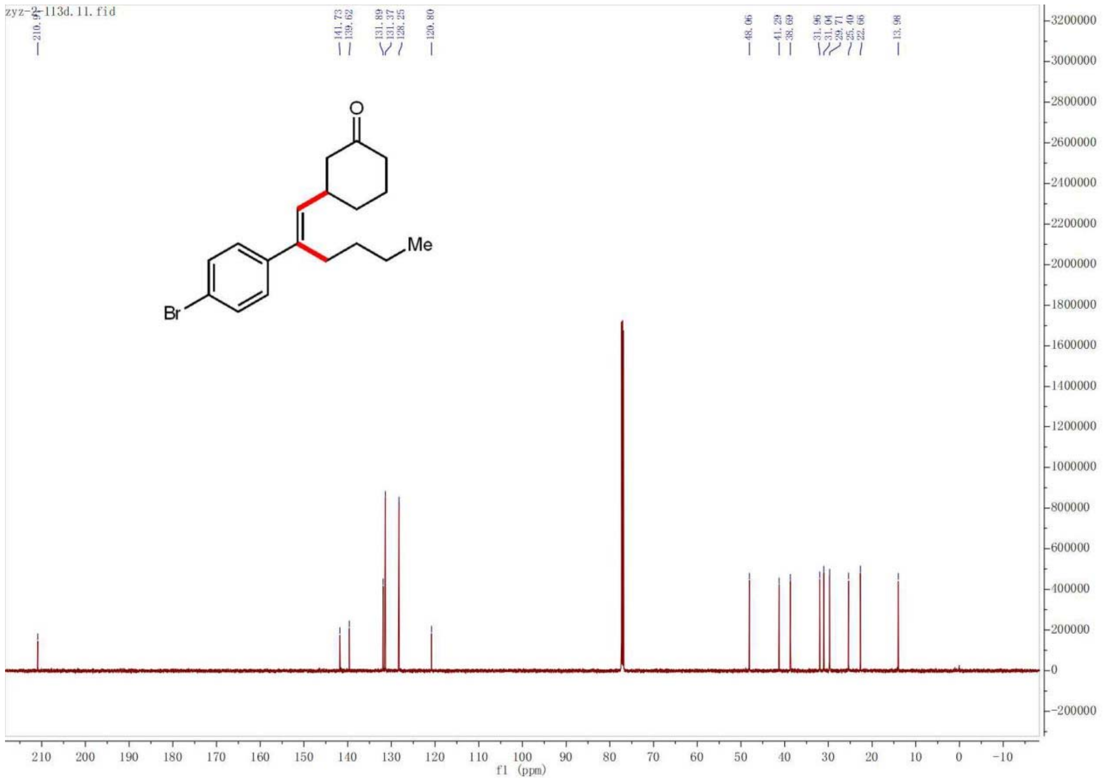

Supplementary Figure 53. ^{13}C NMR (151 MHz, $CDCl_3$) of **5g**

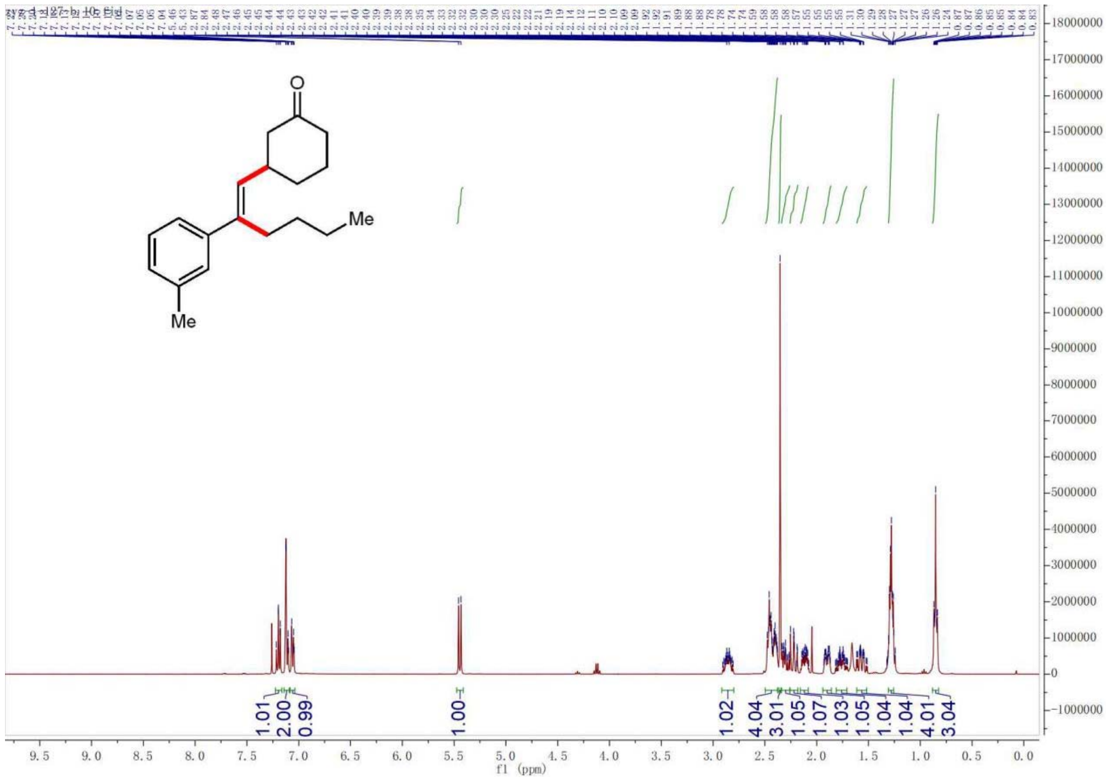

Supplementary Figure 54. ^{1}H NMR (400 MHz, $CDCl_3$) of **5h**

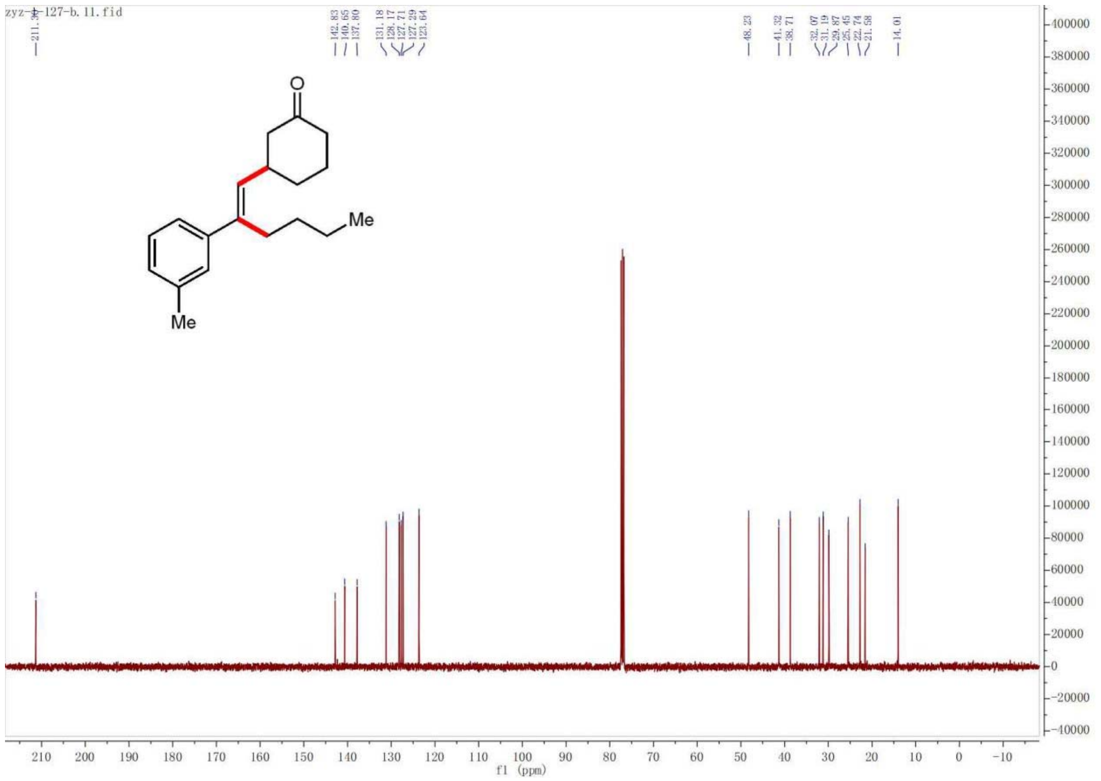

Supplementary Figure 55. ^{13}C NMR (101 MHz, $CDCl_3$) of **5h**

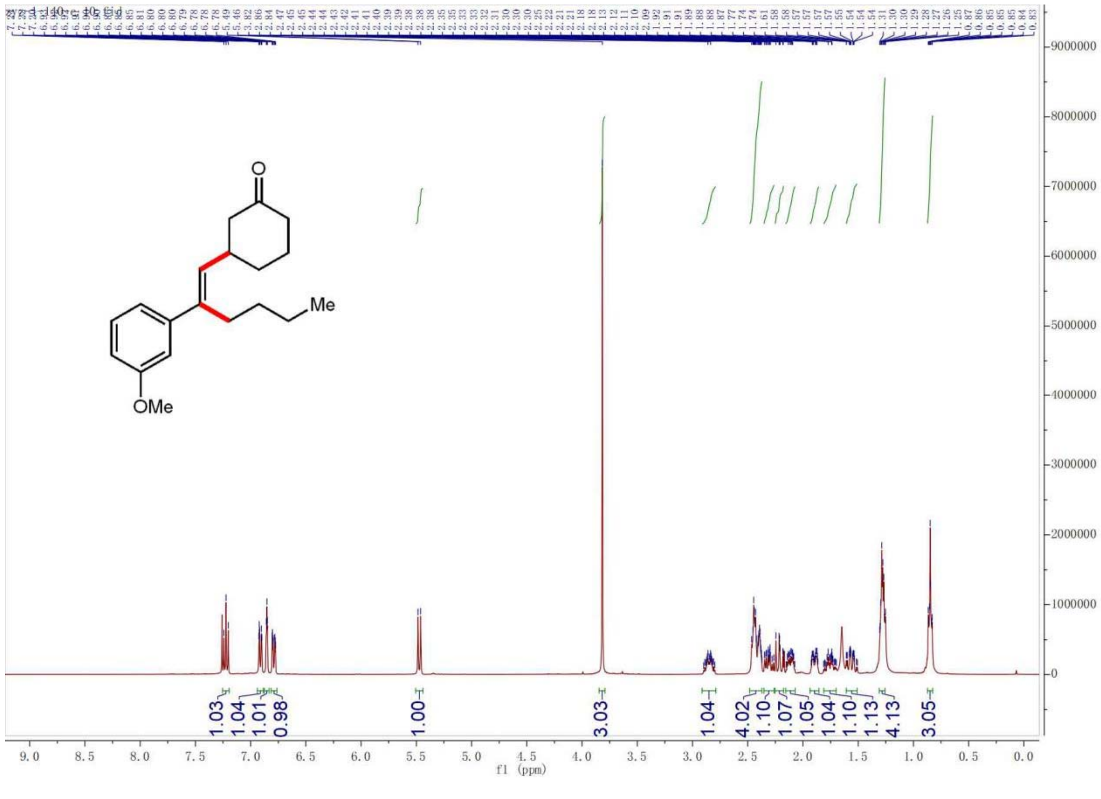

Supplementary Figure 56. ^{1}H NMR (400 MHz, $CDCl_3$) of **5i**

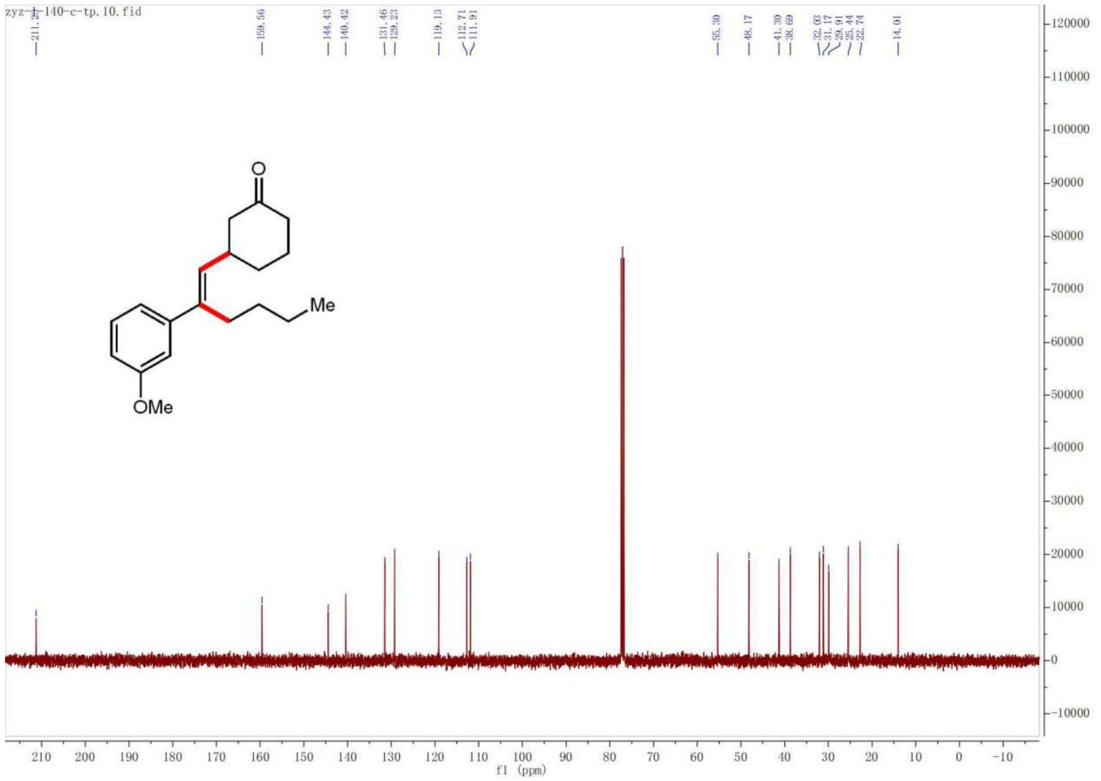

Supplementary Figure 57. ^{13}C NMR (101 MHz, $CDCl_3$) of **5i**

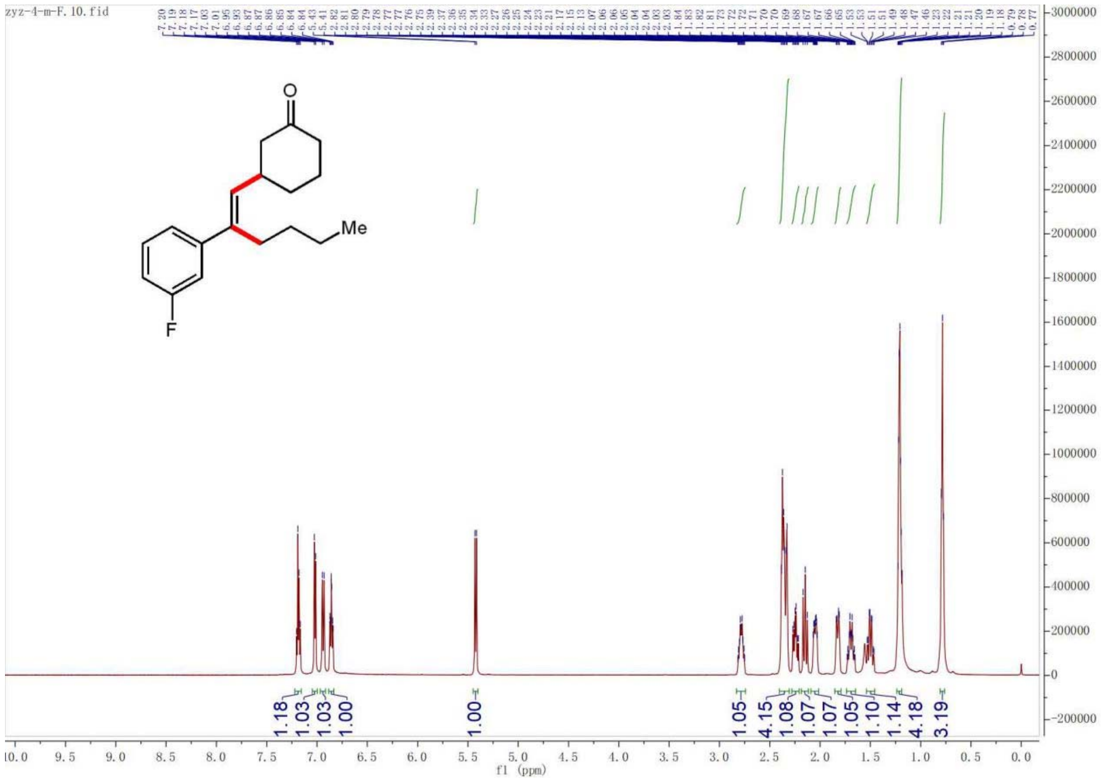

Supplementary Figure 58. ^{1}H NMR (600 MHz, $CDCl_3$) of **5j**

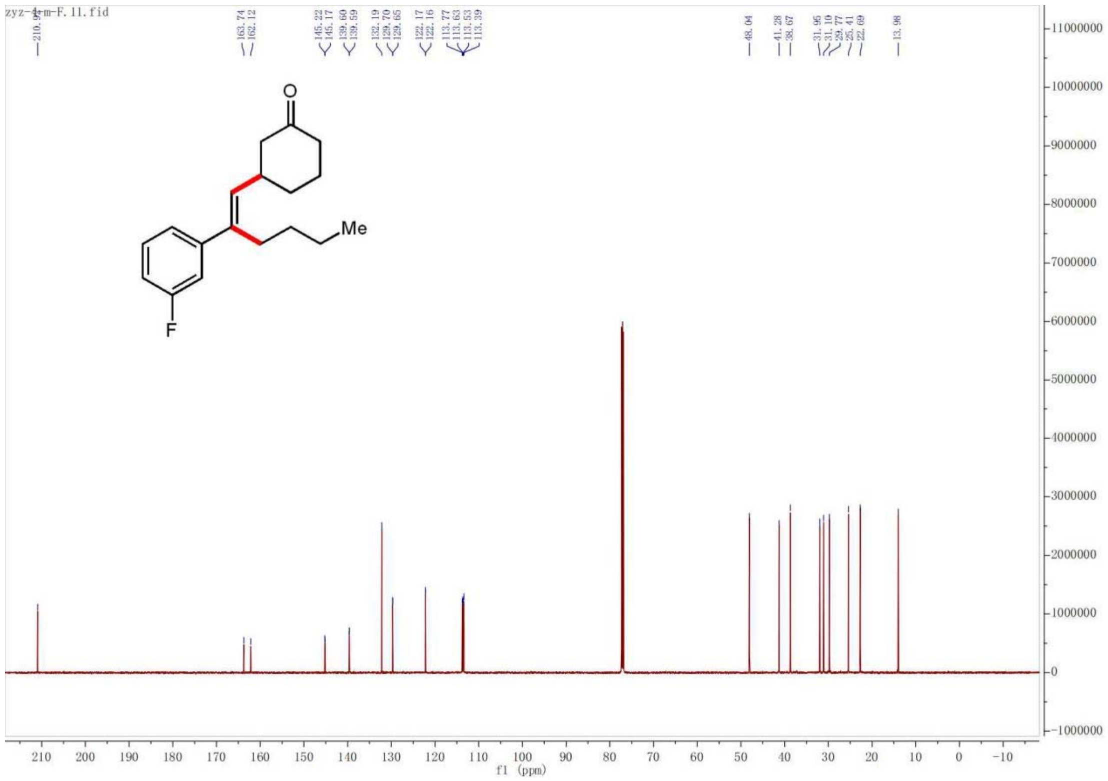

Supplementary Figure 59. ^{13}C NMR (151 MHz, $CDCl_3$) of **5j**

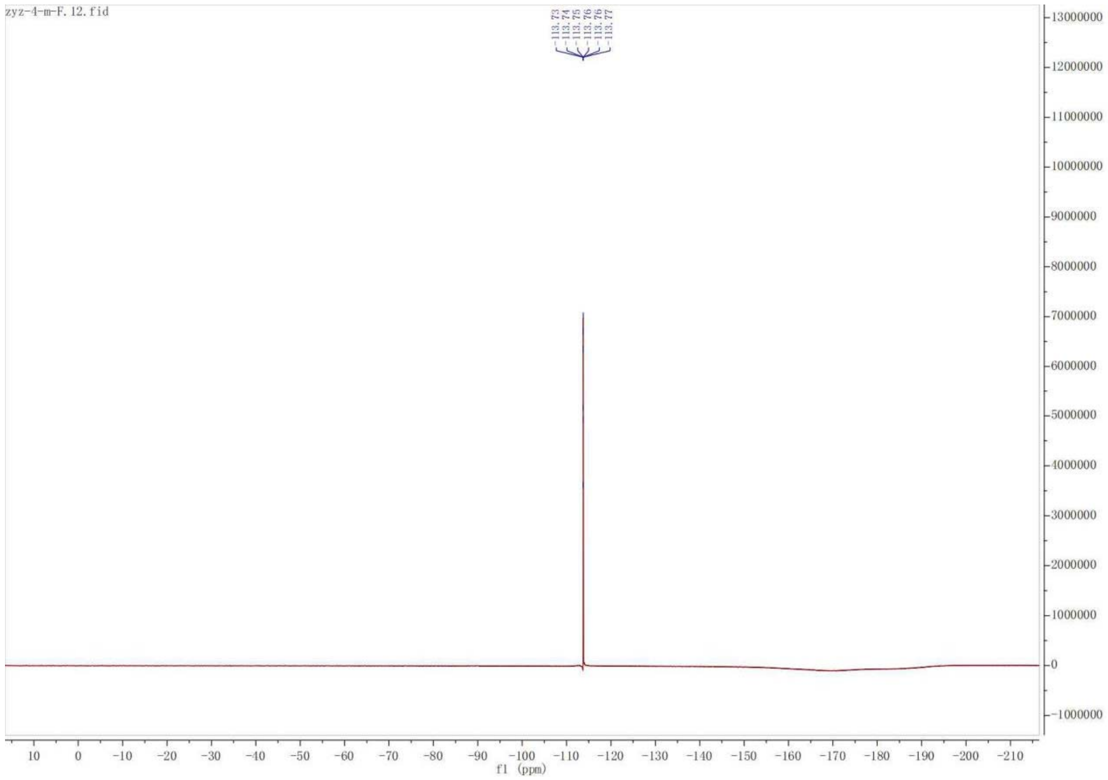

Supplementary Figure 60. ^{19}F NMR (565 MHz, $CDCl_3$) of **5j**

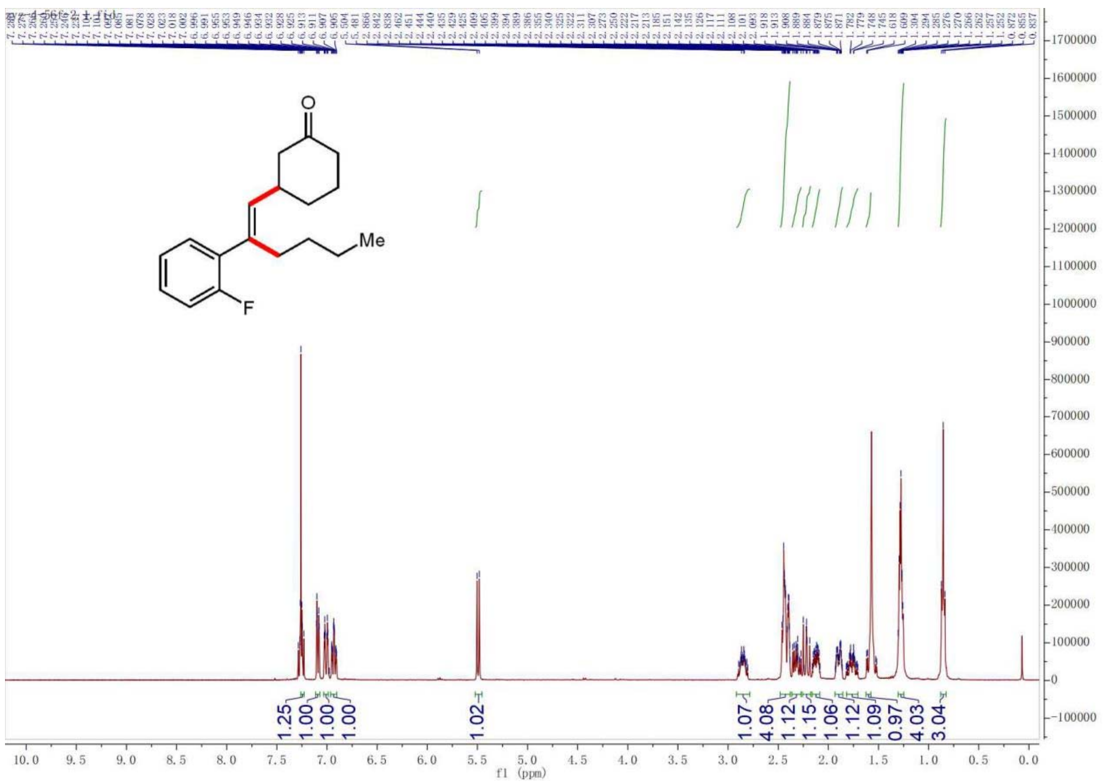

Supplementary Figure 61. ^{1}H NMR (400 MHz, $CDCl_3$) of **5k**

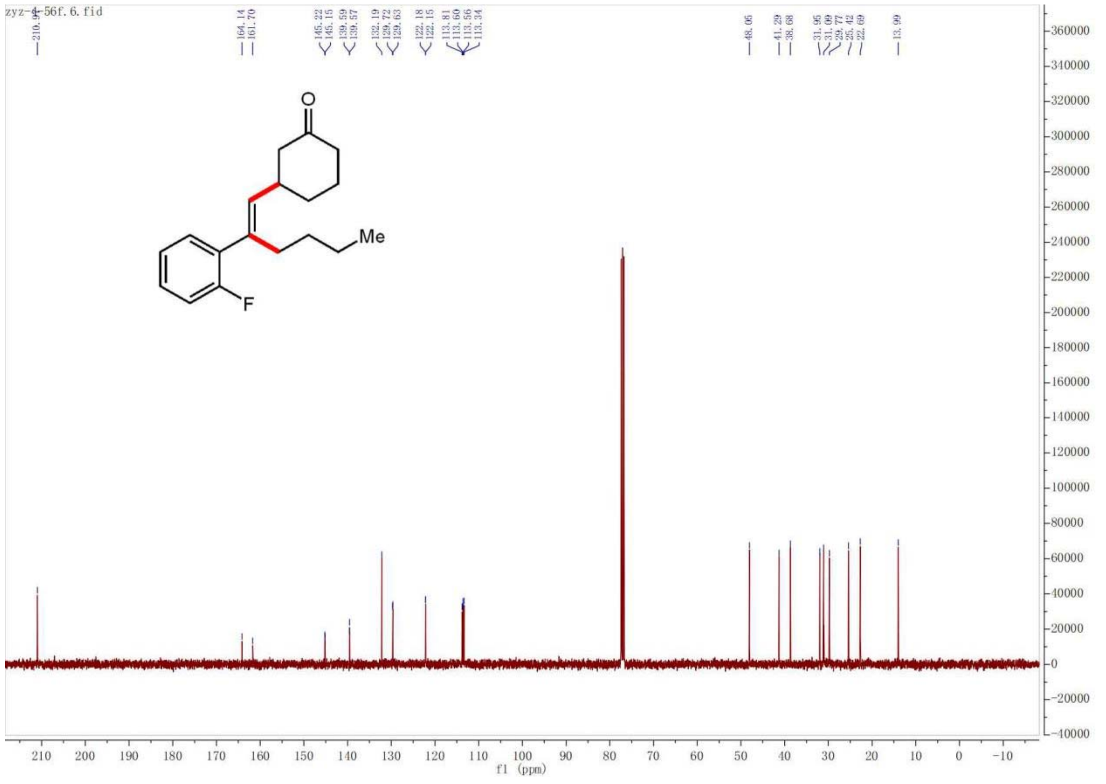

Supplementary Figure 62. ^{13}C NMR (101 MHz, $CDCl_3$) of **5k**

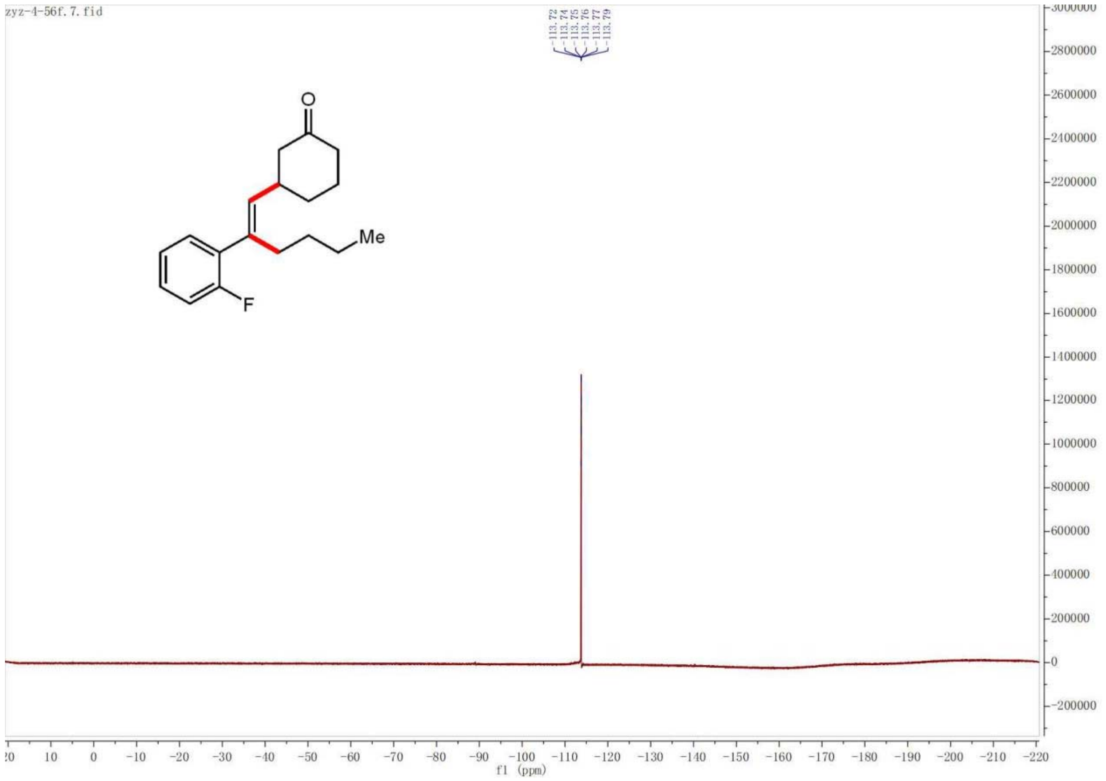

Supplementary Figure 63. ^{19}F NMR (376 MHz, $CDCl_3$) of **5k**

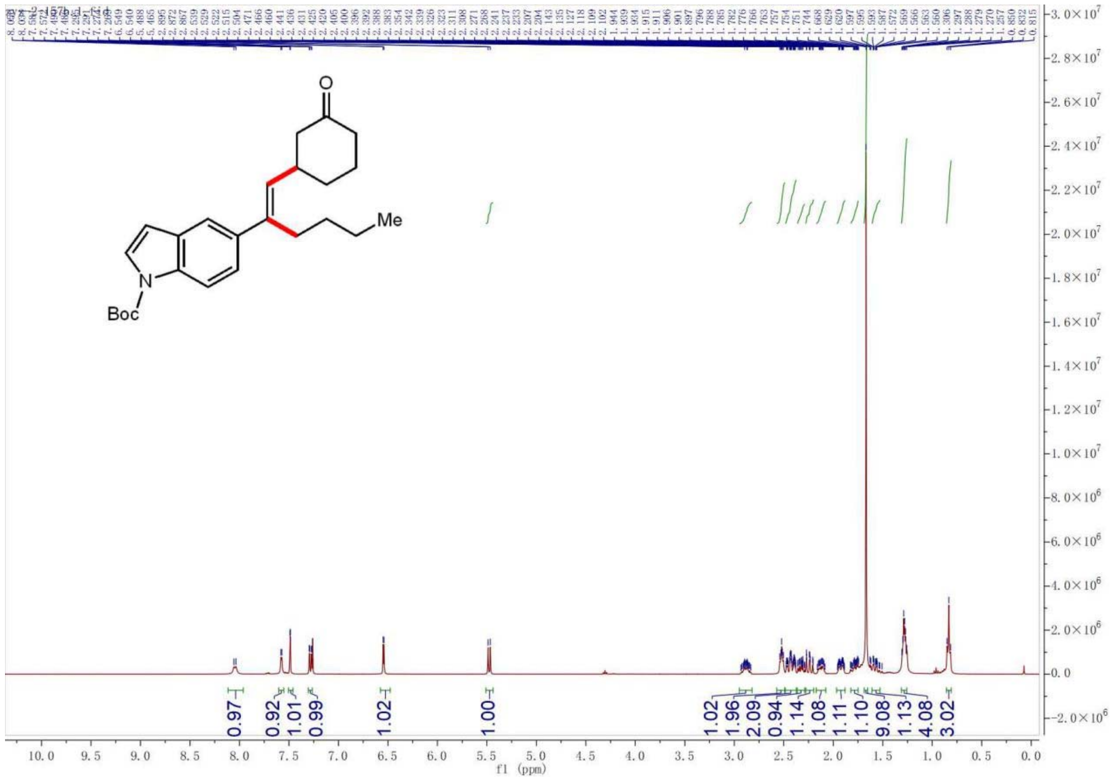

Supplementary Figure 64. ^{1}H NMR (400 MHz, $CDCl_3$) of **5l**

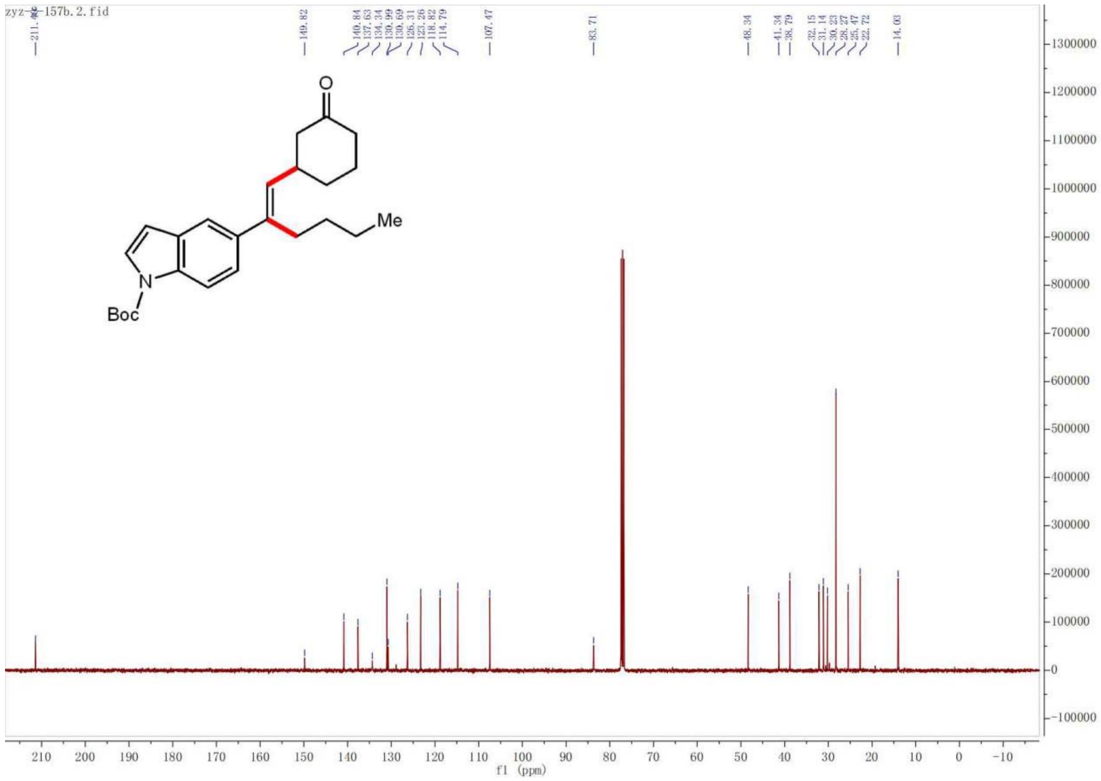

Supplementary Figure 65. ^{13}C NMR (101 MHz, $CDCl_3$) of **5l**

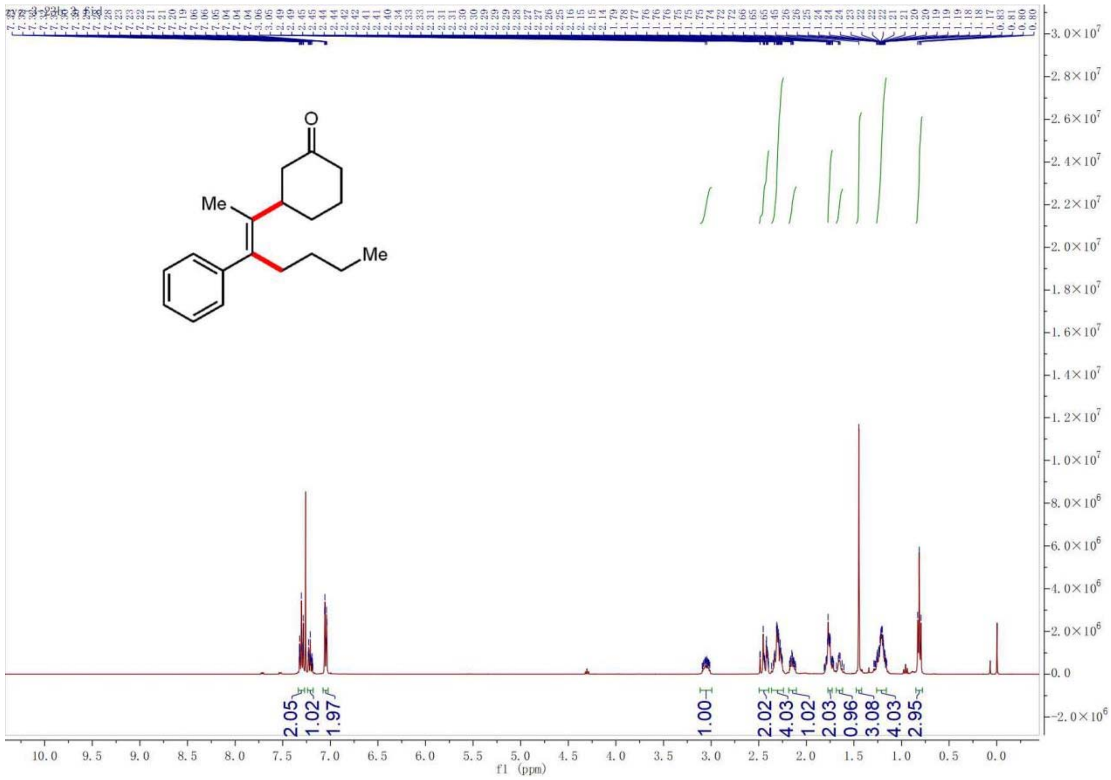

Supplementary Figure 66. ^{1}H NMR (400 MHz, $CDCl_3$) of **6a**

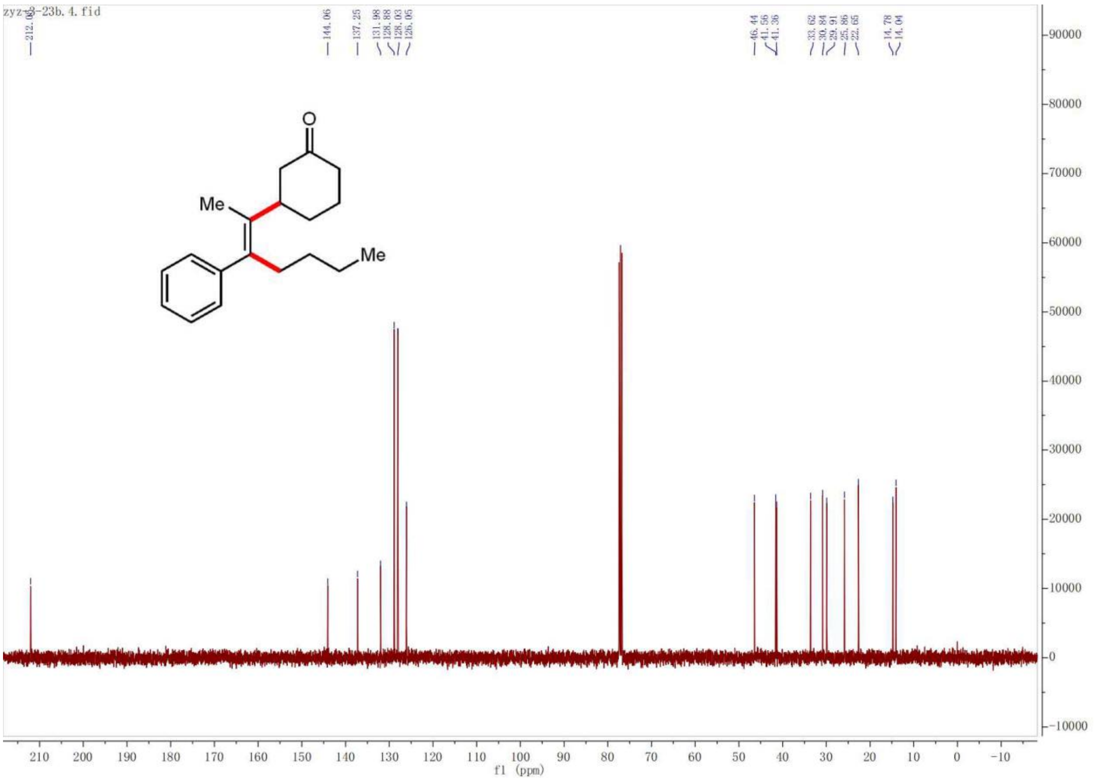

Supplementary Figure 67. ^{13}C NMR (101 MHz, $CDCl_3$) of **6a**

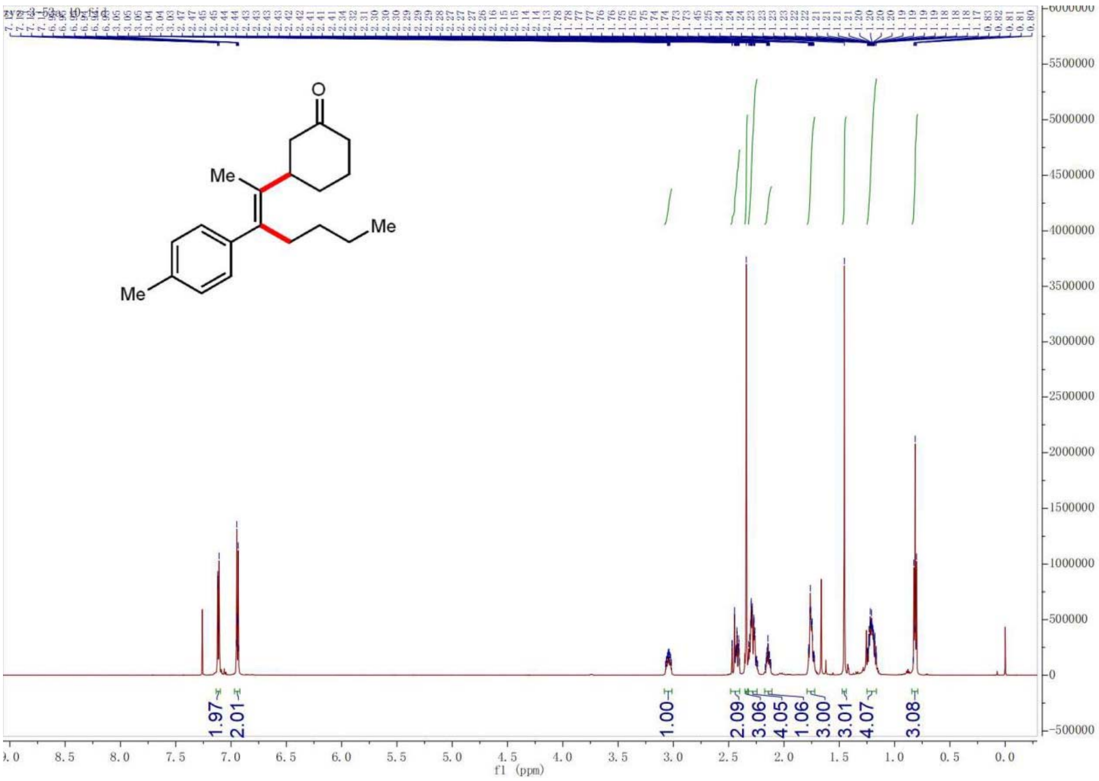

Supplementary Figure 68. ^{1}H NMR (600 MHz, $CDCl_3$) of **6b**

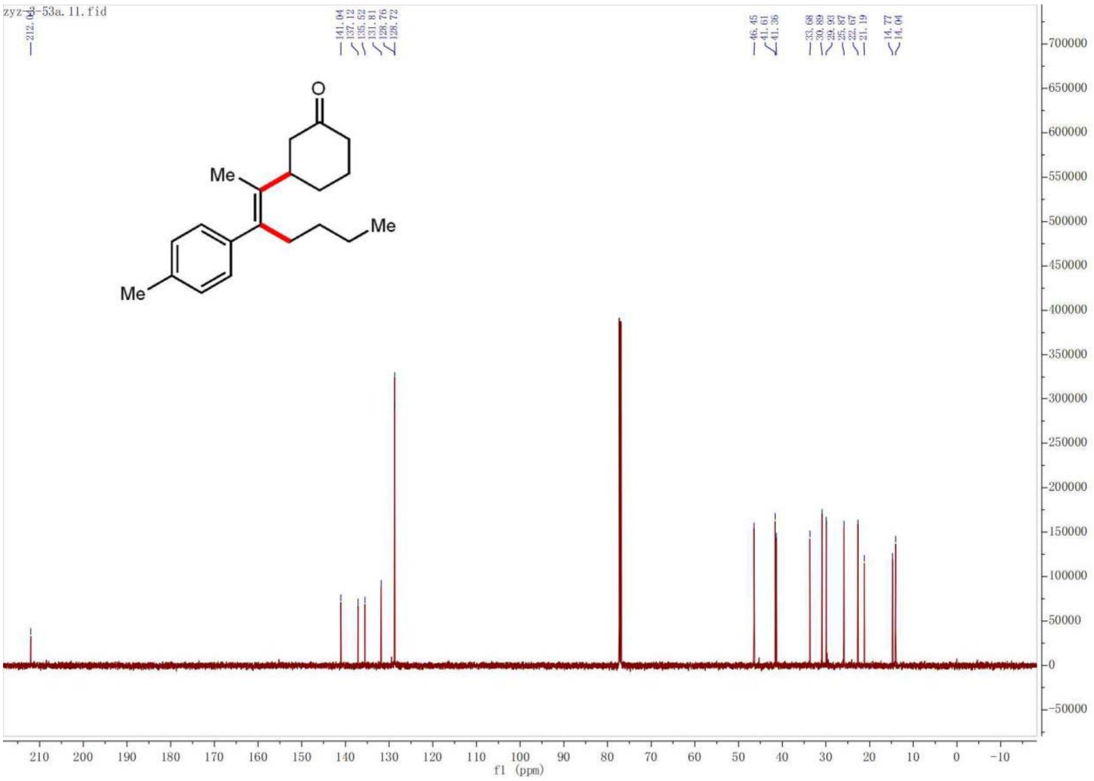

Supplementary Figure 69. ^{13}C NMR (151 MHz, $CDCl_3$) of **6b**

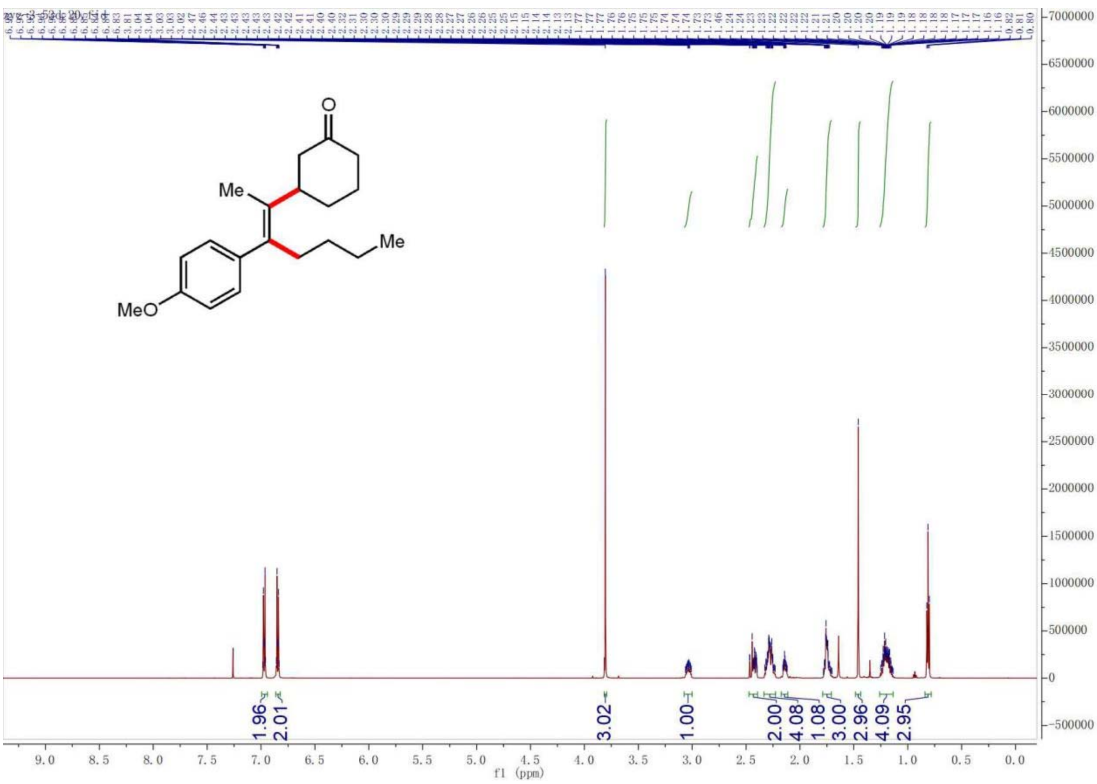

Supplementary Figure 70. ^{1}H NMR (600 MHz, $CDCl_3$) of **6c**

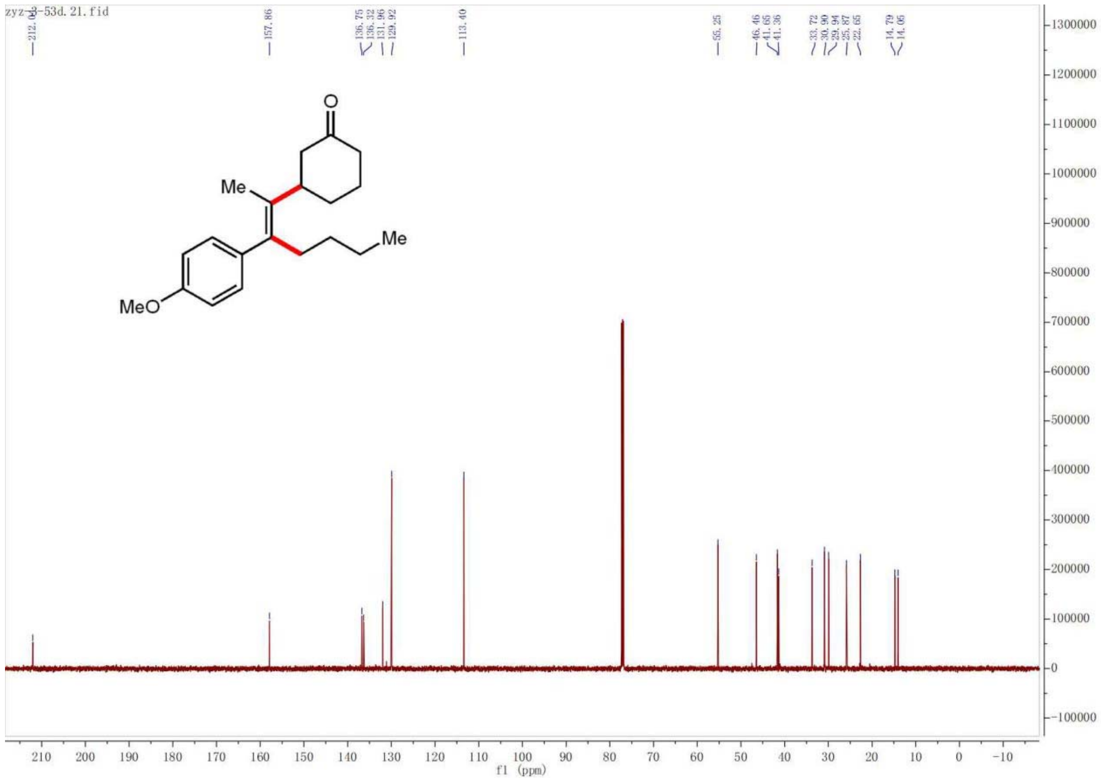

Supplementary Figure 71. ^{13}C NMR (151 MHz, $CDCl_3$) of **6c**

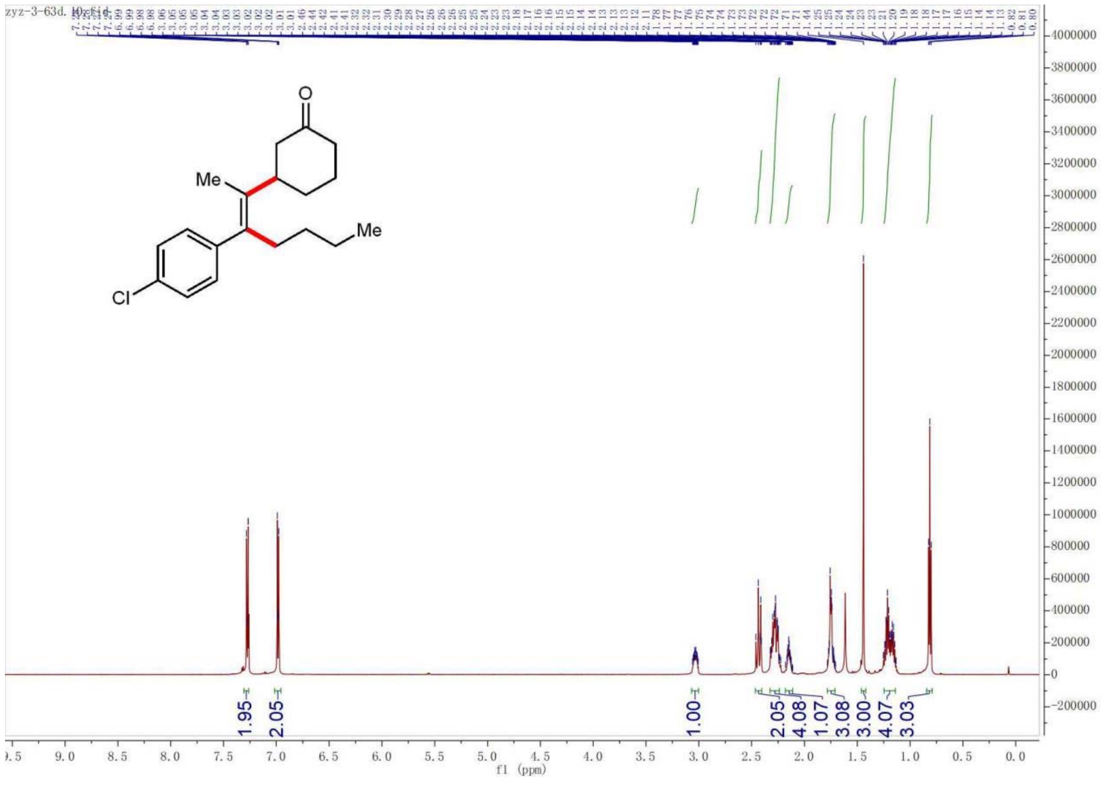

Supplementary Figure 72. ^{1}H NMR (600 MHz, $CDCl_3$) of **6d**

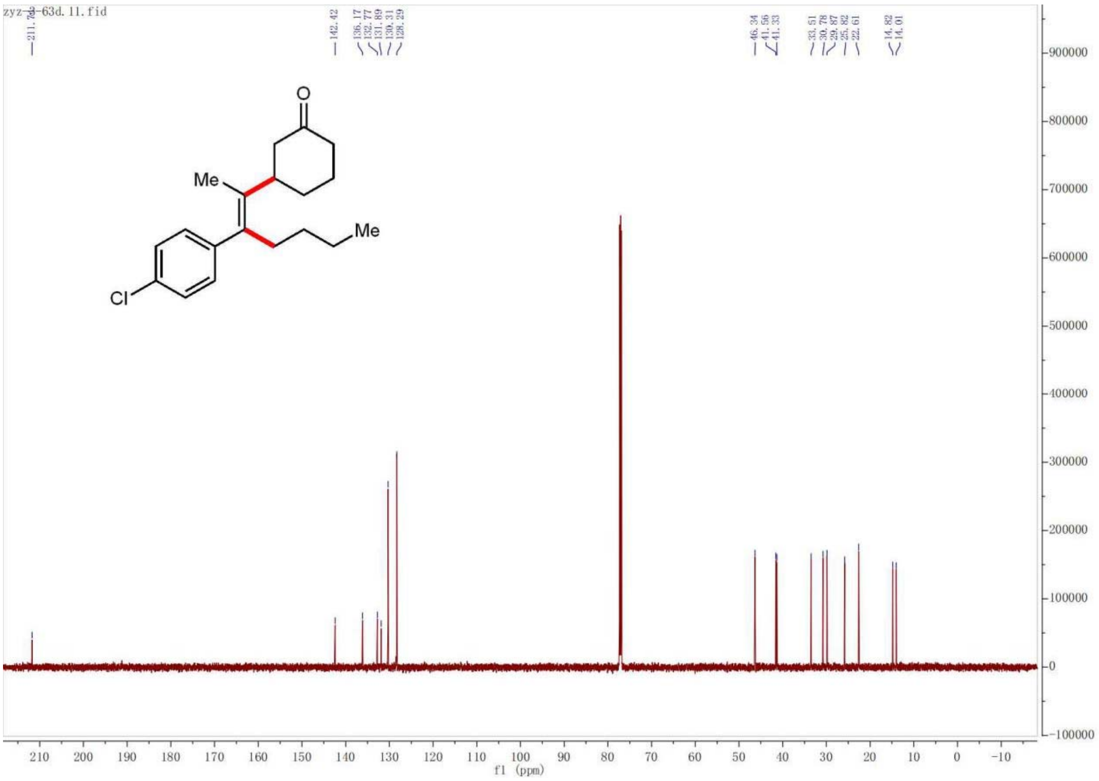

Supplementary Figure 73. ^{13}C NMR (151 MHz, $CDCl_3$) of **6d**

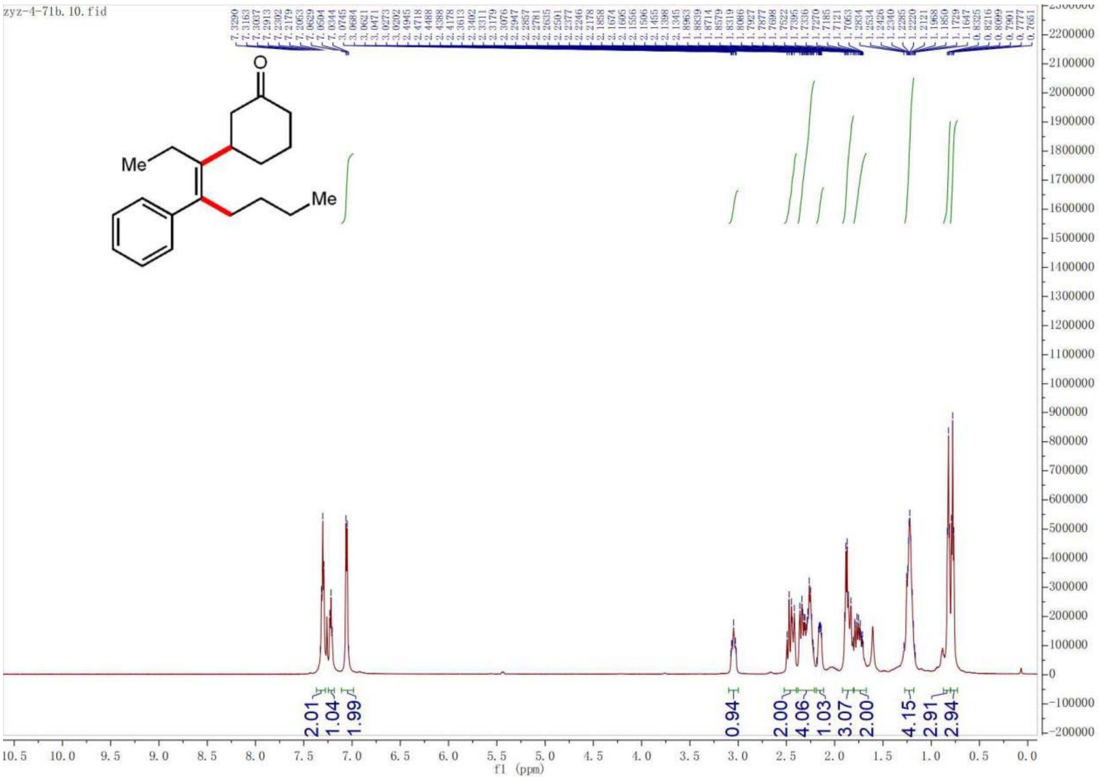

Supplementary Figure 74. ^{1}H NMR (600 MHz, $CDCl_3$) of **6e**

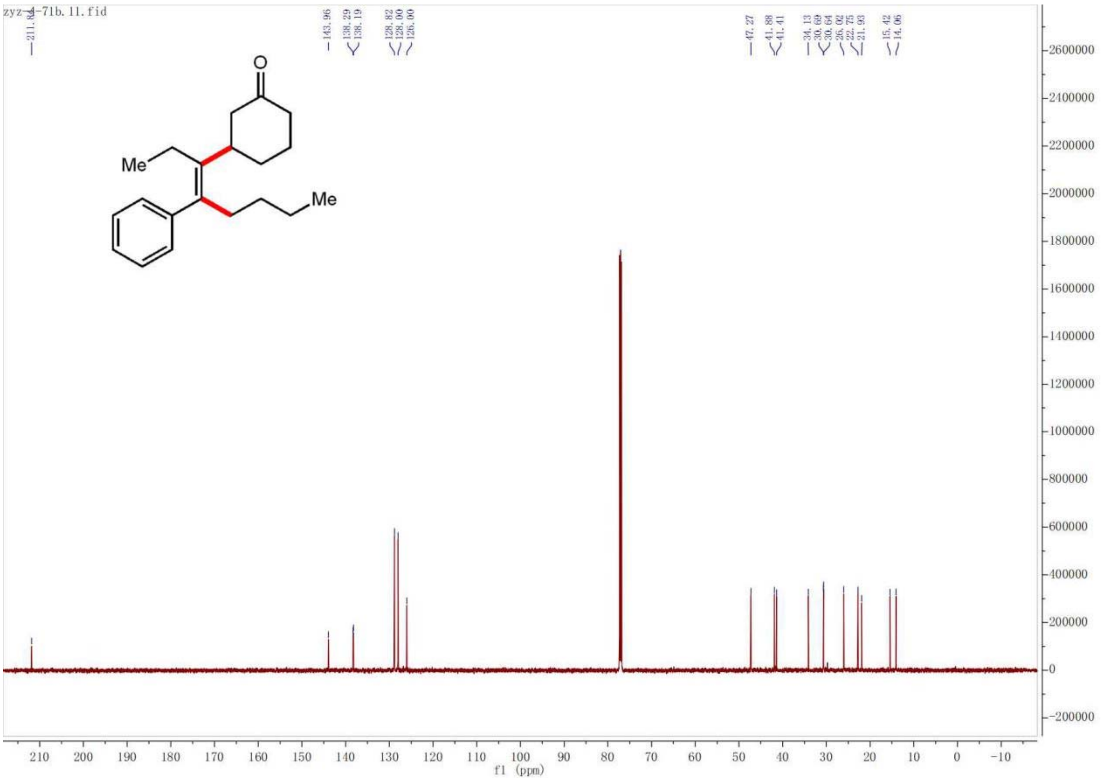

Supplementary Figure 75. ^{13}C NMR (151 MHz, $CDCl_3$) of **6e**

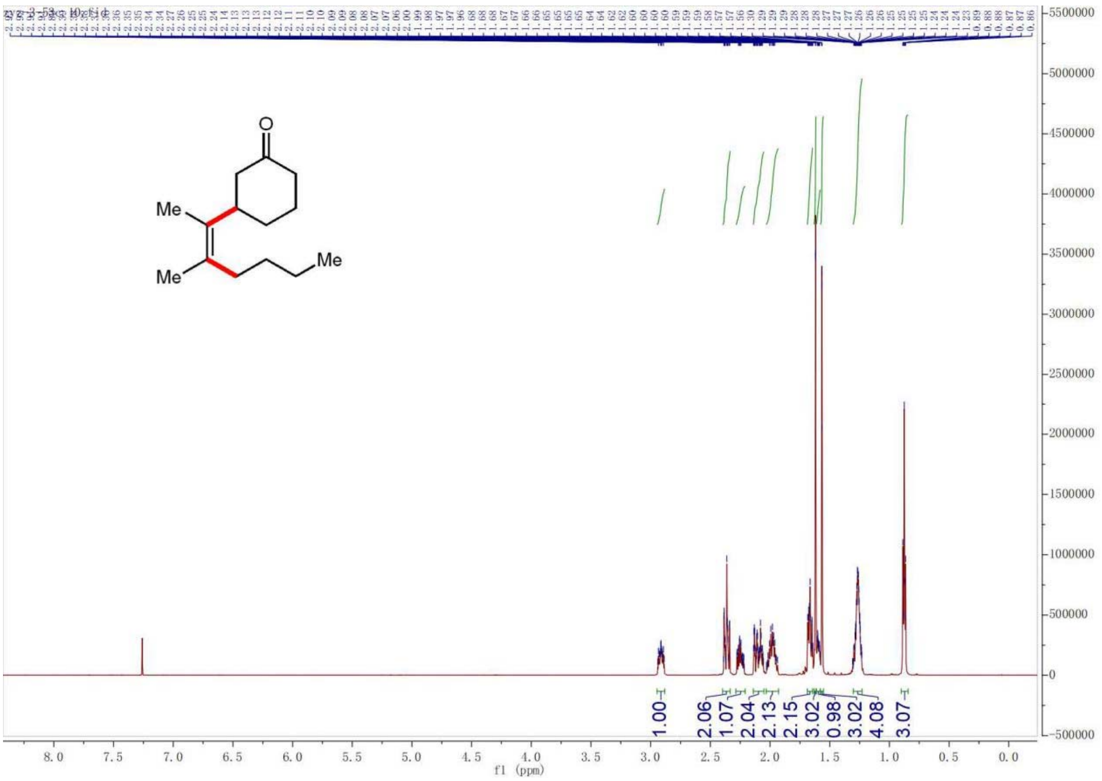

Supplementary Figure 76. ^{1}H NMR (600 MHz, $CDCl_3$) of **6f**

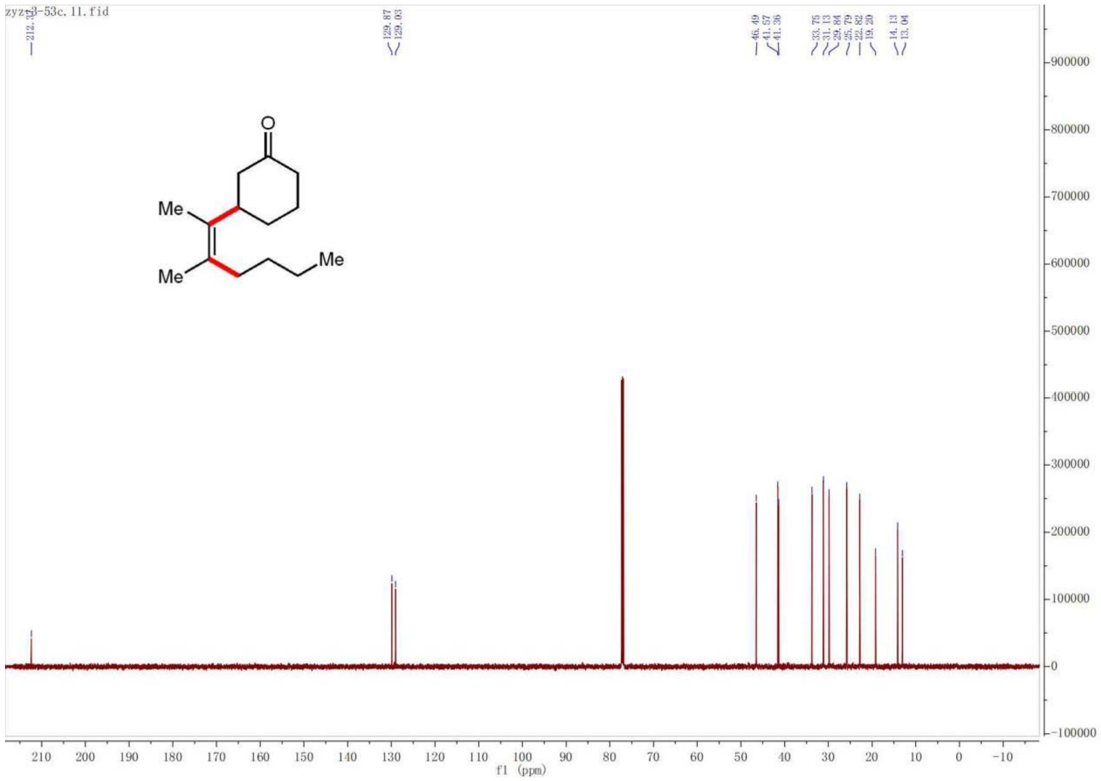

Supplementary Figure 77. ^{13}C NMR (151 MHz, $CDCl_3$) of **6f**

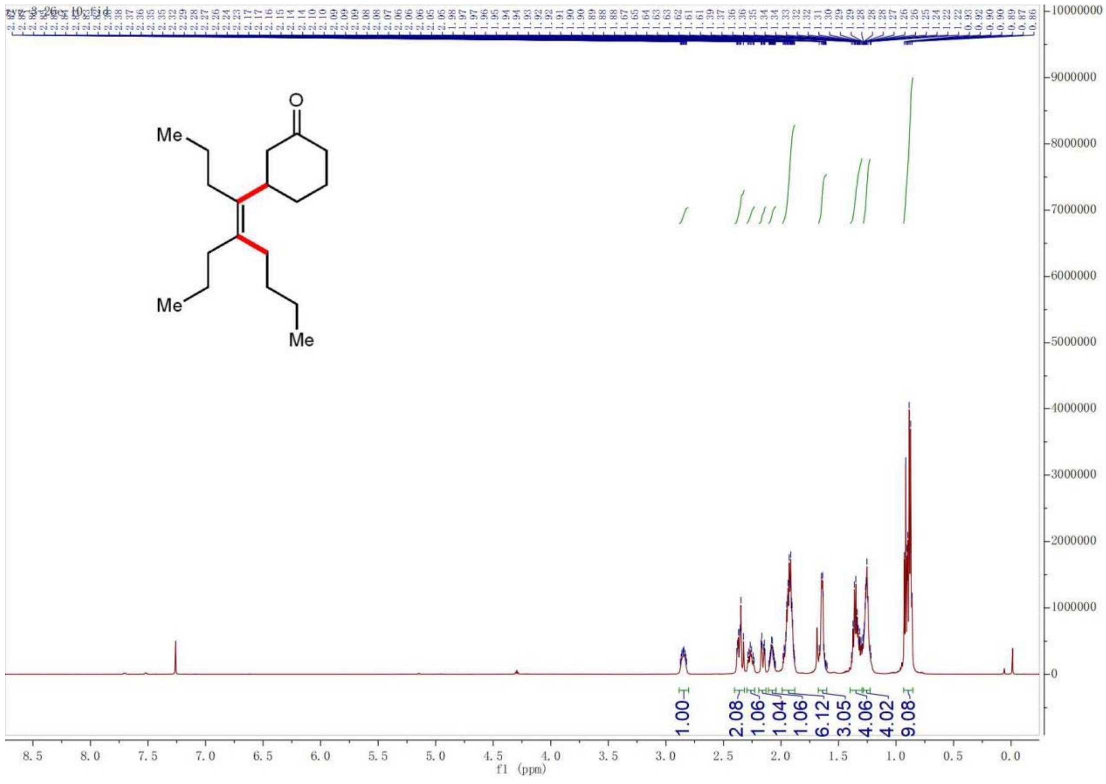

Supplementary Figure 78. ^{1}H NMR (600 MHz, $CDCl_3$) of **6g**

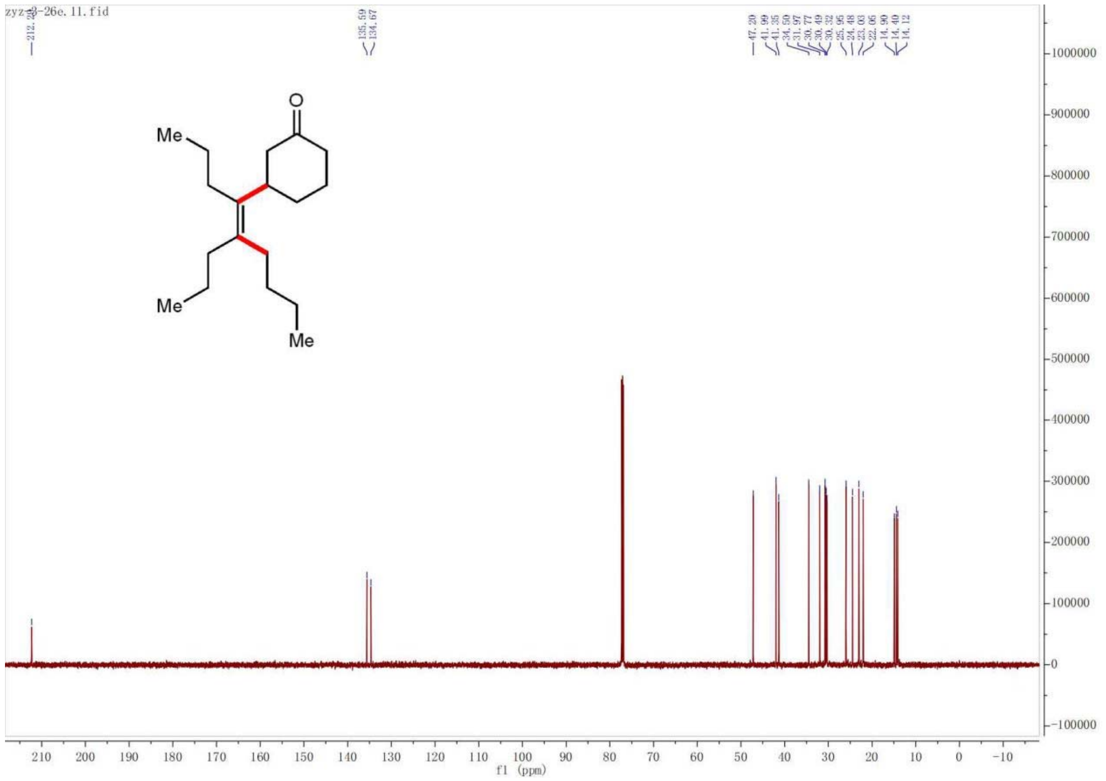

Supplementary Figure 79. ^{13}C NMR (151 MHz, $CDCl_3$) of **6g**

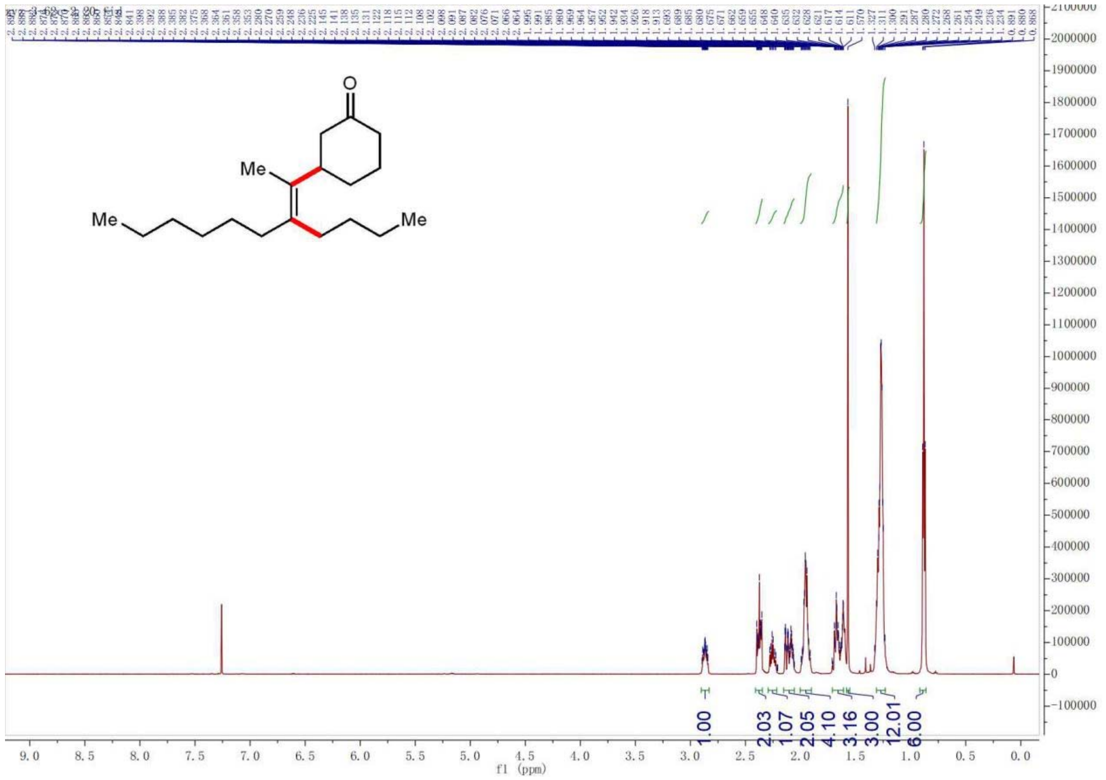

Supplementary Figure 80. ^{1}H NMR (600 MHz, $CDCl_3$) of **6h**

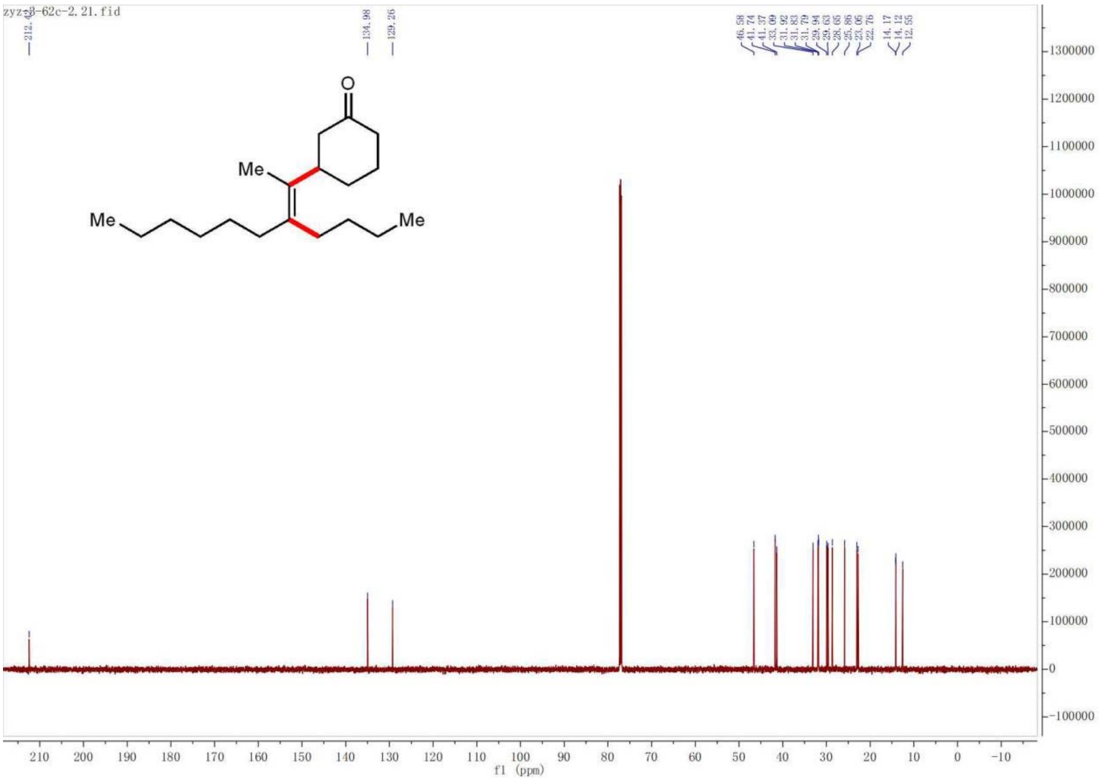

Supplementary Figure 81. ^{13}C NMR (151 MHz, $CDCl_3$) of **6h**

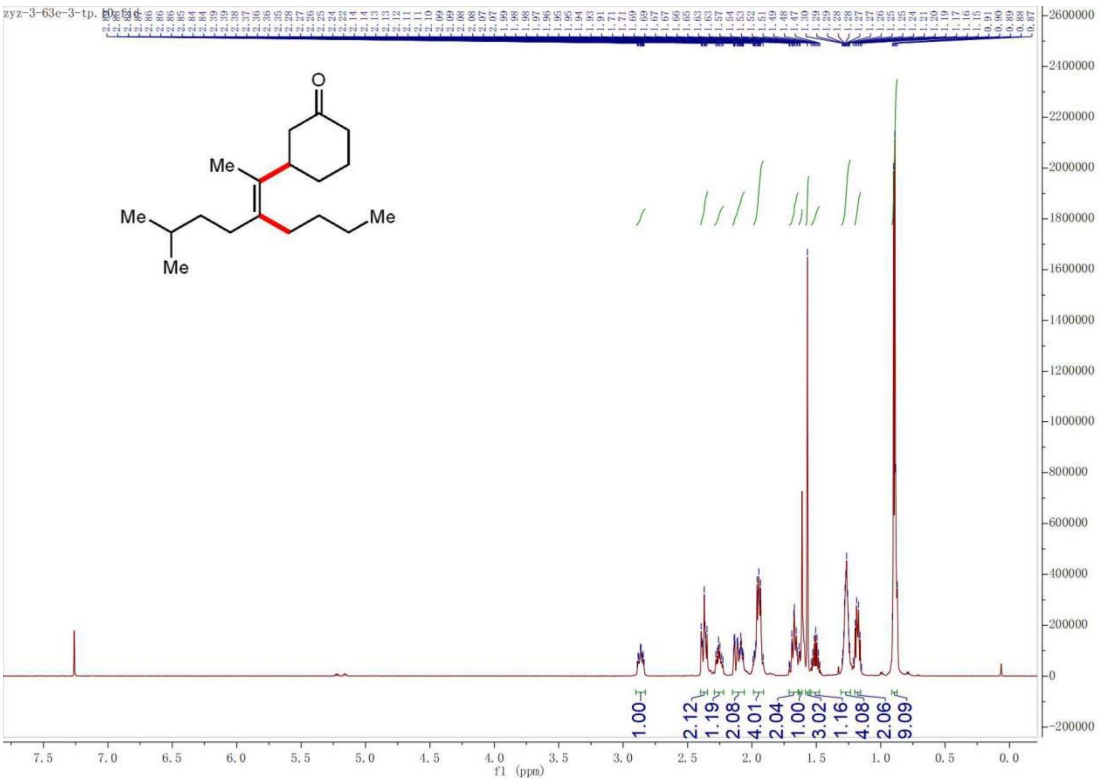

Supplementary Figure 82. ^{1}H NMR (600 MHz, $CDCl_3$) of **6i**

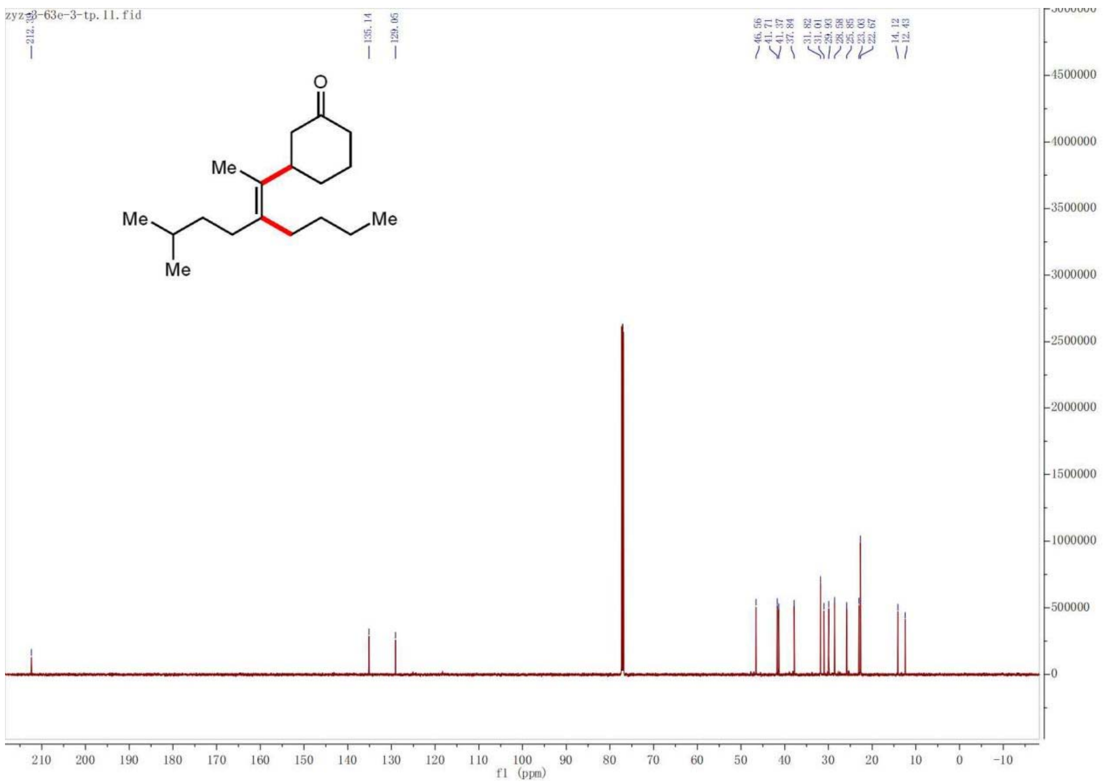

Supplementary Figure 83. ^{13}C NMR (151 MHz, $CDCl_3$) of **6i**

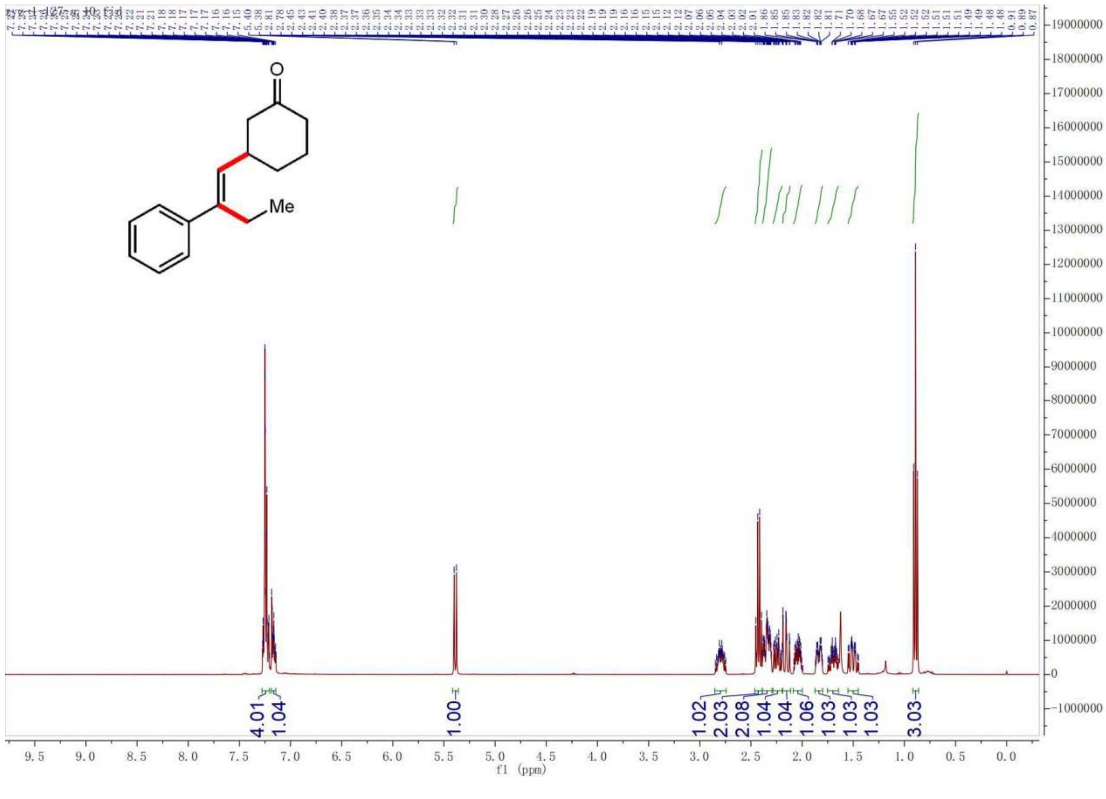

Supplementary Figure 84. ^{1}H NMR (400 MHz, $CDCl_3$) of **7a**

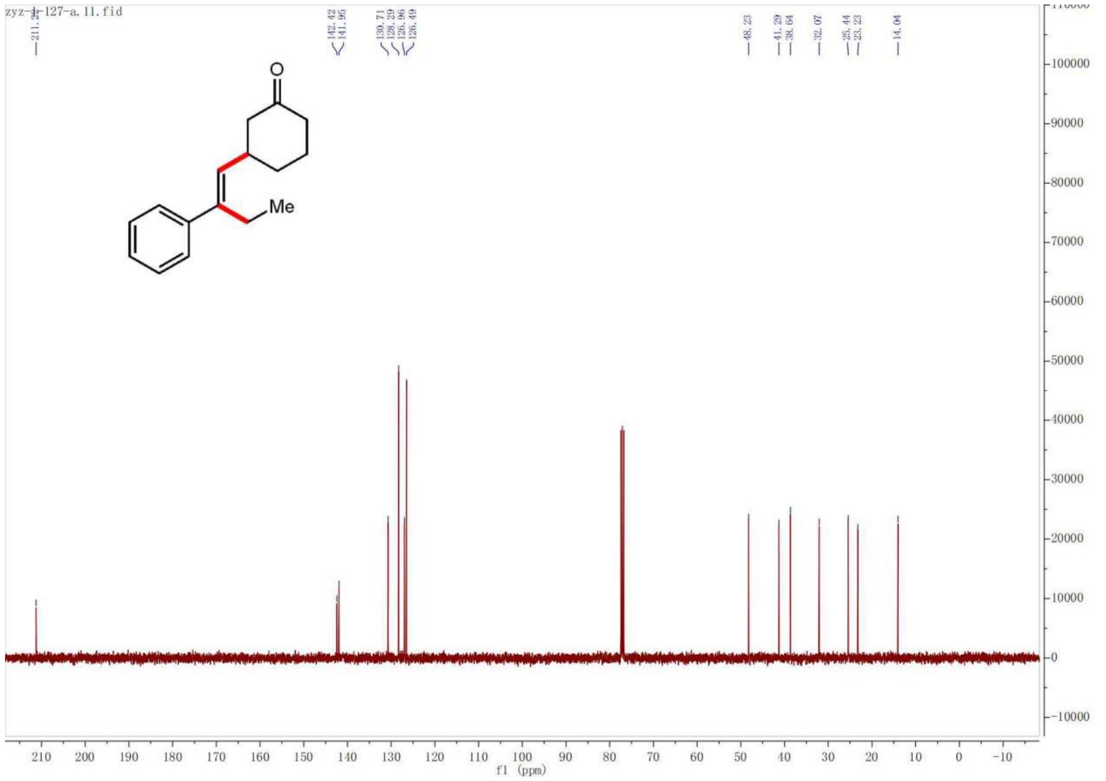

Supplementary Figure 85. ^{13}C NMR (101 MHz, $CDCl_3$) of **7a**

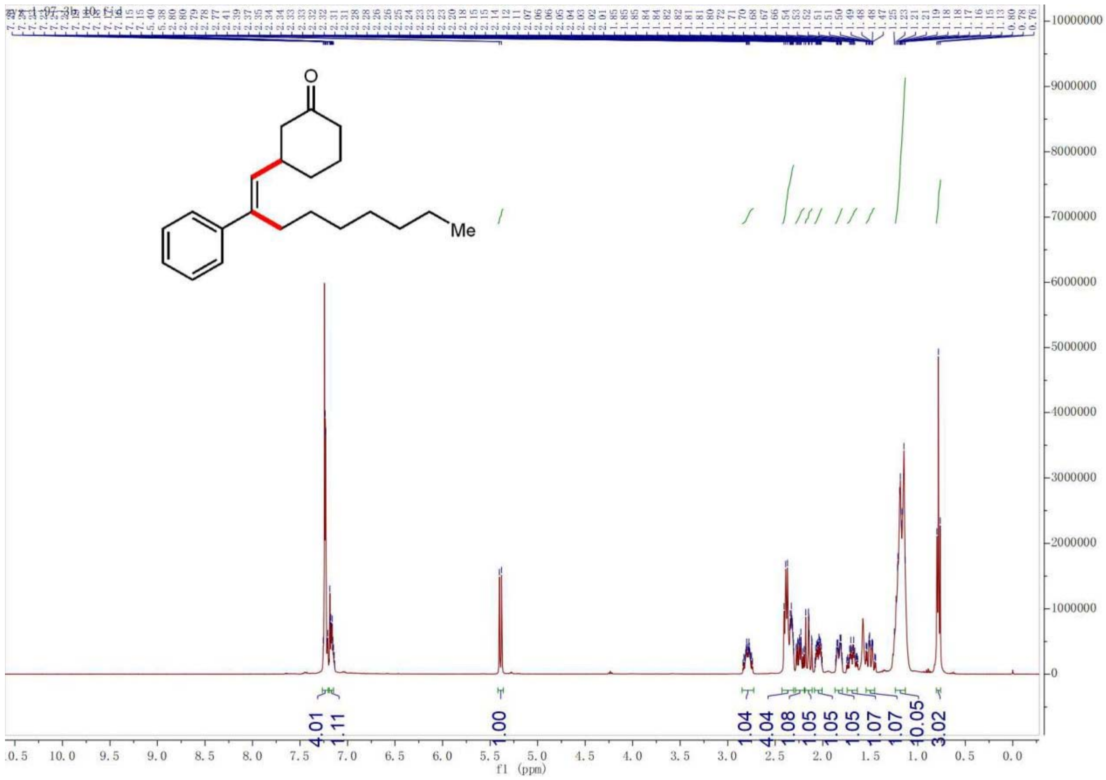

Supplementary Figure 86. ^{1}H NMR (400 MHz, $CDCl_3$) of **7b**

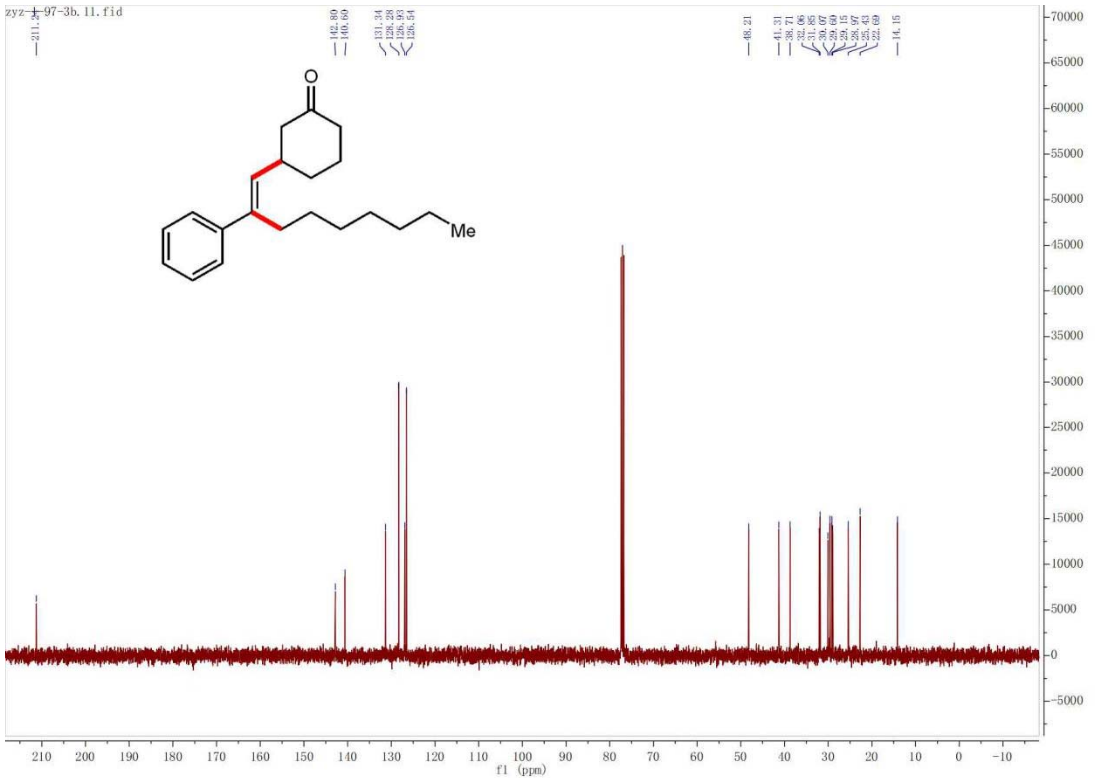

Supplementary Figure 87. ^{13}C NMR (101 MHz, $CDCl_3$) of **7b**

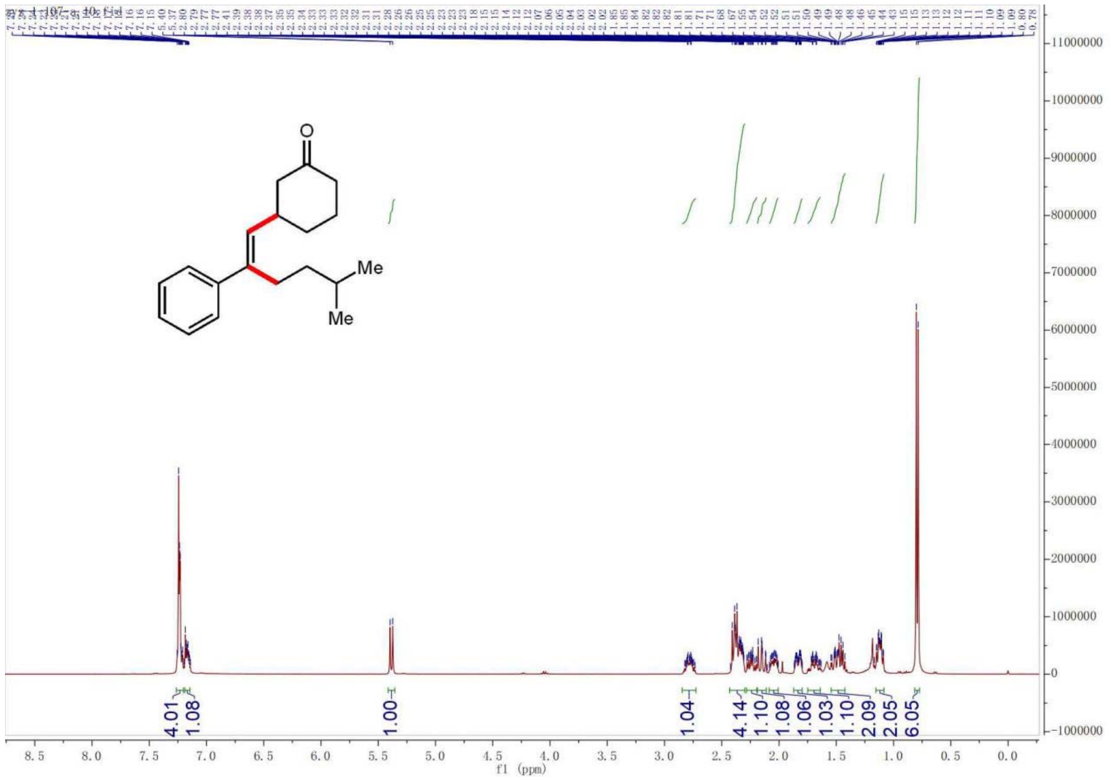

Supplementary Figure 88. ^{1}H NMR (400 MHz, $CDCl_3$) of **7c**

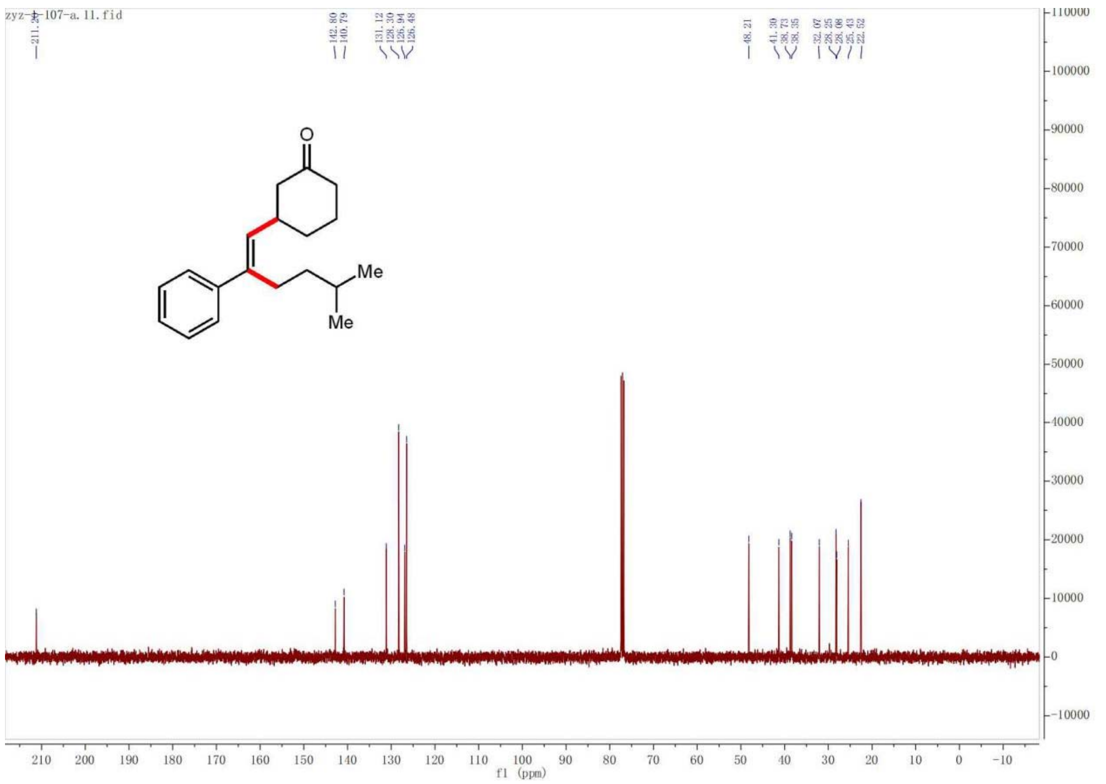

Supplementary Figure 89. ^{13}C NMR (101 MHz, $CDCl_3$) of **7c**

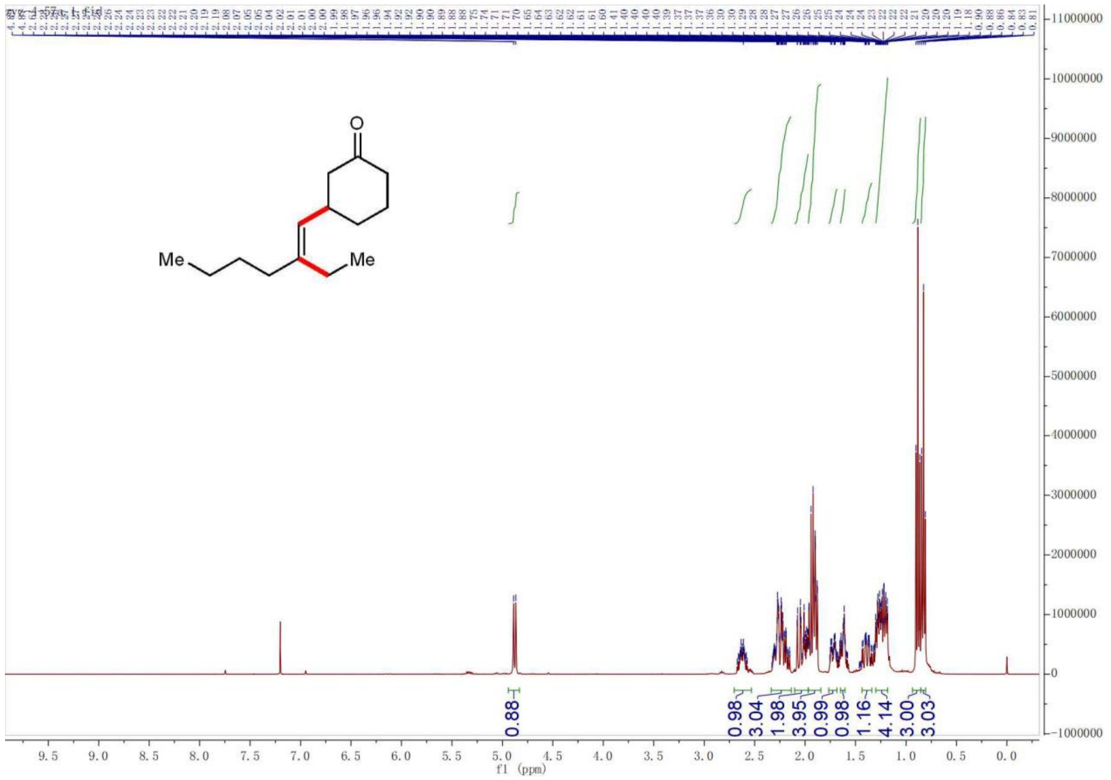

Supplementary Figure 90. ^{1}H NMR (400 MHz, $CDCl_3$) of **7d**

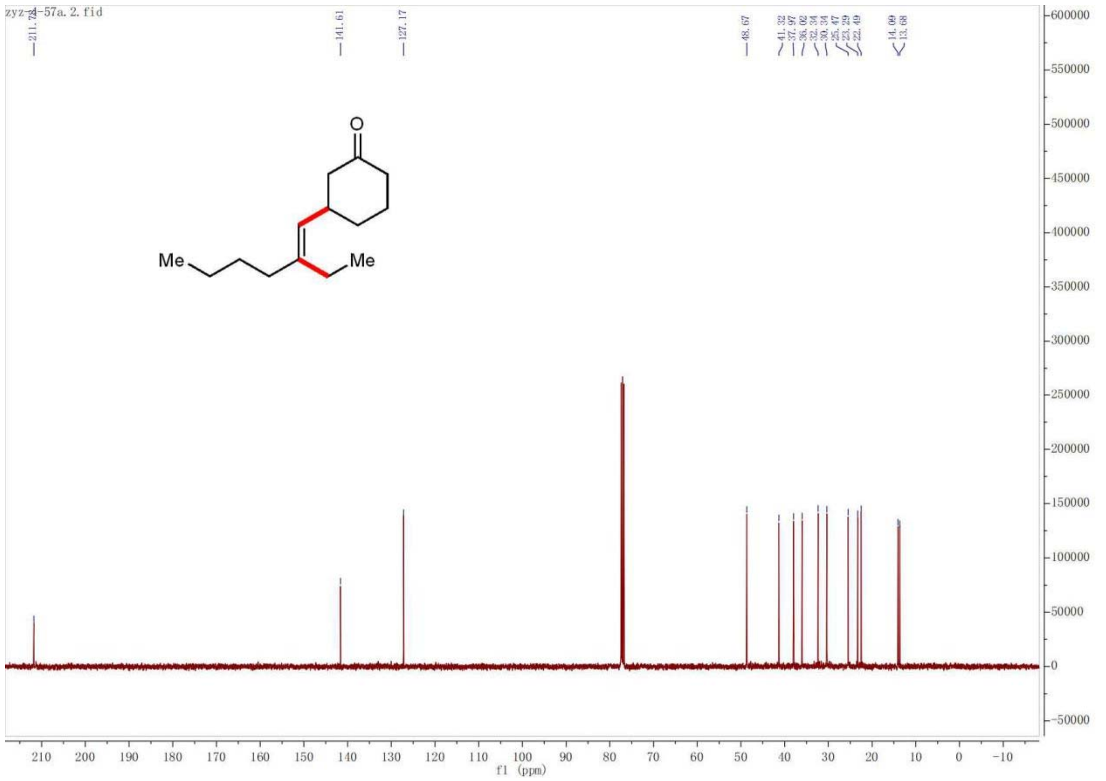

Supplementary Figure 91. ^{13}C NMR (101 MHz, $CDCl_3$) of **7d**

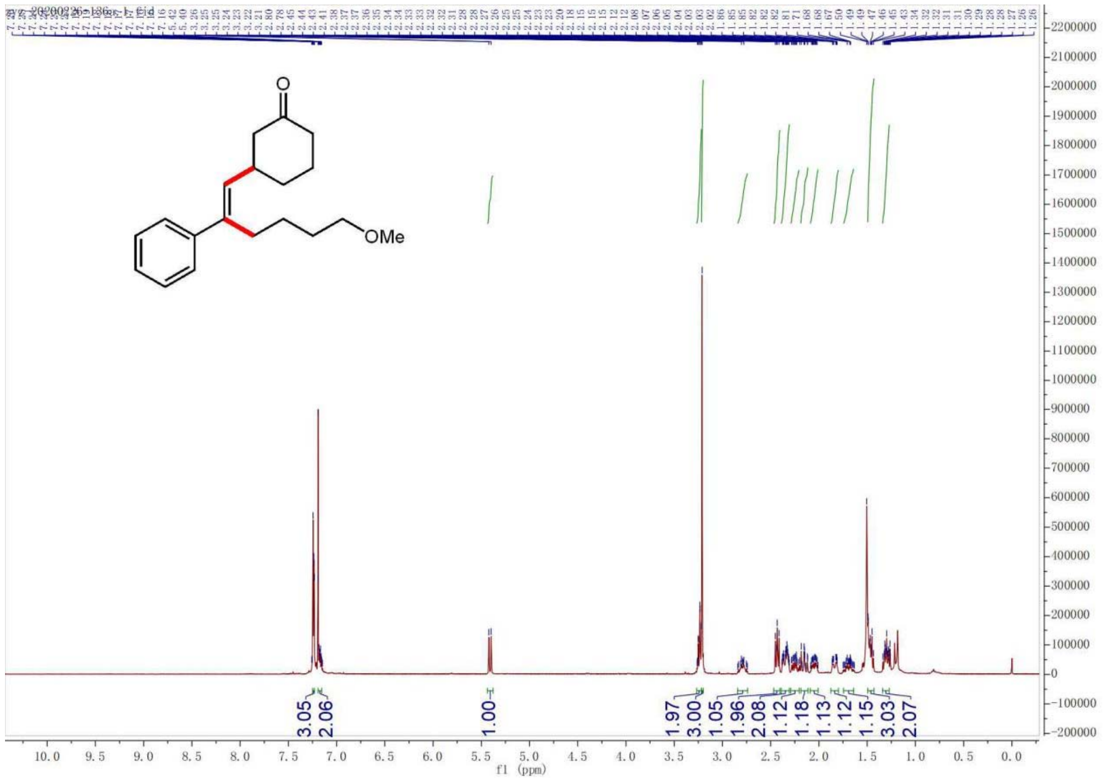

Supplementary Figure 92. ^{1}H NMR (400 MHz, $CDCl_3$) of **7e**

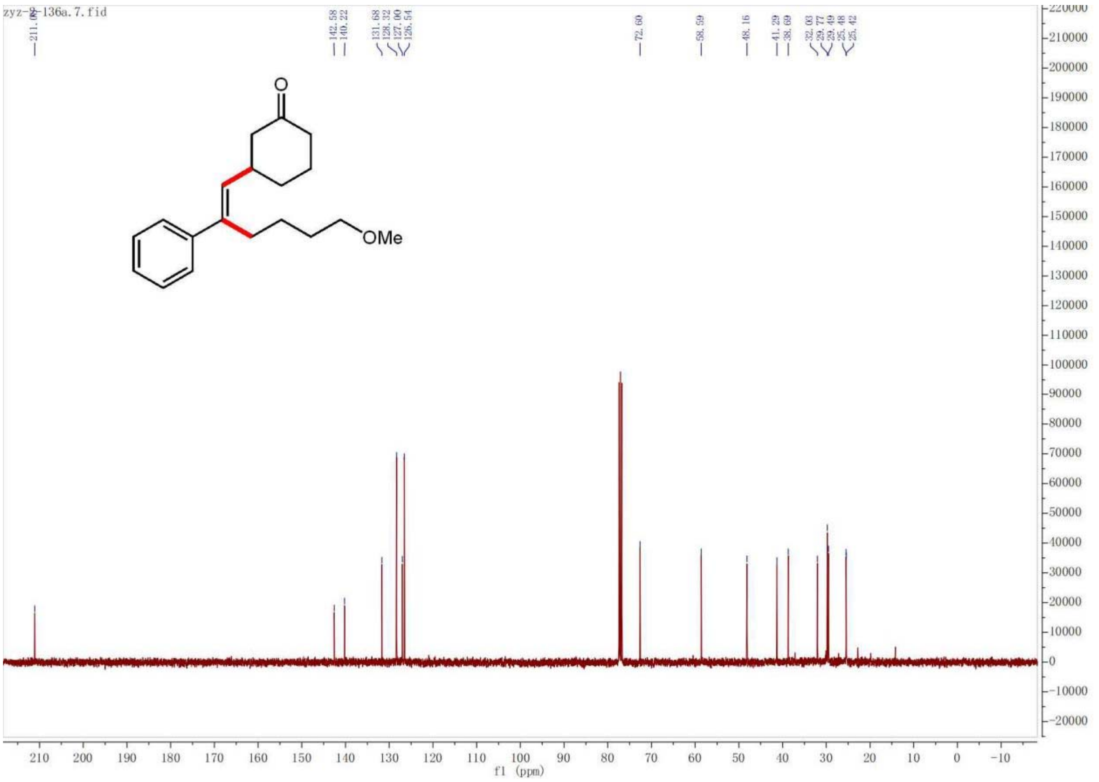

Supplementary Figure 93. ^{13}C NMR (101 MHz, $CDCl_3$) of **7e**

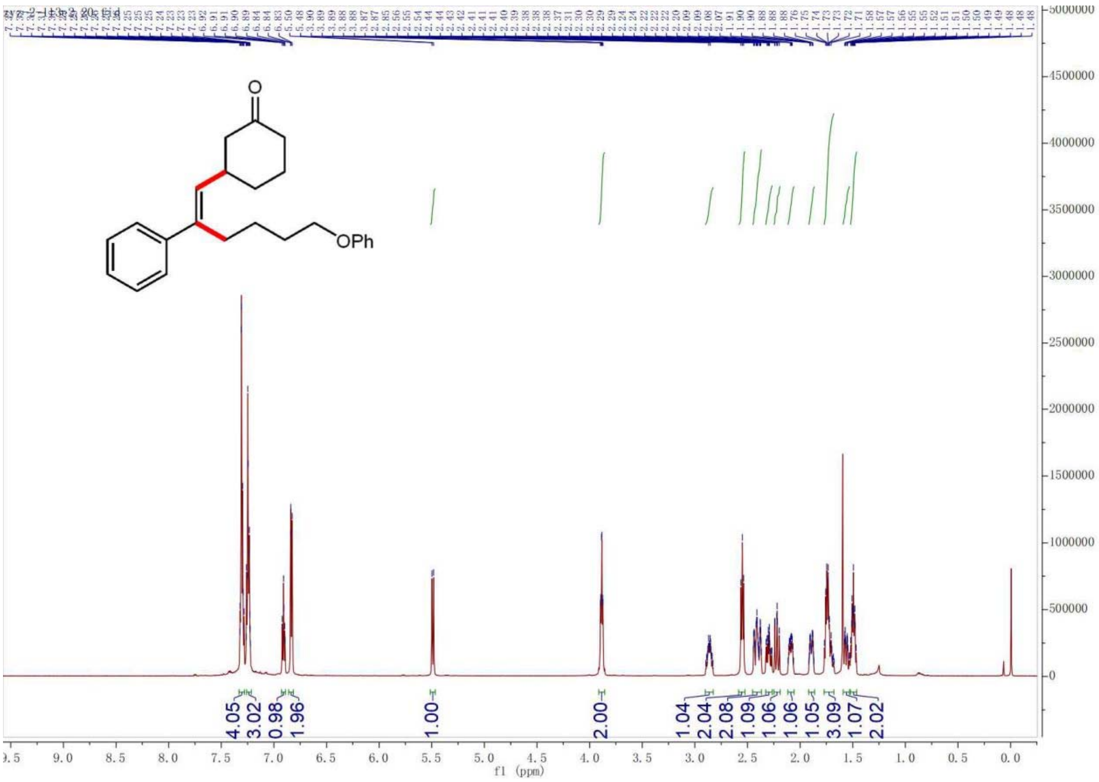

Supplementary Figure 94. ^{1}H NMR (600 MHz, $CDCl_3$) of **7f**

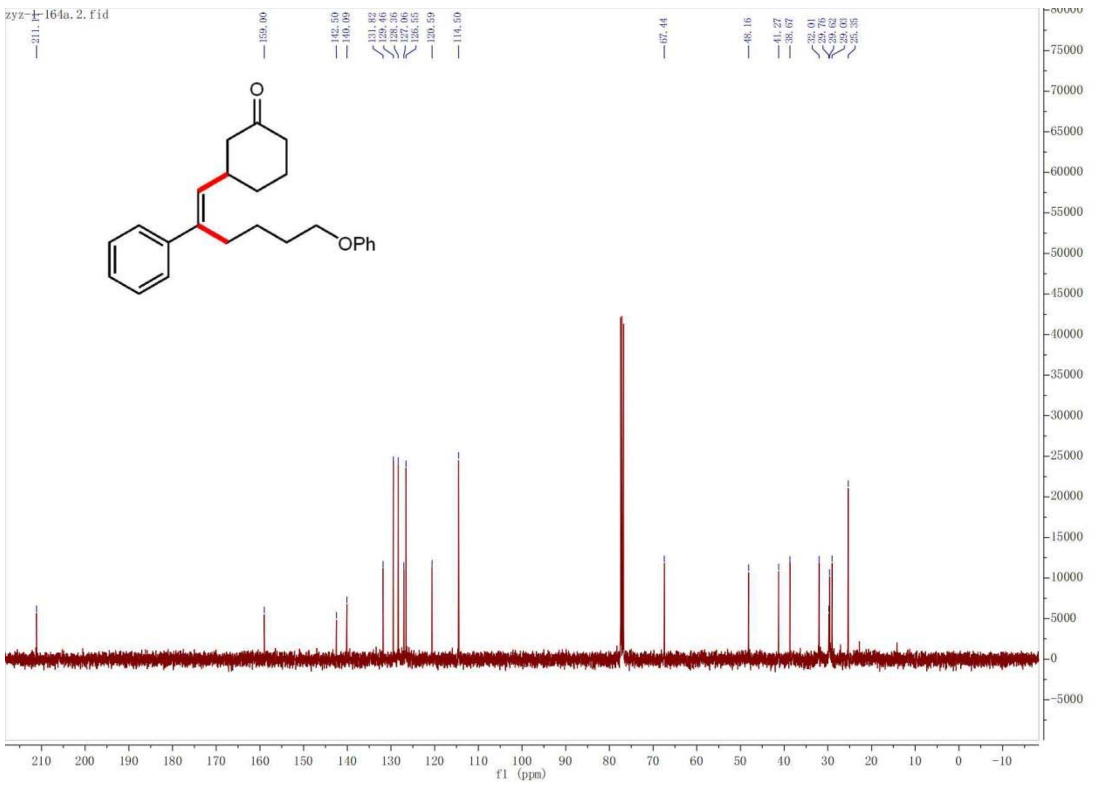

Supplementary Figure 95. ^{13}C NMR (151 MHz, $CDCl_3$) of **7f**

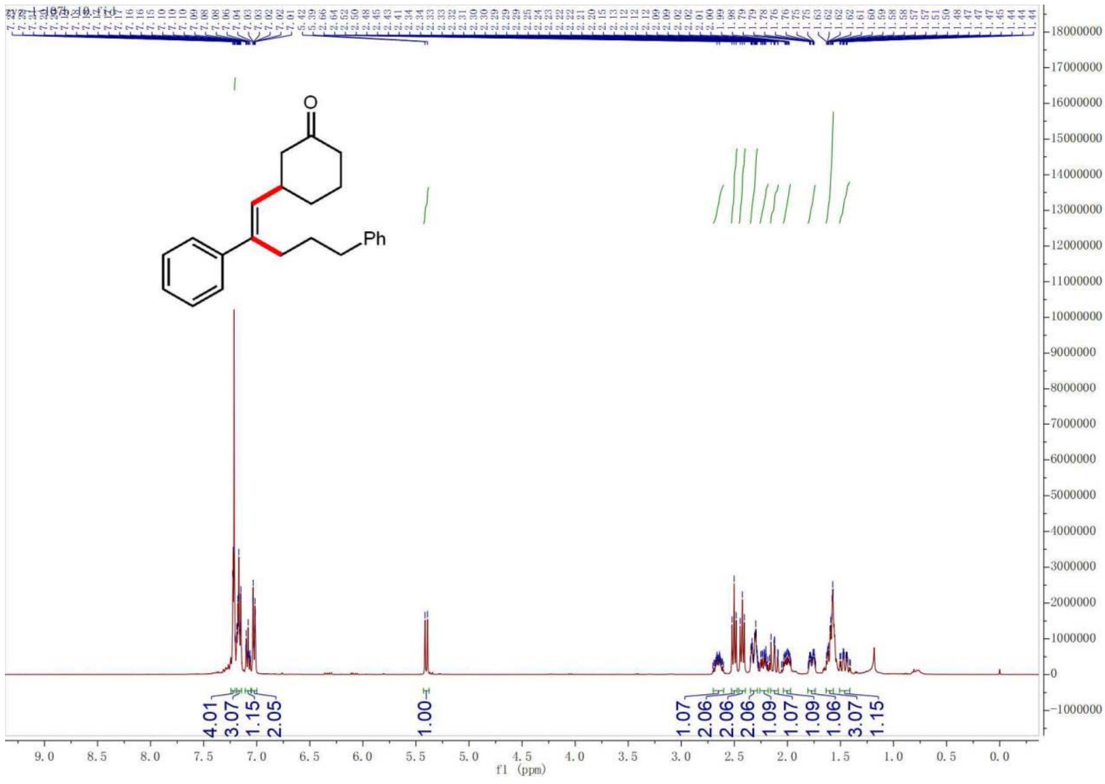

Supplementary Figure 96. ^{1}H NMR (400 MHz, $CDCl_3$) of **7g**

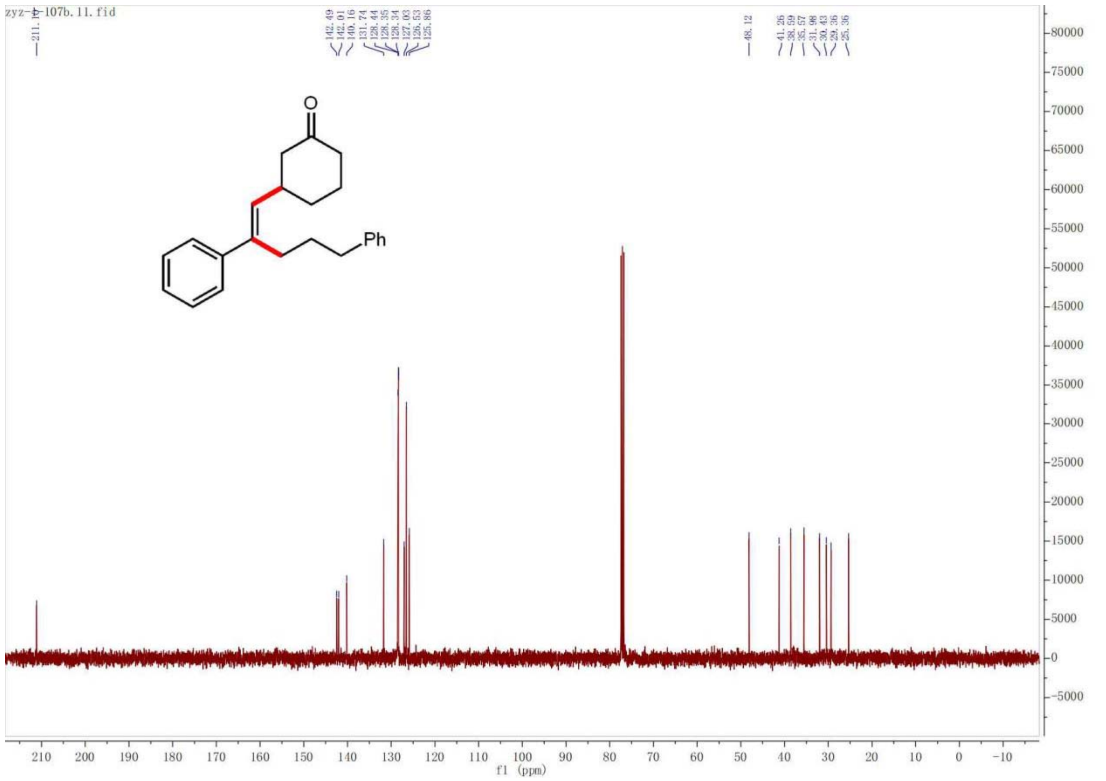

Supplementary Figure 97. ^{13}C NMR (101 MHz, $CDCl_3$) of **7g**

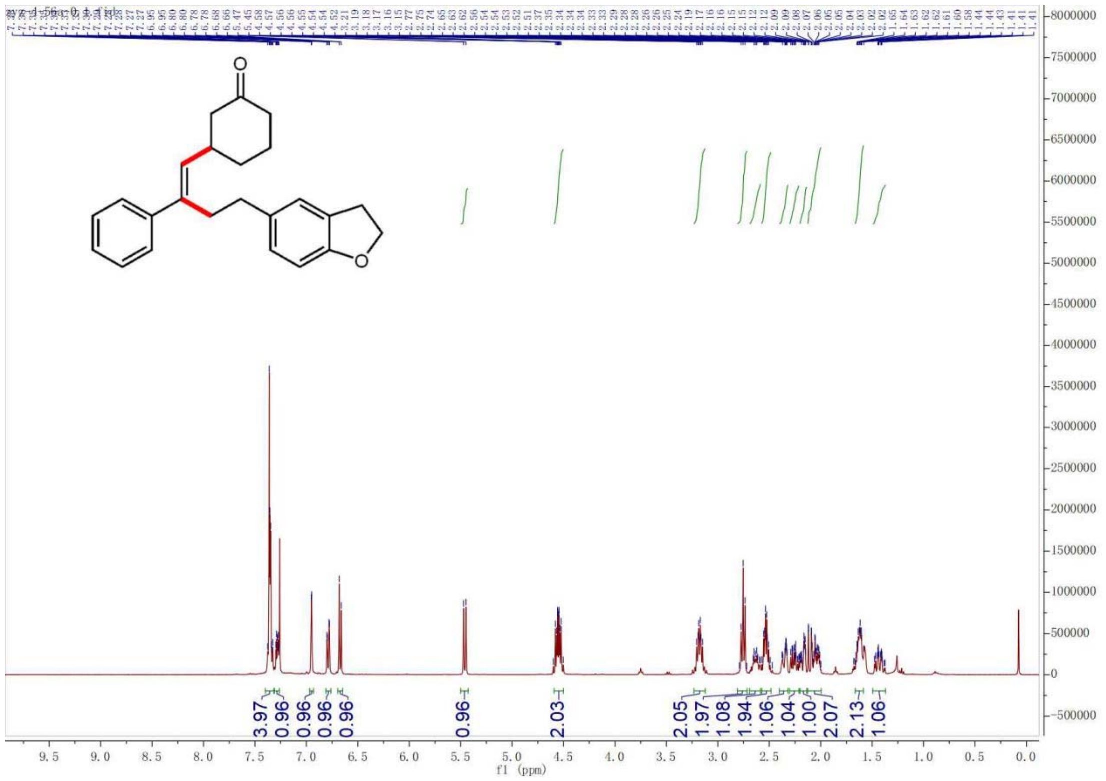

Supplementary Figure 98. ^{1}H NMR (400 MHz, $CDCl_3$) of **7h**

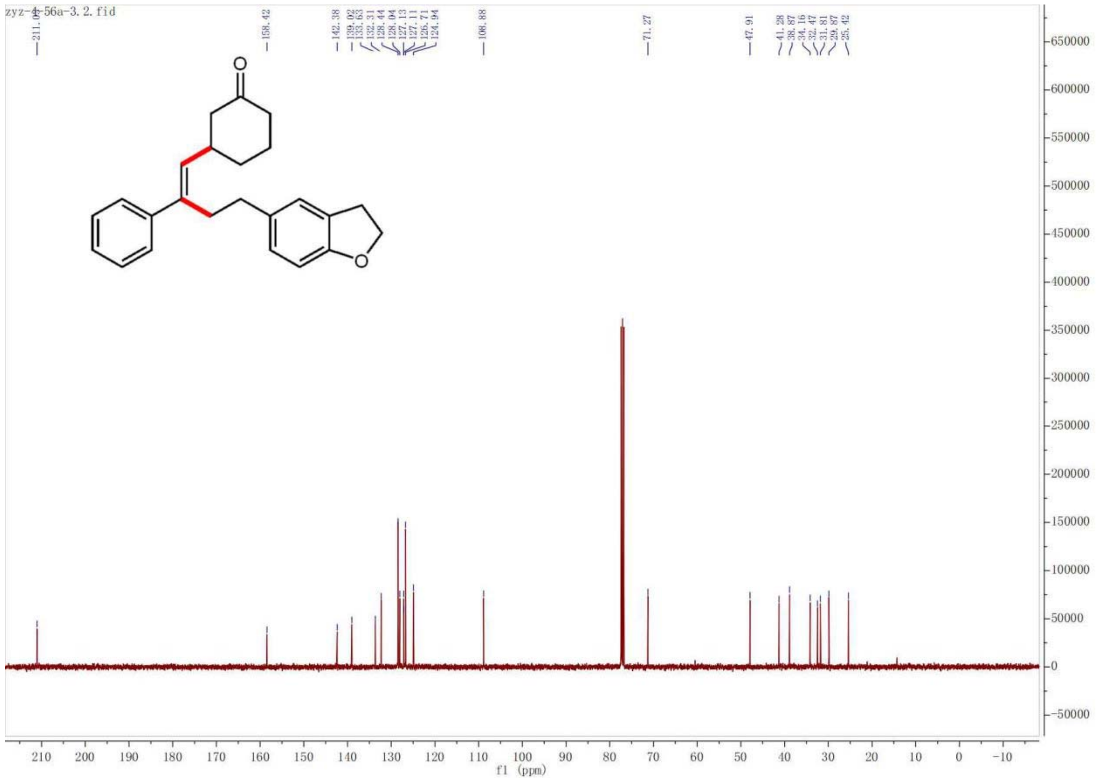

Supplementary Figure 99. ^{13}C NMR (101 MHz, $CDCl_3$) of **7h**

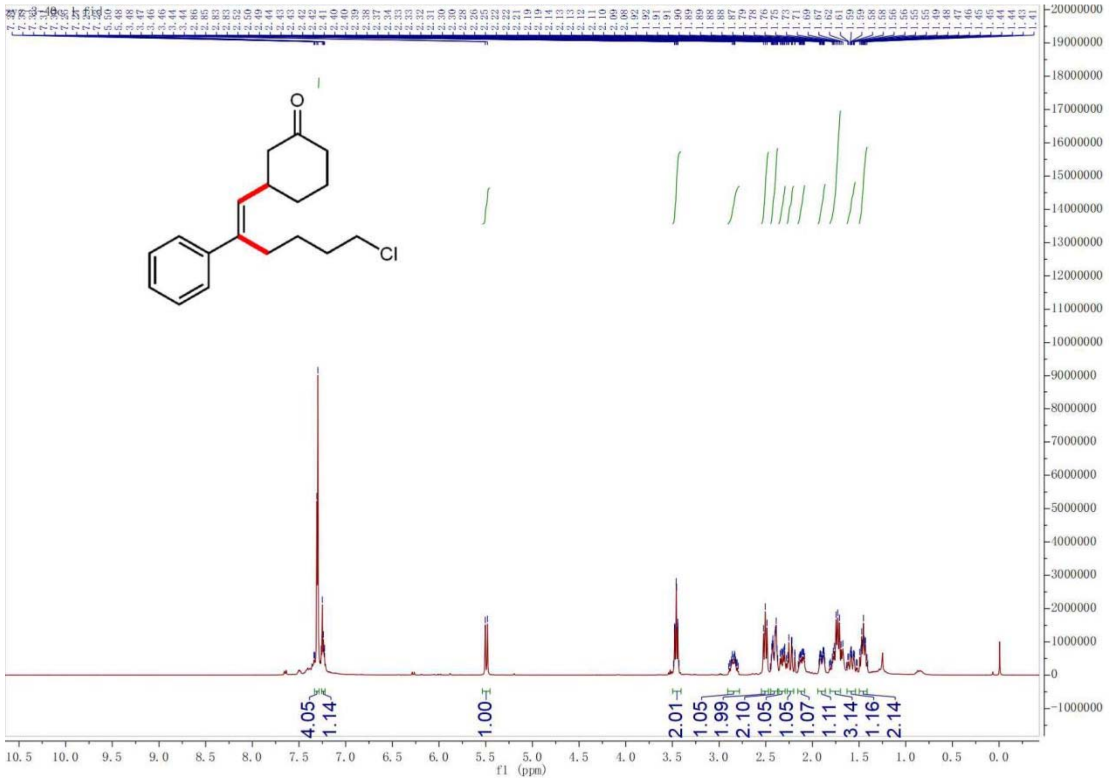

Supplementary Figure 100. ^{1}H NMR (400 MHz, $CDCl_3$) of **7i**

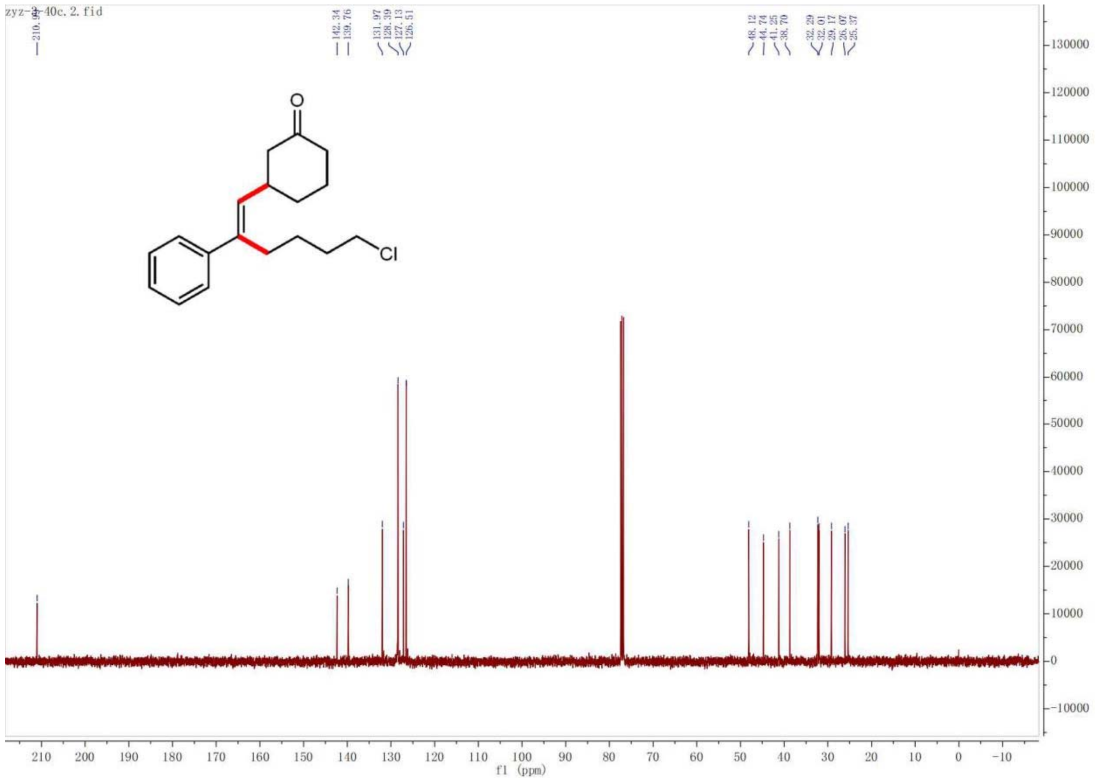

Supplementary Figure 101. ^{13}C NMR (101 MHz, $CDCl_3$) of **7i**

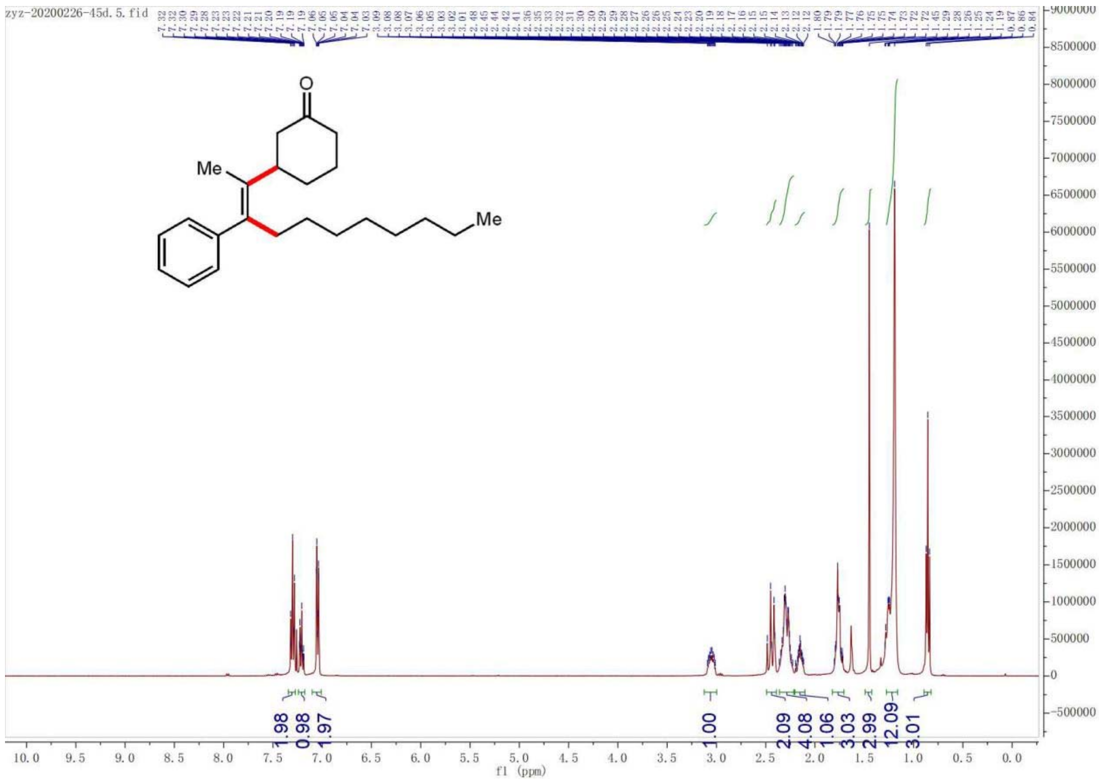

Supplementary Figure 102. ^{1}H NMR (400 MHz, $CDCl_3$) of **7j**

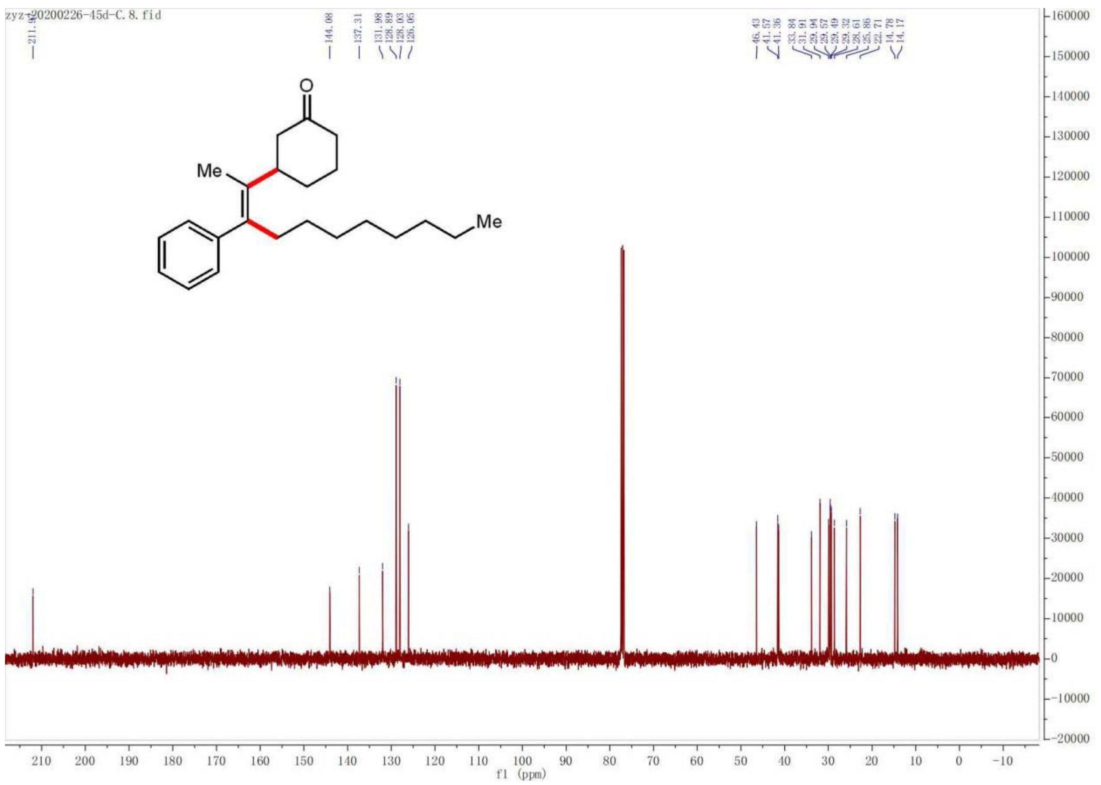

Supplementary Figure 103. ^{13}C NMR (101 MHz, $CDCl_3$) of **7j**

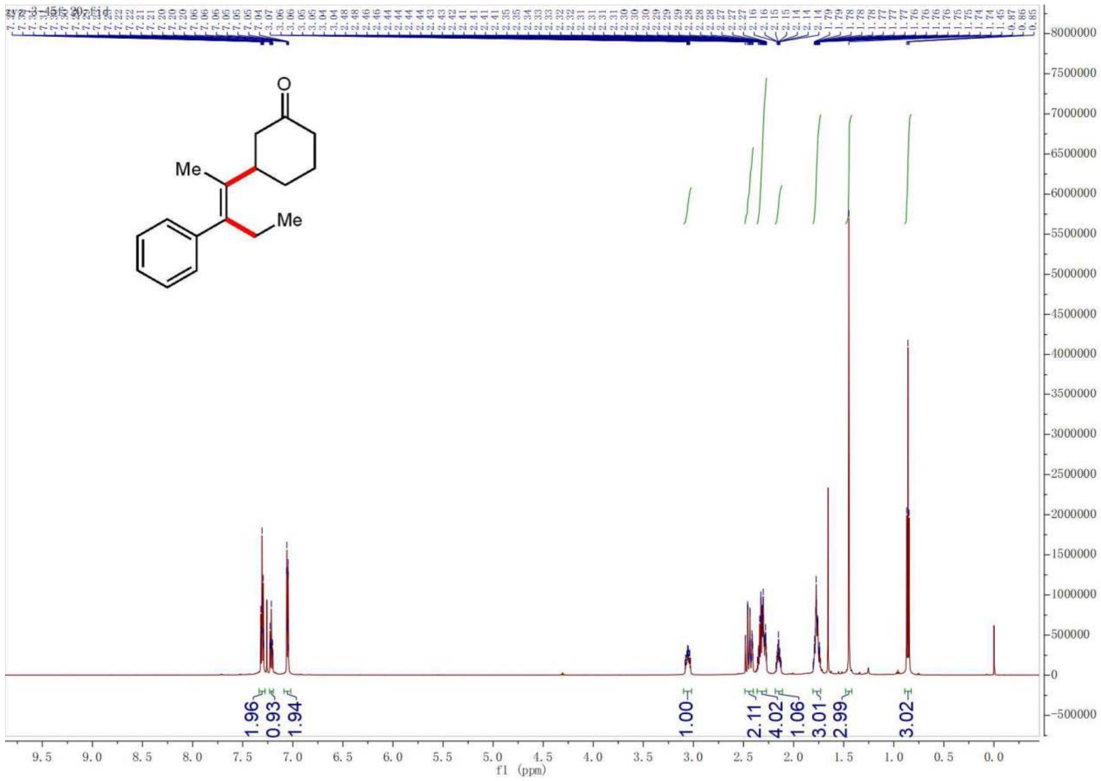

Supplementary Figure 104. ^{1}H NMR (600 MHz, $CDCl_3$) of **7k**

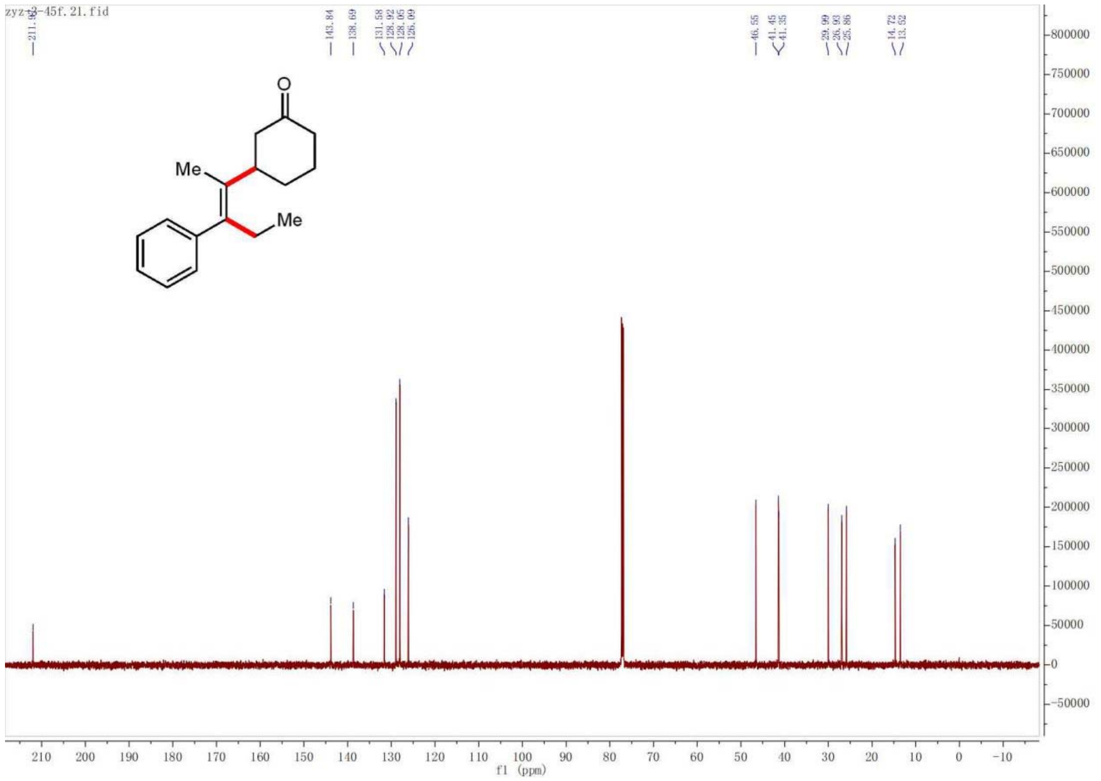

Supplementary Figure 105. ^{13}C NMR (151 MHz, $CDCl_3$) of **7k**

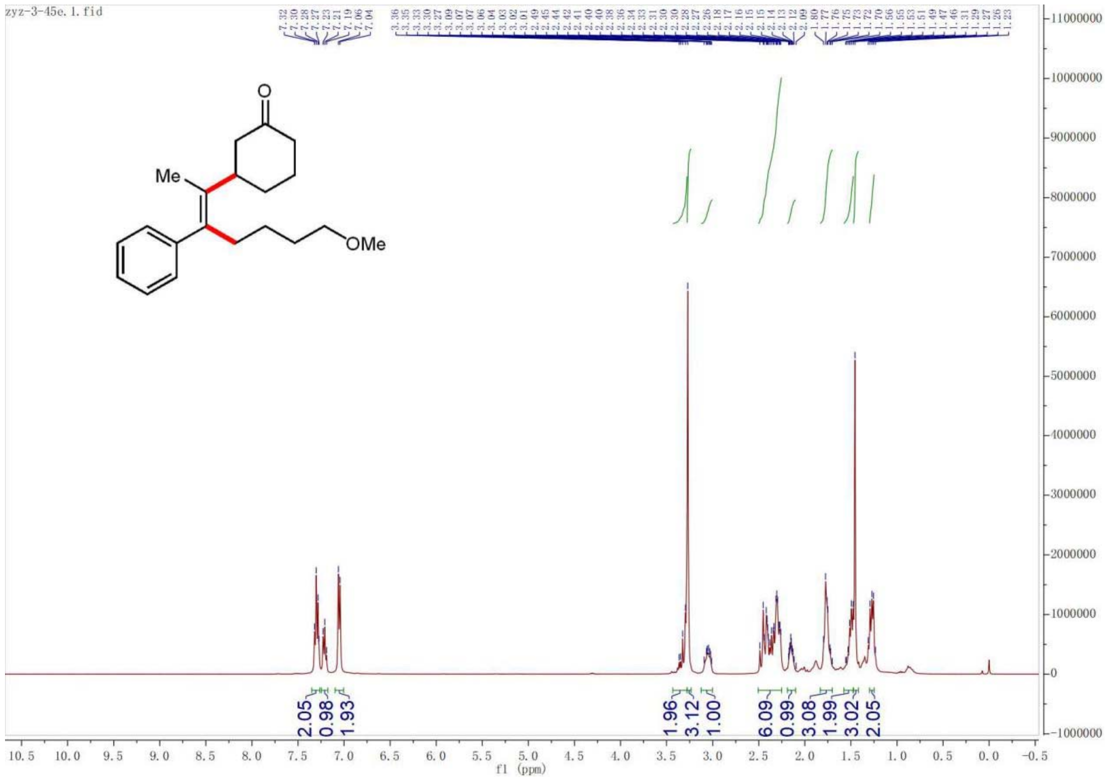

Supplementary Figure 106. ^{1}H NMR (400 MHz, $CDCl_3$) of **7l**

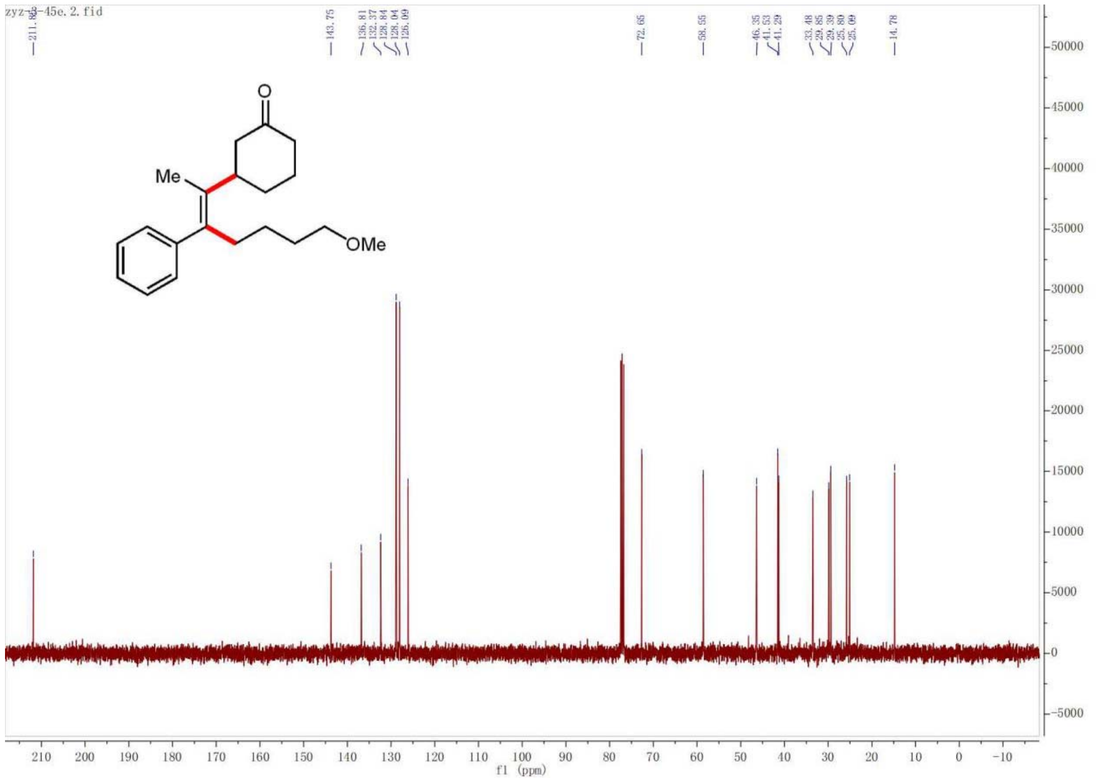

Supplementary Figure 107. ^{13}C NMR (101 MHz, $CDCl_3$) of **7l**

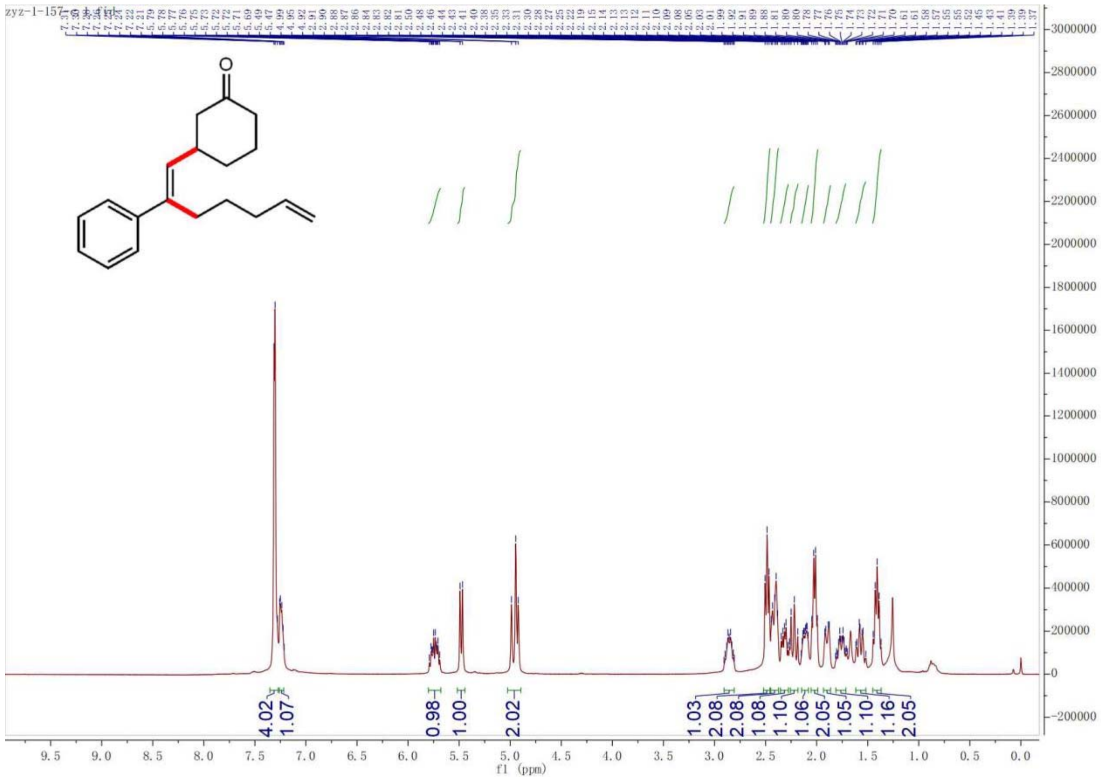

Supplementary Figure 108. ^{1}H NMR (400 MHz, $CDCl_3$) of **7m**

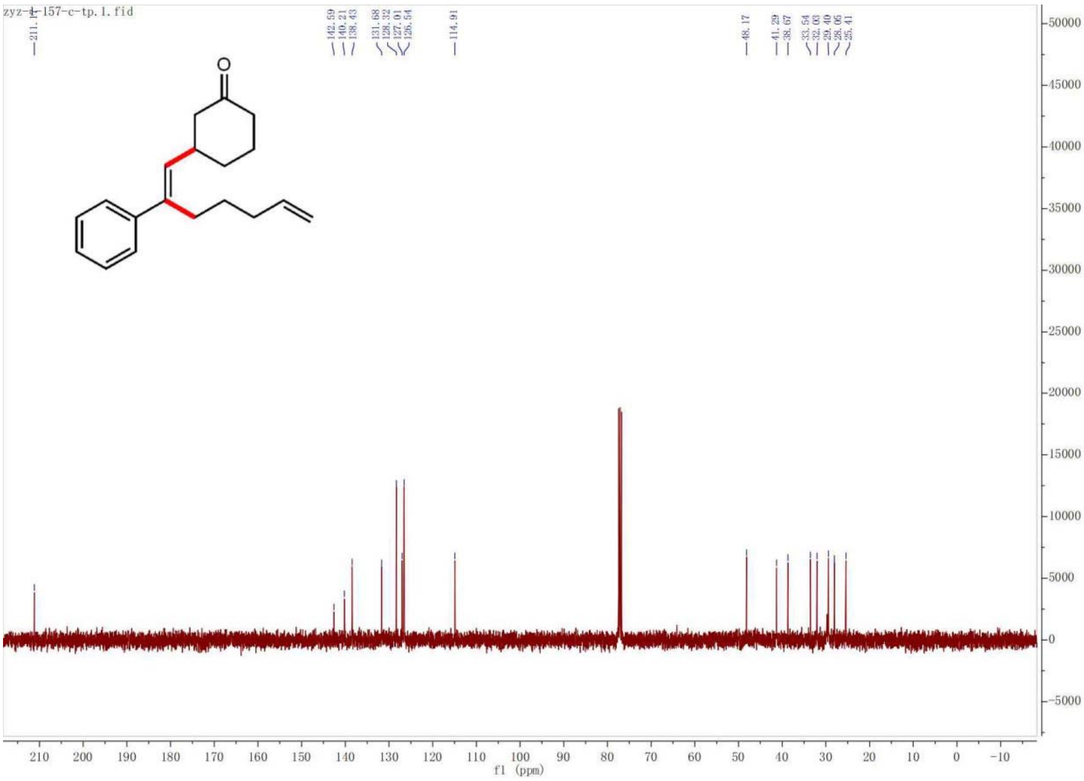

Supplementary Figure 109. ^{13}C NMR (101 MHz, $CDCl_3$) of **7m**

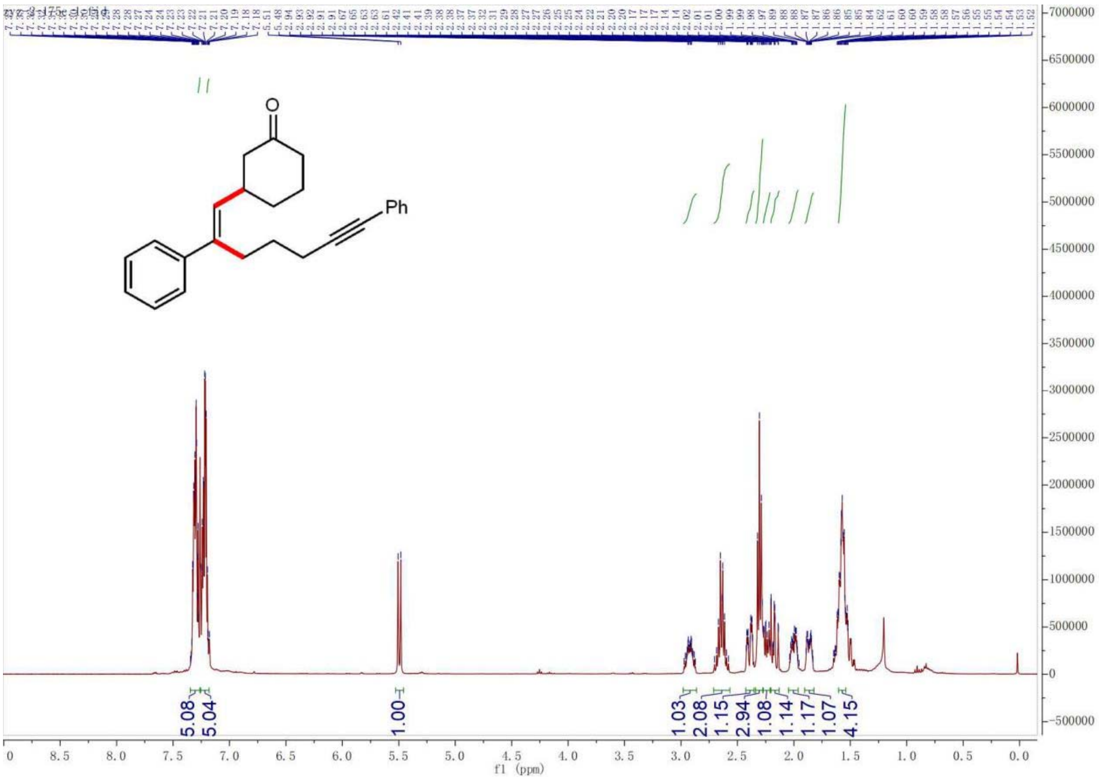

Supplementary Figure 110. ^{1}H NMR (400 MHz, $CDCl_3$) of **7n**

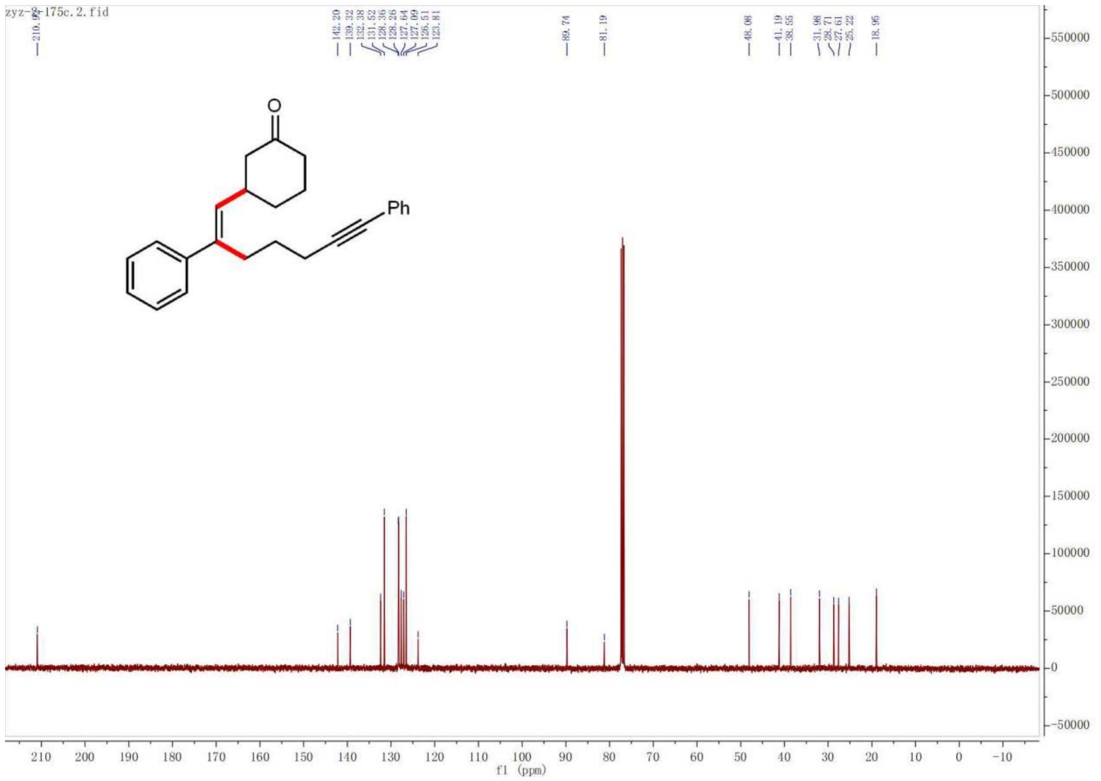

Supplementary Figure 111. ^{13}C NMR (101 MHz, $CDCl_3$) of **7n**

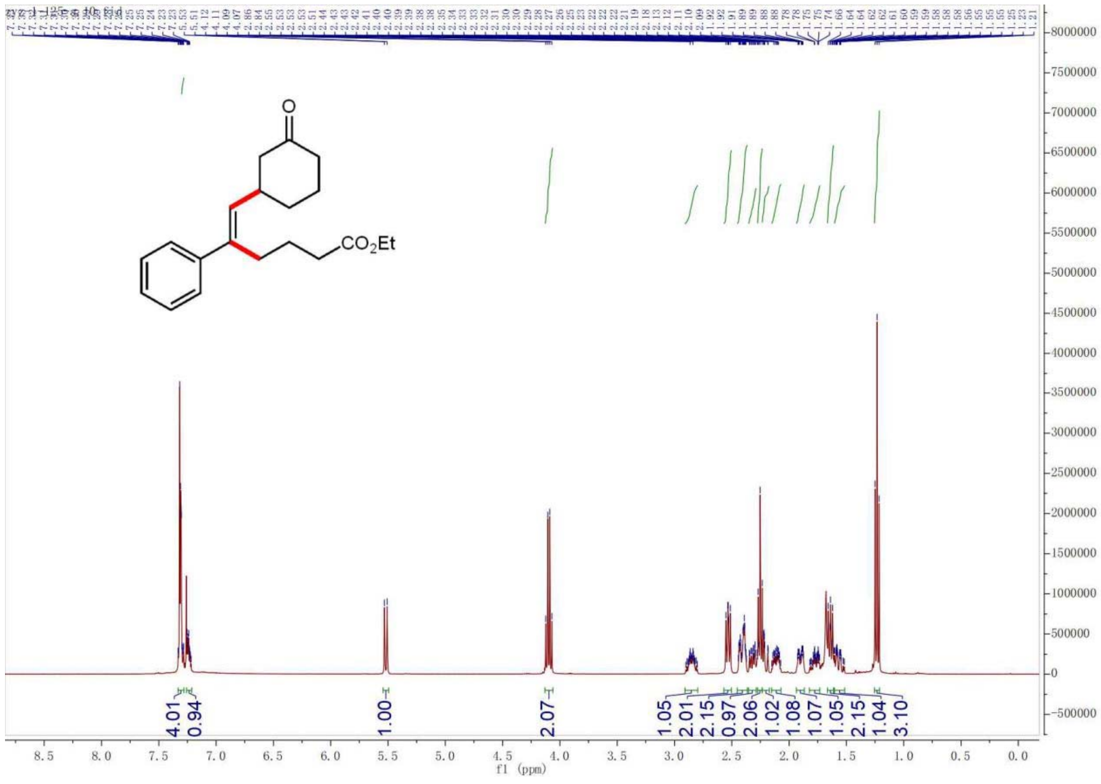

Supplementary Figure 112. ^{1}H NMR (400 MHz, $CDCl_3$) of **7o**

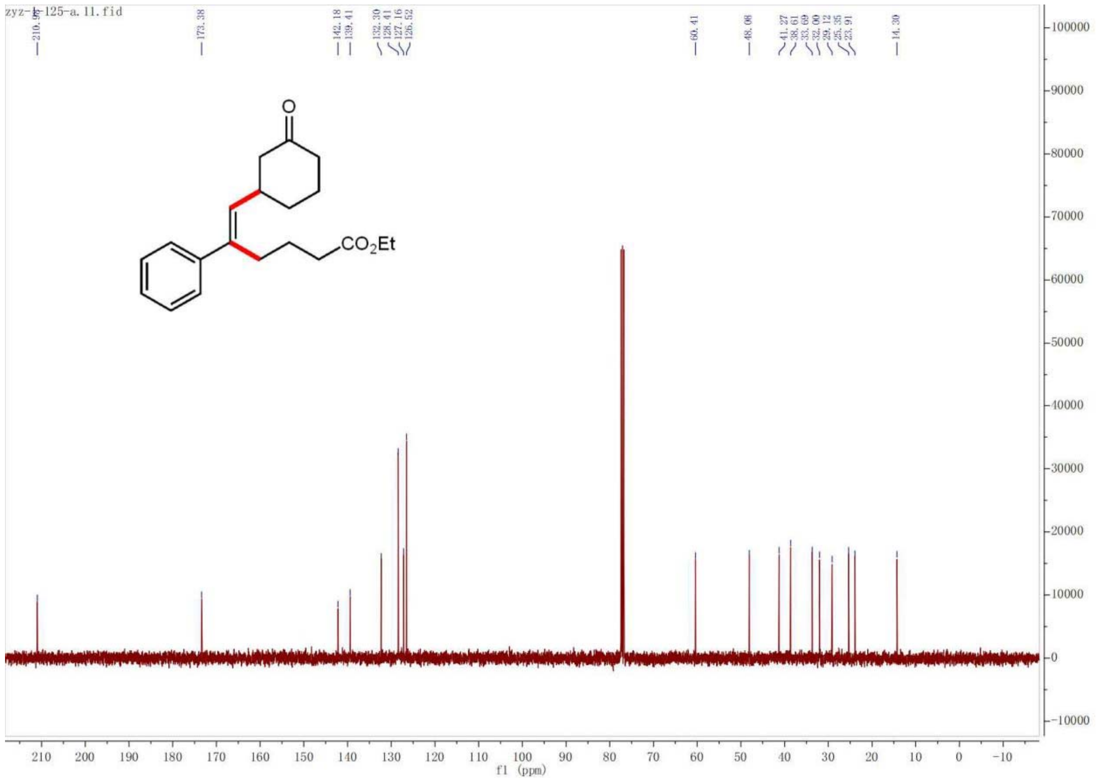

Supplementary Figure 113. ^{13}C NMR (101 MHz, $CDCl_3$) of **7o**

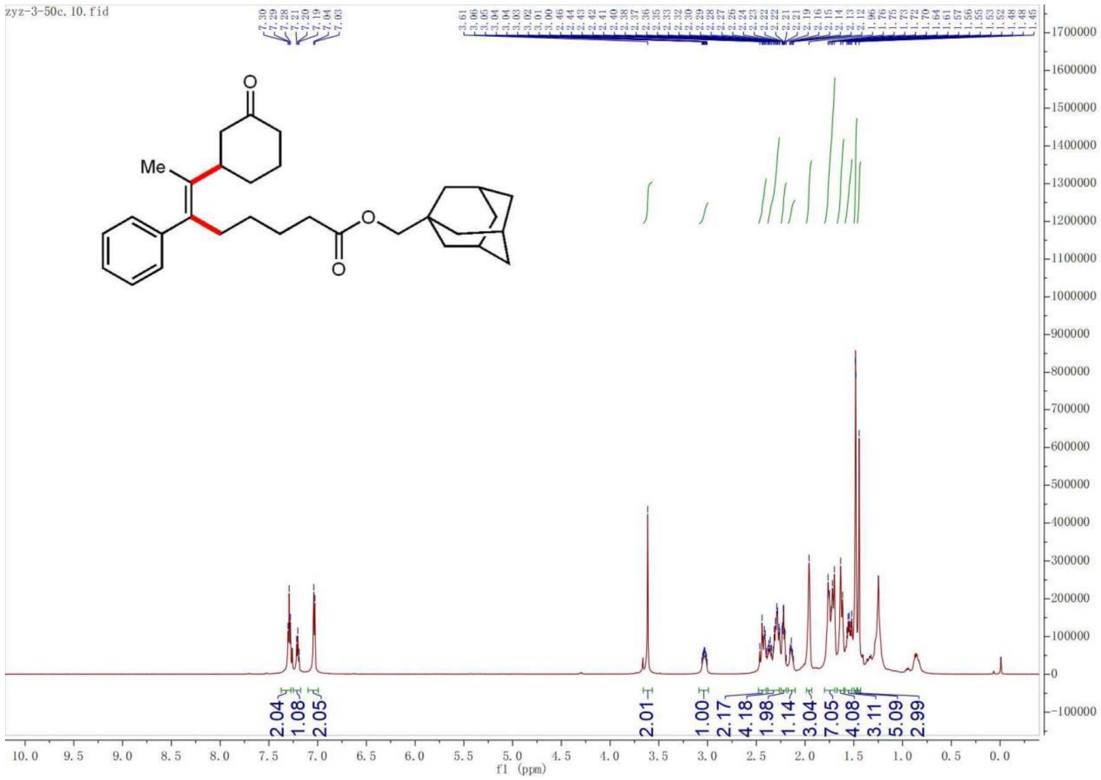

Supplementary Figure 114. ^{1}H NMR (600 MHz, $CDCl_3$) of **7p**

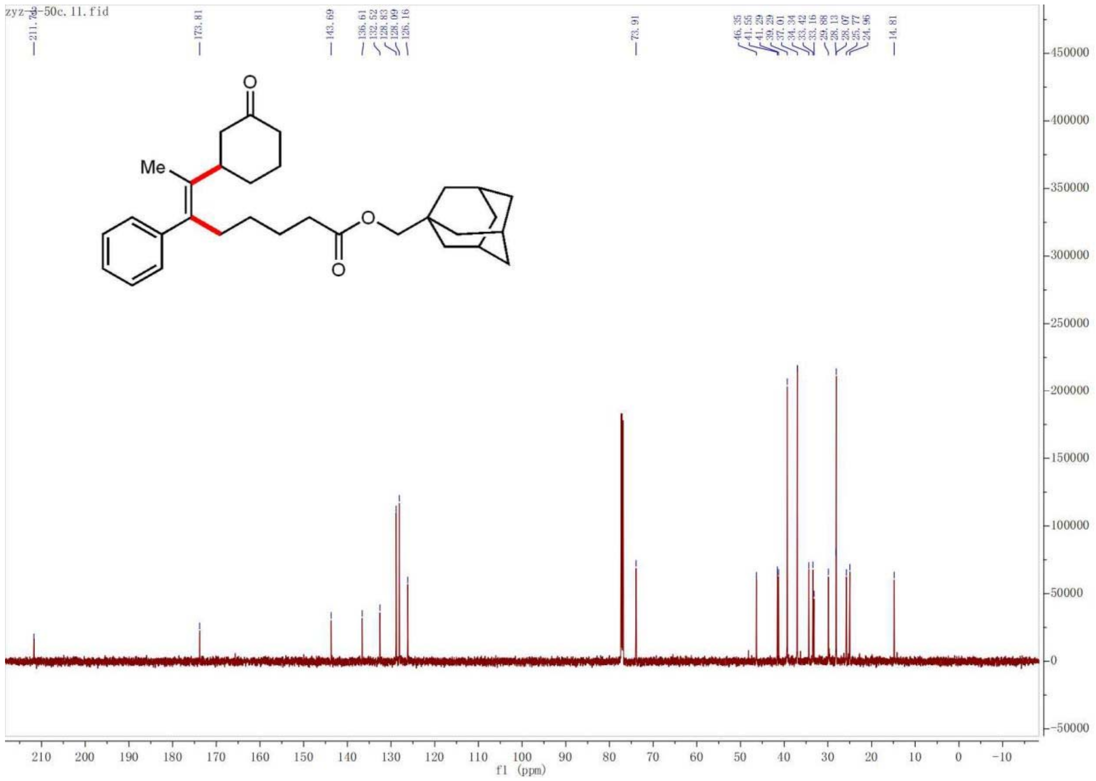

Supplementary Figure 115. ^{13}C NMR (151 MHz, $CDCl_3$) of **7p**

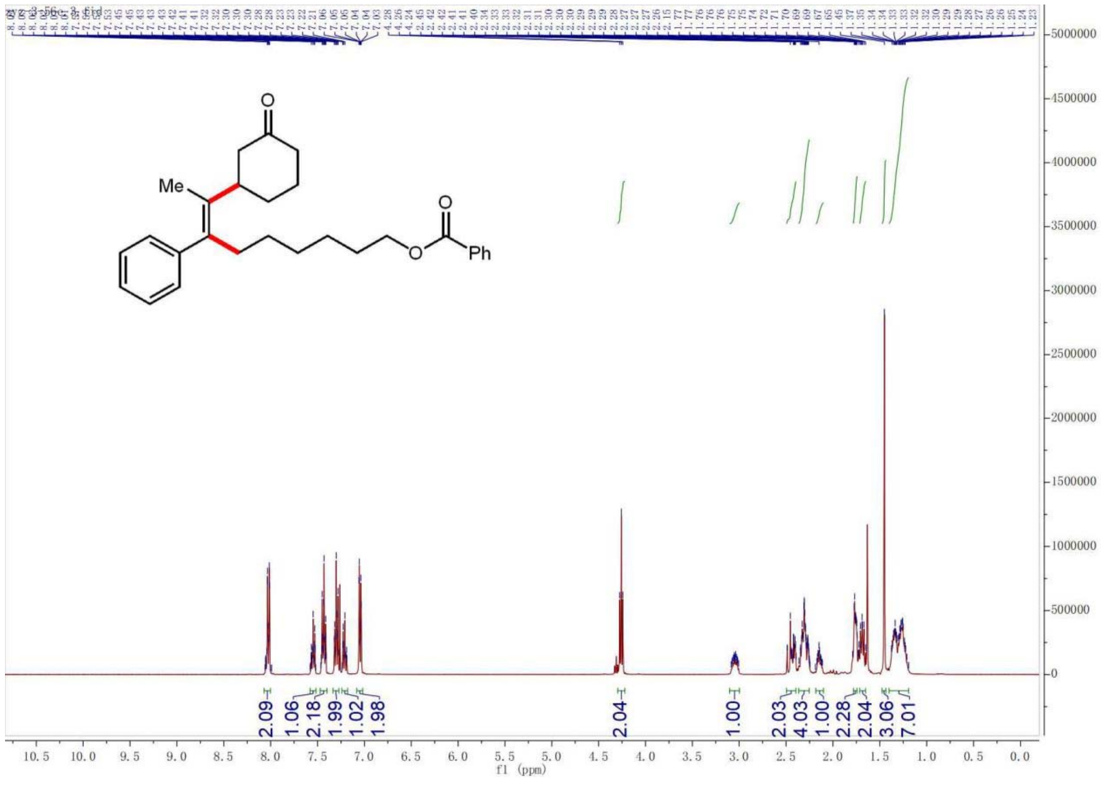

Supplementary Figure 116. ^{1}H NMR (400 MHz, $CDCl_3$) of **7q**

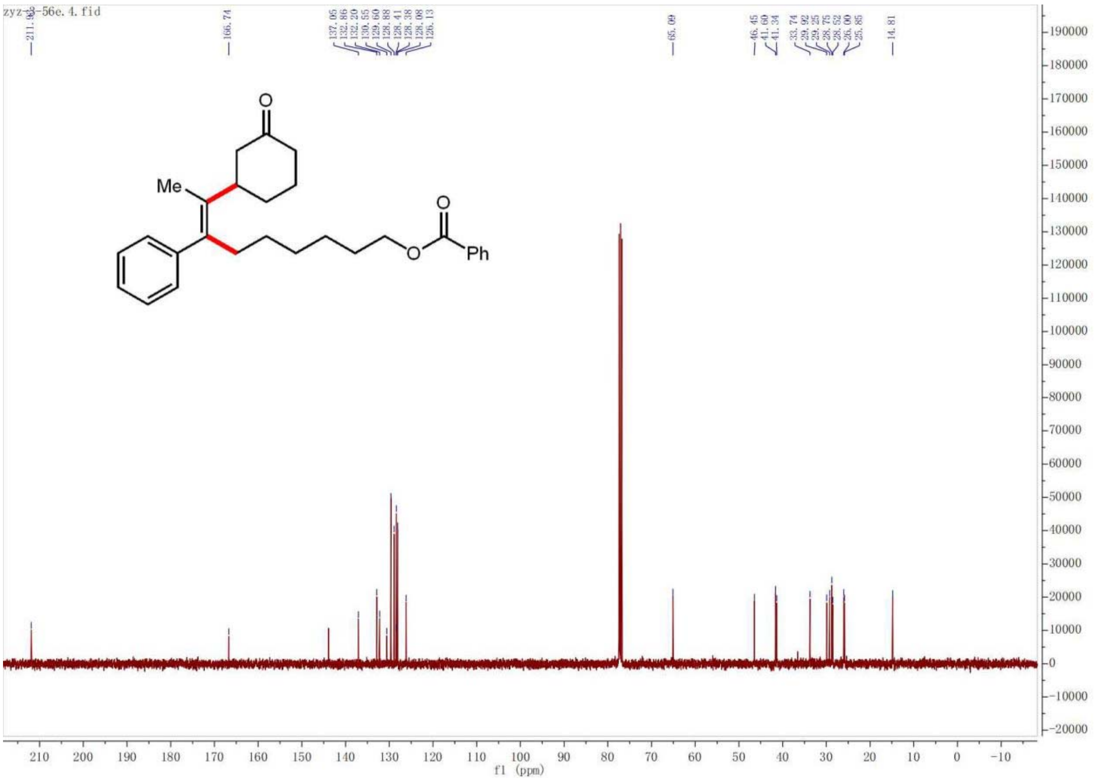

Supplementary Figure 117. ^{13}C NMR (101 MHz, $CDCl_3$) of **7q**

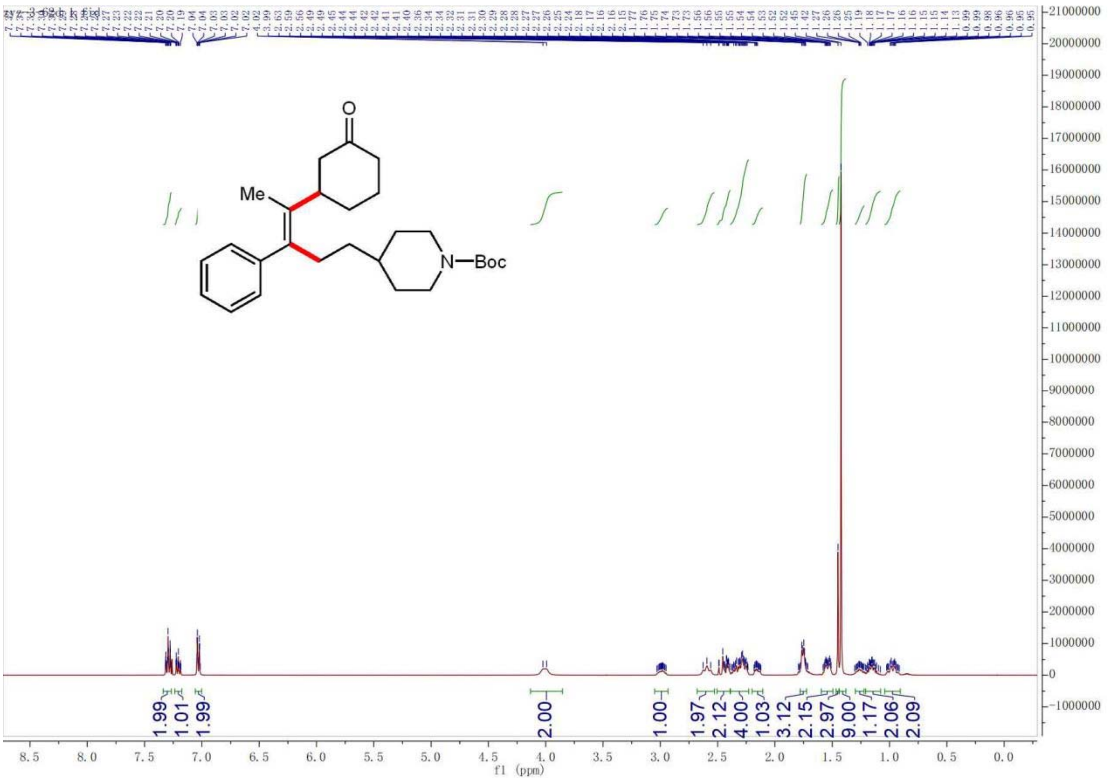

Supplementary Figure 118. ^{1}H NMR (400 MHz, $CDCl_3$) of **7r**

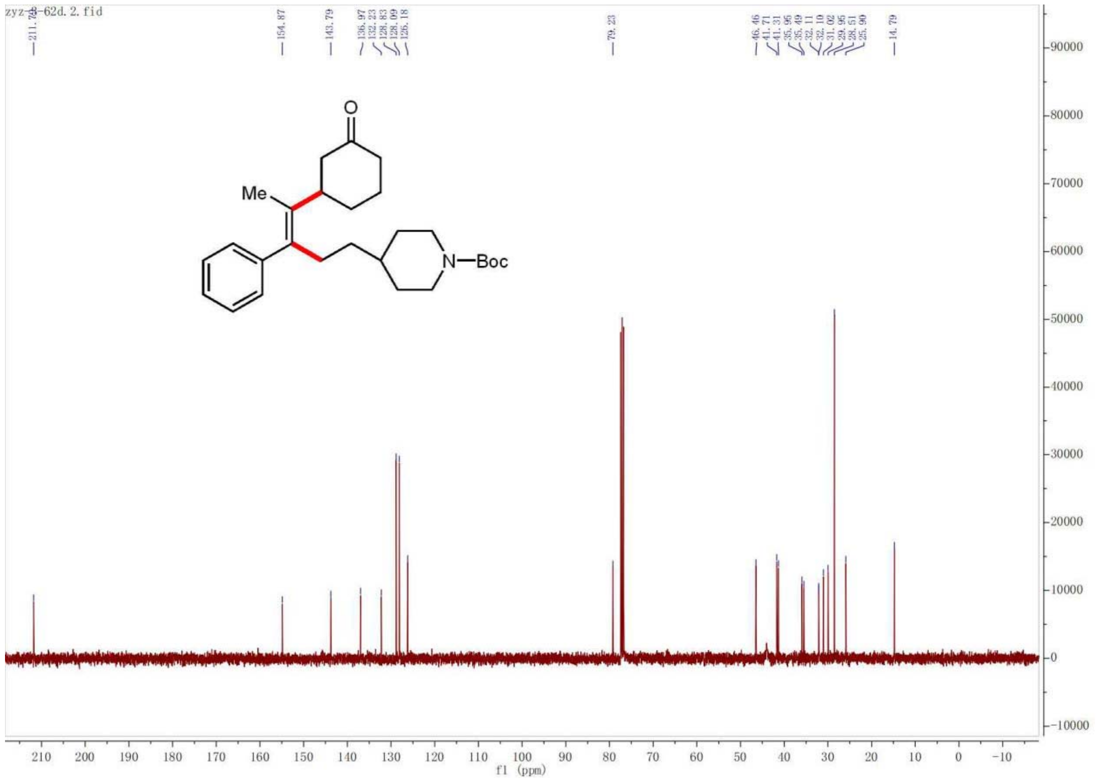

Supplementary Figure 119. ^{13}C NMR (101 MHz, $CDCl_3$) of **7r**

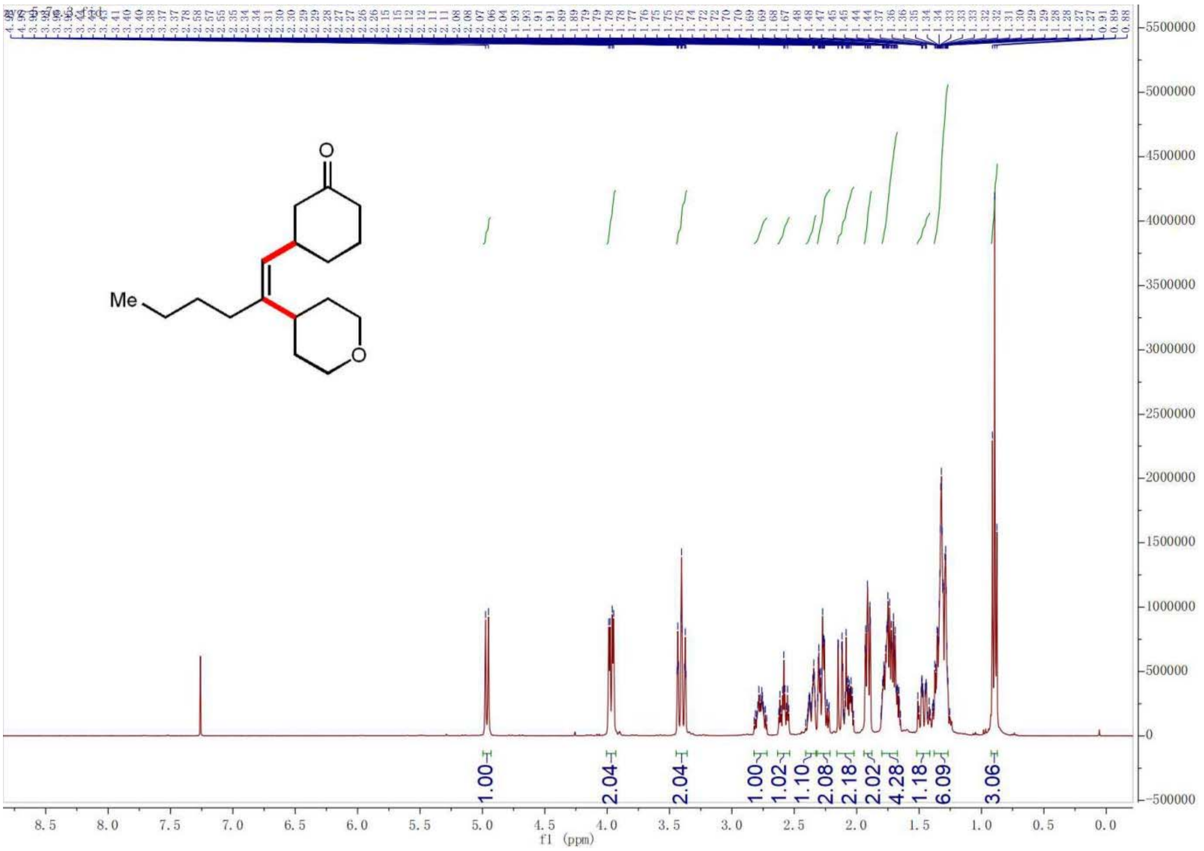

Supplementary Figure 120. ^{1}H NMR (400 MHz, $CDCl_3$) of **7s**

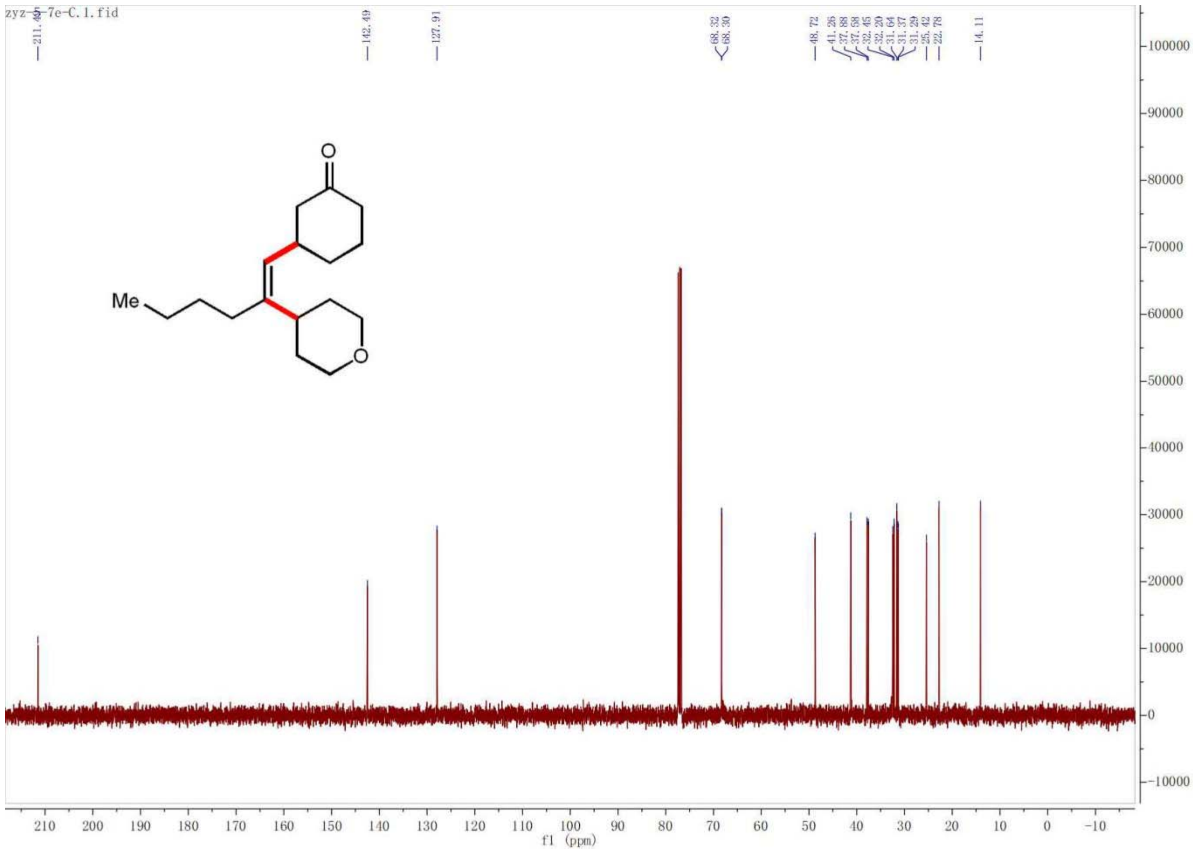

Supplementary Figure 121. ^{13}C NMR (101 MHz, $CDCl_3$) of **7s**

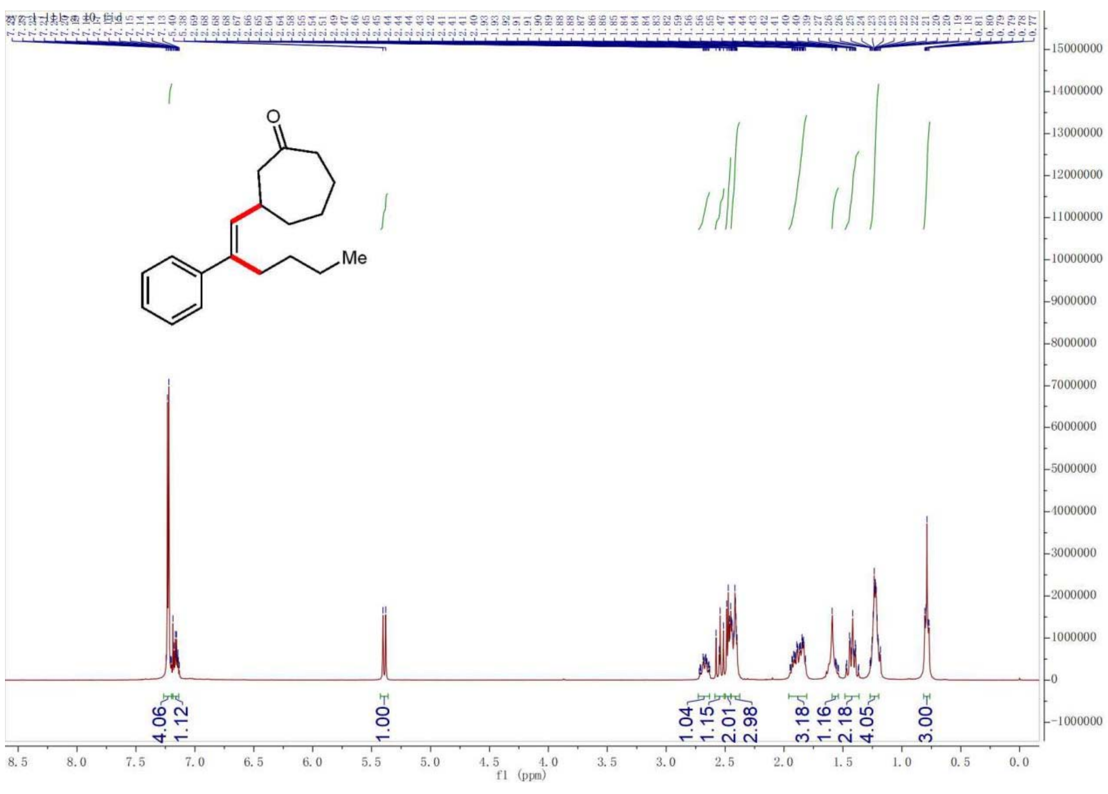

Supplementary Figure 122. ^{1}H NMR (400 MHz, $CDCl_3$) of **8a**

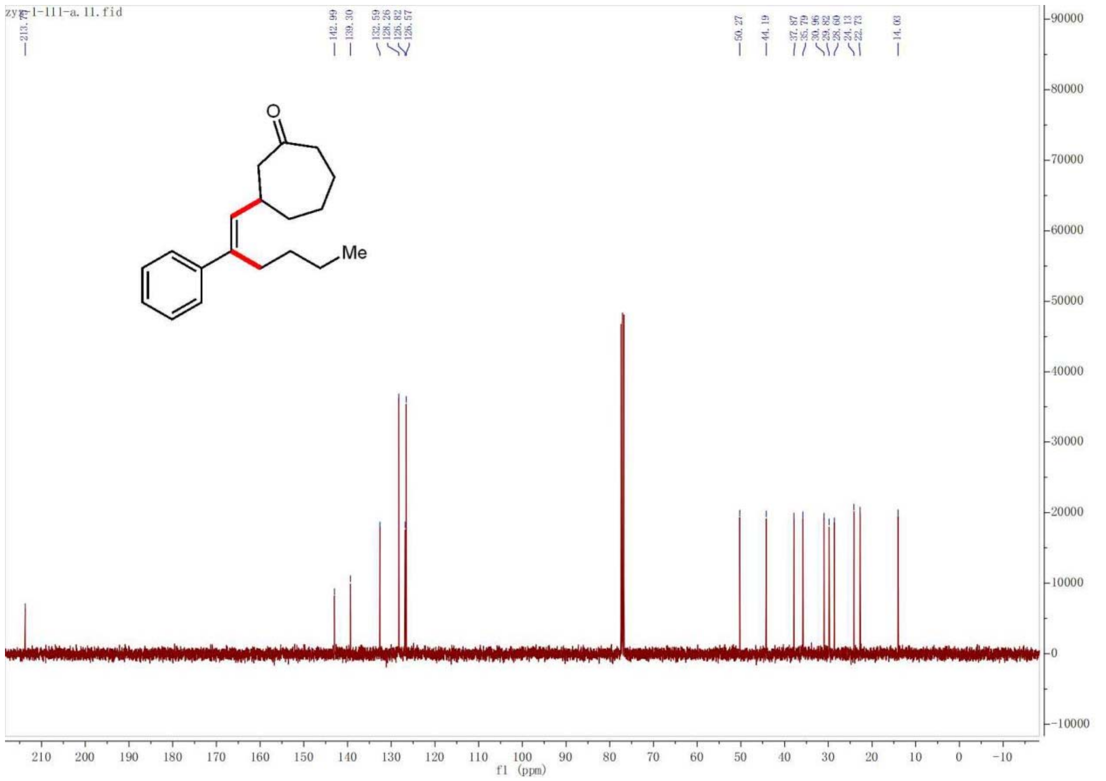

Supplementary Figure 123. ^{13}C NMR (101 MHz, $CDCl_3$) of **8a**

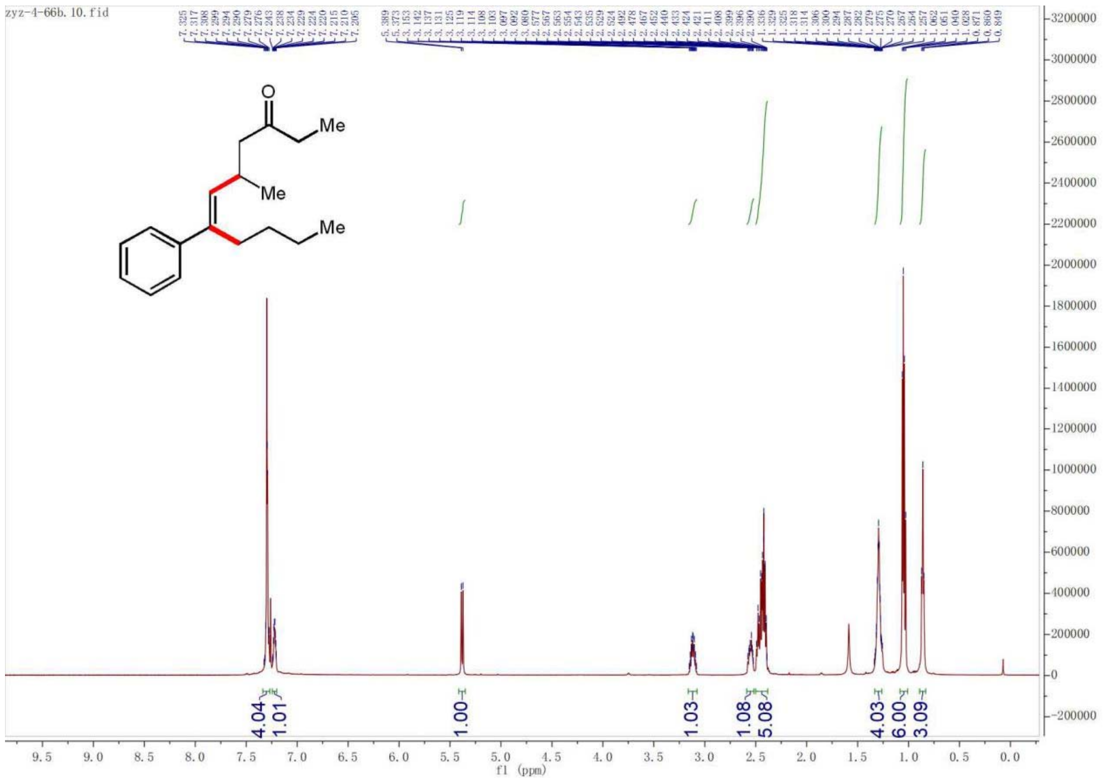

Supplementary Figure 124. ^{1}H NMR (600 MHz, $CDCl_3$) of **8b**

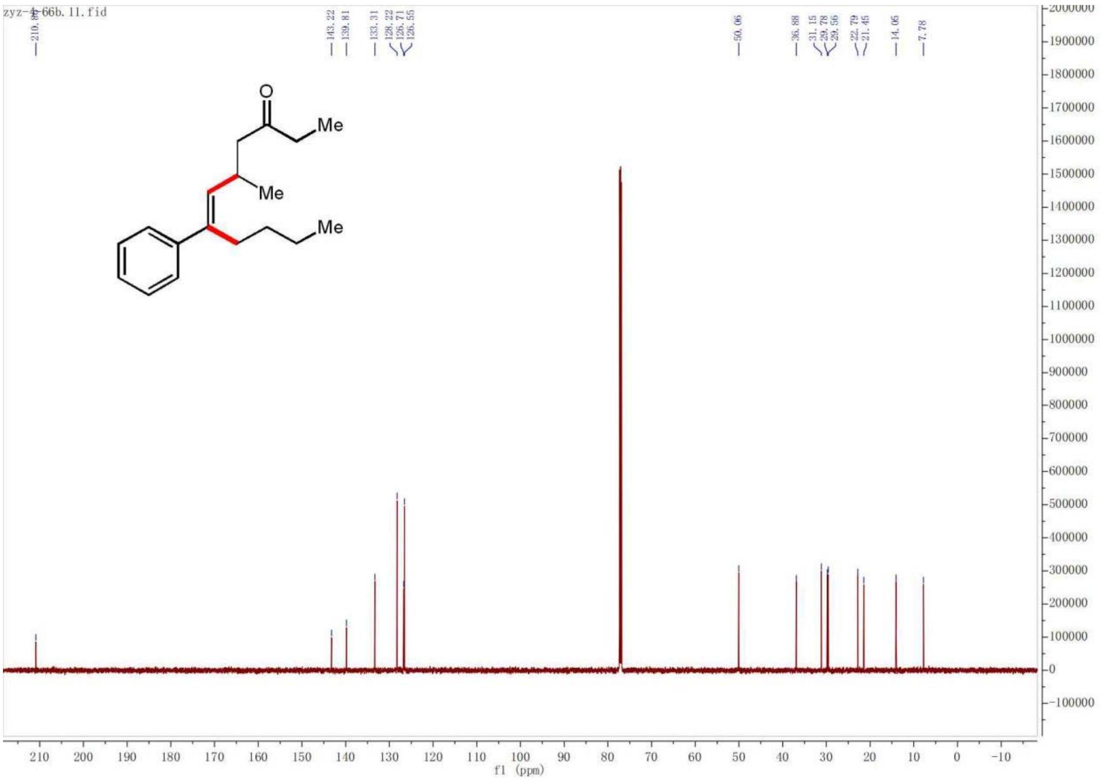

Supplementary Figure 125. ^{13}C NMR (151 MHz, $CDCl_3$) of **8b**

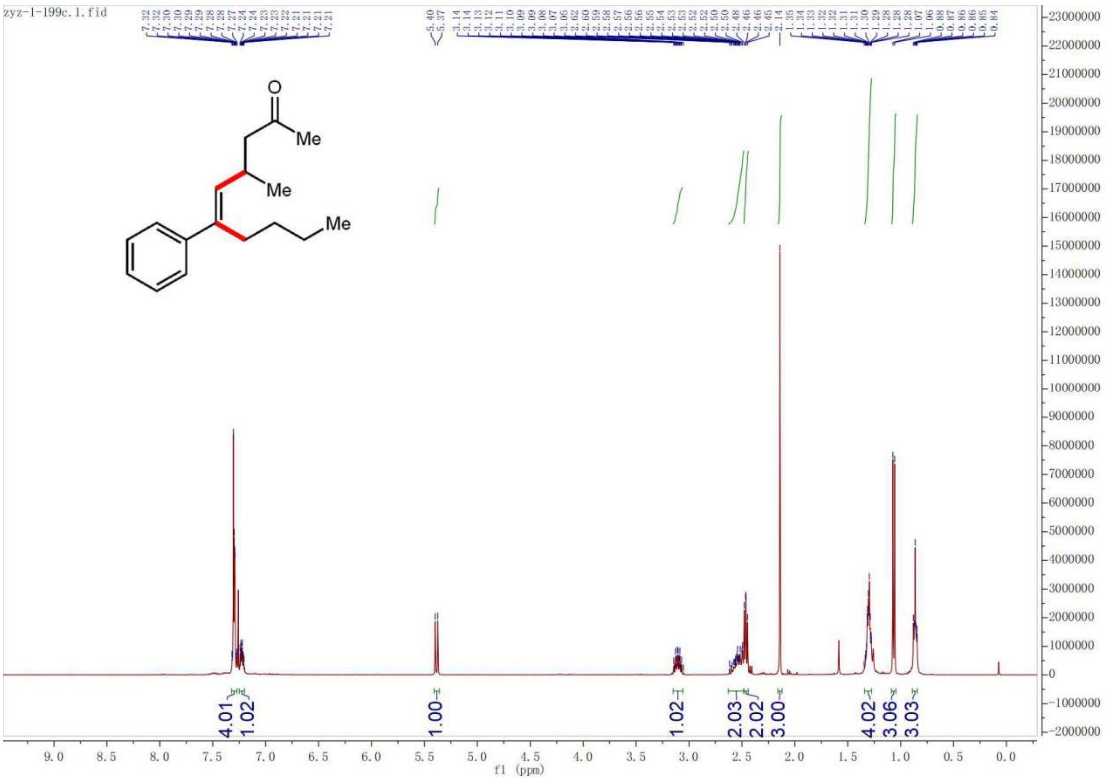

Supplementary Figure 126. ^{1}H NMR (400 MHz, $CDCl_3$) of **8c**

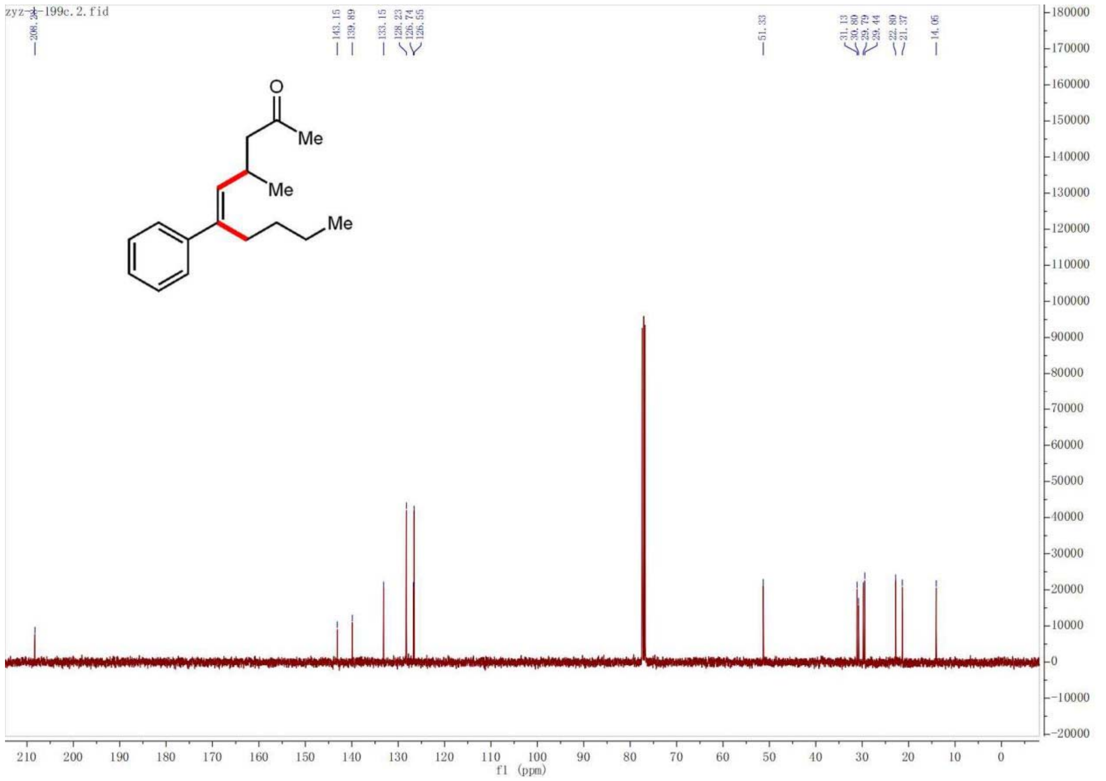

Supplementary Figure 127. ^{13}C NMR (101 MHz, $CDCl_3$) of **8c**

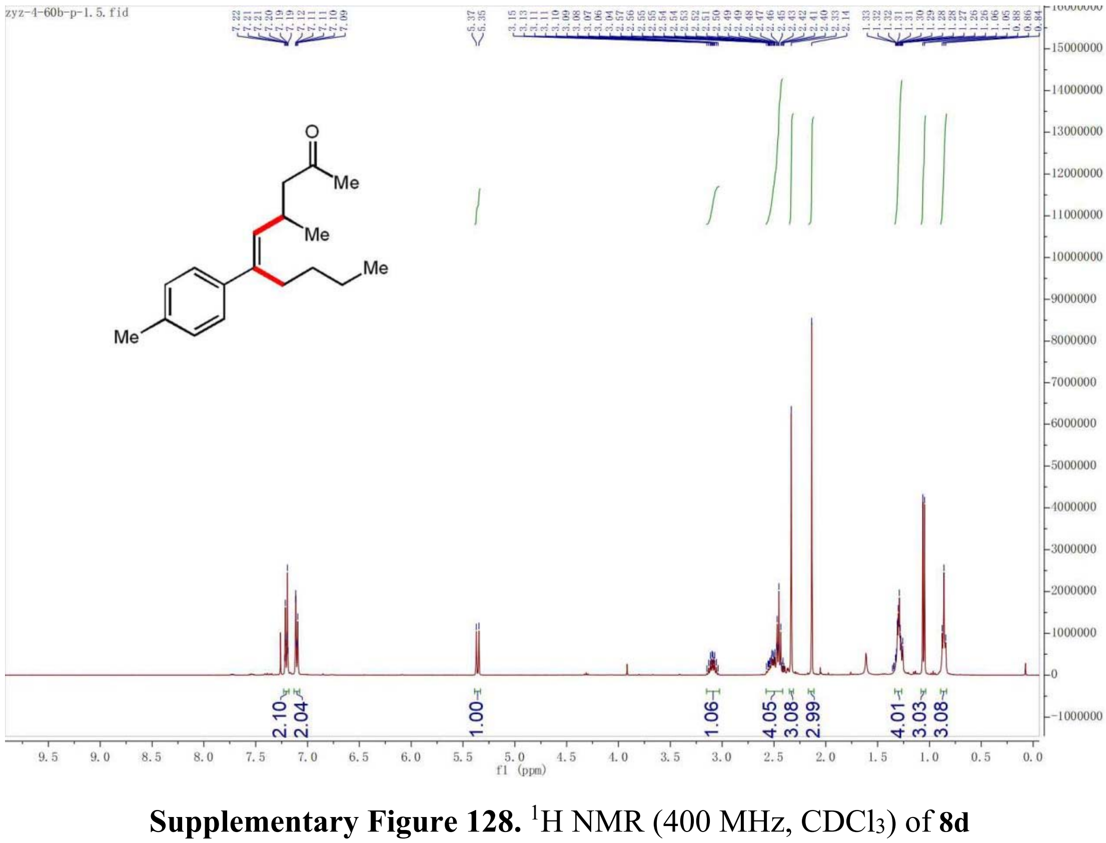

Supplementary Figure 128. ^{1}H NMR (400 MHz, $CDCl_3$) of **8d**

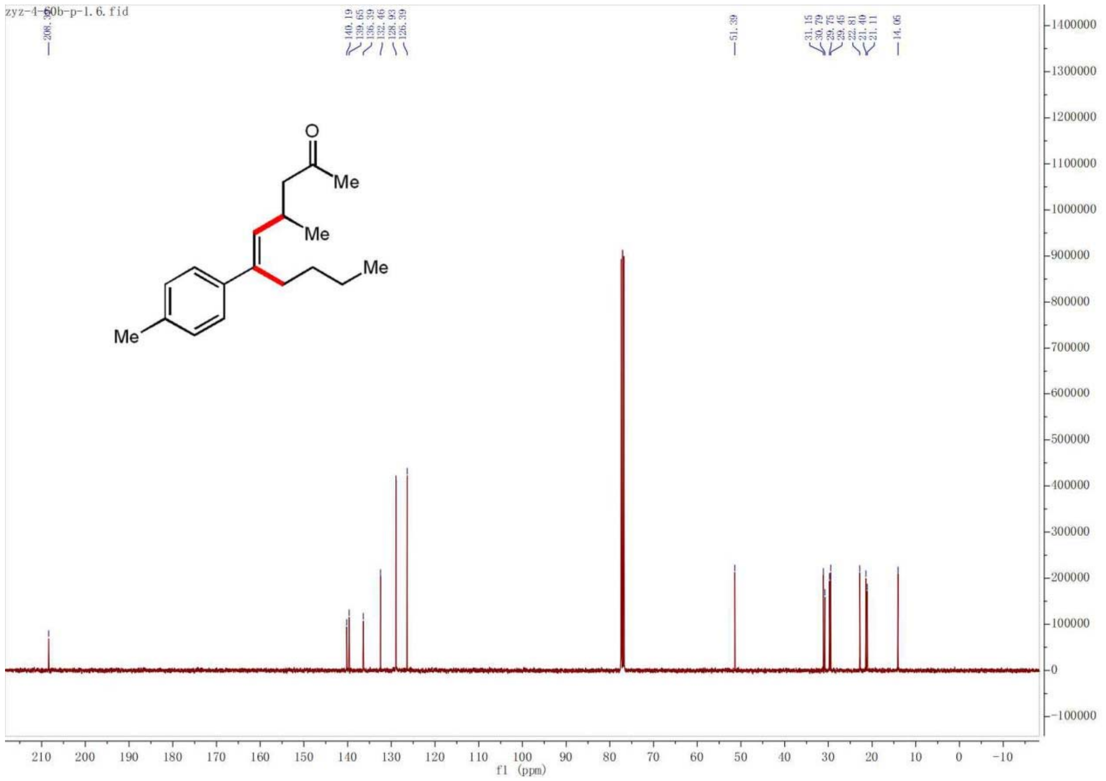

Supplementary Figure 129. ^{13}C NMR (101 MHz, $CDCl_3$) of **8d**

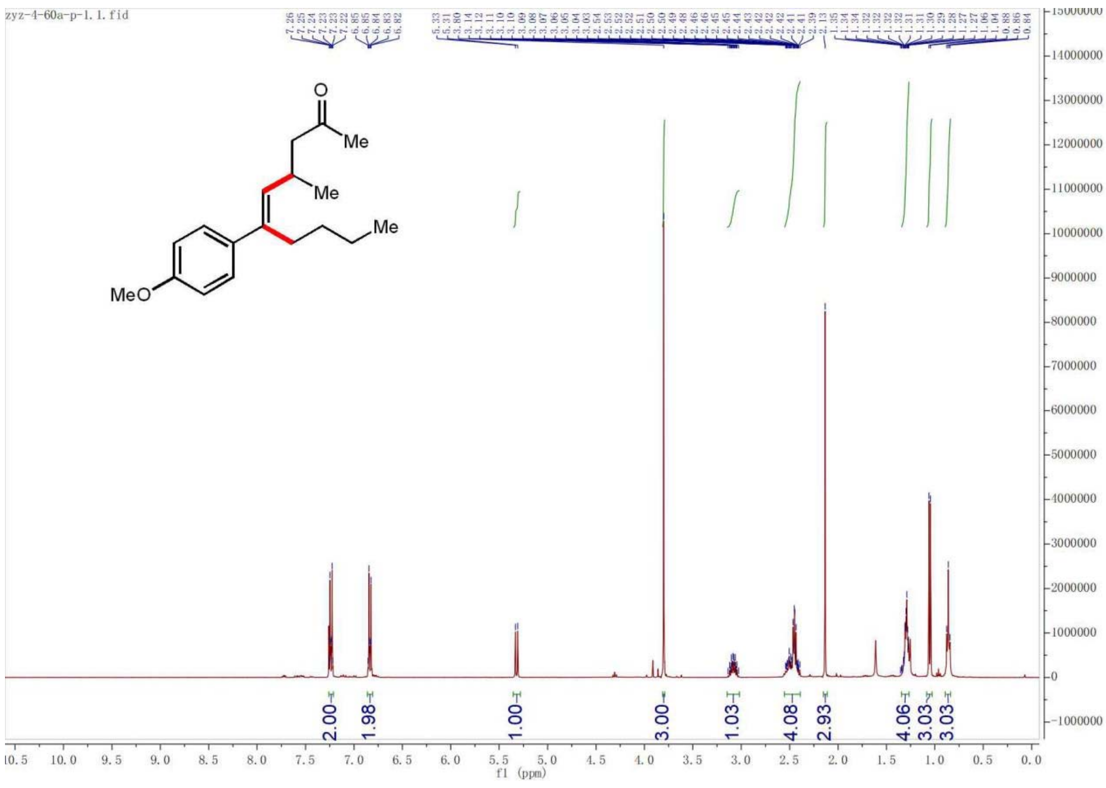

Supplementary Figure 130. ^{1}H NMR (400 MHz, $CDCl_3$) of **8e**

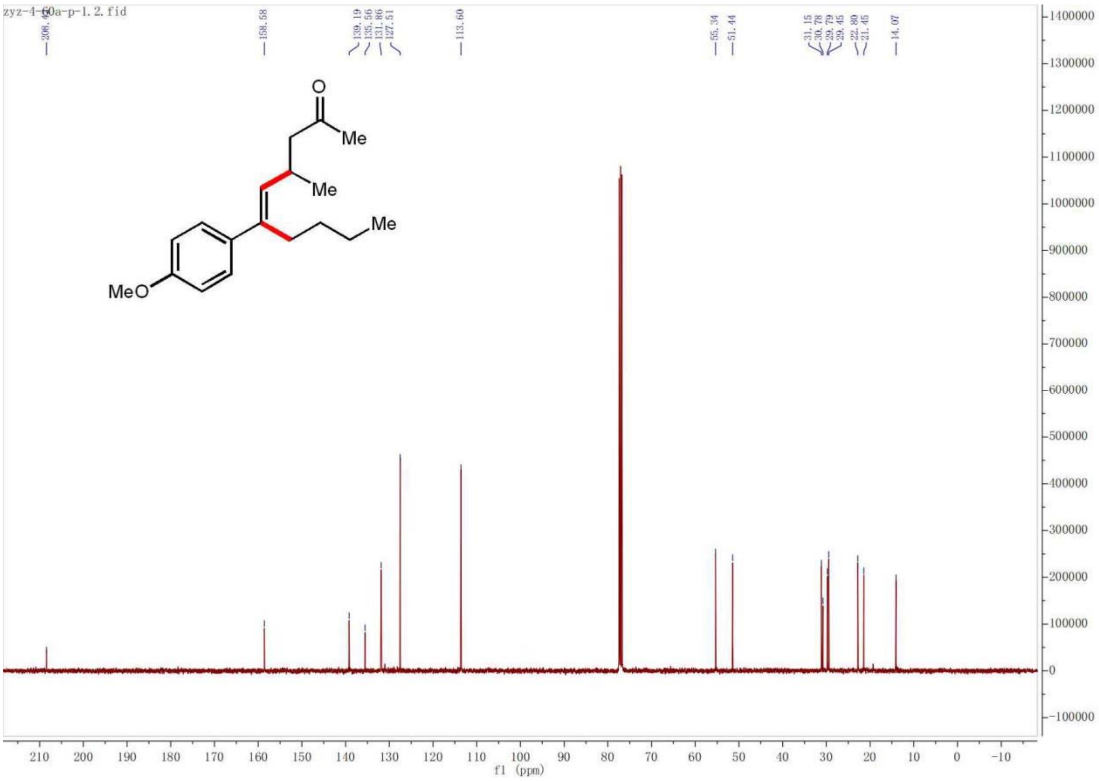

Supplementary Figure 131. ^{13}C NMR (101 MHz, $CDCl_3$) of **8e**

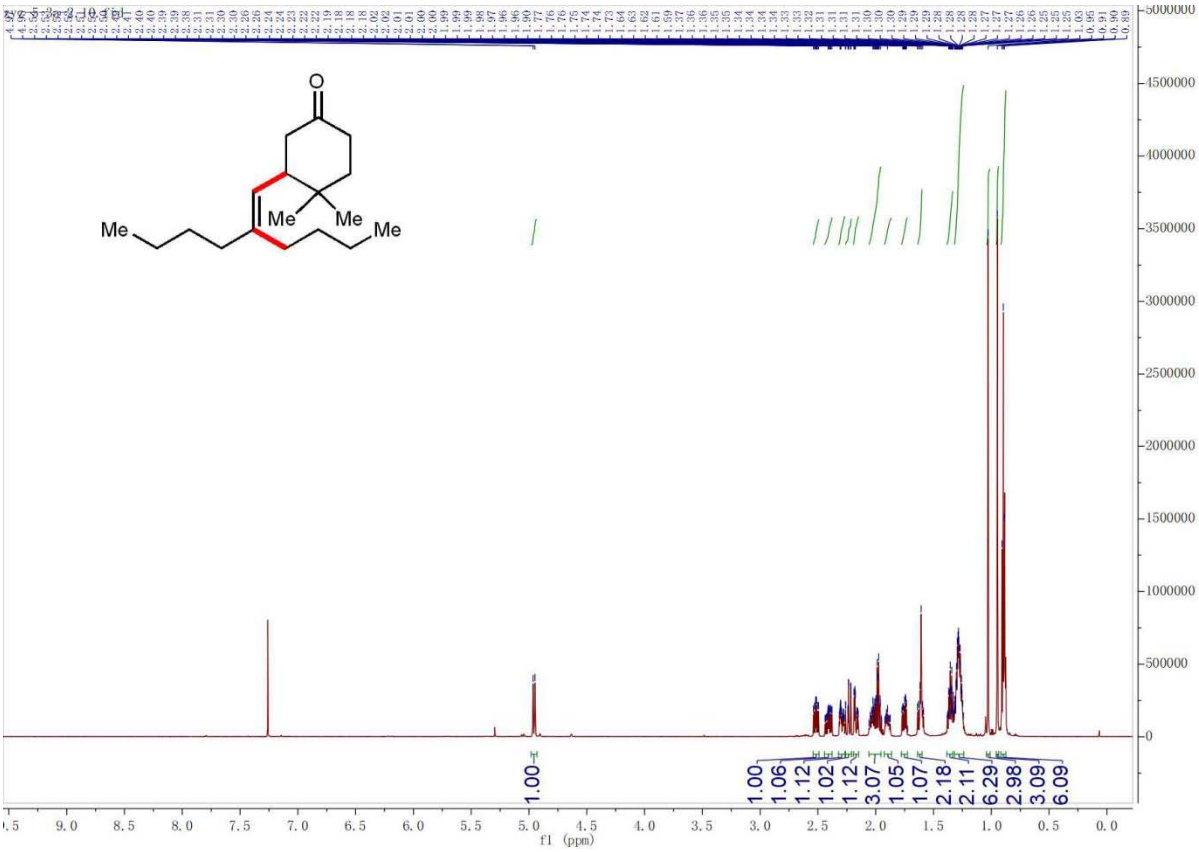

Supplementary Figure 132. ^{1}H NMR (600 MHz, $CDCl_3$) of **8f**

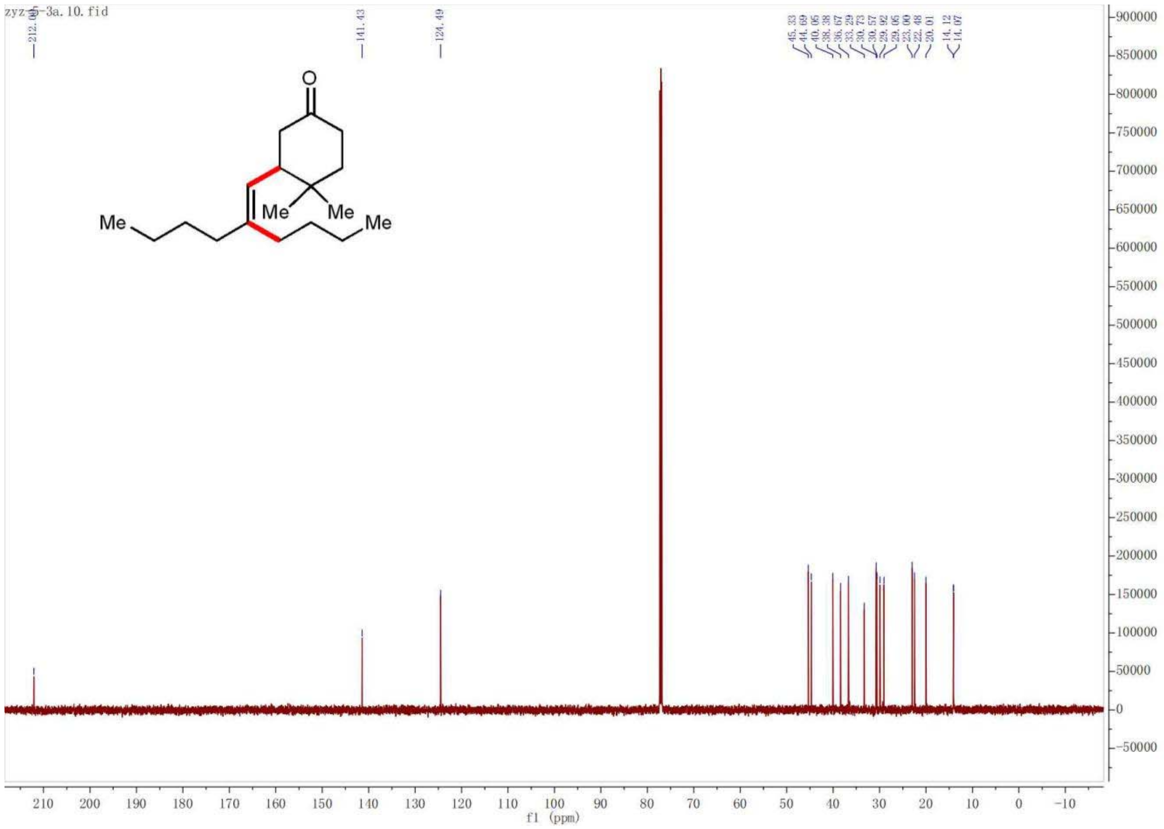

Supplementary Figure 133. ^{13}C NMR (151 MHz, $CDCl_3$) of **8f**

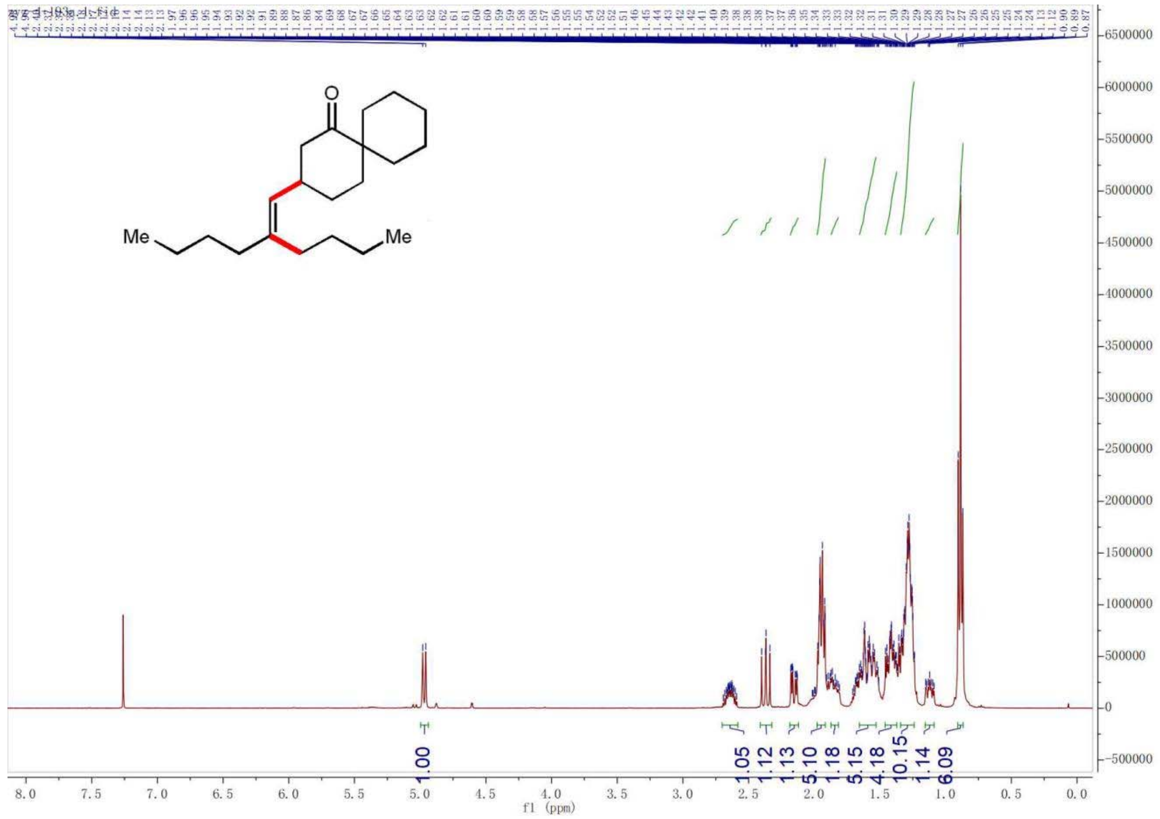

Supplementary Figure 134. ^{1}H NMR (400 MHz, $CDCl_3$) of **8g**

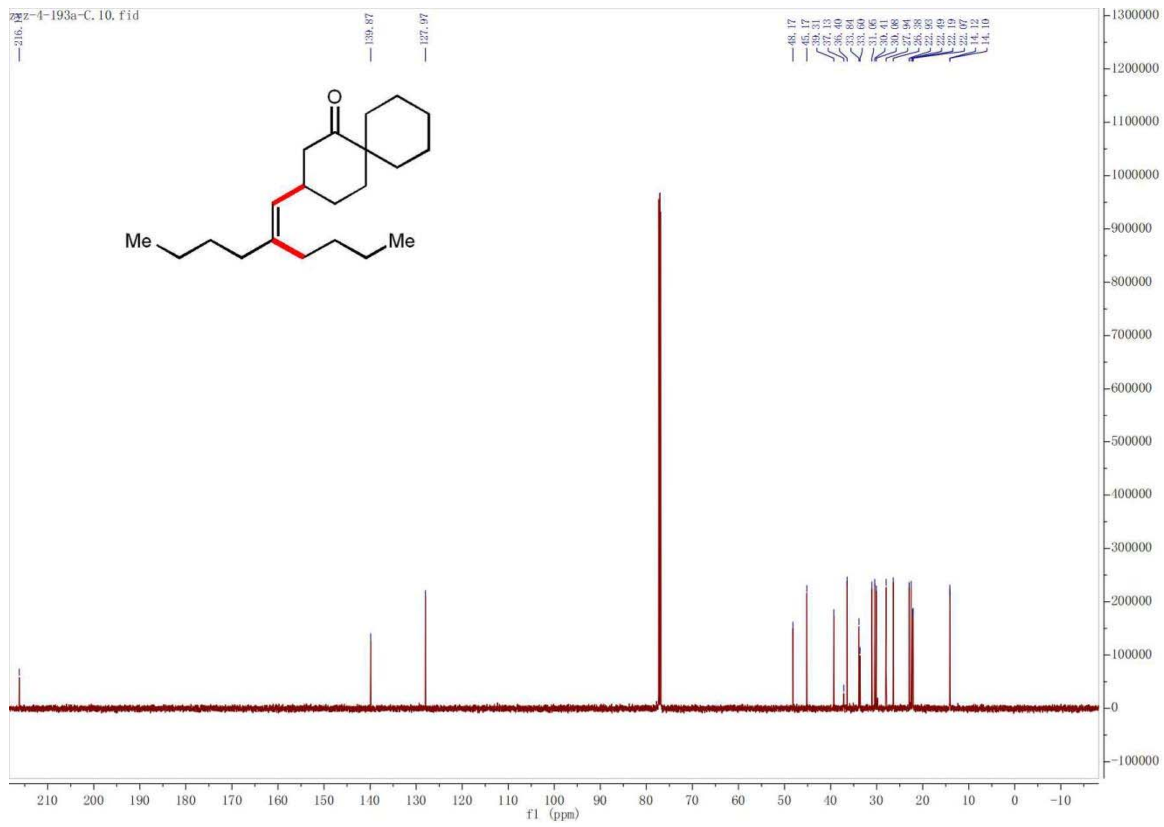

Supplementary Figure 135. ^{13}C NMR (151 MHz, $CDCl_3$) of **8g**

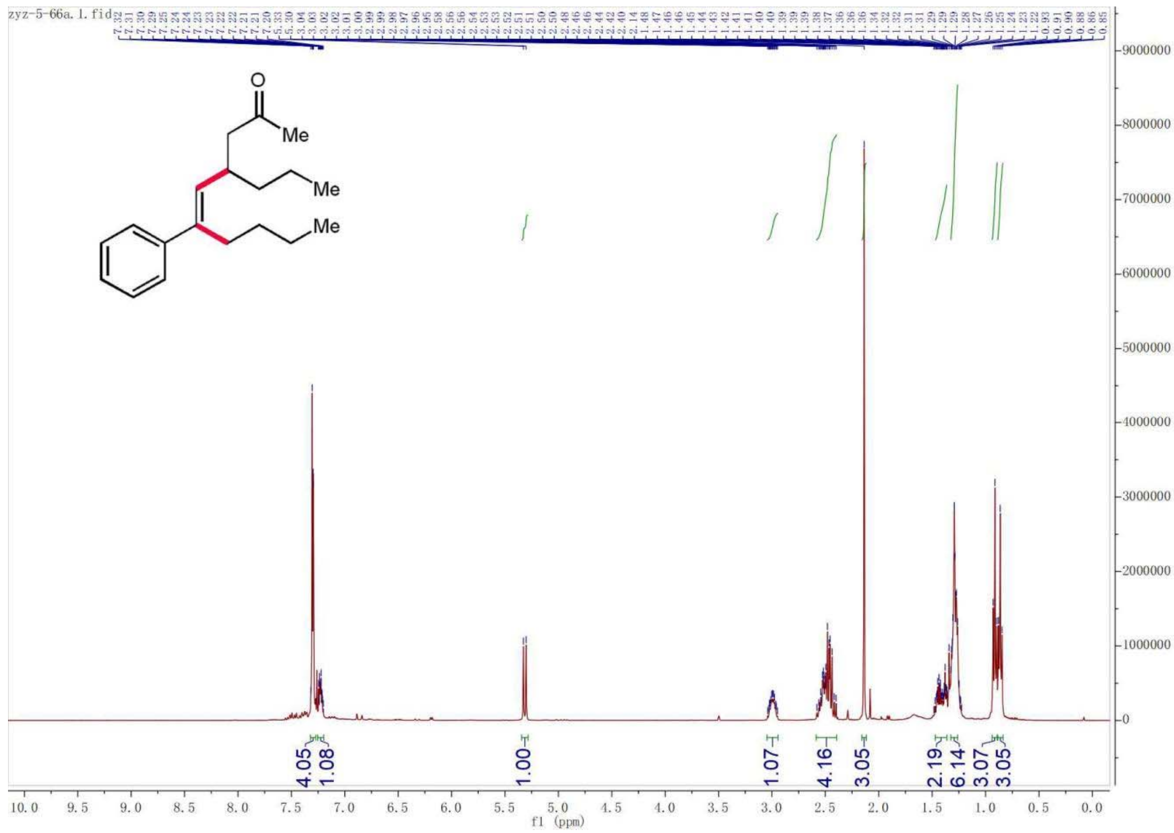

Supplementary Figure 136. ^{1}H NMR (400 MHz, $CDCl_3$) of **8h**

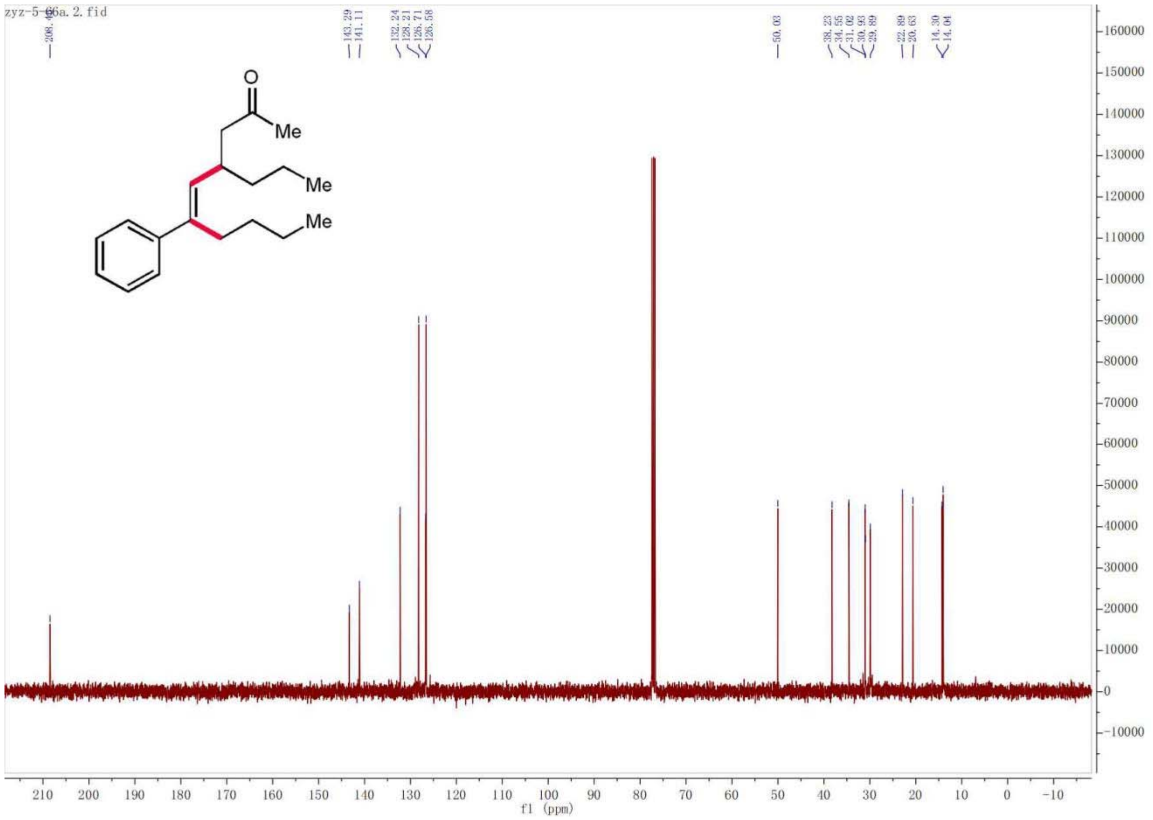

Supplementary Figure 137. ^{13}C NMR (101 MHz, $CDCl_3$) of **8h**

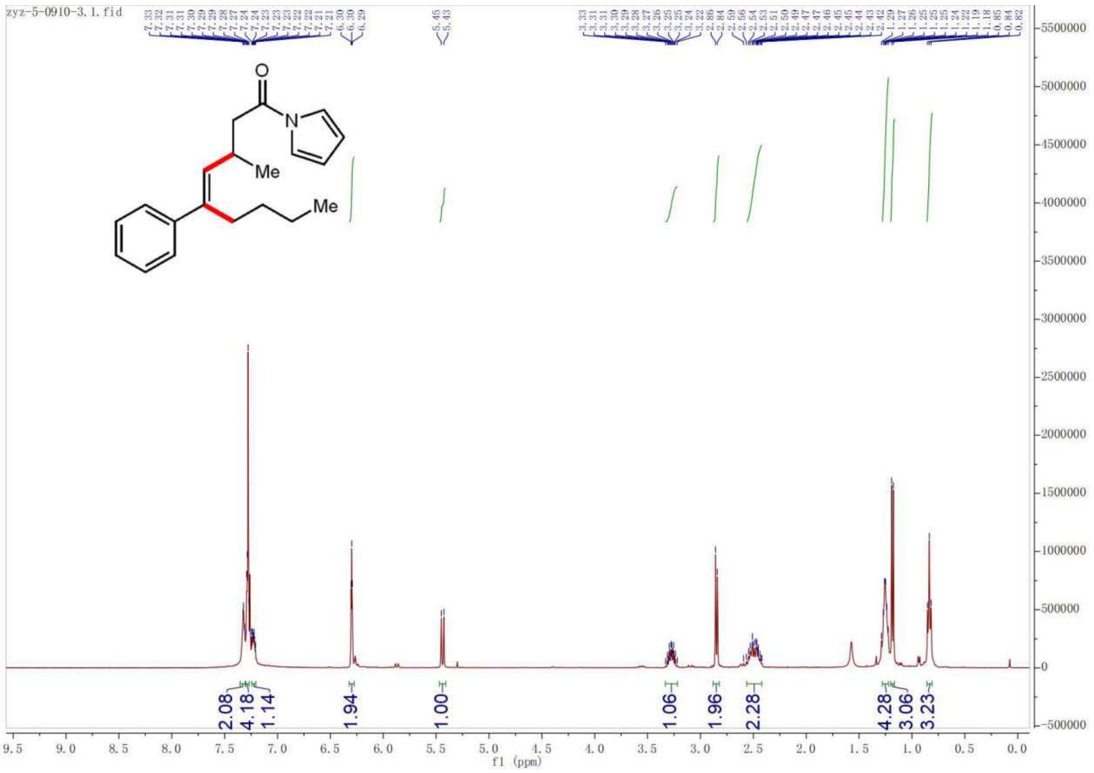

Supplementary Figure 138. ^{1}H NMR (400 MHz, $CDCl_3$) of **8i**

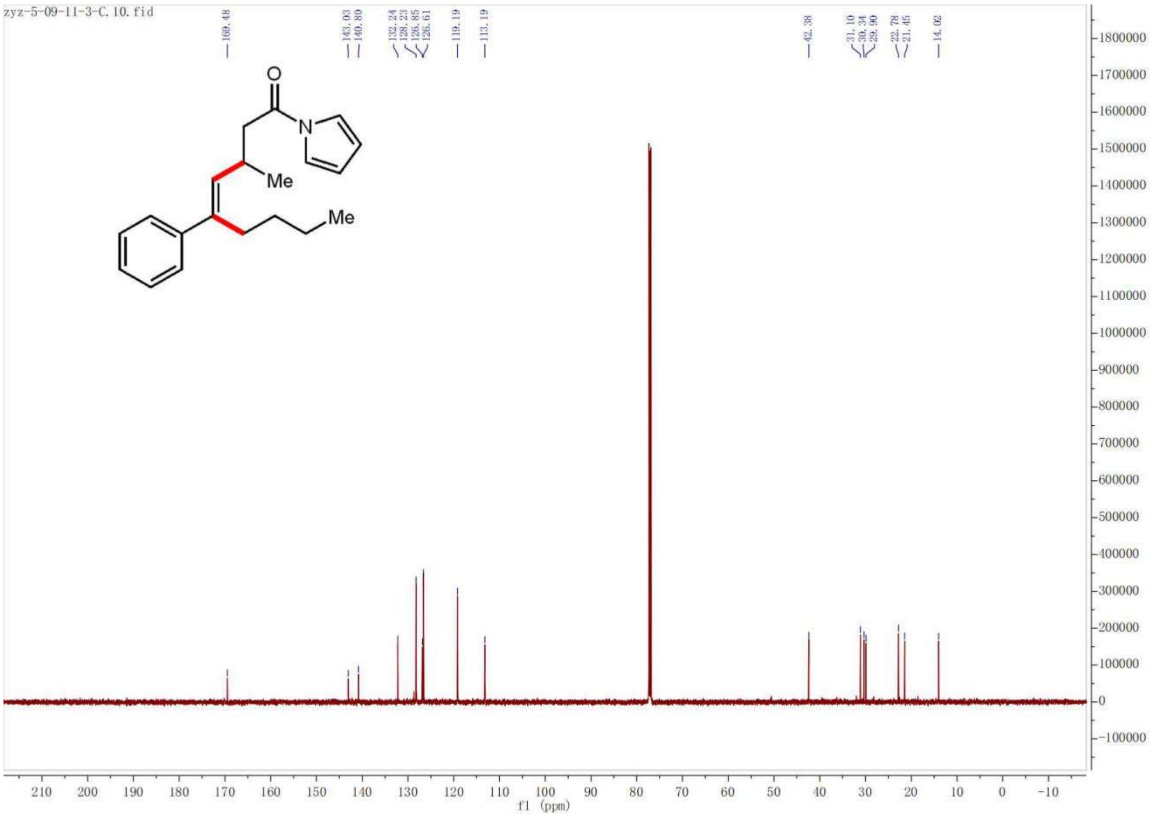

Supplementary Figure 139. ^{13}C NMR (101 MHz, $CDCl_3$) of **8i**

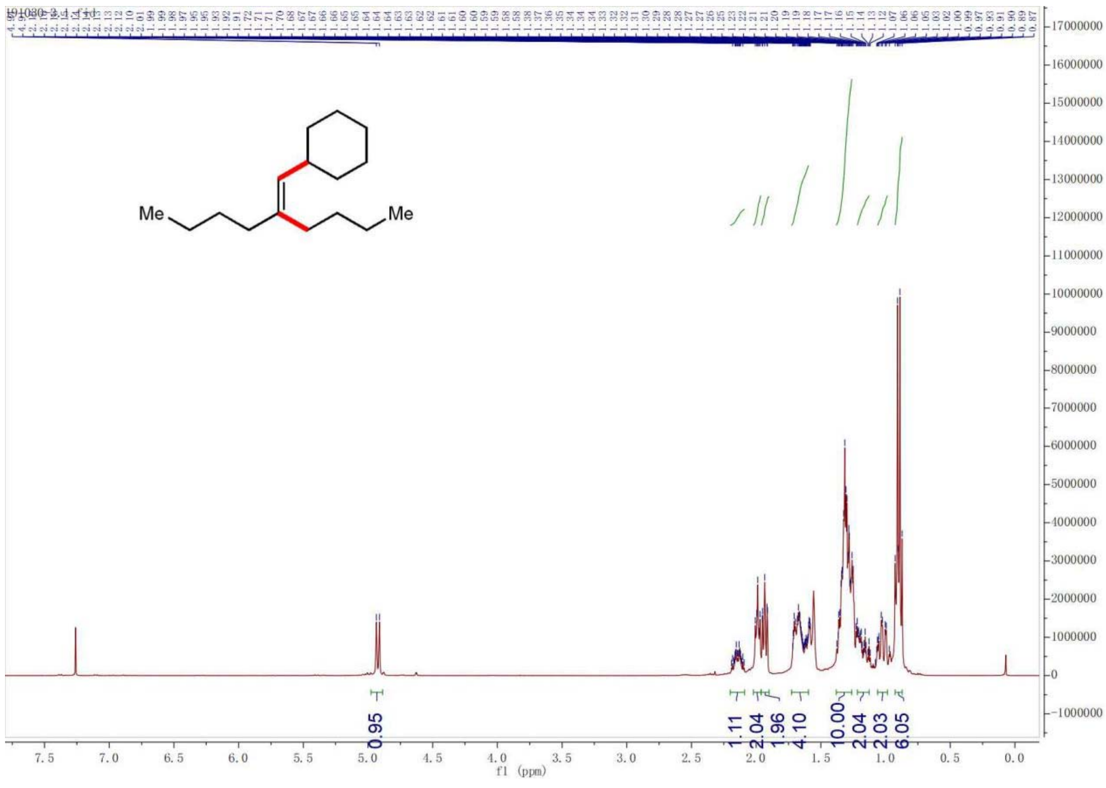

Supplementary Figure 140. ^{1}H NMR (400 MHz, $CDCl_3$) of **9a**

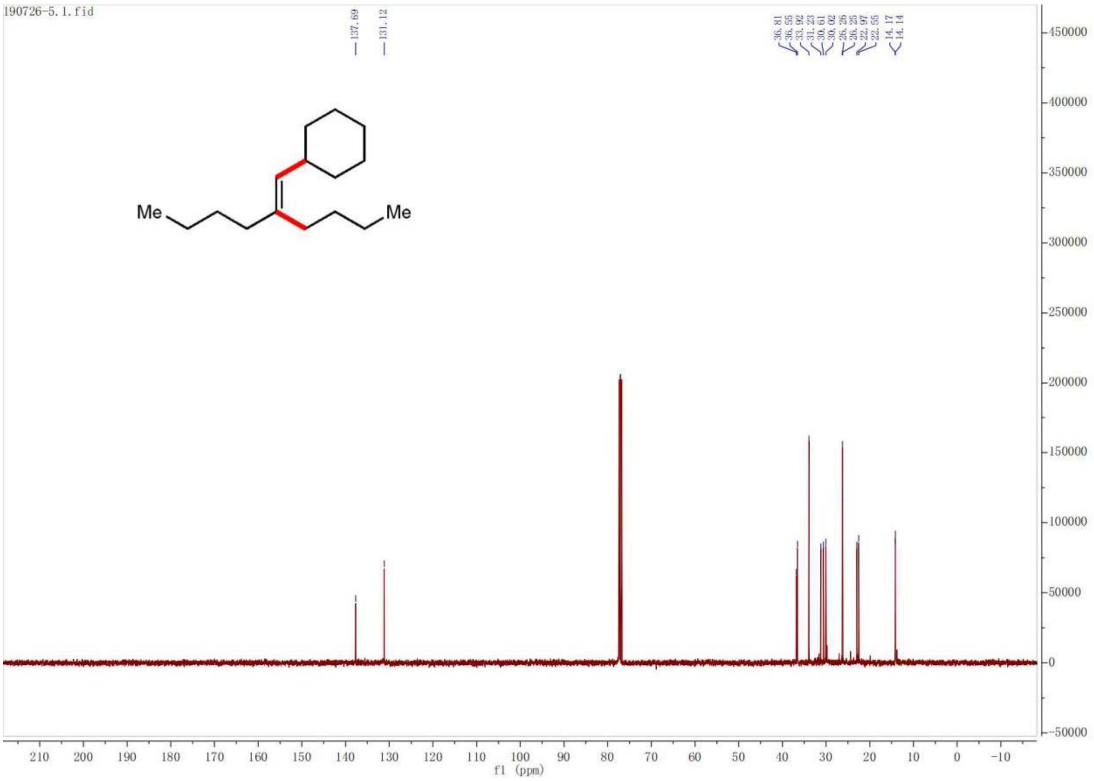

Supplementary Figure 141. ^{13}C NMR (101 MHz, $CDCl_3$) of **9a**

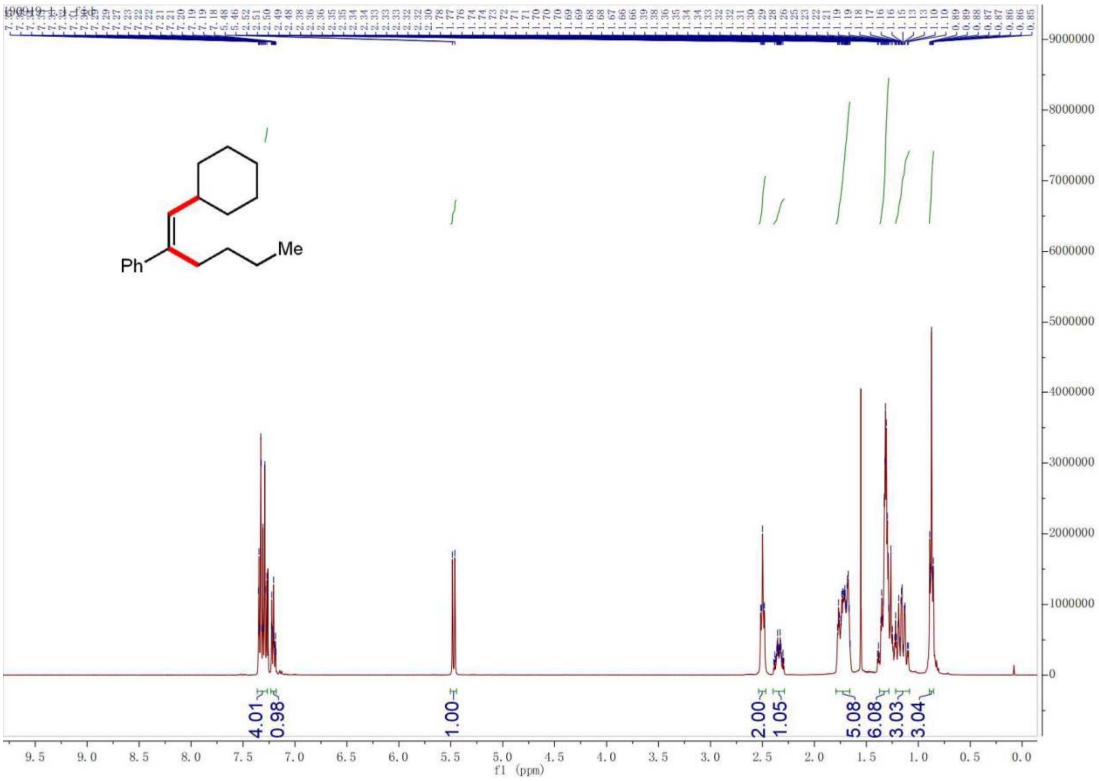

Supplementary Figure 142. ^{1}H NMR (400 MHz, $CDCl_3$) of **9b**

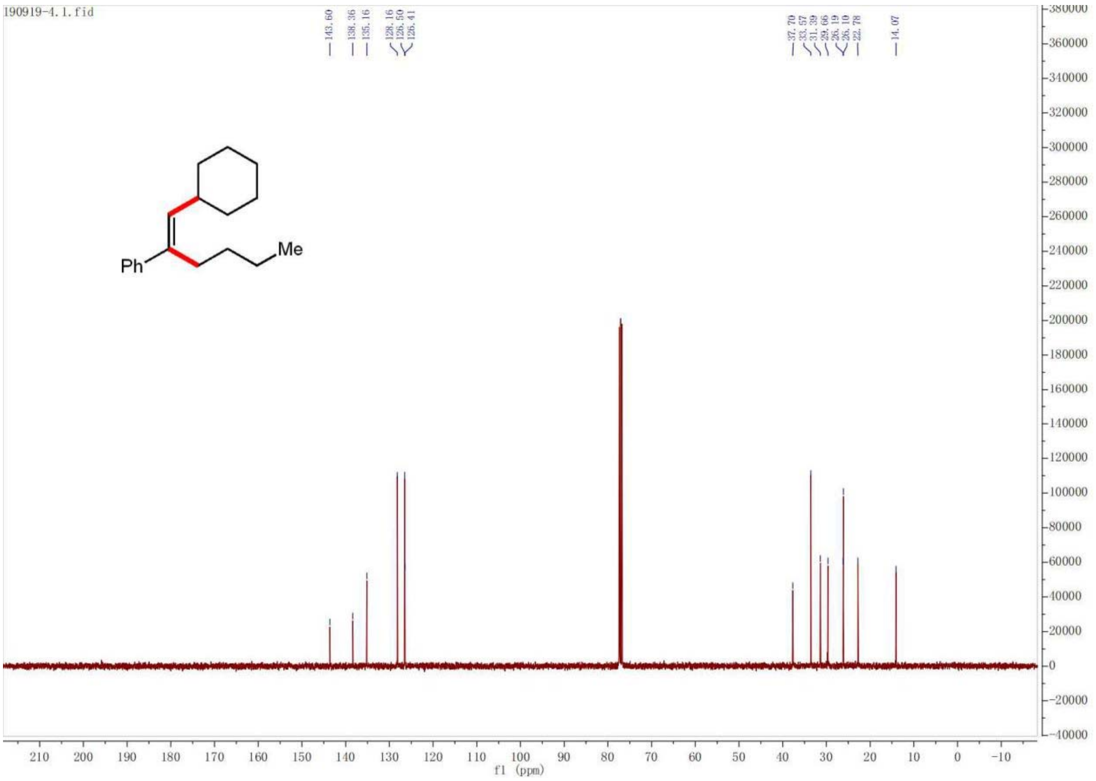

Supplementary Figure 143. ^{13}C NMR (101 MHz, $CDCl_3$) of **9b**

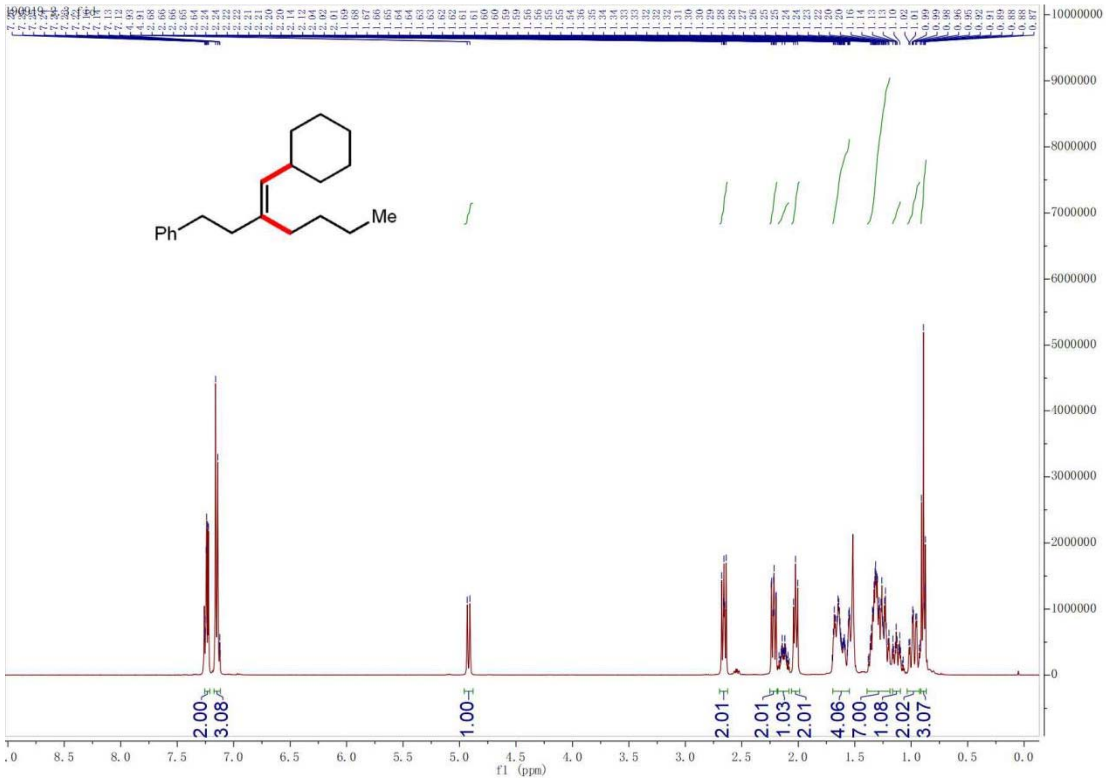

Supplementary Figure 144. ^{1}H NMR (400 MHz, $CDCl_3$) of **9c**

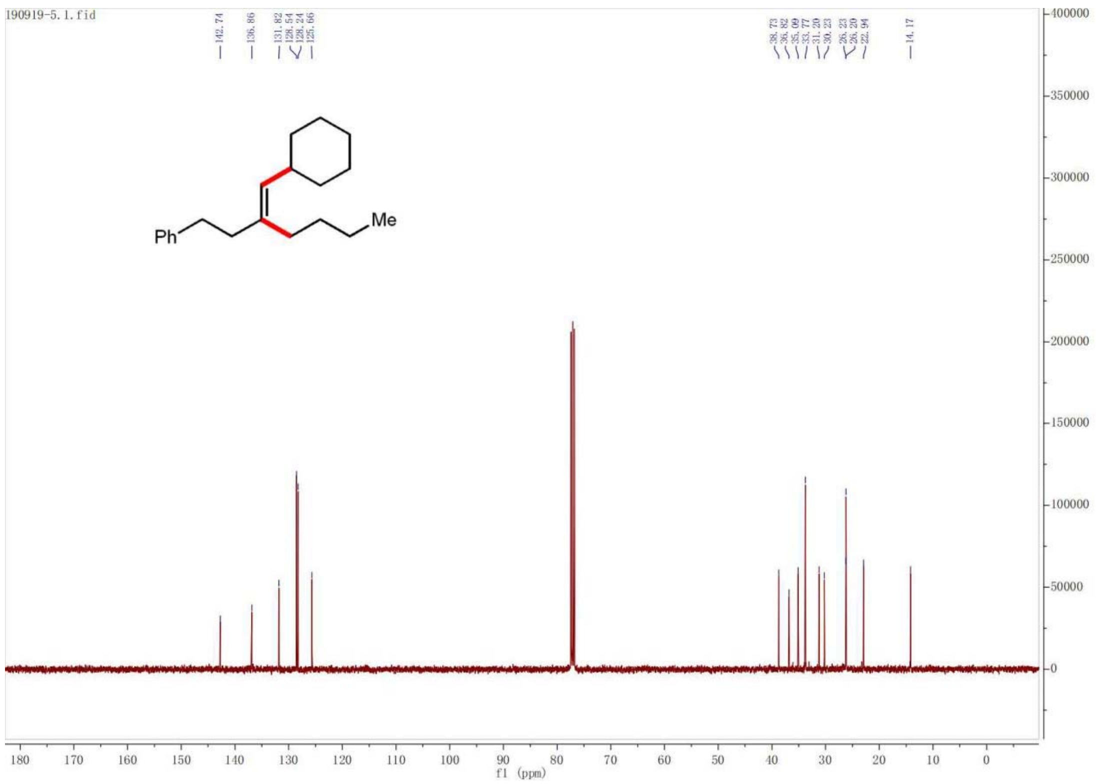

Supplementary Figure 145. ^{13}C NMR (101 MHz, $CDCl_3$) of **9c**

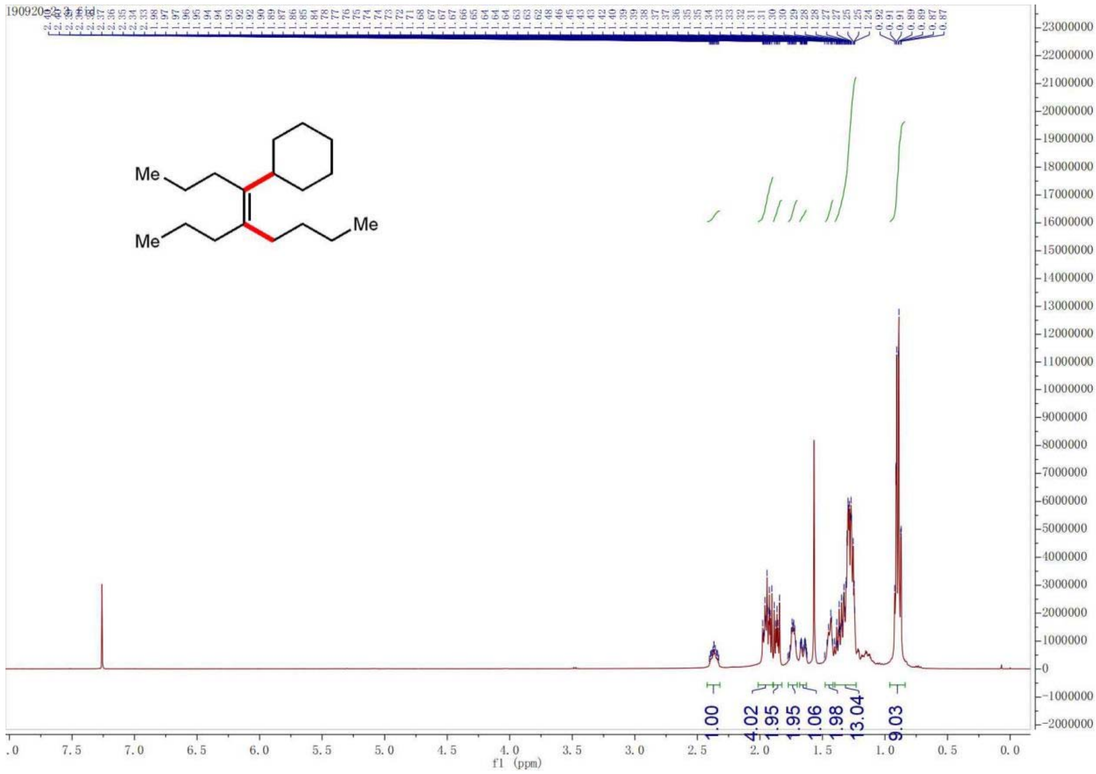

Supplementary Figure 146. ^{1}H NMR (400 MHz, $CDCl_3$) of **9d**

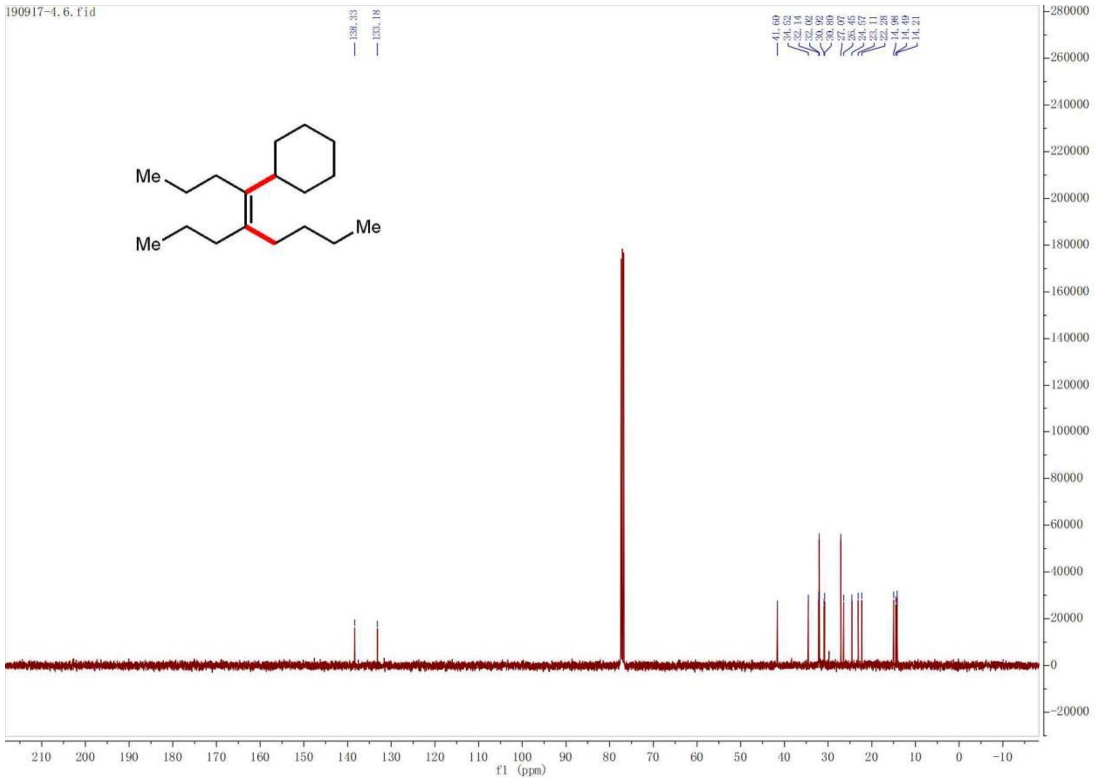

Supplementary Figure 147. ^{13}C NMR (101 MHz, $CDCl_3$) of **9d**

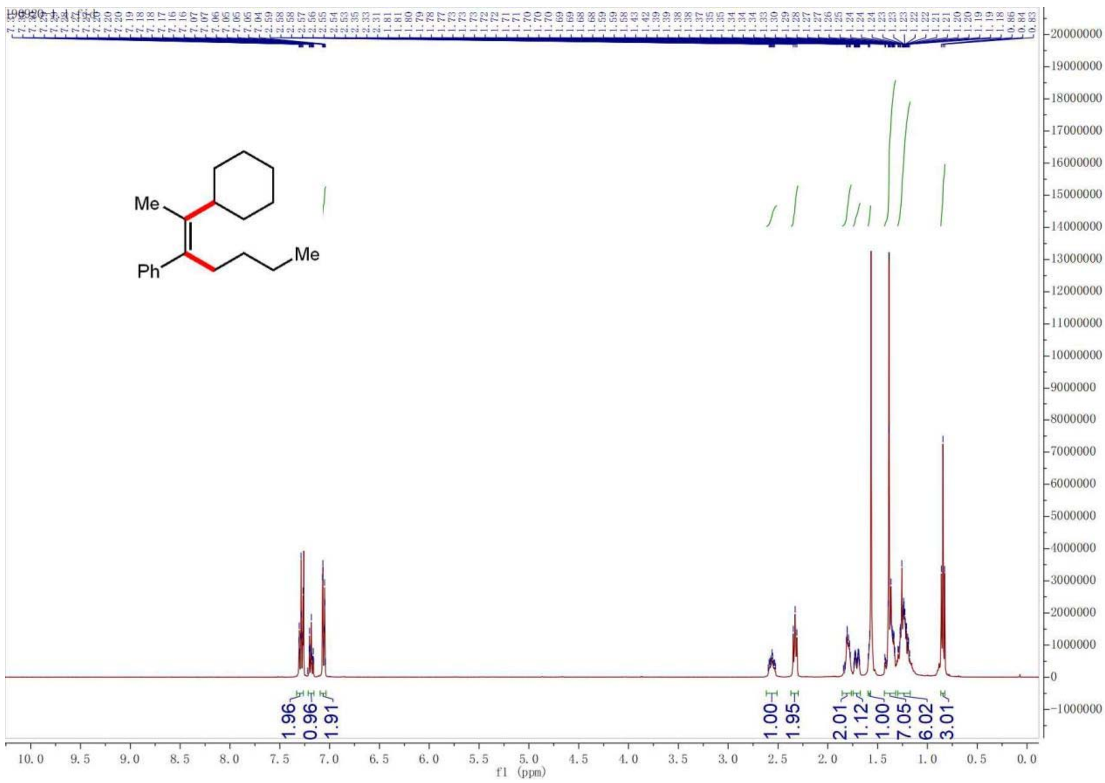

Supplementary Figure 148. ^{1}H NMR (400 MHz, $CDCl_3$) of **9e**

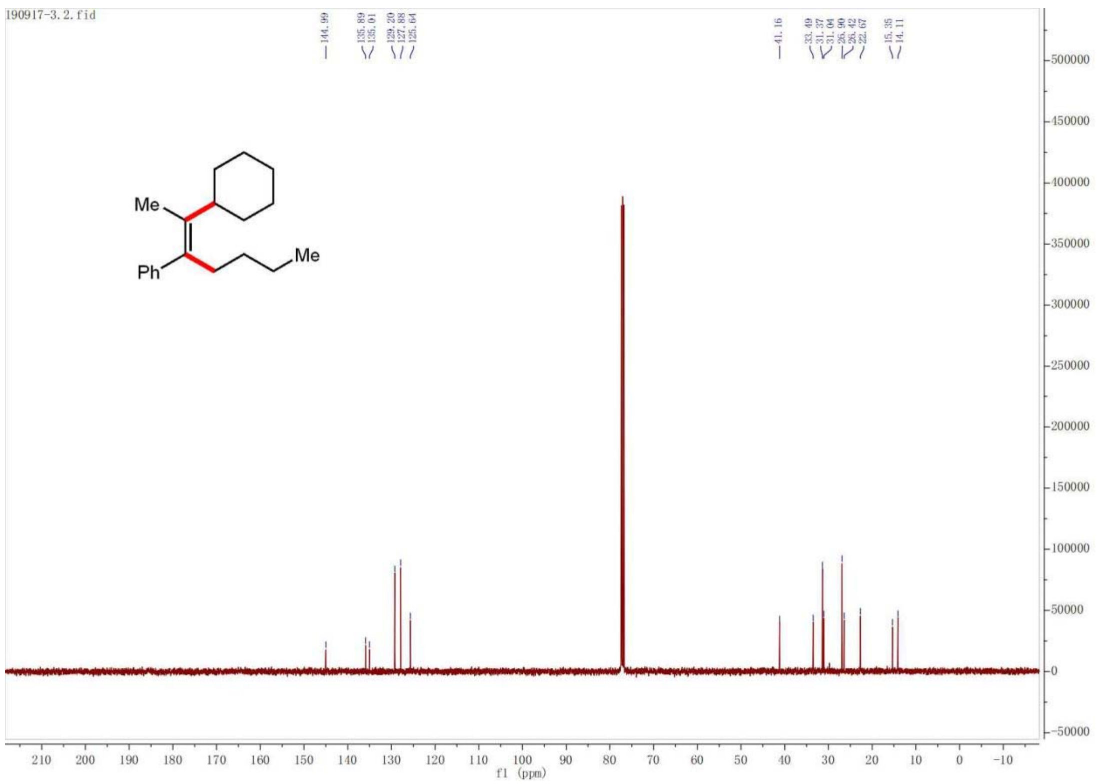

Supplementary Figure 149. ^{13}C NMR (101 MHz, $CDCl_3$) of **9e**

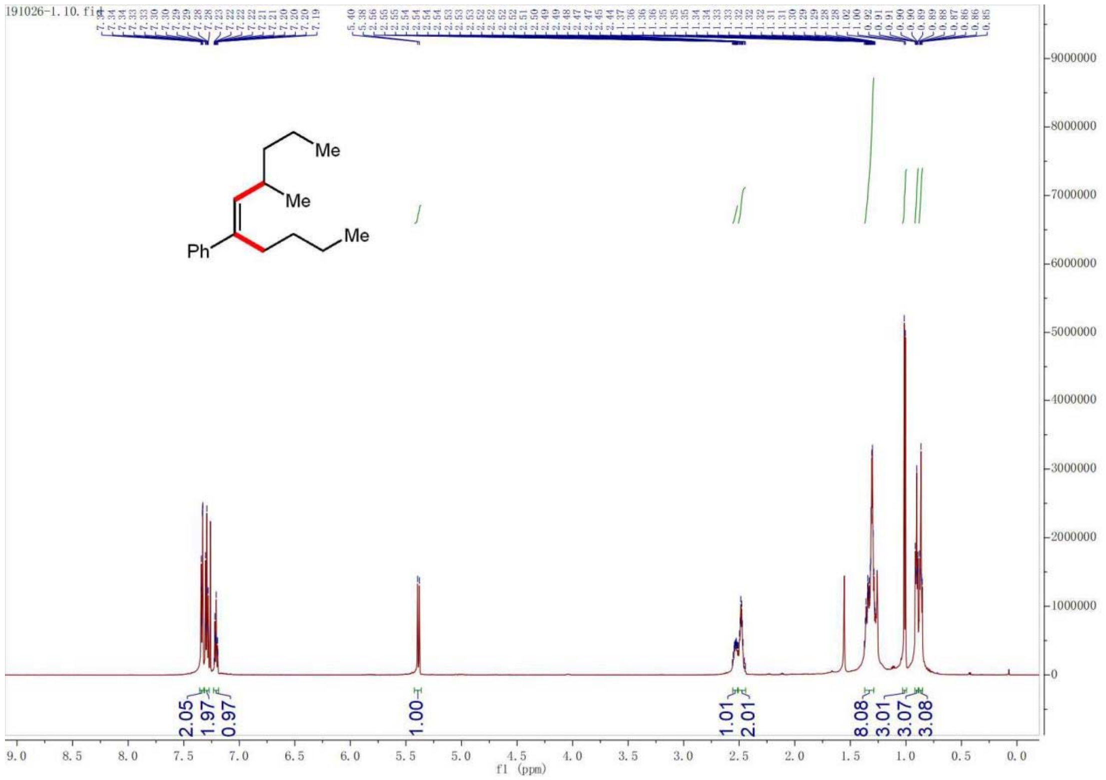

Supplementary Figure 150. ^{1}H NMR (600 MHz, $CDCl_3$) of **9f**

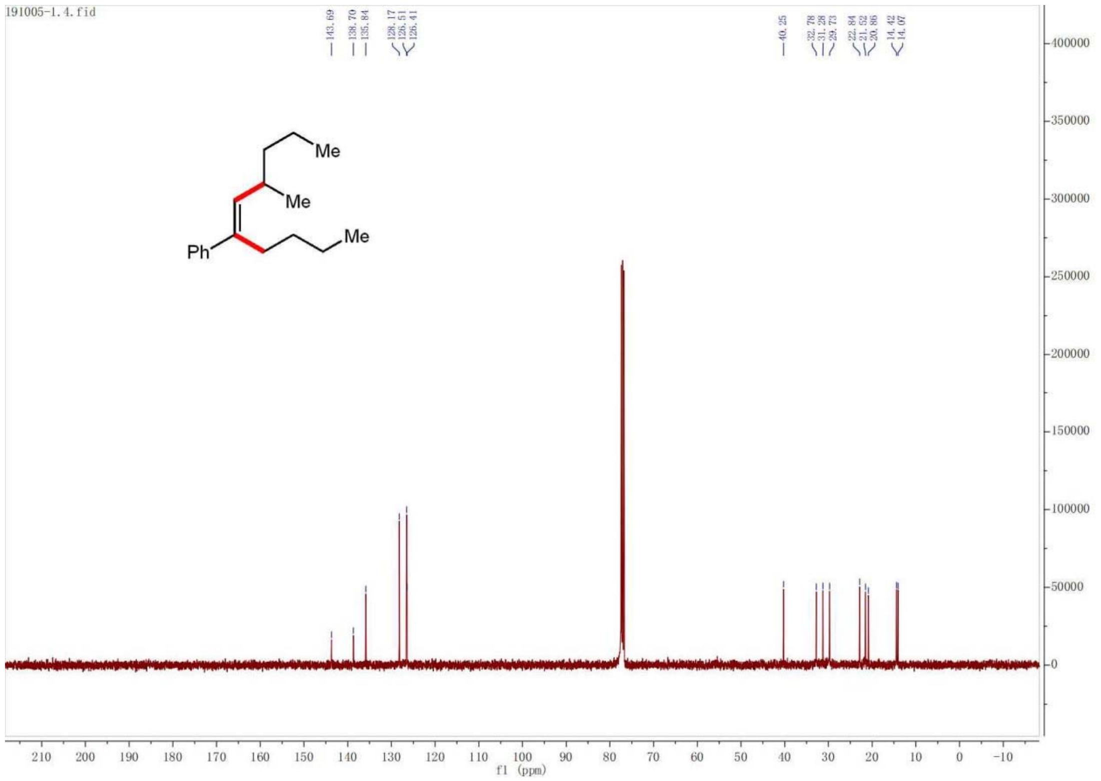

Supplementary Figure 151. ^{13}C NMR (151 MHz, $CDCl_3$) of **9f**

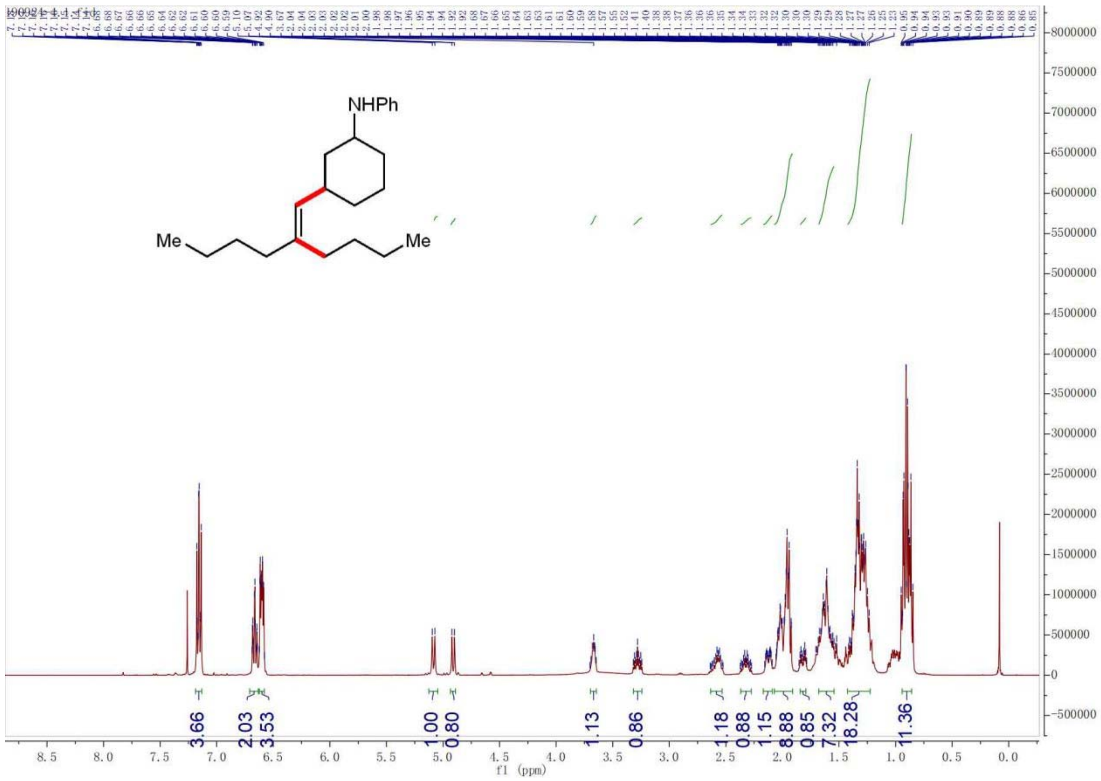

Supplementary Figure 152. ^{1}H NMR (400 MHz, $CDCl_3$) of **10**

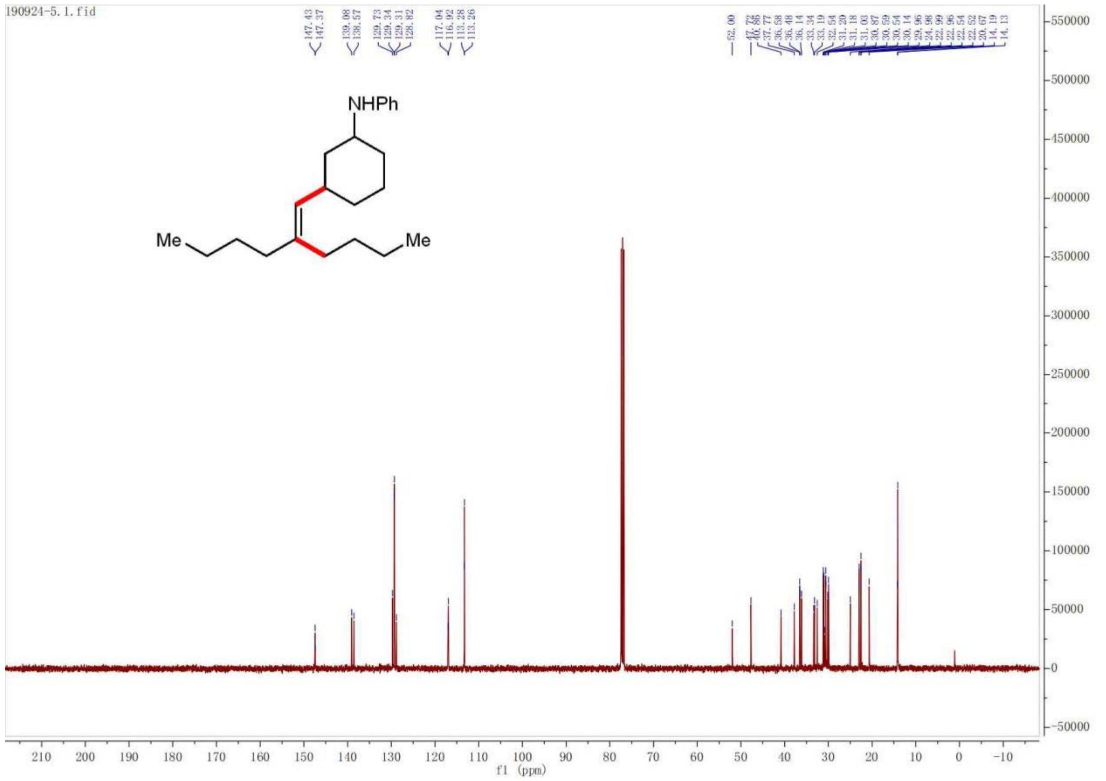

Supplementary Figure 153. ^{13}C NMR (101 MHz, $CDCl_3$) of **10**

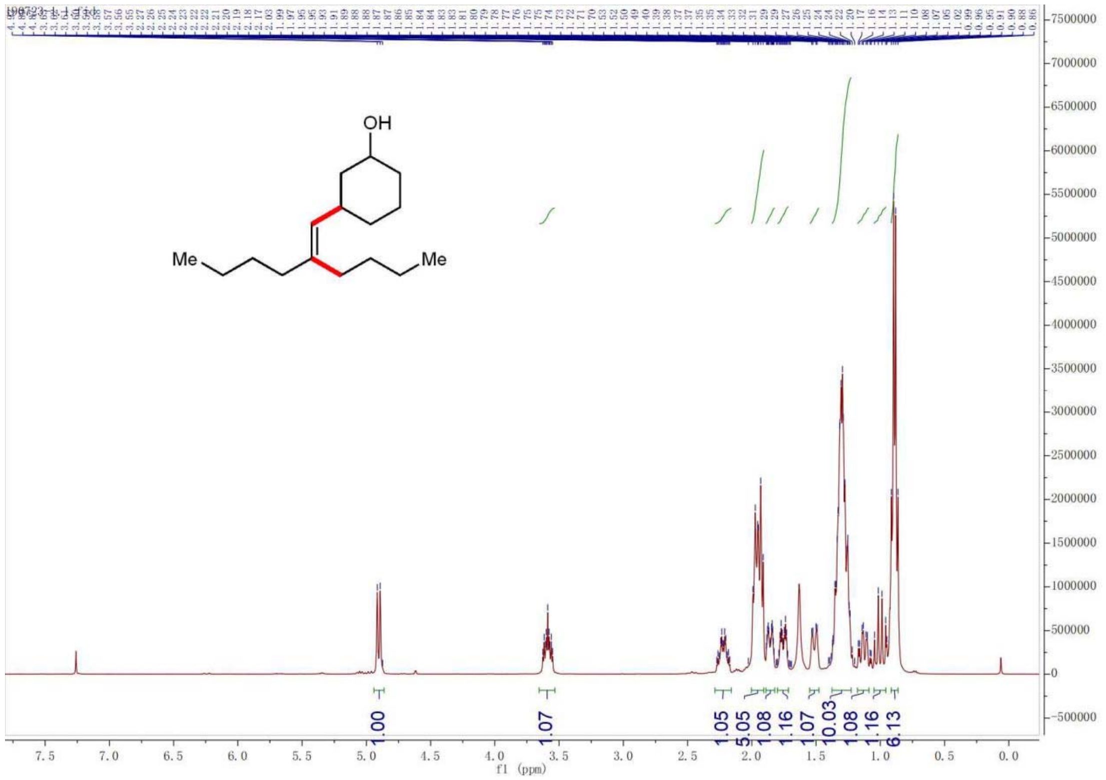

Supplementary Figure 154. ^{1}H NMR (400 MHz, $CDCl_3$) of **11**

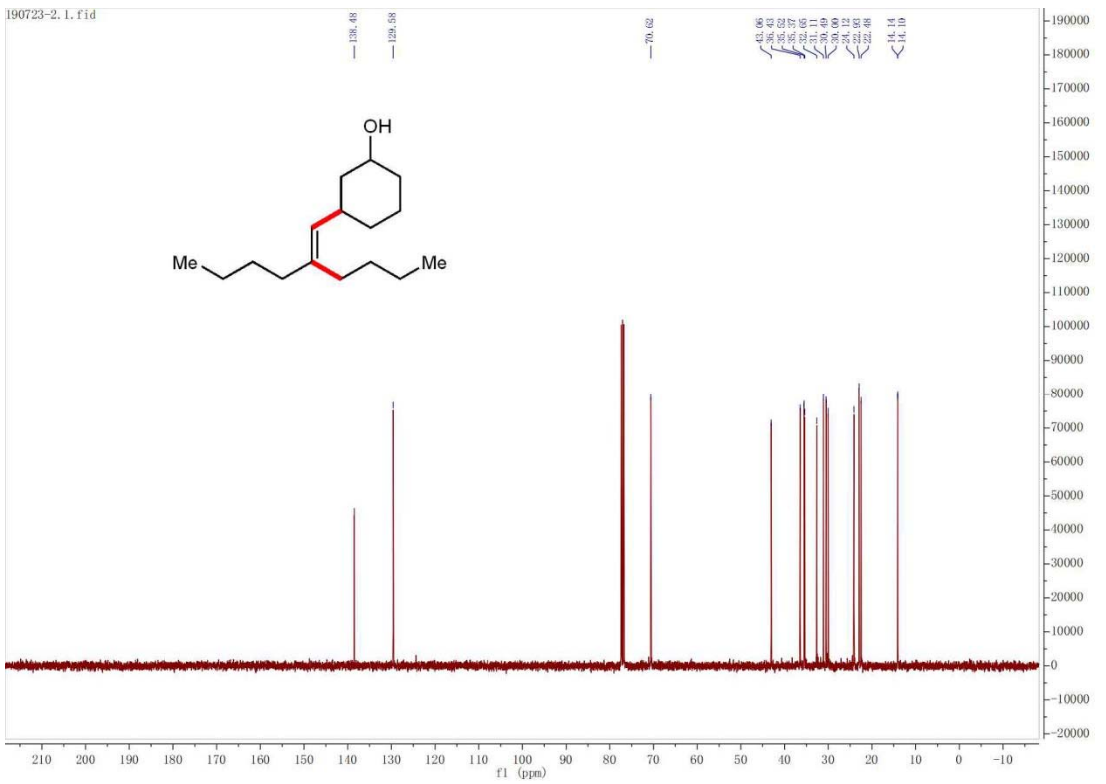

Supplementary Figure 155. ^{13}C NMR (101 MHz, $CDCl_3$) of **11**

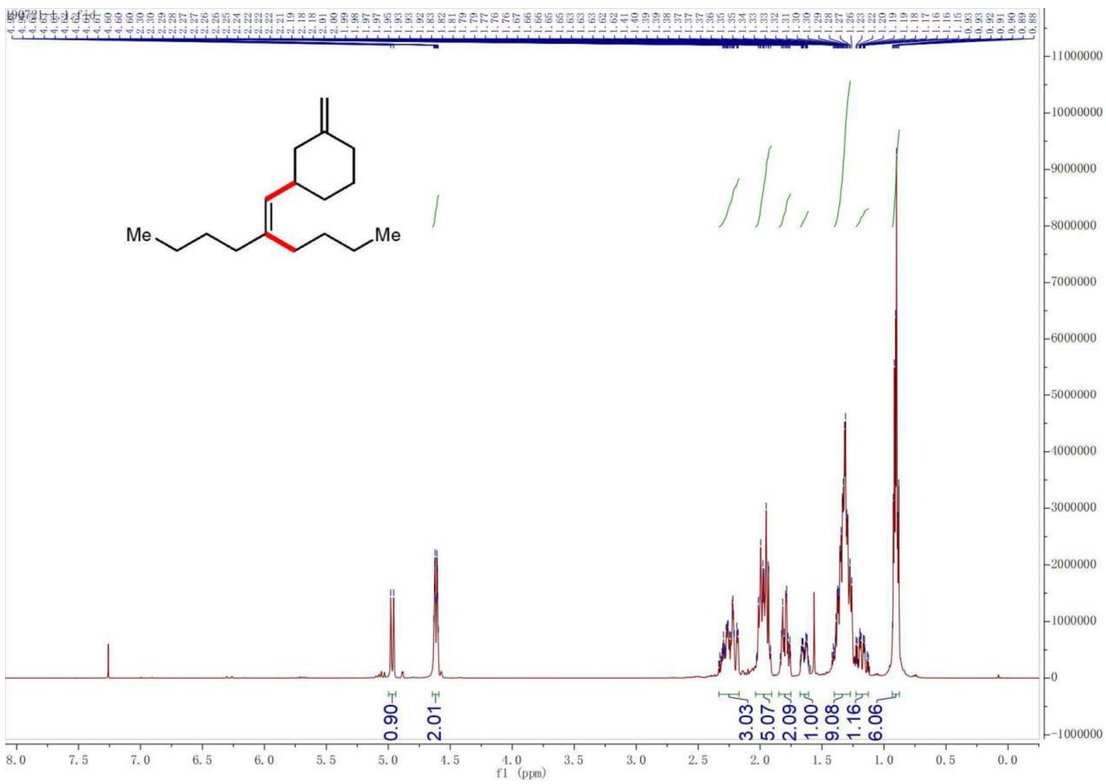

Supplementary Figure 156. ^{1}H NMR (400 MHz, $CDCl_3$) of **12**

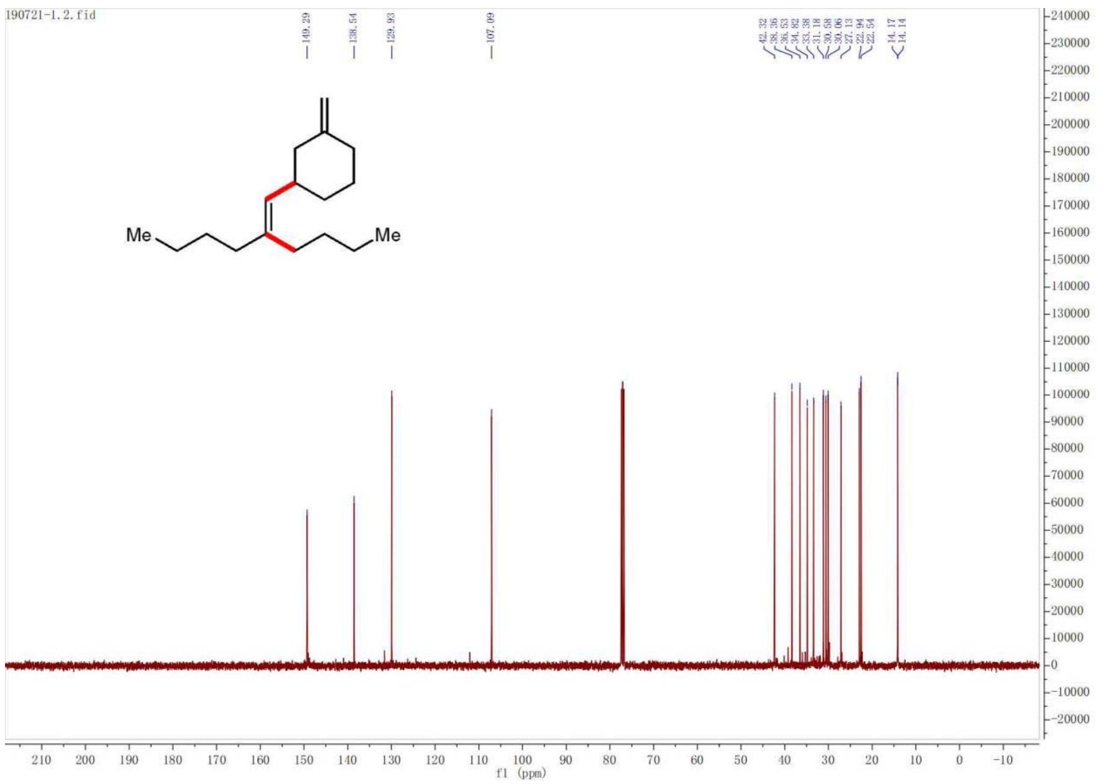

Supplementary Figure 157. ^{13}C NMR (101 MHz, $CDCl_3$) of **12**

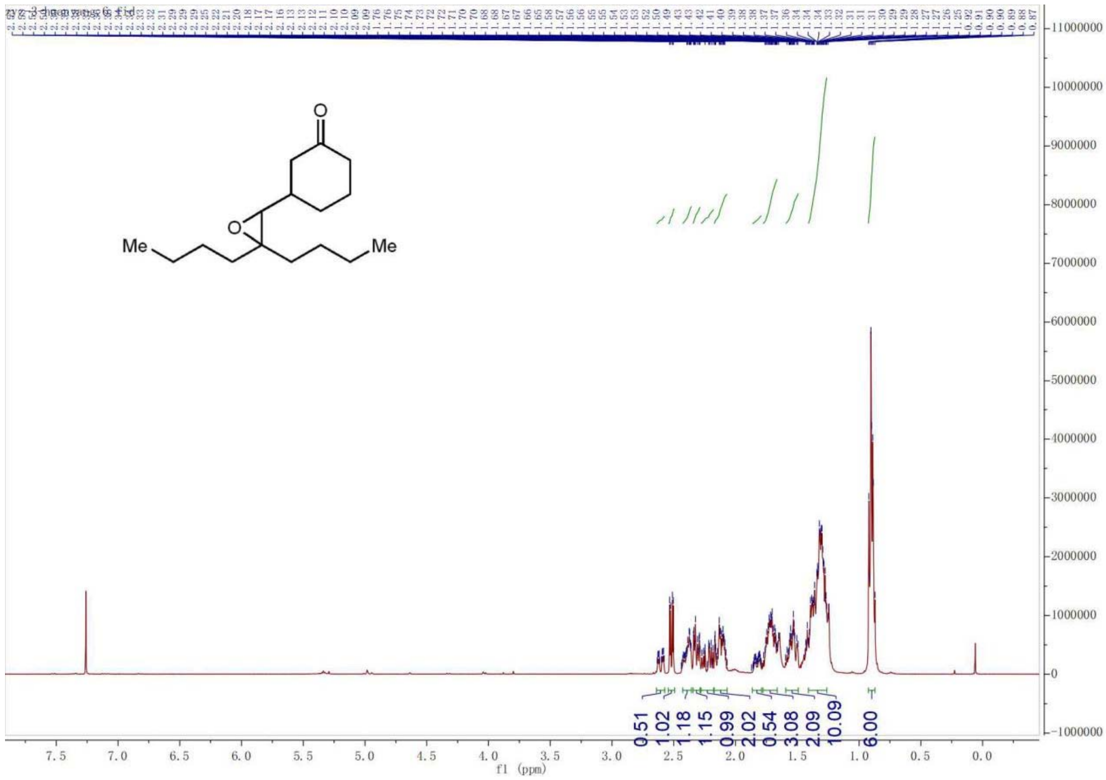

Supplementary Figure 158. ^{1}H NMR (400 MHz, $CDCl_3$) of **13**

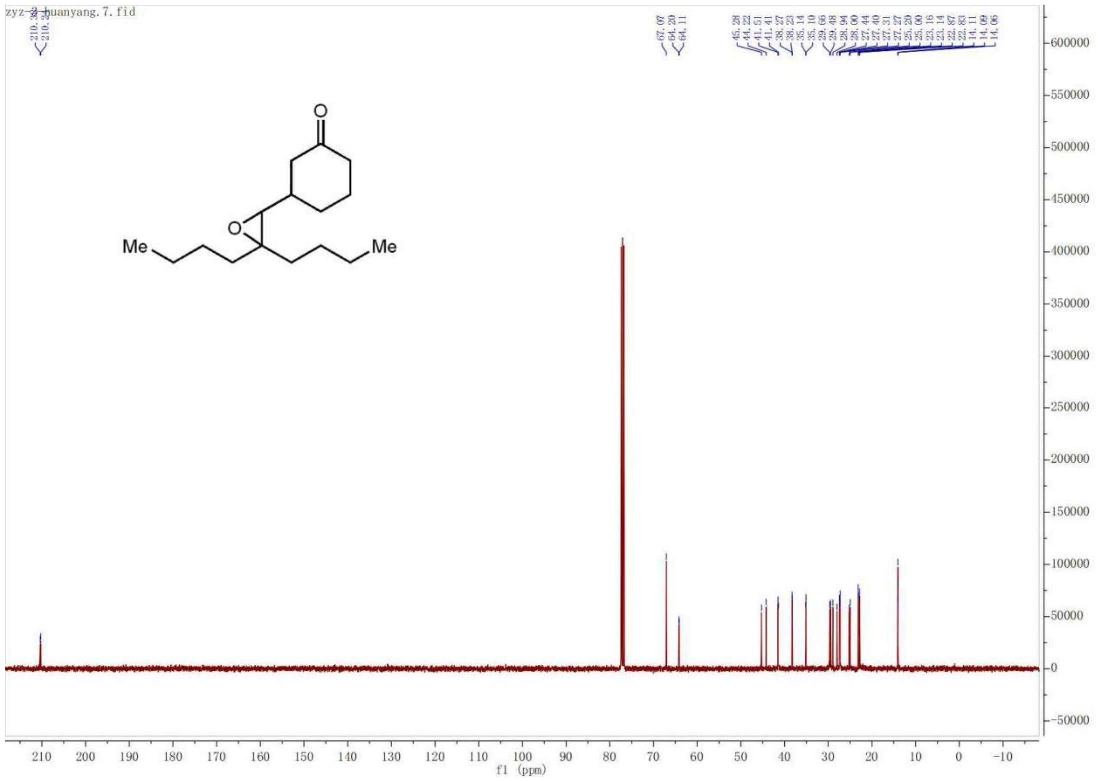

Supplementary Figure 159. ^{13}C NMR (101 MHz, $CDCl_3$) of **13**

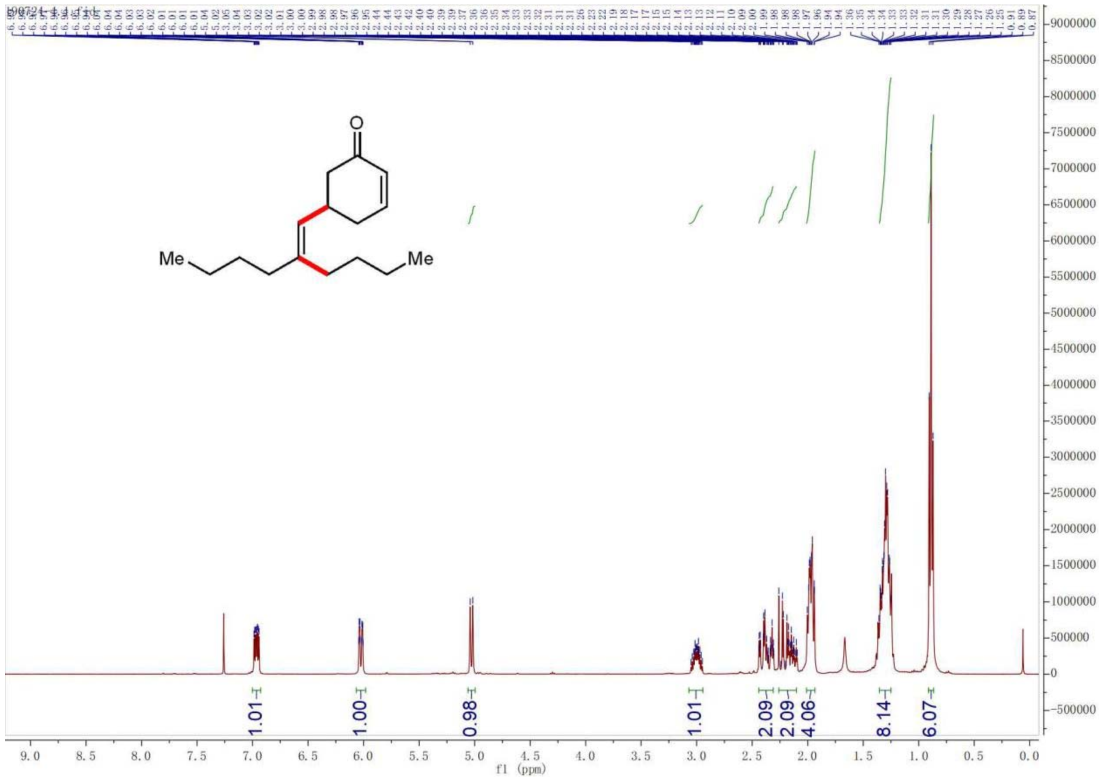

Supplementary Figure 160. ^{1}H NMR (400 MHz, $CDCl_3$) of **14**

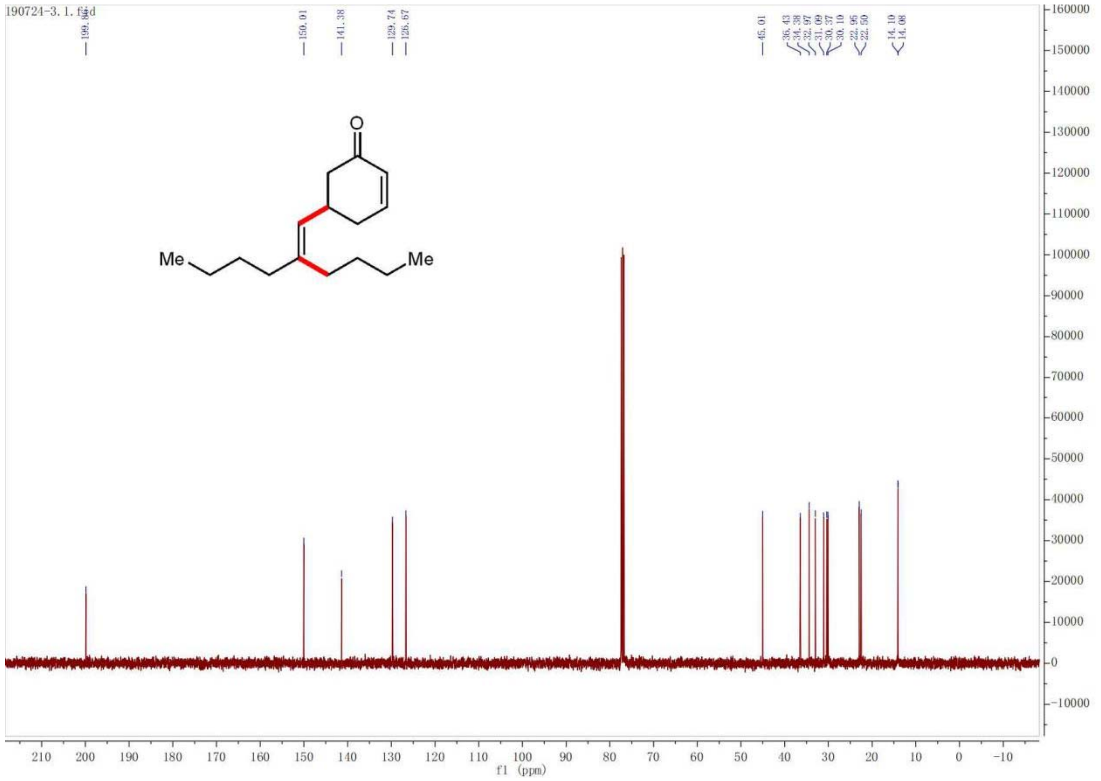

Supplementary Figure 161. ^{13}C NMR (101 MHz, $CDCl_3$) of **14**

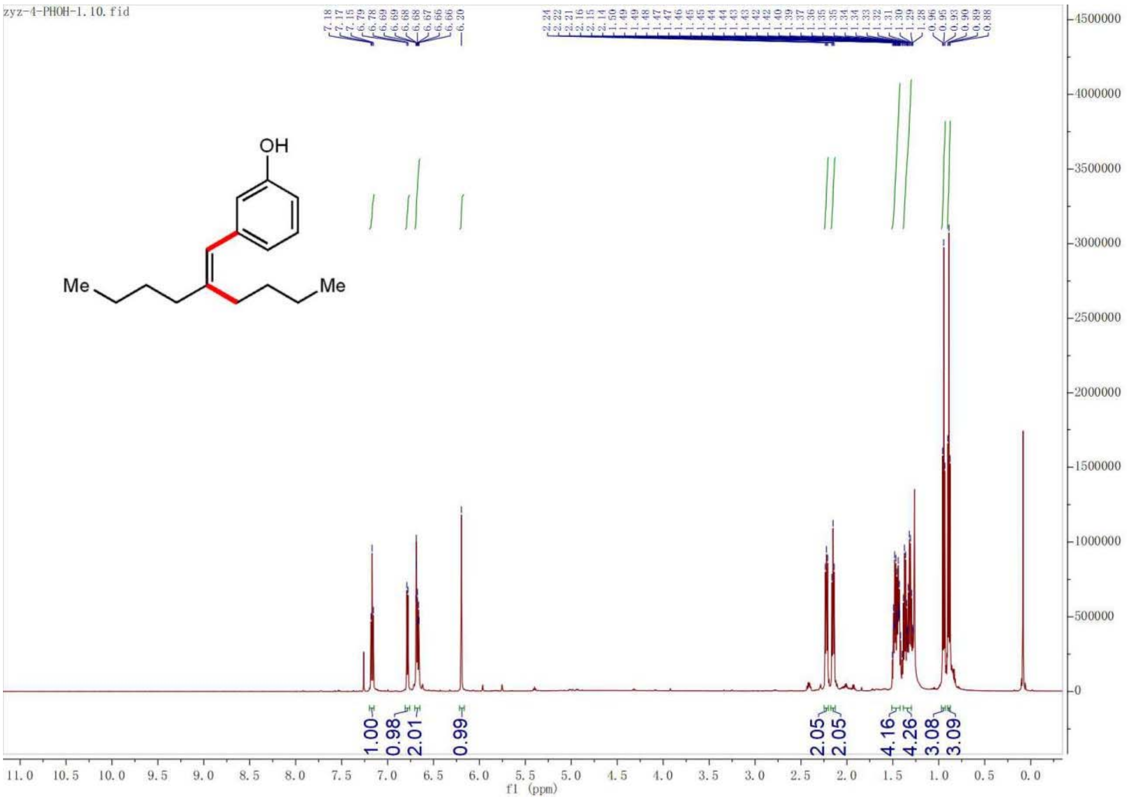

Supplementary Figure 162. ^{1}H NMR (600 MHz, $CDCl_3$) of **15**

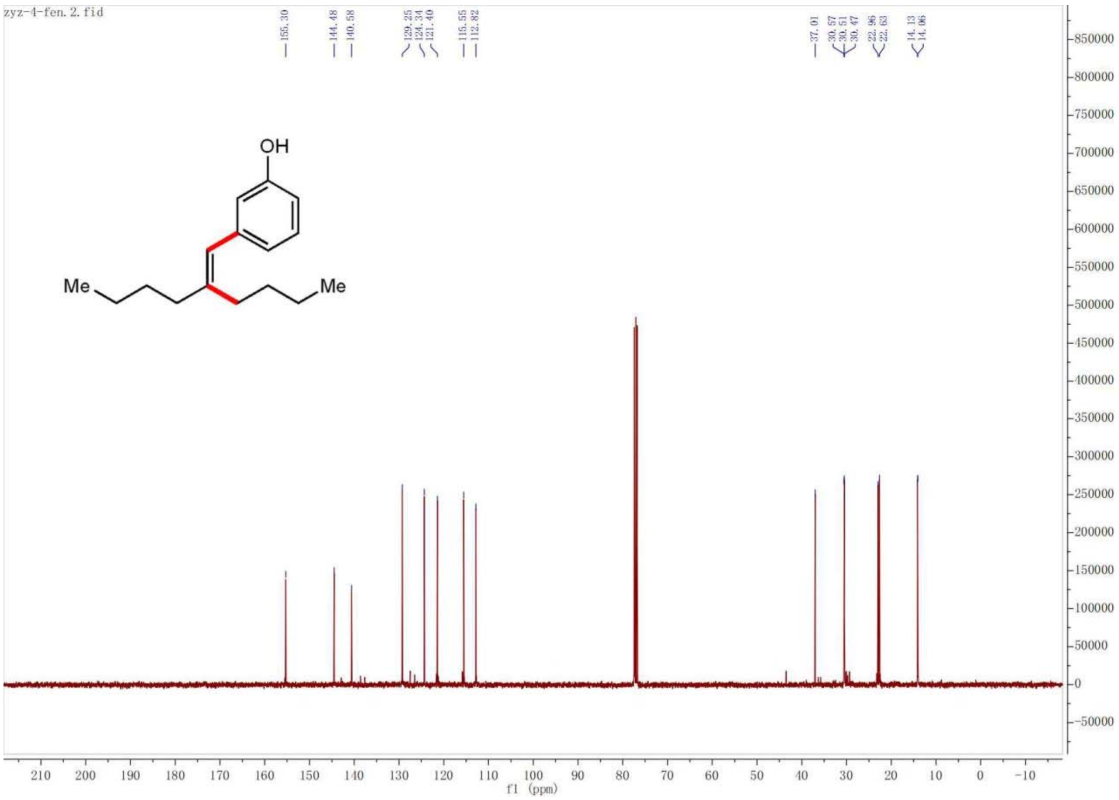

Supplementary Figure 163. ^{13}C NMR (151 MHz, $CDCl_3$) of **15**

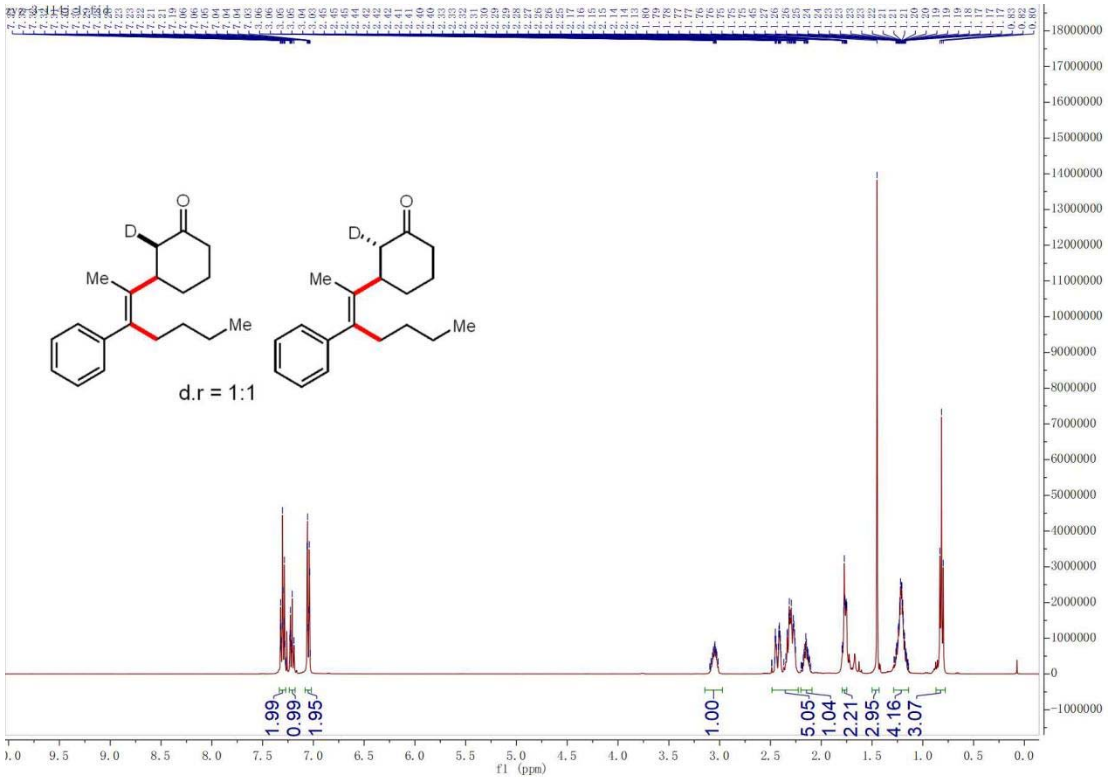

Supplementary Figure 164. ^{1}H NMR (400 MHz, $CDCl_3$) of **6a**-*d*

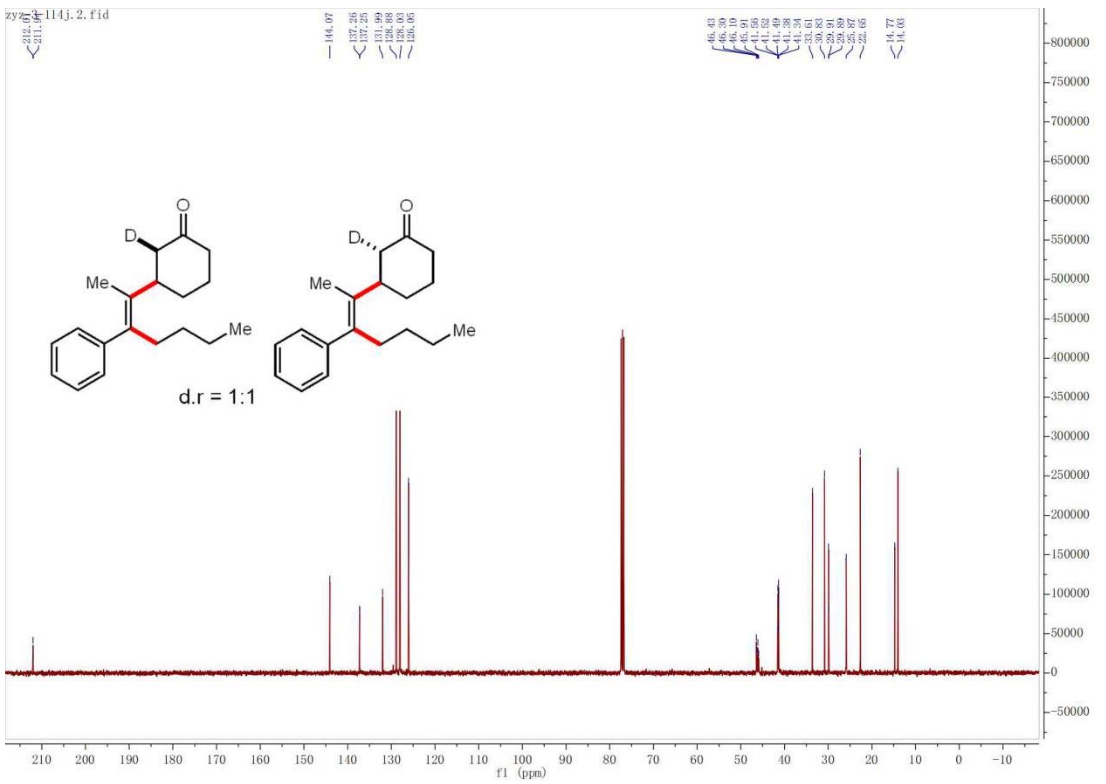

Supplementary Figure 165. ^{13}C NMR (101 MHz, $CDCl_3$) of **6a**-*d*

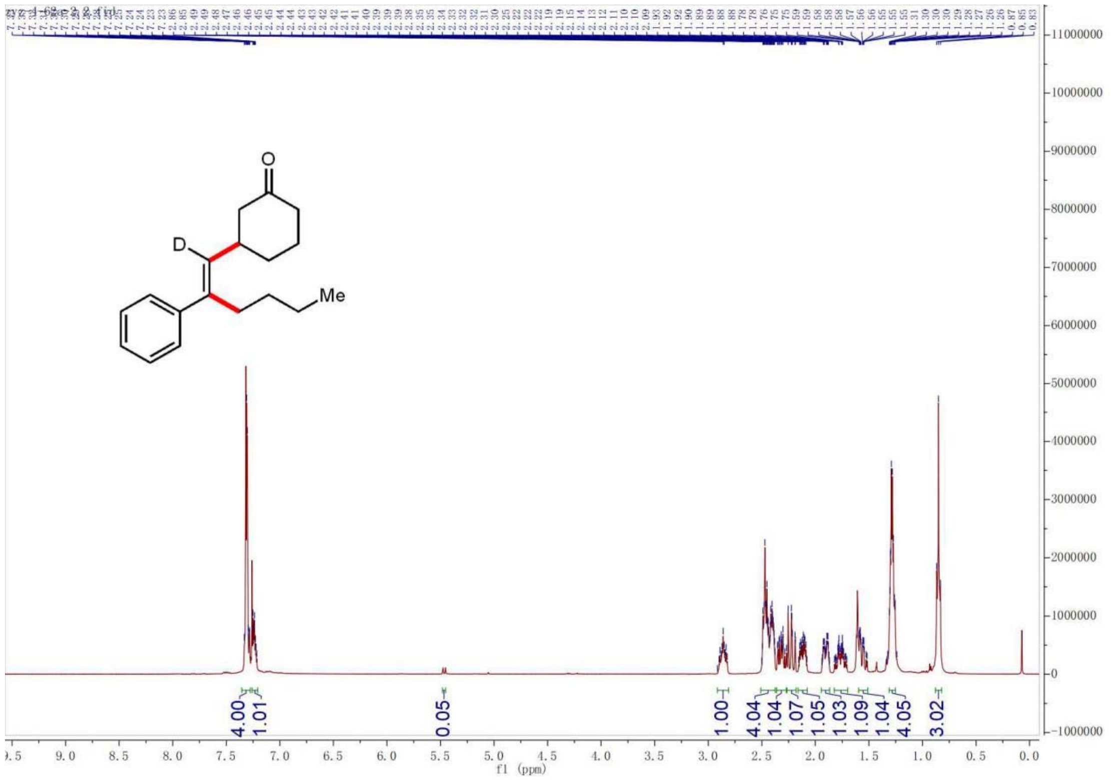

Supplementary Figure 166. ^{1}H NMR (400 MHz, $CDCl_3$) of **5a**-*d*

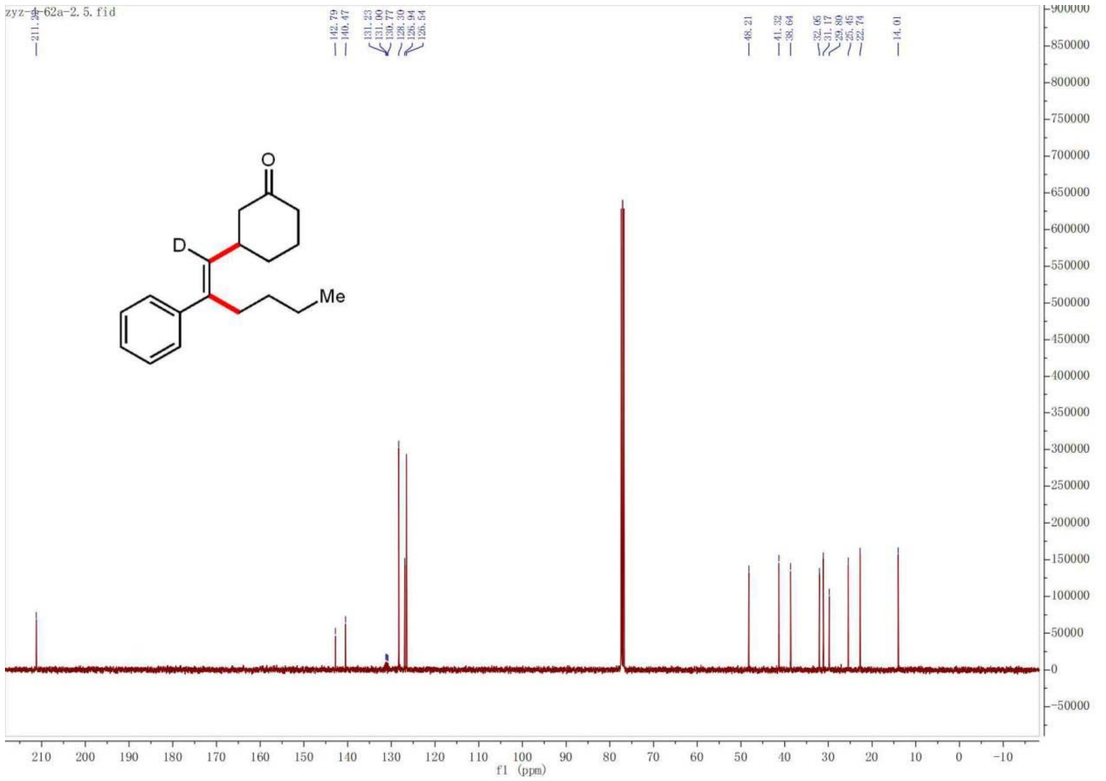

Supplementary Figure 167. ^{13}C NMR (101 MHz, $CDCl_3$) of **5a**-*d*

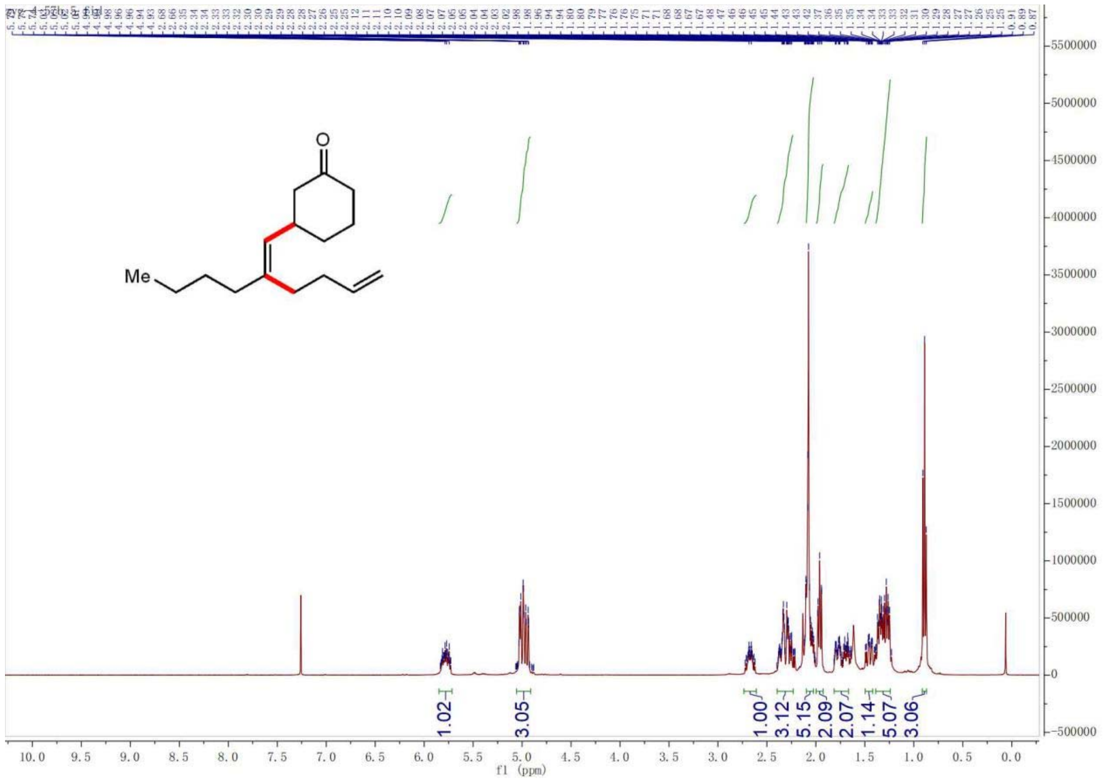

Supplementary Figure 168. ^{1}H NMR (400 MHz, $CDCl_3$) of **16**

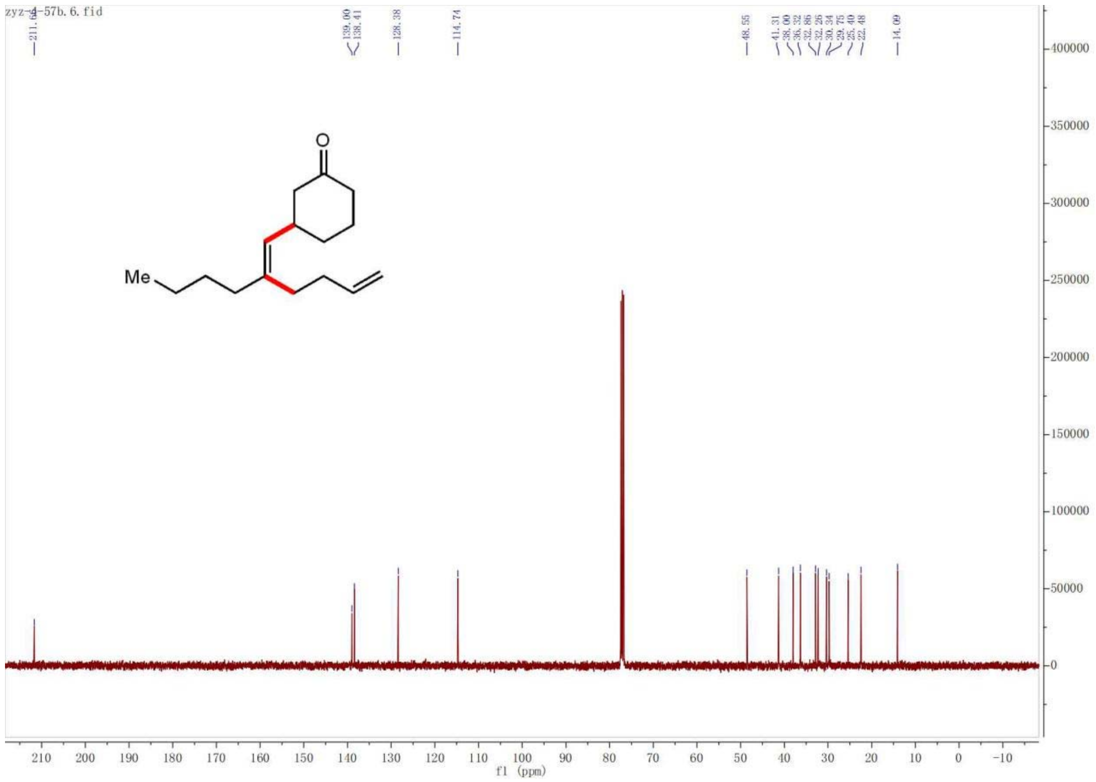

Supplementary Figure 169. ^{13}C NMR (101 MHz, $CDCl_3$) of **16**

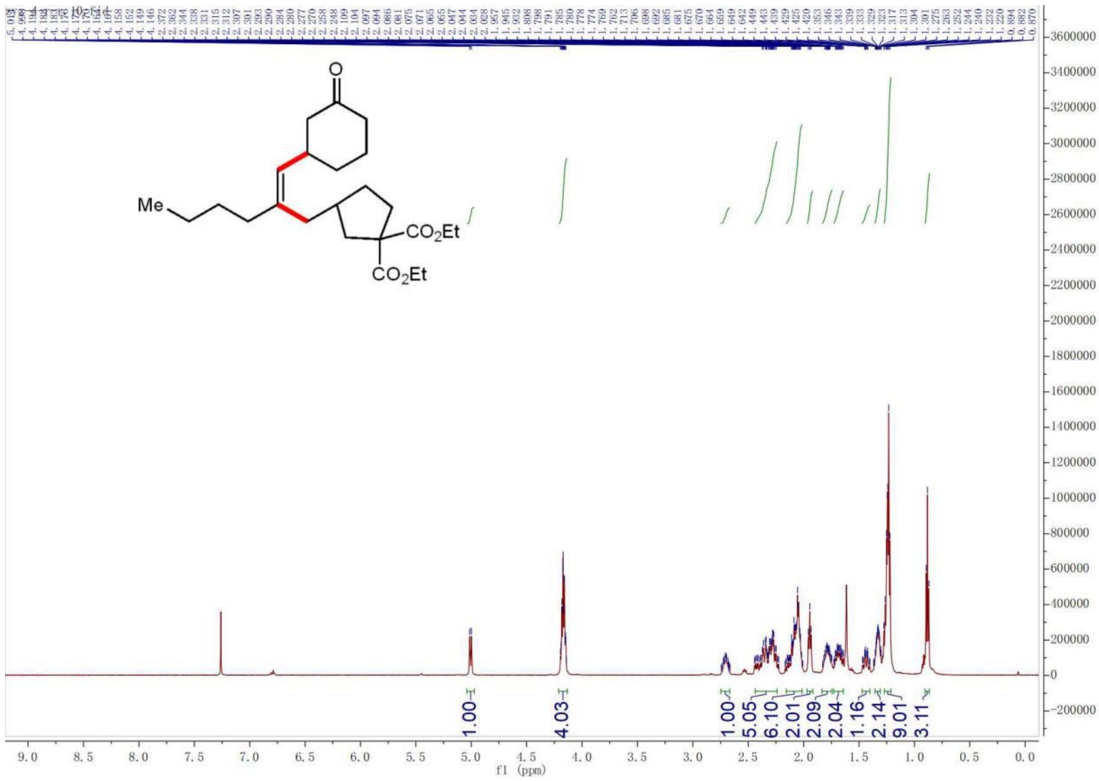

Supplementary Figure 170. ^{1}H NMR (600 MHz, $CDCl_3$) of **18**

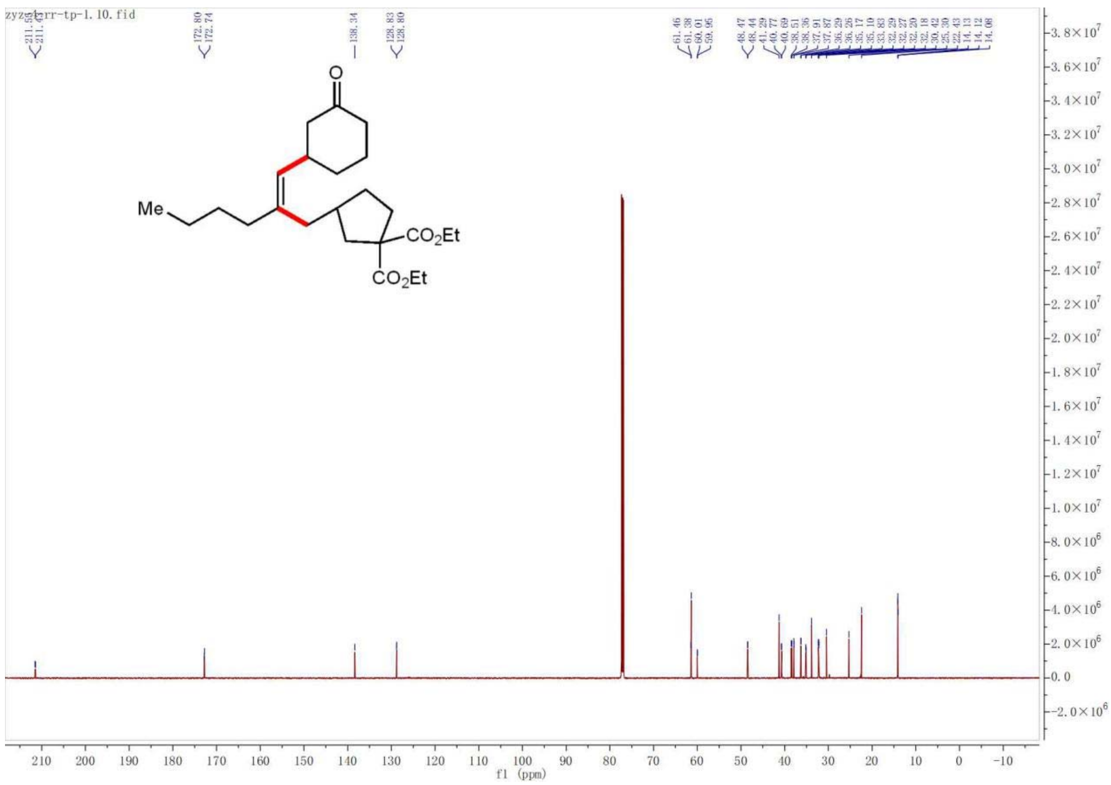

Supplementary Figure 171. ^{13}C NMR (151 MHz, $CDCl_3$) of **18**

2.5 X-ray diffraction data of 5c and 6b

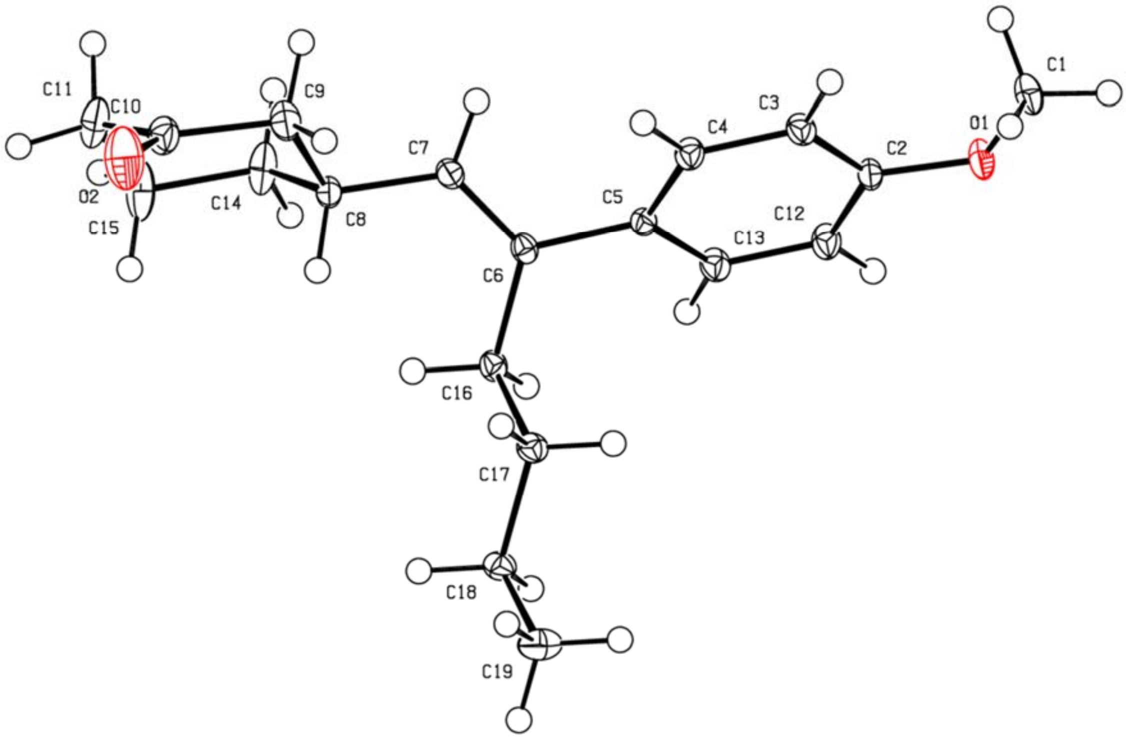

Supplementary Figure 172. X-ray diffraction data of **5c** (CCDC 1983359)

Supplementary Table 3. Crystal data and structure refinement for **5c**

Identification code	cxy0516_0m
Empirical formula	$C_{19}H_{26}O_2$
Formula weight	286.40
Temperature/K	100.0
Crystal system	triclinic
Space group	P-1
a/Å	8.6235(8)
b/Å	9.6199(9)
c/Å	10.8133(10)
α/°	71.884(3)
β/°	87.807(3)
γ/°	71.171(3)
Volume/Å^3	805.03(13)
Z	2
ρ_{calc}g/cm^3	1.182
μ/mm^{-1}	0.074
F(000)	312.0
Crystal size/mm^3	0.42 × 0.38 × 0.09
Radiation	MoKα (λ = 0.71073)
2Θ range for data collection/°	4.714 to 55.104
Index ranges	$-11 \le h \le 11$, $-12 \le k \le 12$, $-14 \le l \le 14$
Reflections collected	23631
Independent reflections	3716 [R_{int} = 0.0693, R_{sigma} = 0.0425]
Data/restraints/parameters	3716/0/193
Goodness-of-fit on F^2	1.070
Final R indexes [I>=2σ (I)]	R_1 = 0.0481, wR_2 = 0.1262

Final R indexes [all data]	$R_1 = 0.0593$, $wR_2 = 0.1334$
Largest diff. peak/hole / e Å^{-3}	0.66/-0.33

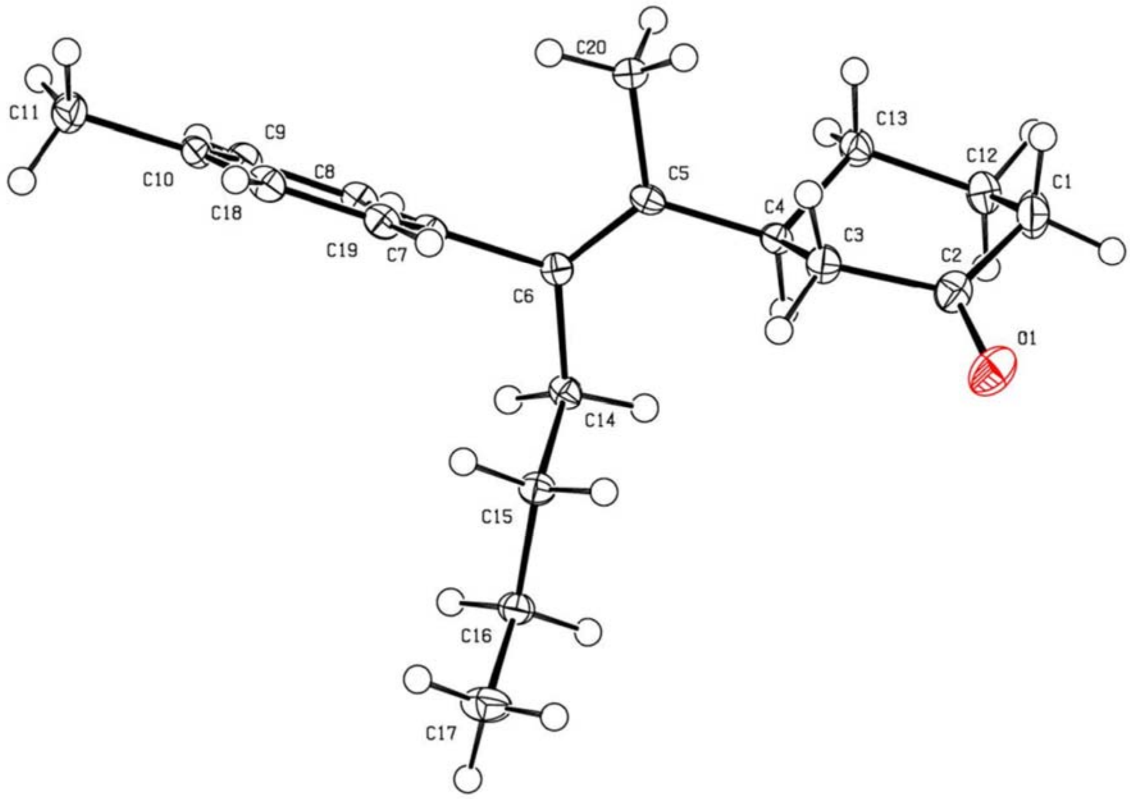

Supplementary Figure 173. X-ray diffraction data of **6b** (CCDC 1983360)

Supplementary Table 4. Crystal data and structure refinement for **6b**

Identification code	cxy0867_0m_4
Empirical formula	$C_{20}H_{28}O$
Formula weight	284.42
Temperature/K	100
Crystal system	monoclinic
Space group	$P2_1/n$
a/Å	12.7249(8)
b/Å	7.3595(4)
c/Å	18.4607(11)
α/°	90

β/°	103.480(2)
γ/°	90
Volume/Å^3	1681.20(17)
Z	4
ρ_{calc}g/cm^3	1.124
μ/mm^{-1}	0.067
F(000)	624.0
Crystal size/mm^3	0.38 × 0.37 × 0.29
Radiation	MoKα (λ = 0.71073)
2Θ range for data collection/°	5.982 to 55.084
Index ranges	-16 ≤ h ≤ 16, 0 ≤ k ≤ 9, 0 ≤ l ≤ 24
Reflections collected	3855
Independent reflections	3855 [R_{int} = ?, R_{sigma} = 0.0477]
Data/restraints/parameters	3855/0/218
Goodness-of-fit on F^2	1.058
Final R indexes [I>=2σ (I)]	R_1 = 0.0630, wR_2 = 0.1637
Final R indexes [all data]	R_1 = 0.0787, wR_2 = 0.1780
Largest diff. peak/hole / e Å^{-3}	0.43/-0.31

3. Supplementary References

1. Revol, G., McCallun, T., Morin, M., Gagosz, F. & Barriault, L. Photoredox Transformations with Dimeric Gold Complexes. *Angew. Chem. Int. Ed.* **52**, 13342-13345 (2013).

2. McTiernan, C. D., Morin, M., McCallum, T., Scaiano, J. C. & Barriault, L. Polynuclear Gold (I) Complexes in Photoredox Catalysis: Understanding Their Reactivity through Characterization and Kinetic Analysis. *Catal. Sci. Technol.* **6**, 201-207 (2016).

3. Chierchia, M., Xu, P.-L., Lovinger, G. J. & Morken, J. P. Enantioselective Radical Addition/Cross-Coupling of Organozinc Reagents, Alkyl Iodides, and Alkenyl Boron Reagents. *Angew. Chem. Int. Ed*. **58**, 14245-14249 (2019).

4. Huihui, K.M., Shrestha, R. & Weix, D. Nickel-Catalyzed Reductive Conjugate Addition of Primary Alkyl Bromides to Enones to Form Silyl Enol Ethers. *Org. Lett*. **19**, 340-343 (2017).

5. Garcialacuna, J., Dominguez, G., Blancourgoiti, J. & Perezcastells, J. Cobalt Octacarbonyl-Catalyzed Scalable Alkyne Cyclotrimerization and Crossed [2 + 2 + 2]-Cycloaddition Reaction in a Plug Flow Reactor. *Org. Lett.* **20**, 5219-5223 (2018).

6. Kakeya, M., Fujihara, T., Kasaya, T. & Nagasawa, A. Dinuclear Niobium(III) Complexes $[\{NbCl_2(L)\}_2(\mu\text{-}Cl)_2(\mu\text{-}L)]$ (L = Tetrahydrothiophene, Dimethyl Sulfide): Preparation, Molecular Structures, and the Catalytic Activity for the Regioselective Cyclotrimerization of Alkynes. *Organometallics* **25**, 4131-4137 (2006).

7. Prinsell, M. R., Everson, D. A. & Weix, D. J. Nickel-Catalyzed, Sodium Iodide-Promoted Reductive Dimerization of Alkyl Halides, Alkyl Pseudohalides, and Allylic Acetates. *Chem. Commun.* **46**, 5743-5745 (2010).
